# The European BestAgeing Study on microRNA candidates reveals distinct signatures with diagnostic and prognostic potential in cardiovascular disease

**DOI:** 10.1186/s12916-025-04502-3

**Published:** 2025-11-28

**Authors:** Christoph Reich, Elham Kayvanpour, Farbod Sedaghat-Hamedani, Ali Amr, Jan Haas, Kai Ueltzhöffer, Mario Plebani, Andrea Padoan, Lars Lind, Bertil Lindahl, Lorenzo Monserrat, Andres Metspalu, Maris Alver, Tarmo Annilo, Alexander Parkhomenko, Sergey Kozhukhov, Tanja Weis, Hugo Katus, Norbert Frey, Andreas Keller, Benjamin Meder

**Affiliations:** 1 Department of Internal Medicine III, University of Heidelberg, Im Neuenheimer Feld 410, Heidelberg, 69120 Germany; 2 Precision Digital Health Unit of the Department of Internal Medicine III, University of Heidelberg, Heidelberg, Germany; 3 Informatics for Life, Heidelberg, Germany; 4 German Center for Cardiovascular Research (DZHK), Heidelberg, Germany; 5 Computational Genomics and System Genetics, German Cancer Research Center (DKFZ), Heidelberg, Germany; 6 Department of Medicine-DIMED, University-Hospital of Padova, Padua, Italy; 7 Department of Medical Sciences, Uppsala University, Uppsala, Sweden; 8 Health in Code SL, A Coruña, Spain; 9 Scientific Department. Dilemma Solutions SL, A Coruña, Spain; 10 Estonian Genome Centre, Institute of Genomics, University of Tartu, Riia 23B, Tartu, Estonia; 11 Emergency Cardiology Department, Institute of Cardiology, Clinical and Regenerative Medicine, Kiev, Ukraine; 12 Clinical Bioinformatics, Saarland University, Saarbrücken, Germany

**Keywords:** MicroRNAs, Acute coronary syndrome, Coronary artery disease, Dilated cardiomyopathy, Ischemic cardiomyopathy, Computational biology

## Abstract

**Background:**

Circulating miRNAs have emerged as promising biomarker candidates due to their stability and their role in regulating key pathological pathways in cardiovascular disease (CVD). Yet, large-scale, multicentre studies examining their diagnostic and prognostic potential are scarce. This study evaluates the potential of miRNA expression profiles to inform disease classification and risk stratification across major CVD phenotypes, including acute coronary syndrome (ACS), chronic coronary artery disease (CAD), dilated cardiomyopathy (DCM), and ischemic cardiomyopathy (ICM), in a large, multicentre European cohort.

**Methods:**

We assessed genome-wide miRNA expression profiles in a total of 1209 cardiovascular patients and 848 controls in a uniform, standardized fashion, which renders this study one of the largest prospective miRNA studies. To focus on only the most biologically plausible miRNAs for clinical translation, we mined all original studies of miRNA candidates in CVD and performed differential miRNA expression and enrichment analysis. We then trained disease-specific binary classification models to evaluate the diagnostic potential of miRNA signatures. Finally, we evaluated prognosis and disease severity based on distinct miRNA levels.

**Results:**

Six hundred thirty four original abstracts were identified, detailing 166 ACS, 181 CAD, 56 DCM, and 182 ICM miRNAs. Without further optimization, the signatures of a priori miRNAs already yielded very good diagnostic performance with ROC AUC of 0.83–0.95. There was an improvement when considering additional miRNAs in a discovery setting. Interestingly, in ACS, CAD, and DCM, we observed a significantly worse prognosis in probands with higher miRNA-derived disease probabilities, indicating an association with prognosis.

**Conclusions:**

The European BestAgeing miRNA study reveals emerging associations of several miRNA signatures with cardiovascular disease discrimination and prognostication, providing a foundation for future external validation and potential clinical translation of this class of markers.

**Supplementary Information:**

The online version contains supplementary material available at 10.1186/s12916-025-04502-3.

## Background

Cardiovascular diseases (CVDs) remain one of the most significant global health challenges, accounting for a substantial proportion of morbidity and mortality worldwide [1, 2]. Within the broad spectrum of CVD, coronary artery disease (CAD), ischemic cardiomyopathy (ICM), dilated cardiomyopathy (DCM) and acute coronary syndrome (ACS) represent complex clinical manifestations that are multifactorial in nature.

MicroRNAs (miRNAs) primarily regulate gene expression at the post-transcriptional level [3]. Recent evidence suggests that miRNAs are involved in several biological processes and cellular pathways that are closely associated with the development and progression of CVD [4]. Their ability to modulate cardiac remodeling, inflammation, apoptosis, and fibrosis, among others, makes them attractive candidates for both diagnostic biomarkers and therapeutic intervention [5–8]. miRNAs can be found in different blood components, for instance in circulating cells, as well as in plasma and serum, where they are either bound to protein complexes or contained in microvesicles or lipoproteins that protect them from being degraded by RNAses [7, 9]. As such, miRNAs have been studied in a wide range of CVD, including ACS, CAD, ICM, and DCM and although their potential of novel blood-borne biomarkers has been recognized, the translation into clinical practice has been minimal [10–22]. This gap is partly due to the limited scale of clinical studies with relatively small number of cases and differences in study cohorts, restricting the robust validation of identified miRNA signatures. Furthermore, variations in sampling procedures, pre-analytic sample handling, as well as poorly standardized and united protocols of RNA extraction and purification are having a significant impact on the results of such studies [23, 24].

The European BestAgeing Study Group was established to translate novel biomarker candidates and omics-entities towards standardized preclinical assessment. In this work, we systematically explore and uniformly evaluate the diagnostic potential of miRNA signatures for a range of cardiovascular diseases, including ACS, CAD, DCM, and ICM, in a large multicentric, prospective cohort with a special focus on validating miRNAs that have been previously identified in the literature or existing patents, hence increasing the likelihood of successful further implementation. Our findings provide valuable information for precision diagnostics and for understanding the involvement of miRNAs in CVD.

## Methods

The study was conducted in accordance with the principles of the Declaration of Helsinki. Written informed consent was obtained from all study participants, and the Ethics Committee and Institutional Review Boards of all participating centers approved the inclusion and study of clinical data and biomaterials. The study and the analysis of the data followed the Standards for Reporting of Diagnostic Accuracy (STARD) reporting guideline.

### Patient recruitment

This study was designed as a prospective multicenter observational study, focusing on four distinct CVDs, including ACS, CAD, DCM, and ICM. In addition, a control group without signs or symptoms of these disease phenotypes was recruited. Two thousand fifty seven participants were enrolled from 11 major centers across Europe from 2013 to 2017. Five years of follow-up data as well as heart catheterization data were available for the lead center (UKHD).

CAD was diagnosed based on coronary angiography, showing at least one significant stenosis of over 50% in at least one coronary artery. Patients with ACS were included in the study if they presented at least two of the following criteria: typical chest pain, elevated high-sensitivity Troponin levels (above the 99th percentile), or characteristic alterations on electrocardiogram. Inclusion criteria for DCM cases were the presence of reduced left ventricular systolic function (LVEF < 45% as measured by echocardiography or magnetic resonance imaging) in the absence of relevant CAD as determined by coronary angiography and a left ventricle end-diastolic diameter > 117% of predicted value according to age and body surface area in echocardiography (Henry equation for predicted LVEDD = 45.3 × 1/3 BSA – 0.03 × Age – 7.2). ICM patients were included in the presence of reduced left ventricular systolic function in conjunction with relevant coronary artery disease (LVEF fraction < 45% as measured by echocardiography or magnetic resonance imaging). A list of phenotype-specific inclusion and exclusion criteria can be found in the Additional file 1: *Supplementary Methods*.

### miRNA extraction and microarray analysis

Five milliliters of peripheral blood was collected and frozen according to a standardized protocol in two PAXgene Blood RNA tubes (BD Biosciences, USA) for each participant. At time point of sample processing the tubes were thawed overnight at room temperature to ensure complete lysis of blood cells. RNA was extracted and purified using the PAXgene Blood miRNA Kit according to the manufacturer’s instructions (Qiagen GmbH, Hilden, Germany). Quantification of RNA eluates was performed with NanoDrop spectrophotometer (Thermo Fisher Scientific, Waltham, USA). Agilent Bioanalyzer and the Nano RNA Kit were used to assess RNA quality and integrity (Agilent Technologies, Santa Clara, USA). The mean (SD) RNA integrity value was 8.2 (1.0). The miRNA expression profile of mature human miRNAs was assessed as described previously using human miRNA microarrays and the miRNA Complete Labeling and Hyb Kit (Agilent Technologies, Santa Clara, USA) [25, 26]. Labeled RNA was hybridized to the array slides for 20 h at 55 °C with 20-rpm rotation. Arrays were washed twice, air dried, and scanned in the microarray scanner with a 3-μm resolution in double-path mode. Raw data were extracted using Agilent Feature Extraction software (Agilent Technologies, Santa Clara, USA). All samples were measured at *Hummingbird Diagnostics* in Heidelberg. We started our analysis on 2549 miRNAs from the miRBase v21. We filtered these miRNAs to improve the signal-to-noise ratio so that our final miRNA set comprised 440, 436, 436 and 453 likely stable miRNAs for the ACS/control, the CAD/control, the DCM/control and the ICM/control comparisons, respectively. See Additional file 1: *Supplementary Methods* for further details.

### Text mining and literature miRNAs

A comprehensive literature search was conducted to identify studies focusing on the role of miRNAs in the different cardiovascular diseases included and their associated diagnostic biomarkers. The primary goal of this literature search was to validate whether these a priori defined miRNAs in given distinct cardiovascular phenotypes are differentially expressed in our dataset, based on the results of our differential miRNA expression analysis. The search was performed in the PubMed database, employing a strategy that utilized both MeSH terms and keywords in titles and abstracts (specific search strategy for each disease is mentioned in the Additional file 1: *Supplementary Methods*). For the comprehensive investigation of miRNAs found, the R package *miRetrieve* was utilized [27]. This package facilitated the extraction and analysis of miRNA-related data from the abstracts and allowed the calculation of a biomarker score based on the occurrence of defined diagnostic and biomarker-related keywords. This score, combined with the frequency of miRNA mentions, allowed a refined assessment of each miRNA’s relevance to CVD. The top 50 miRNAs were then investigated with special focus in this study, reflecting their potential prominence as diagnostic targets in the respective cardiovascular conditions.

### Diagnostic machine learning analysis

Our first analysis included a feature selection step (*m* = 20) using a random forest feature importance score in the preprocessing of the training data to investigate miRNAs with the most information in our dataset (*DISCOVERY-Model*). In a second analysis, we only considered the a priori selected miRNA features from the literature search (A-priori miRNA Model; *aPRIORI-Model*). Latin Hypercube sampling was used to create a hyperparameter grid for the benchmarked models penalized logistic regression, XGBoost and Random Forest. These models were chosen for their complementary strengths in handling high-dimensional, multicollinear, and potentially non-linearly separable tabular data such as miRNA expression profiles [28]. Models were evaluated using the receiver operating characteristic area under the curve (ROC-AUC) metric during hyperparameter tuning only on the training set (75%, 10 times repeated fivefold cross validation). We computed ROC-AUC, accuracy, sensitivity and specificity, positive and negative predictive values, and the harmonic mean of precision and recall (F1 score) on the blinded test data (25%). Calibration plots were constructed to visualize the agreement between predicted probabilities and observed outcomes. We then used a model agnostic approach to compute global variable importance by permutating features. If shuffling a column causes a large degradation in model performance, it is important and vice versa [29, 30]. See Additional file 1: *Supplementary Methods* for further details. In a next step, the trained diagnostic Machine Learning (ML) models were used to calculate probabilities for the model-specific disease for each patient within this subgroup. We then evaluated the influence of the diagnostic model probabilities (stratified by both median and tertiles of predicted disease probability) on all-cause mortality among patients in the Heidelberg subcohort (*n* = 727, lead center) using Kaplan–Meier curves. For comparison of heart-failure related phenotypes with NT-proBNP, we focused on cases where NT-proBNP interpretation is less definitive. First, a grey-zone ROC analysis was conducted in DCM patients with NT-proBNP values below the median NT-proBNP concentration (< 790 pg/mL). Logistic regression models compared NT-proBNP, individual top differentially expressed miRNAs, phenotype-specific miRNA signatures, and combined models. Second, a reclassification analysis was performed in patients within the clinical NT-proBNP grey zone (125–450 pg/mL) to assess whether adding individual miRNAs or the miRNA signature improved classification into rule-in (RI) or rule-out (RO) categories, quantified by net reclassification improvement (NRI). Full methodological details are provided in Additional file 1.

### Statistical analysis

All results for continuous variables are expressed as mean with corresponding standard deviation and median with interquartile range for skewed distributions. For group-wise comparisons, Mann–Whitney test (2 groups), ANOVA, Kruskal–Wallis test (*n* groups), or Student’s *t* test (2 groups) were used as appropriate. For categorical variables, Fisher’s exact test or the *χ*^2^ test were used. In addition, the log-rank test was used to compare all-cause mortality between groups. Unless stated otherwise, the significance threshold was 0.05 and tests were 2-sided. It should be noted that this study represents an exploratory validation of literature-derived miRNA candidates rather than a definitive clinical validation study. All performance metrics should be interpreted within this context. For replicated on array measurements, the median intensity was computed resulting in a matrix of 2058 columns (samples) and 2549 rows (miRNAs). The miRNA expression intensities were background subtracted and the median intensity of replicates was computed. The resulting matrix was filtered for expressed miRNAs and normalized using quantile normalization (Additional file 1: *Supplementary Methods*). The impact of batch-effect correction was evaluated visually using PCA plots before and after correction (Additional file 2: Fig. S1). After correction, outlier clustering is markedly reduced, with samples distributing more evenly across principal components, demonstrating that the correction procedure effectively removed known and SVA-estimated batch effects. Analyses were then performed pairwise on 440, 436, 436 and 453 expressed miRNAs in ACS, CAD, DCM and ICM versus controls respectively. Additionally, PCA was performed to visualize the presence of obvious batch effects. Positive predictive values were calculated for all disease/control comparisons according to Bayes’ theorem. If not specified otherwise, prevalence was defined as the fraction of individuals in the respective scenario. As the hypothesis test, an unpaired 2-tailed *t-*test was applied. In addition, we report raw *p*-values from a logistic regression model with disease status as the outcome variable and each considered miRNA as the independent variable adjusted for age, sex with and without the first 3 principal components to detect differential microRNA expression analysis. To further increase the robustness of our findings, in addition to adjusting for covariates, we also matched for age and sex, as the baseline characteristics showed a clear deviation from the control group. This was done using the *MatchIt* package in R, using the “nearest” method. *P*-values were subjected correction for multiple testing using the Bonferroni-Holm approach. For each miRNA, we computed the area under the receiver-operating characteristics curve (AUC value). Subsequently, we conducted an enrichment analysis using the MiEAA (miRNA Enrichment Analysis and Annotation) tool to assess the biological relevance of miRNAs that were significantly differentially expressed in pairwise comparisons with control samples [31]. The input miRNAs were derived from our differential expression analysis and subsequently submitted to MiEAA for over-representation analysis. All analyses were performed using R Statistical Software (v4.2.0; R Core Team 2022).

## Results

### Study design and patient characteristics

**Fig. 1:**
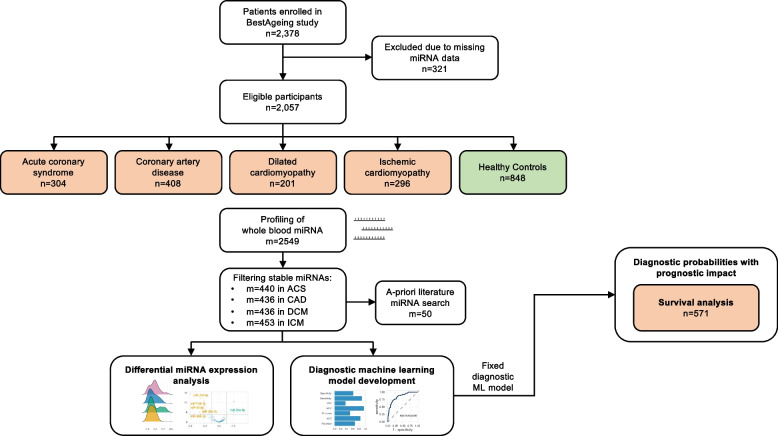
Study design

A total of 2378 participants were included in the study and miRNAs were assessed by standardized assays in as much as 2057 patients (Fig. 1). This included patients across 11 major European cardiovascular centers with ACS (*n* = 304), CAD (*n* = 408), DCM (*n* = 201), and ICM (*n* = 296) and a well-defined clinical control group (*n* = 848). The mean age of the total study population was 62.5 years (± 14.0) and 1365 participants were male (66.4%). Table 1 provides the demographic characteristics of the study population, stratified by disease status. The recruitment of patients according to their respective diagnosis in each center is detailed in Additional file 3: *Table S1*.

**Table 1:** Demographic characteristics of the participants. The table provides an overview of all patients enrolled in the study stratified by adjudicated disease. ACS, acute coronary syndrome, BMI, body mass index, CAD, coronary artery disease, DCM, dilated cardiomyopathy, ICM, ischemic cardiomyopathy, LVEF, left ventricular ejection fraction, SD, standard deviation

Characteristic	ACS (*n* = 304)	CAD (*n* = 408)	DCM (*n* = 201)	ICM (*n* = 296)	Control (*n* = 848)
Sex = Male (%)	214 (70.6)	313 (76.7)	151 (75.1)	227 (76.7)	460 (54.2)
Age (mean (SD)), y	65.88 (12.44)	69.07 (10.30)	57.45 (14.73)	65.10 (12.27)	58.65 (14.58)
BMI (mean (SD)), kg/m^2^	28.51 (14.10)	28.11 (4.81)	28.08 (6.04)	29.32 (16.81)	26.65 (8.96)
Smoking Status, No. (%)					
Ex	97 (35.5)	117 (33.1)	65 (36.1)	72 (35.3)	
No	106 (38.8)	179 (50.7)	81 (45.0)	102 (50.0)	230 (73.7)
Yes	70 (25.6)	57 (16.1)	34 (18.9)	30 (14.7)	82 (26.3)
Diabetes = Present (%)	79 (26.4)	130 (33.4)	38 (20.7)	95 (34.3)	17 (2.0)
Hypertension = Yes (%)	213 (71.7)	332 (84.1)	107 (58.2)	204 (73.6)	215 (25.3)
Dyslipidaemia = Yes (%)	114 (53.0)	273 (70.7)	70 (38.0)	107 (53.8)	102 (28.0)

Left ventricular ejection fraction < 45% per inclusion criteria for ICM and normal for Control group

### Literature-derived miRNA signatures in cardiac disease

The application of text mining and literature-based analysis on the role of miRNAs in CVDs was comprehensive, encompassing a total of 634 studies that met the search strategy. After processing abstracts, a total of 585 unique miRNAs were found. Most unique miRNAs were described for ICM (*m* = 182) followed by CAD (*m* = 181), ACS (*m* = 166) and DCM (*m* = 56). These miRNAs partially overlapped between disease entities. A detailed breakdown of the total number retrieved and processed abstracts is given in Additional file 3: *Table S2* and the intersection of literature miRNAs is given as a resource in Additional file 3: *Table S3*. Due to the overlap of miRNAs related to the diseases of interest, we found 112 unique miRNAs. The 20 most frequently mentioned miRNAs are shown in a bar chart in Additional file 2: *Figure* *S2*. The top 50 miRNAs per disease were then identified and ranked according to the weighted biomarker score that was calculated using the *miRetrieve* algorithm based on keywords. In Additional file 3: *Table* *S4*, these a priori selected miRNAs are arranged according to their univariate AUCs, and their corresponding PubMed Identifier (PMID) are given. These values collectively provide insight into the potential significance and diagnostic relevance of each miRNA in the context of the particular cardiovascular condition being studied.

### Cardiovascular disease leads changes in miRNA expression patterns

**Fig. 2:**
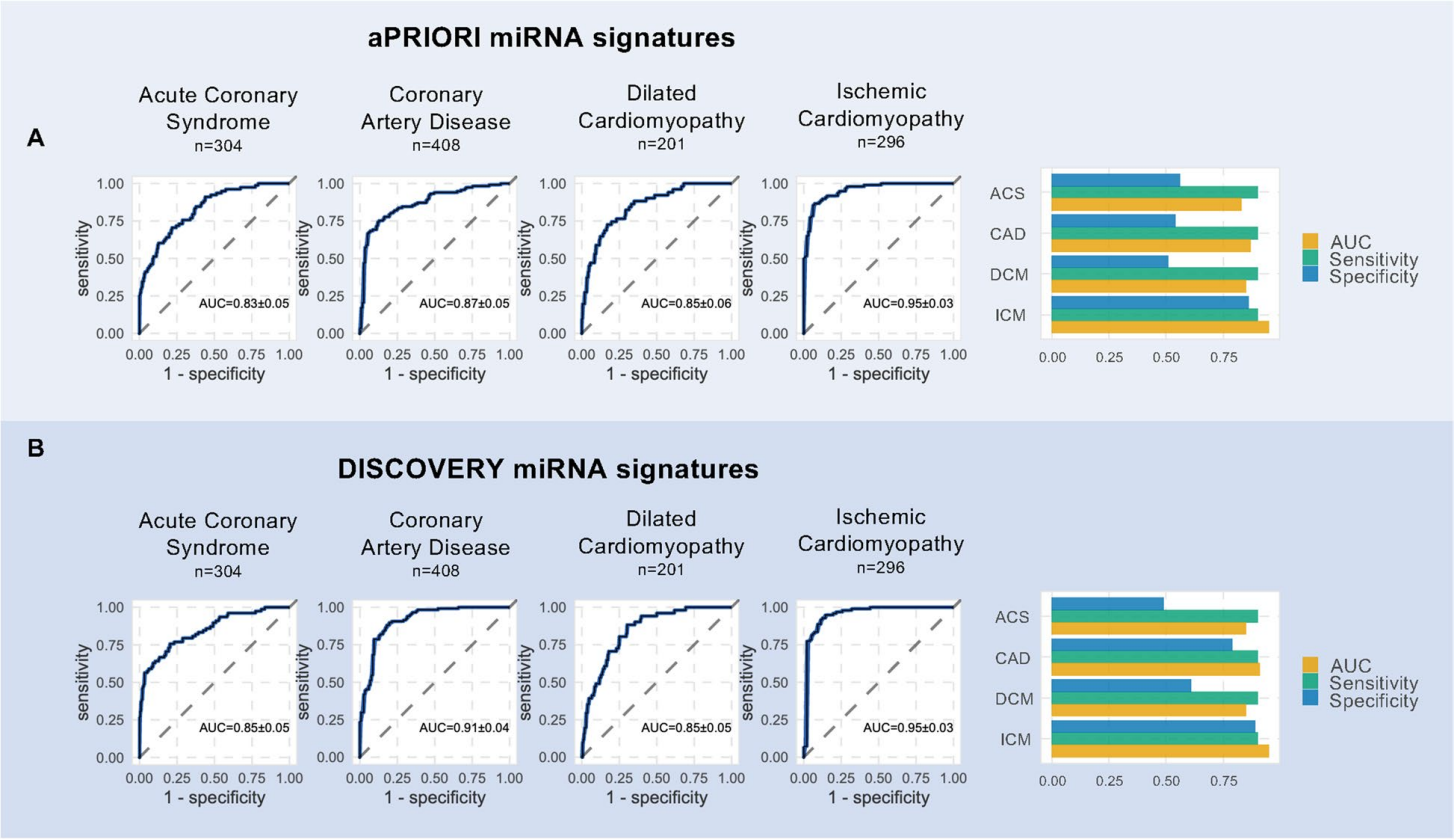
Comparative performance of machine learning models on miRNA signatures in cardiovascular disease. Panels show ROC curves (left) and bar plots of key performance metrics (right) for each disease group (ACS, CAD, DCM, ICM from left to right). **A** aPRIORI miRNA signatures with models trained using only pre-specified miRNAs identified from literature mining. **B** DISCOVERY miRNA signatures: models trained using miRNAs selected from the full dataset by feature selection. ROC curves display mean classification performance, with AUC values given as mean ± SD from 10-times repeated fivefold cross-validation on the training set, evaluated on the blinded 25% holdout test set. Bar plots summarize AUC (orange), sensitivity (green), and specificity (blue) for each disease group. ACS, acute coronary syndrome, AUROC, area under the receiver operating curve, CAD, coronary artery disease, DCM, dilated cardiomyopathy, ICM, ischemic cardiomyopathy, ROC, Receiver operating characteristic

**Fig. 3:**
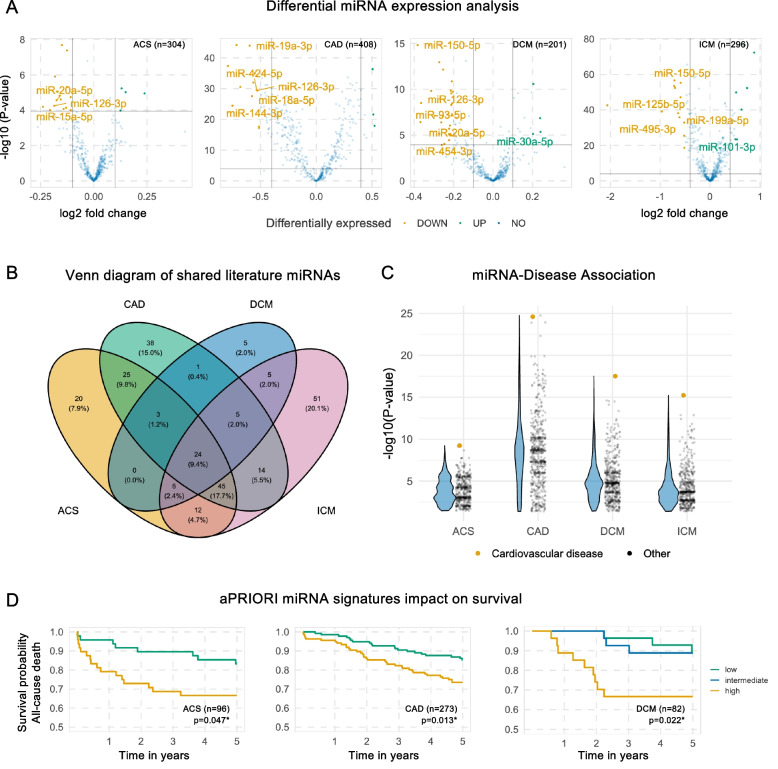
Expression and overlap of miRNA biomarkers in different CVD. **A** Differential miRNA expression in cardiovascular disease. Volcano plots illustrating differentially expressed miRNAs after Bonferroni-Holm adjustment. Points above the threshold line indicate miRNAs with statistically significant changes in expression, with those of particular interest (found in the literature) highlighted. **B** Venn diagram of shared literature miRNAs in distinct cardiovascular phenotypes.** C** Top differentially expressed miRNAs show strong enrichment in cardiovascular disease. Using MiEAA, we were able to show that miRNAs that were significantly differentially expressed in pairwise comparisons with control samples in our analysis were most significantly associated with cardiovascular disease in a subsequent enrichment analysis in the MNDR v3.0. Black points indicate enrichments associated with 598 diseases other than “Cardiovascular disease” (“Other”). **D** aPRIORI diagnostic disease model and impact on patient survival. Kaplan–Meier survival curves stratified by median probability and tertiles for ACS, CAD, and DCM derived from the aPRIORI models, highlighting the prognostic significance and more severe phenotype of the high-risk group. ACS, acute coronary syndrome, CAD, coronary artery disease, DCM, dilated cardiomyopathy; MiEAA, miRNA Enrichment Analysis and Annotation; MNDR, Mammal ncRNA-Disease Repository

All samples were measured in a blinded and randomized fashion. First, we present the analysis of samples from patients suffering from ACS (*n* = 304) and compared them to controls (*n* = 848). From the 75% training set, a signature of 20 miRNAs was identified by feature selection (DISCOVERY-model). When the finalized DISCOVERY-model was applied on the blinded test set, the signature distinguished between ACS and controls with an AUC of 0.85 (95% CI, 0.80*–*0.90), a sensitivity of 92.9% (95% CI, 86.4–96.9%) and a specificity of 39.3% (95% CI, 32.1–46.9%). Next, the aPRIORI-model was trained only on pre-specified literature miRNAs and the performance statistics on the blinded test set resulted in an AUC of 0.83 (95% CI, 0.78–0.88), a sensitivity of 93.7% (95% CI, 88.0–97.2%) and a specificity of 42.9% (95% CI, 35.2–50.9%, Fig. 2 and Table 2). The global variable importance calculated by permutation of the aPRIORI features highlighted miR-21-5p, miR-363-3p and miR-15a-5p, although there were only minor differences in importance in the aPRIORI signature. In total, a good model calibration was observed on the test set (Additional file 2: *Figures S3-4*). Considering the high biomarker scores in Additional file 3: *Table* *S4*, the frequently described miRNA miR-21-5p shows a significant but weak univariate discrimination with an AUROC of 0.56 (95% CI, 0.51–0.62), whereas the well described miRNA 126-3p allows a slightly better discrimination with an AUROC of 0.61 (95% CI, 0.56–0.66). Overall, weak univariate statistics were obtained with the highest AUROC values of 0.63 (95% CI, 0.58–0.68) for miR-144-5p and 0.63 (95% CI, 0.57–0.68) for miR-126-5p, both of which were also selected in the ACS DISCOVERY-model. Raw and adjusted *P*-values and AUROC values for all univariate miRNAs are given in Additional file 3:*Tables S5-8.* The volcano plots for differential miRNA expression are shown in Fig. 3A and highlight miRNAs that not only show significant differential expression but have also been identified in literature. The Venn diagram in Fig. 3B shows a representation of shared literature miRNAs and their association and overlap with specific cardiovascular diseases (detailed information is provided in Additional file 3: *Table* *S3*).

**Table 2:** Diagnostic performance of ML models. The table presents the performance metrics of the ML model on the blinded test set and shows the estimate (CI). Statistics are given for the full model with feature selection as well as for the a priori selected miRNAs from the literature and presented as point estimates with 95% confidence intervals in parentheses. Abbreviations: AUROC, area under the receiver operating characteristics curve; NPV, negative predictive value; PPV, positive predictive value

Statistic	aPRIORI Model	DISCOVERY-Model
**Acute Coronary Syndrome**		
Sensitivity	0.937 (0.880; 0.972)	0.929 (0.864; 0.969)
Specificity	0.429 (0.352; 0.509)	0.393 (0.321; 0.469)
PPV	0.561 (0.492; 0.629)	0.491 (0.421; 0.560)
NPV	0.897 (0.808; 0.955)	0.897 (0.808; 0.955)
AUROC	0.829 (0.777; 0.881)	0.848 (0.795; 0.900)
**Coronary Artery Disease**		
Sensitivity	0.840 (0.737; 0.914)	0.885 (0.807; 0.939)
Specificity	0.660 (0.581; 0.734)	0.808 (0.729; 0.872)
PPV	0.538 (0.444; 0.631)	0.786 (0.701; 0.857)
NPV	0.897 (0.828; 0.946)	0.897 (0.828; 0.946)
AUROC	0.868 (0.821; 0.915)	0.912 (0.875; 0.948)
**Dilated Cardiomyopathy**		
Sensitivity	0.956 (0.901; 0.986)	0.963 (0.915; 0.988)
Specificity	0.309 (0.236; 0.390)	0.357 (0.274; 0.446)
PPV	0.514 (0.445; 0.583)	0.608 (0.539; 0.675)
NPV	0.902 (0.786; 0.967)	0.902 (0.786; 0.967)
AUROC	0.848 (0.791; 0.905)	0.845 (0.792; 0.898)
**Ischemic Cardiomyopathy**		
Sensitivity	0.892 (0.811; 0.947)	0.896 (0.817; 0.949)
Specificity	0.861 (0.778; 0.922)	0.888 (0.808; 0.943)
PPV	0.856 (0.770; 0.919)	0.887 (0.806; 0.942)
NPV	0.897 (0.819; 0.949)	0.897 (0.819; 0.949)
AUROC	0.953 (0.926; 0.979)	0.955 (0.924; 0.986)

For CAD, involving 408 patients, the derived 20-miRNA DISCOVERY-model distinguished patients from controls with an AUC of 0.91 (95% CI, 0.87–0.95), combining high sensitivity (88.5%; 95% CI, 80.7–93.9%) and specificity (80.8%; 95% CI, 72.9–87.2%). The aPRIORI model, again based on literature miRNAs, showed similarly good performance (AUC 0.87; 95% CI, 0.82–0.92) with only slightly reduced sensitivity and specificity. miR-144-3p and miR-19a-3p were identified as most significant markers, with moderate univariate and individual AUROC values indicating fair discriminatory power. The univariate discriminatory power of the top features was good with AUROC values of 0.76 (95% CI, 0.73–0.79) for miR-19a-3p and 0.68 (95% CI, 0.65–0.72) for miR-144-3p. Among the most commonly reported miRNAs, moderate AUROC values of 0.69 (95% CI, 0.65–0.72) were found for miR-126-3p and 0.68 (95% CI, 0.64–0.71) for miR-21-5p.

In DCM (*n* = 201), the DISCOVERY-model signature demonstrated an AUC of 0.85 (95% CI, 0.79–0.90), along with a sensitivity of 96.3% (95% CI, 91.5–98.8%) and a specificity of 35.7% (95% CI, 27.4–44.6%) on the blinded test set. The a priori model performance was comparable (AUC 0.88; 95% CI, 0.84–0.93), with similar sensitivity and specificity. The aPRIORI global variable importance underscored the importance of miR-150-5p and miR-30a-5p. The AUROC for miR-150-5p was 0.66 (95% CI, 0.61–0.71), indicating a modest ability to distinguish DCM cases from controls. miR-30a-5p showed a similar AUROC of 0.62 (95% CI, 0.56–0.67). For mir-126-3p, we observed the highest discrimination of DCM with an AUROC of 0.67 (95% CI, 0.62–0.72), which was supported by the highest biomarker score as shown in Additional file 3: Table S4. Identified DCM miRNAs in the literature had the highest overlap with miRNAs identified for ICM, which has been more extensively studied in terms of miRNA candidates (Fig. 3B, Additional file 3: Tables S2-3). ICM analysis, involving 296 samples, revealed the highest discrimination capability with an AUC of 0.96 (95% CI, 0.92–0.99) using the DISCOVERY model, and an AUC of 0.95 (95% CI, 0.93–0.98) with the aPRIORI model, both showing high sensitivity and specificity. Among the most consistently reported miRNAs, fair AUROC values of 0.77 (95% CI, 0.74–0.81) were found for miR-150-5p, 0.74 (95% CI, 0.70–0.77) for miR-106b-5p, 0.73 (95% CI, 0.69–0.76) for miR-22-3p and 0.71 (95% CI, 0.68–0.75) for miR-199a-5p (Additional file 3: *Table S4*).

Our downstream enrichment analysis showed a strong enrichment of the top differentially expressed miRNAs in cardiovascular disease for all phenotypes investigated and thus emphasizes the importance of differentially expressed miRNAs in our dataset as cardiovascular markers (Fig. 3C). The overall performance of both aPRIORI and DISCOVERY models across diseases is summarized in Fig. 2, with detailed performance metrics in Table 2. Additional file 2: Figures S3-4 provides additional insights into model calibration and feature importance. To complement the literature-based intersections (Additional file 3: Table S3), we summarized data-driven overlaps of significantly dysregulated miRNAs from our pairwise disease–control analyses (Additional file 3: Table S9). Intersections align with clinical spectra: CAD/ICM (“Ischemic spectrum”, largest pairwise overlap, *n* = 155 miRNAs) reflects shared expression profiles from chronic CAD to heart-failure stages; CAD/DCM/ICM (“Pan-cardiomyopathy”, *n* = 32 miRNAs) is consistent with remodeling processes observed across cardiomyopathy phenotypes, and DCM/ICM (“Heart failure phenotypes”, *n* = 11 miRNAs) captures signals related to systolic ventricular dysfunction. These patterns are visually corroborated by the annotated heatmap (Additional file 2: Fig. S5) which shows the expression of the top 25 dysregulated miRNAs per disease after hierarchical clustering. The heatmap, based on *z*-scores of miRNAs, highlights upregulated miRNAs in the upper right for both ICM and CAD, whereas downregulated miRNAs can be detected in the lower left for a cluster of DCM and CAD.

### Diagnostic signatures are associated with worse prognosis and more severe phenotype in ACS, CAD and DCM

To assess the potential prognostic value of selected miRNA signatures, we analyzed all-cause mortality in patients recruited and followed up at the University Hospital of Heidelberg. Of the 727 patients, only 156 were lost to follow-up (follow-up rate 79%). As shown in Additional file 2: Fig. S6A, the Kaplan–Meier survival analysis revealed different survival outcomes between disease groups over a 5-year follow-up period. Patients diagnosed with ACS had the lowest survival rate (0.75, 95% CI 0.67–0.83), whereas survival rates were more similar for CAD (0.80, 95% CI 0.75–0.85) and DCM (0.82, 95% CI 0.74–0.90), and the control group had the highest survival rate (0.92, 95% CI 0.87–0.97). For model introspection and to assess the impact of the predicted disease probability on patient all-cause mortality, we analyzed ACS patients stratified by median and tertiles of miRNA-predicted disease probability and performed a Kaplan–Meier survival analysis (*n* = 96). We observed 33.3% all-cause mortality in the high-risk group, compared with 16.7% in the low-risk group (log-rank *p*-value = 0.047) (Fig. 3D, Additional file 2: Fig S6B). Interestingly, we also observed a significant difference in hs-TroponinT levels, with median values of 108.50 pg/mL (IQR: 41.75–211.00) in the low-risk group and 182.00 pg/mL (IQR 77.00–649.00) in the high-risk group (*p* = 0.025) (Table 3). Detailed patient characteristics stratified by predicted probabilities are provided in Additional file 3: Tables S10-13.

**Table 3:** ACS risk groups stratified by aPRIORI ACS model probabilities. This table provides the patient demographics and laboratory values stratified by median probability values for ACS derived from the aPRIORI model, analyzing only patients from the University Hospital of Heidelberg. ACS, acute coronary syndrome; ac, all-cause; INR, international normalized ratio

Variable	Low (*n* = 48)	High (*n* = 48)	*P*-value
AC-Mortality = Died (%)	8 (16.7)	16 (33.3)	0.047
Age, years (mean (SD))	69.10 (12.91)	67.85 (12.86)	0.636
Gender = Male (%)	33 (68.8)	29 (60.4)	0.522
Weight, kg (mean (SD))	82.74 (15.11)	78.62 (18.62)	0.307
Body Mass Index, kg/m^2^ (mean (SD))	28.10 (4.73)	26.82 (5.45)	0.295
Smoking = Yes (%)	28 (58.3)	31 (64.6)	0.675
Diabetes = Yes (%)	15 (31.2)	19 (39.6)	0.522
Hypertension = Yes (%)	40 (83.3)	42 (87.5)	0.772
Family History = Yes (%)	18 (37.5)	10 (20.8)	0.116
Creatinine, mg/dl (median [IQR])	0.88 [0.79, 1.00]	0.87 [0.74, 1.11]	0.866
hs-TroponinT, ng/l (median [IQR])	108.50 [41.75, 211.00]	182.00 [77.00, 649.00]	0.025
nt-proBNP, pg/ml (median [IQR])	568.00 [227.00, 2,384.50]	172.00 [86.75, 7,314.00]	0.529
Hemoglobin, g/dl (median [IQR])	13.70 [12.53, 14.40]	13.80 [11.10, 15.10]	0.531
INR (median [IQR])	1.02 [0.99, 1.09]	1.03 [1.01, 1.08]	0.613
Bilirubin, mg/dl (median [IQR])	0.60 [0.60, 0.80]	0.65 [0.50, 0.83]	0.620
Leukocytes,/nl (median [IQR])	9.19 [7.56, 10.80]	9.16 [7.04, 12.29]	0.777
Cholesterol, mg/dl (median [IQR])	204.50 [158.00, 240.25]	180.00 [156.50, 211.50]	0.284
**aPRIORI ACS Probability (mean (SD))**	**0.50 (0.16)**	**0.86 (0.08)**	** < 0.001**

We also analyzed DCM patients stratified by median and tertiles of predicted disease probability and performed a Kaplan–Meier survival analysis. We observed an all-cause mortality rate of 10.7% in the low-risk group, 11.1% in the intermediate-risk group and 33.3% in the high-risk group (log-rank *p*-value = 0.022, Fig. 3D), indicating that patients with the highest likelihood of disease had significantly the highest mortality rate, even though the models were trained solely on miRNA features alone, without prognostic information. A trend was observed when patients were stratified by median threshold and mortality was observed: 24.4% all-cause mortality in the high-risk group, compared with 12.2% in the low-risk group (log-rank *p*-value = 0.116) (Fig. 3D). A more severe DCM phenotype was also observed in the high-risk group, with a significantly lower left ventricular ejection fraction (25.4 ± 11.4% vs 33.5 ± 14.2, *p*-value = 0.027) and a trend towards higher nt-proBNP levels (*p*-value = 0.195) (Table 4).

**Table 4:** DCM risk groups stratified by aPRIORI DCM model probabilities. This table provides the patient demographics and laboratory values stratified by median probability values for DCM derived from the aPRIORI model, analyzing only patients from the University Hospital of Heidelberg. DCM, dilated cardiomyopathy

Variable	Low (*n* = 41)	High (*n* = 41)	*P*-value
AC-Mortality = Died (%)	5 (12.2)	10 (24.4)	0.116
Age, years (mean (SD))	63.83 (13.71)	56.37 (16.90)	0.031
Gender = Male (%)	29 (70.7)	34 (82.9)	0.295
Weight, kg (mean (SD))	87.60 (20.05)	88.38 (27.71)	0.887
Body Mass Index, kg/m^2^ (mean (SD))	27.82 (4.97)	28.30 (7.88)	0.750
Smoking = Yes (%)	20 (48.8)	19 (46.3)	1.000
Diabetes = Yes (%)	9 (22.0)	14 (34.1)	0.325
Hypertension = Yes (%)	24 (58.5)	27 (65.9)	0.649
Family History = Yes (%)	10 (24.4)	12 (29.3)	0.803
Creatinine, mg/dl (median [IQR])	0.98 [0.90, 1.14]	0.99 [0.83, 1.23]	0.872
hs-TroponinT, ng/l (median [IQR])	15.50 [12.25, 25.50]	12.00 [8.50, 15.50]	0.177
nt-proBNP, pg/ml (median [IQR])	554.00 [199.50, 1,803.00]	886.50 [476.75, 3,233.50]	0.195
Hemoglobin, g/dl (median [IQR])	14.30 [12.97, 15.43]	13.70 [12.50, 15.30]	0.494
INR (median [IQR])	1.03 [0.99, 1.08]	1.02 [0.98, 1.19]	0.935
Bilirubin, mg/dl (median [IQR])	0.60 [0.45, 0.80]	0.85 [0.62, 1.23]	0.156
Leukocytes,/nl (median [IQR])	7.20 [6.09, 9.06]	7.38 [6.24, 9.56]	0.691
Cholesterol, mg/dl (median [IQR])	170.00 [159.50, 202.00]	163.00 [153.75, 182.25]	0.498
Ejection Fraction, % (mean (SD))	33.52 (14.16)	25.39 (11.37)	0.027
**aPRIORI DCM Probability (mean (SD))**	**0.20 (0.08)**	**0.60 (0.18)**	** < 0.001**

**Fig. 4:**
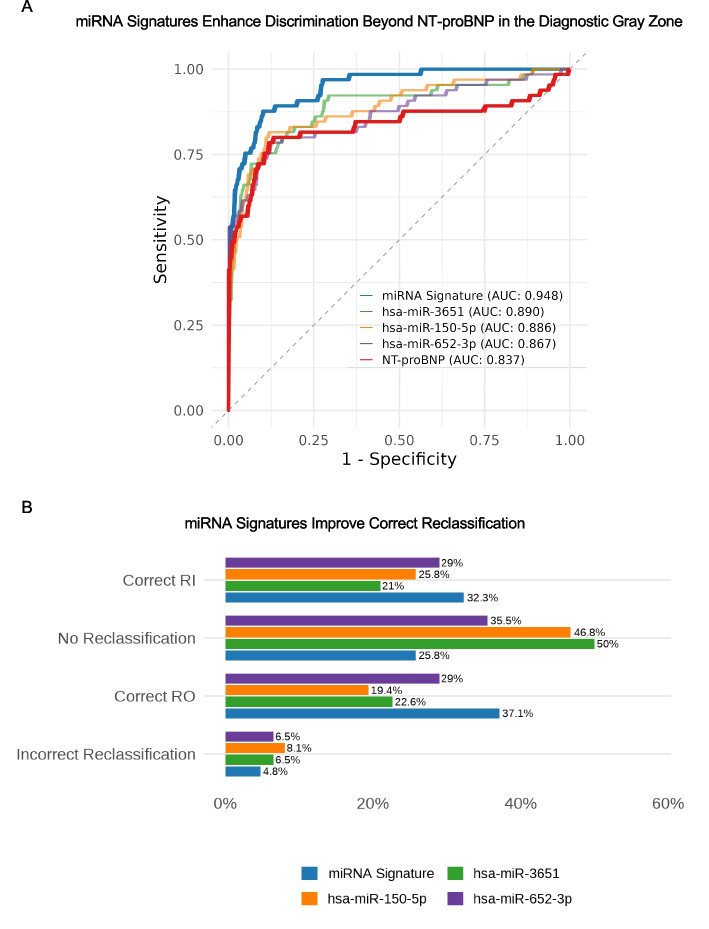
Incremental diagnostic gains from adding miRNAs to NT-proBNP. **A** Receiver operating characteristic (ROC) curves for NT-proBNP, top-performing individual miRNAs (hsa-miR-3651, hsa-miR-150-5p, hsa-miR-652-3p), and the composite miRNA signature, using a cohort-specific NT-proBNP cutoff defined as the median among cases. This threshold approximates a grey-zone where NT-proBNP interpretation is more challenging. NT-proBNP alone performed well (AUC = 0.837). Multi-marker strategies combining miRNAs with NT-proBNP yielded the greatest improvements (ΔAUC up to + 0.111, *p* < 0.001). **B** Net reclassification analysis for patients within the NT-proBNP clinical grey zone (125–450 pg/mL). Baseline classification was based on NT-proBNP thresholds alone; patients were reclassified after adding individual top miRNAs or the composite miRNA signature to NT-proBNP. The miRNA signature achieved the largest net improvement, increasing the proportion of correct rule-in (RI) and rule-out (RO) classifications while minimizing incorrect reclassifications (NRI = 0.645). Gains from individual miRNAs were smaller but still significant (NRI = 0.371–0.516). NRI, Net reclassification improvement, RI, Rule-in, RO, Rule-out

To further benchmark the diagnostic performance of miRNAs against the established biomarker NT-proBNP, we focused on a subset of DCM patients with NT-proBNP values below the median observed in HF cases (790 pg/mL; *n* = 294). This subgroup falls within the clinical grey zone range (125–450 pg/mL) where NT-proBNP interpretation is less definitive and, importantly, avoids the circularity inherent in full-cohort comparisons, as NT-proBNP was part of the adjudication criteria in our study. In this analysis, NT-proBNP alone achieved an AUC of 0.837. When combined with NT-proBNP, the miRNA signature significantly improved classification performance (AUC 0.948; ΔAUC = + 0.111, *p* < 0.001), with smaller but significant gains for individual miRNAs (ΔAUC = + 0.030 to + 0.054, all *p* < 0.05; Fig. 4, Table 5). A complementary reclassification analysis in patients within the clinical NT-proBNP grey zone (125–450 pg/mL, *n* = 62) showed that adding the miRNA signature to NT-proBNP increased the proportion of correct rule-in (RI) and rule-out (RO) classifications while minimizing incorrect reclassifications (Net reclassification improvement (NRI) 0.645, with individual miRNAs yielding NRIs from 0.371*–*0.516; Fig. 4B). For completeness, diagnostic performance metrics for NT-proBNP, individual miRNAs, and combined models in the full DCM cohort are provided in Additional file 3: Table S14, showing the increased performance of NT-proBNP on the full DCM cohort (AUC 0.926), but that integration with miRNAs still yielded significant incremental gains.

**Table 5:** Diagnostic performance of miRNAs and combined models with NT-proBNP in DCM. Performance metrics for individual miRNAs, the DCM aPRIORI miRNA signature, and combined NT-proBNP + miRNA models are shown. Incremental Δ represents the absolute AUC gain of the combined model compared with NT-proBNP alone. P-values were obtained from likelihood-ratio (χ^2^) tests comparing nested logistic regression models

	**Model Performance**			
miRNA	**miRNA AUC**	**Combined NTproBNP AUC**	**Incremental Δ**	***P*-value**
miRNA Signature	0.882	0.948	0.111	< 0.001
hsa-miR-150-5p	0.717	0.886	0.049	< 0.001
hsa-miR-3651	0.741	0.890	0.054	< 0.001
hsa-miR-652-3p	0.725	0.867	0.030	< 0.001
hsa-miR-139-3p	0.714	0.885	0.048	< 0.001
hsa-miR-1275	0.704	0.868	0.031	0.014

*N* = *294 patients (median threshold NT-proBNP* < *790 pg/mL); NT-proBNP alone AUC* = *0.837*

## Discussion

To our knowledge, this is the largest multicentric cohort study involving 2057 patients with genome-wide miRNA assessment in CVD. Our study provides preliminary findings for the diagnostic potential of several miRNAs that have been investigated both in the pathogenesis and as potential biomarkers of CVD. We cover more than 600 published papers and present an Atlas of miRNAs to evaluate the performance of each of the postulated markers in this standardized cohort.

Biomarkers play a fundamental role in the diagnosis of cardiovascular diseases such as ACS and heart failure, and a multiple biomarker strategy may improve diagnostic performance and allow a more personalized approach. Several novel potential biomarkers have been proposed, including numerous miRNAs. Our analysis suggests that individual miRNAs may contribute to CVD discrimination, which is consistent with previous studies that found that miRNAs contribute additive diagnostic information in CVD [10–21]. When these a priori defined miRNAs were combined in miRNA signatures, our aPRIORI models showed very good diagnostic performance with class-specific AUCs ranging from 0.83 to 0.95. Additionally, cardiac biomarkers including miRNAs can provide information on disease severity and prognosis in addition to their diagnostic potential [15, 32, 33]. Integrating the diagnostic ML predictions with key disease features showed that a higher miRNA-probability of disease was associated with a more severe phenotype. This could also lead to redefinition of disease classes based on biomarker expression, as was the case for acute coronary syndromes and troponin biomarkers [34]. The presence of lower levels of miRNA-150 is associated with worse HF severity and outcomes [35, 36]. In line with this, our data identified miR-150-5p as the top differentially expressed miRNA in DCM, with significantly lower expression in cases compared to controls (adjusted *p*-value < 0.001, log₂FC = –0.382). miR-150 regulates cardiomyocyte survival by targeting the transcription factor c-Myb, modulating inflammatory NFκB pathways and directly repressing profibrotic Hoxa4 [37]. Beyond these targets, miR-150 has also been shown to attenuate cardiomyocyte apoptosis via suppression of SPRR1A and to mitigate post-injury remodeling, fibrosis, and inflammation—key processes in the pathophysiology of DCM [38]. A summary of known targets, signaling pathways, and disease processes associated with the promising miRNA candidates in our dataset is provided in Table 6. Beyond single marker effects, the pattern of dysregulated miRNAs in our dataset showed overlap across phenotypes, consistent with shared cardiovascular biology. We observed sets that map onto clinically coherent spectra: *Ischemic spectrum* suggesting continuity from chronic coronary disease to ischemic heart failure; *Pan-cardiomyopathy* consistent with cardiac remodeling processes across aetiologies or *Heart-failure phenotypes* capturing signatures related to advanced ventricular dysfunction.

**Table 6:** Summary of selected miRNA functions and biological relevance in cardiovascular disease

miRNA	Implicated Targets/Pathways	Key Disease Processes	Evidence Summary
miRNA‑150	*SPRR1A*; β‑adrenergic/β‑arrestin signaling; c-Myb, NF-κB, *EGR2*, Hoxa4; *AdiponectinR2*	Cardiac apoptosis, adverse remodeling; heart failure progression (protective), cardiac fibrosis	Downregulated in failing and ischemic hearts, where it normally plays a protective role [39]. miR‑150 directly targets pro-apoptotic SPRR1A, which attenuates cardiomyocyte apoptosis and post-MI remodeling [35]. Accordingly, lower miR‑150 levels are associated with worse HF severity and outcomes. Circulating miR‑150 was associated with 1-year heart failure after acute MI in a clinical study [36]. miR-150 also mediates cardioprotection by functionally repressing profibrotic HOXA4 within the MIAT/miR-150/HOXA4 axis, where its overexpression mitigates maladaptive post-MI remodeling and may underlie the beneficial effects of β-arrestin–biased β-blockers such as carvedilol [37]
miRNA‑126	*SPRED1*, *PIK3R2* (PI3K/Akt pathway); *VCAM-1*; *IRS1, MAPK; VEGF/FGF signaling; Angiopoietin-1/Tie2*	Endothelial function, angiogenesis; anti-inflammatory, vascular integrity	An endothelial-specific miRNA critical for vascular homeostasis. miR‑126 promotes angiogenesis by targeting negative regulators SPRED1 and PI3K-regulatory subunit (PIK3R2), thereby enhancing pro-angiogenic VEGF/PI3K–MAPK signaling [40]. It concomitantly suppresses VCAM-1 expression, reducing leukocyte adhesion and vascular inflammation [41]. Loss of miR-126 leads to endothelial dysfunction (e.g. in diabetes), while higher plasma miR‑126-3p levels are associated with fewer cardiovascular events and considered indicative of endothelial health
miRNA‑19a	*JCAD/ZWINT* (Hippo–YAP pathway); *PTEN*, *BIM* (cell cycle/apoptosis), HIF-1α/VEGF, Hippo/YAP	Endothelial dysfunction (atherosclerosis); cardiac regeneration (post-MI), cardiac fibrosis, hypertrophy, cardiomyocyte proliferation	Member of the miR-17–92 cluster, upregulated during cardiac stress. In vasculature, miR‑19a-3p targets JCAD (junctional protein) to inhibit YAP/TAZ signaling, which blunts TNF-induced endothelial activation and improves endothelial function in atherosclerosis [42]. In the heart, miR‑19a/b are induced in failing myocardium and, when delivered after MI, they enhance cardiomyocyte proliferation and attenuate inflammatory injury [43]. In vivo studies show that intramyocardial or AAV-mediated miR‑19a/19b treatment post-MI reduces infarct size and preserves function, supporting its role in cardiac regeneration [43]
miRNA‑21	*TGFβRIII/Smad*, *Spry1/ERK-MAPK*; *PTEN/Akt*, SPRY1, PDCD4, VEGF pathway targets	Cardiac fibrosis, fibroblast activation; cardiomyocyte apoptosis (protective), angiogenesis, cardiac remodeling	Upregulated after cardiac injury; promotes fibroblast–myofibroblast transition and fibrosis by targeting TGFβRIII and Spry1, activating TGF‑β/Smad and ERK–MAPK signaling [44, 45]. Conversely, miR‑21 exerts cardioprotective effects by suppressing PTEN (enhancing Akt/Bcl-2 signaling and cell survival). Circulating miR‑21 is elevated in heart failure and acute MI patients [46, 47], and nanoparticle delivery of miR‑21 mimics in post-MI mice reduced fibrosis and improved cardiac function [48]
miRNA‑15a	*Smad7/TGF‑β* pathway; *DYRK2/NFAT*; Bcl-2 family (intrinsic apoptosis)	Myocardial fibrosis; cardiomyocyte apoptosis (pathological), oxidative stress	A pro-fibrotic, pro-apoptotic miRNA of the miR-15 family often upregulated in ischemic and hypertrophic hearts [49]. miR‑15a-5p promotes cardiac fibrosis by directly targeting Smad7, an inhibitor of TGF-β signaling, thereby amplifying TGF‑β/Smad pro-fibrotic pathways [50]. It also enhances fibroblast-to-myofibroblast transition via DYRK2 suppression, leading to NFAT dephosphorylation and active fibrosis gene expression [51]
miRNA‑106b	*STAT3* (JAK/STAT pathway); *VEGFA*; *JAK1, MAPK14, PPARy/CREB signaling*	Angiogenesis (negative regulator); atherosclerosis progression, hypertension, vascular dysfunction	miR‑106b-5p exhibits an anti-angiogenic function: it directly targets STAT3, leading to reduced STAT3 levels and impaired endothelial cell tube formation. In endothelial cells, overexpression of miR-106b suppresses multiple angiogenic genes (e.g. STAT3, VEGFA), which may limit plaque neovascularization and contribute to CAD pathology [52]. Accordingly, high serum miR-106b-5p has been proposed as a biomarker for CAD risk and severity [53]
miRNA‑199a	*AGTR1* (AT1 receptor); *MARK4* (microtubule kinase); *CLIC5; HIF-1α; GSK3β; Caveolin-1*	Cardiomyocyte survival (anti-apoptotic); contractility & regeneration	miR-199a-5p targets AGTR1 to blunt angiotensin II-mediated oxidative injury in acute MI, and inhibits MARK4 to improve long-term cardiomyocyte contractility and also directly promotes cardiomyocyte proliferation by targeting cell-cycle regulators (e.g., CLIC5) involved in cell division [54, 55]. Animal models found miR-199a delivery leading to significant ventricular regeneration and functional recovery [56, 57]. New approaches using targeted nanoparticle delivery of miR-199a-5p achieved sustained cardiac repair without adverse effects, underscoring its therapeutic potential [58]

One issue in biomarker studies is the overestimation of discovery approaches. With hundreds of features and often small number of patients, the false positive rate is considerable and results often not reproducible. Hence, this European consortium was established to primarily validate existing markers, also beyond the intellectual property of this consortium—with the aim to foster translation into clinical reality. By carefully synthesizing findings from multiple studies, we have identified a list of miRNAs that could be validated as diagnostic markers in our uniform study. It is important to note, however, that our methodology may be influenced by a selection bias, driven by the tendency within the scientific community to focus on a set of popular miRNAs. This bias is a common challenge in secondary literature reviews, but our analytical methodology, which also included analysis and reporting of genome-wide expressed miRNAs, has the advantage of clearly identifying and acknowledging these biases, thereby increasing the transparency and traceability of conclusions [59].

Technically, miRNAs are promising biomarkers because they are stable with a long half-life in biological samples and easy to detect in available samples without special handling. In areas such as ACS, miRNA-biomarkers have already gained considerable momentum. While recent studies provide a glimpse of the potential for miRNAs to provide additional diagnostic insight—such as identifying cases of unstable angina in patients with ACS—these findings have yet to be validated in larger cohorts [60]. In particular, in areas where there is an urgent need and considerable scope for advanced multi-biomarker diagnostics (e.g., prediction of significant coronary artery stenosis or DCM or even mortality risk), miRNA research is still in the early stages of demonstrating consistent and value-added results. Given the existing literature and the results of our investigation, we advocate the use of multi-faceted molecular datasets that include CVD-specific miRNAs in combination with established markers such as cardiac troponins and nt-proBNP to provide a deeper picture of the disease.

### Study limitations and clinical translation

The most significant limitation of our study is the absence of external validation in independent cohorts for the ML models. While we employed rigorous internal validation methodology including repeated cross-validation and blinded test set evaluation to minimize overfitting, external validation remains essential before clinical implementation. The primary aim of this work was to generate an open-access genome-wide miRNA atlas to inform and prioritize further research. Future studies are needed to experimentally validate key candidates identified here. Our findings should therefore be interpreted as exploratory evidence requiring replication in diverse, independent populations. Additionally, our cohort was predominantly of European ancestry, which may limit generalizability; future studies should include multi-ethnic external validation to ensure broader applicability. The modest specificity observed in several models (ACS 39–43%, DCM 31–36%) underscores the need for signature refinement and external validation before clinical translation. Although our miRNA signature models have been primarily trained for high sensitivity, the observed low specificity levels may reflect several factors. First, while our control population has been carefully selected to exclude overt CVD, it may include individuals with subclinical pathology or cardiovascular risk factors that influence miRNA expression patterns. Second, some miRNAs may be elevated in response to general cardiovascular stress or aging processes present in apparently healthy individuals. Finally, the modest specificity observed suggests that further refinement—such as incorporating clinical multi-modal variables, adjusting classification thresholds, or employing multi-class modeling strategies—may be necessary to enhance clinical applicability, pending external validation.

## Conclusions

In conclusion, our research has shown that specific well-studied miRNAs have the potential to provide clinicians with valuable information to complement, e.g., cardiac troponin levels in the diagnosis and prognostication of CVD. The open-access miRNA atlas generated by this study provides a valuable resource for the scientific community to prioritize candidates for further mechanistic investigation and therapeutic development. Following rigorous external validation and comparison with established biomarkers, the integration of miRNA profiling into clinical practice may ultimately provide additional information for a more personalized approach to patient care.

## Supplementary Information

Additional file 1: Supplementary Methods.

Additional file 2: Supplementary figures. Figure S1 Principal component analysis plots before and after batch-effect correction for each cardiovascular disease group. Each row shows one disease group. Samples are color-coded by recruitment center, with controls indicated separately. Left panels: raw data before batch-effect correction, where clustering patterns and outliers reflect both known and latent sources of technical variation. Right panels: data after batch-effect correction using known covariates (center, microarray chip ID) and surrogate variable analysis (SVA) to capture unknown batch factors. After correction, outlier clustering is markedly reduced and samples are more evenly distributed across principal components, indicating effective removal of both known and latent batch effects. Figure S2 Most frequently mentioned miRNAs. Bar plots show the 20 most frequently mentioned miRNAs identified through the miRetrieve literature search, ranked by their occurrence in unique PubMed abstracts for each cardiovascular disease group: (A) acute coronary syndrome, (B) coronary artery disease, (C) dilated cardiomyopathy, and (D) ischemic cardiomyopathy. Orange bars indicate miRNAs that are also among the top 20 in at least one other disease group (shared signatures), whereas green bars represent miRNAs unique to the disease category shown. The top 3 most commonly mentioned miRNAs are printed bold. Notably, hsa-miR-21, hsa-miR-126, and hsa-miR-133a are among the most frequently reported miRNAs across multiple conditions, while others such as hsa-miR-155 in DCM appear to be more disease-specific. Figure S3 Diagnostic accuracy, calibration and role of the aPRIORI models. A. AUROC curve for the trade-off between sensitivity and specificity. B. A bar chart is given to summarize key performance measures of the trained model on the test set: specificity, sensitivity, positive predictive value, negative predictive value, F1 score, area under the curve and accuracy. C. The calibration plot shows observed and predicted probabilities to assess the agreement between predictions and observations in different deciles of the predicted values. D. Distribution of predicted probabilities for cases versus controls, highlighting the discriminative power of the model. Panel E details feature importance, with means of permutation-based variable importance measures (over 10 permutations) for miRNA features, using 1-AUC as the loss function, to identify the most influential predictors in the aPRIORI model. Figure S4 Diagnostic accuracy, calibration and role of the DISCOVERY models. A. AUROC curve for the trade-off between sensitivity and specificity. B. A bar chart is given to summarize key performance measures of the trained model on the test set: specificity, sensitivity, positive predictive value, negative predictive value, F1 score, area under the curve and accuracy. C. The calibration plot shows observed and predicted probabilities to assess the agreement between predictions and observations in different deciles of the predicted values. D. Distribution of predicted probabilities for cases versus controls, highlighting the discriminative power of the model. Panel E details feature importance, with means of permutation-based variable importance measures (over 10 permutations) for miRNA features, using 1-AUC as the loss function, to identify the most influential predictors in the DISCVOERY models. Figure S5 miRNA expression clusters and disease overlap patterns in cardiovascular disease subtypes. Heatmap displays Z-score normalized expression of the top 25 most significantly dysregulated miRNAs per disease (ACS, CAD, DCM, ICM) using complete linkage clustering with Euclidean distance. Columns represent 100 randomly sampled patients per disease group and 100 controls (n=500 total). Disease groups are color-coded and separated by vertical lines. Row annotations indicate miRNA clusters (left) and disease overlap patterns (right), highlighting miRNAs dysregulated in CAD/ICM (ischemic cardiomyopathy signature), DCM/ICM (heart failure signature), CAD/DCM/ICM (pan-cardiomyopathy signature) or all or across all disease phenotypes (pan-cardiovascular signature). Dendrograms show hierarchical relationships between miRNAs (left) and patients (top). Expression values are row-wise Z-score normalized, with blue indicating lower and red indicating higher relative expression. Figure S6 Kaplan-Meier survival curves stratified by patient group. A. This figure shows the survival probabilities over time for the patients followed up at the University Hospital of Heidelberg. B. C. aPRIORI diagnostic disease model and impact on patient survival. Kaplan-Meier survival curves stratified by median probability and tertiles for ACS, CAD and DCM derived from the aPRIORI models, highlighting the prognostic significance and more severe phenotype of the high-risk group. Ac-mortality, all-cause mortality, ACS, acute coronary syndrome, CAD, coronary artery disease, DCM, dilated cardiomyopathy, FU, follow-up, ICM, ischemic cardiomyopathy, UKHD, University Hospital Heidelberg. Figure S7 Center-stratified diagnostic performance of miRNA signatures across cardiovascular disease phenotypes. ROC curves showing aPRIORI miRNA model performance stratified by recruitment center for (A) acute coronary syndrome, (B) coronary artery disease, (C) dilated cardiomyopathy, and (D) ischemic cardiomyopathy. Each colored line represents a different study center with corresponding AUC values and sample sizes shown in the legend. For centers that recruited only cases for specific phenotypes, random sampled controls from other centers were used for evaluation. Performance was generally consistent across centers, supporting the robustness and generalizability of miRNA signatures across centers. Note: Analyses were performed on full datasets including training samples due to limited holdout test set sizes when stratified by center. Figure S8 Enriched categories from miRNA enrichment analysis: Insights from miEAA’s comprehensive repository. This bar plot displays the top 20 most significantly enriched categories identified through a comprehensive miRNA enrichment analysis, conducted using all 19 miRNA repositories available in MiEAA. The miRNAs analyzed (significantly differentially expressed in our dataset) were found to be highly associated with the disease category Cardiovascular disease. The analysis integrates data from various repositories covering Gene Ontology annotations, KEGG pathways, ncRNA-disease associations, tissue-specific expression, immune cell specificity, and drug interactions. MiEAA, miRNA Enrichment Analysis and Annotation.

Additional file 3: Supplementary tables. Table S1 Patient recruitment by center and disease. This table lists the number of patients recruited at each participating centre who underwent miRNA profiling and the specific diseases for which they were recruited. Recruiting centers were Amsterdam UMC- Department of Cardiology, Universitätsklinikum Frankfurt - Department of Cardiology, Frankfurt am Main, Universitätsklinikum Heidelberg - Department of Cardiology, Institut National de la Santé et de la Recherche Médicale (INSERM), National Scientific Center, Institute of Cardiology Named After Academician M.D. Strazhesko of the National Academy of Medical Sciences of Ukraine - Department of Cardiology, Kyiv, Servicio Madrileño de Salud - Department of Cardiology, Madrid, Spain, Azienda Ospedaliera San Filippo Neri - Department of Cardiology, Rome, Italy, Azienda Ospedaliera di Padova (Università degli Studi di Padova) - Department of Cardiology, Padua, Italy, Ethniko kai Kapodistriako Panepistimio Athinon (National and Kapodistrian University of Athens) - Department of Cardiology, Athens, Greece, Uppsala Universitetssjukhus (Uppsala Universitet) - Department of Cardiology, Uppsala, Sweden. Table S2 Summary of abstract extraction and miRNA Identification. The table provides a detailed breakdown of the total number of PubMed abstracts loaded, the count of abstracts successfully extracted using the miRetrieve package, and the subsequent identification of unique miRNAs. Table S3 Intersections of miRNAs in Distinct Cardiovascular Phenotypes. This table details the shared top 50 literature miRNAs per disease as indicated by the weighted biomarker score retrieved via miRetrieve across different cardiovascular conditions. This summary provides insights into the common miRNAs observed in various cardiovascular diseases. Table S4 Selected top 50 miRNAs identified from the literature search and grouped by disease of interest. The weighted biomarker score was calculated using the miRetrieve package, taking into account the number of associated PMIDs and biomarker keywords. Univariate AUCs were calculated for each miRNA and miRNAs ranked accordingly. AUROC; area under the receiver operating characteristic curve, PMID; PubMed unique identifier. Table S5 Result metrics for the comparison of ACS versus control. This table shows the statistical results from matched analysis and logistic regression, adjusted for age and sex. The 'ttest_rawp' column shows the raw p-values from the matched analysis t-test, 'ttest_adjp' shows the Bonferroni-Holm adjusted p-values, 'glm_rawp' shows the raw p-values from the logistic regression model, 'AUC' shows the area under the curve values reflecting the diagnostic accuracy, and 'log2FoldChange' shows the magnitude of differential expression on a logarithmic scale. Table S6 Result metrics for the comparison of CAD versus control. Table S7 Result metrics for the comparison of DCM versus control. Table S8 Result metrics for the comparison of ICM versus control. Table S9 Intersections of differentially expressed miRNAs in distinct cardiovascular phenotypes. This table details the shared top differentially expressed miRNAs per disease as indicated by an adjusted p-value of 0.05 and an abs(log2 Fold Change) > 0.2 across different cardiovascular conditions. This summary provides insights into the common miRNAs observed in various cardiovascular diseases. This data-driven table mirrors Supplementary Table S3 (literature-based overlaps), enabling comparison between reported and observed patterns. “Number of miRNAs” indicates the total count for each intersection. Table S10 ACS risk groups stratified by aPRIORI ACS model probabilities. This table provides the patient demographics and laboratory values stratified by tertiles of probability for ACS derived from the aPRIORI model, analysing only patients from the University Hospital of Heidelberg. ACS, acute coronary syndrome. Table S11 DCM risk groups stratified by aPRIORI DCM model probabilities. This table provides the patient demographics and laboratory values stratified by tertiles of probability for DCM derived from the aPRIORI model, analysing only patients from the University Hospital of Heidelberg. DCM, dilated cardiomyopathy. Table S12 CAD risk groups stratified by aPRIORI DCM model probabilities (tertiles). This table provides the patient demographics and laboratory values stratified by tertiles of probability for CAD derived from the aPRIORI model, analysing only patients from the University Hospital of Heidelberg. CAD, coronary artery disease. Table S13 CAD risk groups stratified by aPRIORI CAD model probabilities (median). This table provides the patient demographics and laboratory values stratified by median probability values for CAD derived from the aPRIORI model, analysing only patients from the University Hospital of Heidelberg. CAD, coronary artery disease. Table S14 Diagnostic performance of miRNAs and combined models with NT-proBNP in all DCM patients. Performance metrics for individual miRNAs, the DCM aPRIORI miRNA signature, and combined NT-proBNP + miRNA models are shown. Incremental Δ represents the absolute AUC gain of the combined model compared with NT-proBNP alone. P-values were obtained from likelihood-ratio (χ²) tests comparing nested logistic regression models.

## Data Availability

De-identified, normalized miRNA intensity matrices and associated metadata (age, sex, diagnosis) generated in this study have been deposited in the European Genome-phenome Archive (EGA) under accession number EGAS00001008346 ([https://ega-archive.org/studies/EGAS00001008346]). Access to the data is controlled and can be obtained by applying through the EGA Data Access Committee.

## Abbreviations

ACS: Acute coronary syndrome
aPRIORI-Model: A-priori miRNA Model
BMI: Body mass index
CAD: Coronary artery disease / chronic coronary artery disease
CRP: C-reactive protein
CVD: Cardiovascular disease
DCM: Dilated cardiomyopathy
EGA: European Genome–phenome Archive
HF: Heart failure
hs-TroponinT: High-Sensitivity Troponin T
ICM: Ischemic cardiomyopathy
IQR: Interquartile range
log₂FC: Log₂ fold change
LVEDD: Left ventricle end-diastolic diameter
LVEF: Left ventricular ejection fraction / left ventricular systolic function
MeSH: Medical Subject Headings
MiEAA: MiRNA Enrichment Analysis and Annotation
miRNA: MicroRNA
ML: Machine Learning
NFκB: Nuclear Factor kappa-light-chain-enhancer of activated B cells
NRI: Net reclassification improvement
NT-proBNP: N-terminal pro-B-type natriuretic peptide
PCA: Principal component analysis
RI: Rule-in
RO: Rule-out
ROC-AUC: Receiver operating characteristic area under the curve
STARD: Standards for Reporting of Diagnostic Accuracy
SVA: Surrogate variable analysis
