# Supplementary material for: The European BestAgeing Study on microRNA candidates reveals distinct signatures with diagnostic and prognostic potential in cardiovascular disease
Source: BMC Med. 2025 Nov 28;23:670. doi: 10.1186/s12916-025-04502-3 (PMC12670801; doi:10.1186/s12916-025-04502-3)

**Additional file 1**

**Supplementary Methods**

**Supplementary Methods**

**Defined Inclusion and Exclusion Criteria by BestAgeing study**

The recruitment targeted individuals diagnosed with coronary artery disease (CAD), ischemic cardiomyopathy (ICM), dilated cardiomyopathy (DCM), acute coronary syndrome (ACS), and a control cohort of healthy individuals.

Phenotype specific inclusion criteria

1. Acute coronary syndrome (ACS)

New or crescendo typical angina or at least two of the following:

- typical chest pain,
- elevated hsTnT (>99% percentile),
- typical ECG alterations

1. Coronary artery disease (CAD)

Atherosclerotic lesions < 50 % in at least one coronary artery as judged by coronary angiography and symptoms of angina pectoris, or atherosclerotic lesions >= 50% in at least one coronary artery as judged by coronary angiography.

1. Dilated Cardiomyopathy (DCM)

DCM was diagnosed based on statements by the Collaborative Research Group of the European Human and Capital Mobility Project on Familial Dilated Cardiomyopathy in 1999 and by the European Society of Cardiology (ESC) Working Group on Myocardial and Pericardial Diseases in 2008 (1,2).

- Genetic and/or idiopathic origin (with or without arteriosclerosis, without relevant stenosis)
- Asymptomatic and symptomatic HF
- Ejection fraction < 45 % (measured by echocardiography or magnetic resonance imaging or angiography)
- Left ventricle end-diastolic diameter >117 % of predicted value according to age and body surface area in echocardiography (Henry equation for predicted LVEDD = 45,3 x 1/3 BSA - 0,03 x Age - 7,2)
- Absence of causal factors such as coronary artery disease or intrinsic valvular disease, documented myocarditis, systemic disease, sustained rapid supraventricular arrhythmia or congenital malformation.

1. Ischemic cardiomyopathy (ICM)

- Ejection fraction < 45 % (measured by echocardiography or magnetic resonance imaging or angiography)
- Asymptomatic or symptomatic patients
- DCM criteria not fulfilled

1. Heathy control patients (Control)

Patients in whom through diagnostic work up cardiovascular disease the above mentioned diagnoses and the below listed exclusion criteria (medical history, physical examination, laboratory values) were ruled out

General exclusion criteria

- Clinically significant concurrent illness or psychological, familial, sociological, geographical or other concomitant condition that would not permit adequate informed consent
- Patient refusal to participate in the study
- History of coronary insufficiency, myocardial infarction, CAD and peripheral artery disease, all within 3 months of study inclusion
- Liver cirrhosis
- Renal insufficiency (estimated glomerular filtration rate (eGFR) < 30 ml/min/1,73qm)
- Anemia (Hb < 9 g/dl)
- Active carcinoma
- Exacerbated COPD

Phenotype specific exclusion criteria

1. Dilated cardiomyopathy (DCM)

Infectious, autoimmune and/or toxic causes of cardiomyopathy

1. Heathy control patients (Control)

Medical history of:

- Previous myocardial infarction or other heart disease, previous percutaneous coronary intervention or open heart surgery, chronic heart failure, angina pectoris or other heart disease
- Severe co-morbidities (e.g. renal insufficiency)
- Active carcinoma
- COPD or other serious lung disease
- Inflammatory disease (e.g. rheumatoid arthritis)
- Anemia (Hb < 10 g/dl)
- Diabetes mellitus*
- Uncontrolled hypertension

Physical examination:

- Blood pressure > 140/90mmHg
- Body mass index (BMI) > 30 or < 18

Laboratory values:

- NTproBNP/BNP > age adjusted cut-offs
- eGFR < 60

* 17 patients were included in the control group despite the presence of diabetes mellitus

The recruitment of patients according to their respective diagnosis in each center is detailed in Additional file: Table 1. In total 2,057 patients were measured in this study. All samples were measured in Heidelberg.

**Text mining and literature miRNAs**

A comprehensive literature search was conducted to identify studies focusing on the role of microRNAs in cardiovascular disease and their associated diagnostic biomarkers.

Search query (as performed on June 14^th^, 2023):

1. Acute Coronary Syndrome

'(("MicroRNAs"[Mesh] OR "miRNAs"[Title/Abstract] OR "micro RNA"[Title/Abstract])

AND ("Acute Coronary Syndrome"[Mesh] OR "Acute Coronary Syndrome" OR "Myocardial Infarction"[Mesh])

AND ("Biomarkers"[Mesh] OR "Biomarkers"[Title/Abstract])

AND ("Diagnosis"[Mesh] OR "Diagnostic"[Title/Abstract] OR "Diagnostic Techniques and Procedures"[Mesh] OR "Diagnosis"[Title/Abstract])

AND "Humans"[Mesh] AND 2000:2023[DP]

NOT ("Animals"[Mesh] NOT "Humans"[Mesh] OR "Cell Line"[Mesh] OR "Animal Experimentation"[Mesh]

OR "In Vitro Techniques"[Mesh] OR "Cell Culture Techniques"[Mesh] OR "animal model"[Title/Abstract] OR "laboratory study"[Title/Abstract] OR "cell line"[Title/Abstract]))'

1. Coronary Artery Disease

'(("MicroRNAs"[Mesh] OR "miRNAs"[Title/Abstract] OR "microRNA"[Title/Abstract] OR "miRNA"[Title/Abstract])

AND ("Coronary Artery Disease"[Mesh] OR "Coronary Artery Disease" ))

AND ("Biomarkers"[Mesh] OR "Biomarkers"[Title/Abstract])

AND ("Diagnosis"[Mesh] OR "Diagnostic"[Title/Abstract] OR "Diagnostic Techniques and Procedures"[Mesh] OR "Diagnosis"[Title/Abstract])

AND "Humans"[Mesh] AND 2000:2023[DP]

NOT ("Animals"[Mesh] NOT "Humans"[Mesh] OR "Cell Line"[Mesh] OR "Animal Experimentation"[Mesh]

OR "In Vitro Techniques"[Mesh] OR "Cell Culture Techniques"[Mesh] OR "animal model"[Title/Abstract] OR "laboratory study"[Title/Abstract] OR "cell line"[Title/Abstract]))'

1. Dilated Cardiomyopathy

‘(("MicroRNAs"[Mesh] OR "miRNAs"[Title/Abstract] OR "microRNA"[Title/Abstract] OR "miRNA"[Title/Abstract])

AND ("Cardiomyopathy, Dilated"[Mesh] OR "Cardiomyopathy, Dilated" OR "dilated cardiomyopathy"))

AND ("Biomarkers"[Mesh] OR "Biomarkers"[Title/Abstract])

AND ("Diagnosis"[Mesh] OR "Diagnostic"[Title/Abstract] OR "Diagnostic Techniques and Procedures"[Mesh] OR "Diagnosis"[Title/Abstract])

AND "Humans"[Mesh] AND 2000:2023[DP]

NOT ("Animals"[Mesh] NOT "Humans"[Mesh] OR "Cell Line"[Mesh] OR "Animal Experimentation"[Mesh]

OR "In Vitro Techniques"[Mesh] OR "Cell Culture Techniques"[Mesh] OR "animal model"[Title/Abstract] OR "laboratory study"[Title/Abstract] OR "cell line"[Title/Abstract]))'

1. Ischemic cardiomyopathy

'(("MicroRNAs"[Mesh] OR "miRNAs"[Title/Abstract] OR "microRNA"[Title/Abstract] OR "miRNA"[Title/Abstract])

AND ("Heart Failure"[Mesh] OR "Heart Failure, Systolic"[Mesh] OR "Heart Failure, Systolic" OR "ischemic cardiomyopathy"))

AND ("Biomarkers"[Mesh] OR "Biomarkers"[Title/Abstract])

AND ("Diagnosis"[Mesh] OR "Diagnostic"[Title/Abstract] OR "Diagnostic Techniques and Procedures"[Mesh] OR "Diagnosis"[Title/Abstract])

AND "Humans"[Mesh] AND 2000:2023[DP]

NOT ("Animals"[Mesh] NOT "Humans"[Mesh] OR "Cell Line"[Mesh] OR "Animal Experimentation"[Mesh]

OR "In Vitro Techniques"[Mesh] OR "Cell Culture Techniques"[Mesh] OR "animal model"[Title/Abstract] OR "laboratory study"[Title/Abstract] OR "cell line"[Title/Abstract]))'

All PubMed Abstracts were downloaded and further processed with the R package *miRetrieve*. In detail the packages functions `subset_research()` and `extract_mir_df()` were used to extract miRNA names from abstracts of research articles only. Abstracts from other article types such as *Review* or *Letter* were dropped. The threshold argument for how often a miRNA must be mentioned in an abstract to be extracted was set to 1. Ultimately, abstracts not containing any miRNA names were dropped. To see how many abstracts were retrieved and filtered in the following, see Additional file 3: *Table 2*. Within the *miRetrieve* framework, a biomarker integer score was calculated for each abstract to indicate the potential use of specific miRNAs as biomarkers. This scoring was based on the presence of the following keywords: "circulating," "biomarker," "bio-marker," "extracellular vesicles," "exosomes," "exosomal," "diagnostic," "biological marker," "serum," and "plasma." The occurrence of these keywords within the abstracts helped assess the relevance of the miRNAs to the diseases in question. The calculated biomarker scores were then combined with the number of abstracts associated with a specific miRNA for each disease, thus weighting the a-priori miRNA value. This combined scoring approach allowed for a more nuanced evaluation of the miRNAs' potential significance in the context of the specific cardiovascular diseases being studied.

**Quantile Normalization**

We applied standard quantile normalization to make the distribution of all samples identical. Specifically, we applied quantile normalize of all distributions without a reference distribution. All samples are sorted and then the average of all values is assigned. Thereby, the highest value in all samples becomes the average of the highest values. The second highest value becomes the average of all second highest expression values. This process is iterated over all miRNAs.

**miRNA filtering and batch correction**

We started our analysis on 2,549 miRNAs from the miRBase v21. Following an initial analysis, we filtered those miRNAs in order to improve the signal to noise ratio. We used the *AgiMicroRNA* package to perform background correction, quantile normalization and summarization of all probesignals per feature/microRNA. Following that we filtered miRNAs according to their detection rate: features had to be detected in 90 % of the samples in either the disease group or the control group. The final data set contained 440, 436, 436, 453 likely stable miRNAs for the ACS/control, the CAD/control, the DCM/control and the ICM/control comparison, respectively. We corrected for technical batch effects on the log-transformed quantile normalized expression data for the biochip identification number. As all samples were measured at *Hummingbird Diagnostics* in Heidelberg, there was no need for batch removal of the site where the sample was profiled. The batch effects were removed using a linear model via the *removeBatchEffect* method of the limma package 3 (version 3.58.1).

**Diagnostic machine learning analysis**

We implemented a benchmarking ML approach to analyze the dataset using the *tidymodels* framework.For each disease the dataset was first strictly split into 75 % training and 25 % test sets and stratified by the disease variable to ensure that both sets retained a representative proportion of each disease category. The training set was cross validated 5 times and repeated 10 times. The cross-validation was again stratified by the disease variable to ensure consistency in the representation of different disease classes across all folds. This approach reduces the variance of the model's performance estimate, leading to a more robust selection of hyperparameters and an accurate evaluation of the model's predictive capacity. Preprocessing of the selected miRNA features and the covariates age and sex included removal of zero variance predictors, removal of highly correlated features (threshold correlation=0.8), and standardization and normalization of numerical predictors. To further increase the robustness of our findings, in addition to adjusting for covariates, we also matched for age and sex, as the baseline characteristics showed a clear deviation from the control group. This was done using the *MatchIt* package in R, using the „nearest” method. Latin Hypercube sampling was used to create a hyperparameter grid for the benchmarked models penalized logistic regression, XGBoost and Random Forest. Penalized logistic regression (L1/L2) was included as an interpretable linear baseline, offering embedded regularization to mitigate overfitting and reduce variance in high-dimensional settings. Random Forest and XGBoost, both ensemble tree-based methods, were selected due to their capacity to model complex, non-linear interactions between features without strong parametric assumptions. These algorithms are robust to noise, automatically handle variable interactions, and provide reliable measures of feature importance for model interpretation. Models were then evaluated using the receiver operating characteristic area under the curve (ROC-AUC) metric during hyperparameter tuning. A race-based resampling method was implemented, enabling parallel computation and additional control over the selection process, such as burn-in iterations, number of ties, significance level, and randomization. The best hyperparameters were then used with a finalized workflow that was fitted on the test data. We computed AUC, accuracy, sensitivity and specificity, positive and negative predictive values, and the harmonic mean of precision and recall (F1 score) on the blinded test data. Calibration plots were constructed to visualize the agreement between predicted probabilities and observed outcomes. We then used a model agnostic approach to compute global variable importance by permutating features. If shuffling a column causes a large degradation in model performance, it is important and vice versa.

**Penalized Logistic Regression (L1/L2 Regularization)**

Selected as an interpretable linear baseline with embedded feature selection capabilities.

Specific Strengths considered:

- High-dimensional data handling: genome-wide miRNA assessments contain hundreds to thousands of molecular features with relatively smaller sample sizes, creating the classical "p >> n" problem that penalized regression specifically addresses
- Multicollinearity management:  strong intercorrelations is effectively managed by L1/L2 regularization
- Clinical interpretability: Linear coefficients provide direct, clinically interpretable effect sizes and directions of association
- Embedded feature selection: L1 regularization (LASSO) automatically identifies the most predictive features, reducing model complexity and focusing on clinically relevant features

**Random Forest**

Selected for robust ensemble prediction with automatic variable interaction detection.

Specific strengths considered:

- Non-linear relationship modeling: miRNAs may involve complex, non-linear interactions (e.g., threshold effects, multiplicative interactions between miRNA expression profiles)
- Noise robustness: Biological measurements inherently contain technical and biological noise; Random Forest's averaging across multiple trees provides natural noise reduction
- Interaction capture: Automatically detects and models interactions between miRNAs without requiring a priori specification, important for discovering novel biomarker combinations
- Feature importance ranking: Provides interpretable variable importance measures to identify key features driving the model’s predictions

**XGBoost (Extreme Gradient Boosting)**

Selected for maximum predictive performance through advanced ensemble optimization.

Specific Strengths considered:

- Sequential error correction: Gradient boosting iteratively corrects prediction errors, particularly valuable for capturing subtle signals that may be masked in initial models
- Advanced regularization: Built-in L1/L2 regularization prevents overfitting in high-dimensional biomarker spaces while maintaining predictive power
- Computational efficiency: Optimized implementation allows analysis of large-scale biomarker datasets common in cardiovascular genomics and proteomics studies
- Feature importance with interactions: Provides sophisticated feature importance measures that account for both main effects and interaction contributions

**Multimarker analysis using NT-proBNP and miRNAs**

To evaluate the incremental diagnostic value of circulating miRNAs beyond NT-proBNP in situations where NT-proBNP interpretation is less definitive. NT-proBNP was incorporated into the adjudication criteria for DCM and ICM diagnosis in this study, potentially inflating its apparent diagnostic accuracy in the full cohort. Restricting the analysis to intermediate NT-proBNP values reduces this risk of circularity and reflects a recognized diagnostic challenge in clinical practice, where NT-proBNP is often less specific due to factors such as age, obesity, and comorbidities. We identified DCM patients with available NT-proBNP concentrations and used the median NT-proBNP level as cutoff for our analysis. We fitted logistic regression models adjusted for age and sex to discriminate cases from controls within this gray-zone subgroup using:

1. NT-proBNP alone
2. Individual top differentially expressed miRNAs for the phenotype (identified in prior univariate analysis)
3. Pre-specified miRNA signatures developed in this study
4. Combined models including NT-proBNP, the miRNA signature, age, and sex

For each model, we calculated the ROC-AUC with 95% CIs.

**Reclassification Analysis**

A reclassification analysis was conducted to quantify whether adding miRNAs to NT-proBNP improved diagnostic classification within the clinical gray zone. Patients with NT-proBNP concentrations in the clinical gray-zone range (125–450 pg/mL) were included. Those with NT-proBNP <125 pg/mL (clinical rule-out) or >450 pg/mL (clinical rule-in) were not reclassified. Only patients with measured NT-proBNP values were included; controls with imputed NT-proBNP values (equal to the stored imputation constant) were excluded.

Initial classification was based solely on clinical NT-proBNP thresholds:

- Rule-out: <125 pg/mL
- Grey zone: 125–450 pg/mL
- Rule-in: >450 pg/mL

We fitted a logistic regression model adjusted for age and sex to classify grey-zone patients as cases or controls. Predicted probabilities were interpreted as: Rule-in (RI) probability >0.70; Rule-out (RO) probability <0.30; no reclassificatio: probability between 0.30 and 0.70. Each patient was assigned to one of five categories: Correct RI (predicted rule-in, true case), correct RO (predicted rule-out, true control), incorrect RI (predicted rule-in, true control), incorrect rule-out (predicted rule-out, true case), no reclassification. We then calculated the net reclassification improvement (NRI). Percentages for each category were reported.


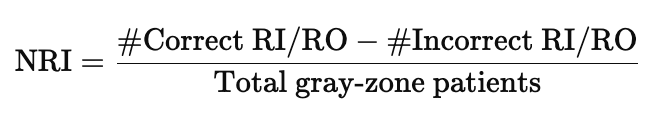

Supplement: Supplementary file 1 — Additional file 1: Supplementary Methods. [file 12916_2025_4502_MOESM1_ESM.docx]
