# Supplementary material for: The European BestAgeing Study on microRNA candidates reveals distinct signatures with diagnostic and prognostic potential in cardiovascular disease
Source: BMC Med. 2025 Nov 28;23:670. doi: 10.1186/s12916-025-04502-3 (PMC12670801; doi:10.1186/s12916-025-04502-3)
Supplement: Supplementary file 2 — Additional file 2: Supplementary figures. Figure S1 Principal component analysis plots before and after batch-effect correction for each cardiovascular disease group. Each row shows one disease group. Samples are color-coded by recruitment center, with controls indicated separately. Left panels: raw data before batch-effect correction, where clustering patterns and outliers reflect both known and latent sources of technical variation. Right panels: data after batch-effect correction using known covariates (center, microarray chip ID) and surrogate variable analysis (SVA) to capture unknown batch factors. After correction, outlier clustering is markedly reduced and samples are more evenly distributed across principal components, indicating effective removal of both known and latent batch effects. Figure S2 Most frequently mentioned miRNAs. Bar plots show the 20 most frequently mentioned miRNAs identified through the miRetrieve literature search, ranked by their occurrence in unique PubMed abstracts for each cardiovascular disease group: (A) acute coronary syndrome, (B) coronary artery disease, (C) dilated cardiomyopathy, and (D) ischemic cardiomyopathy. Orange bars indicate miRNAs that are also among the top 20 in at least one other disease group (shared signatures), whereas green bars represent miRNAs unique to the disease category shown. The top 3 most commonly mentioned miRNAs are printed bold. Notably, hsa-miR-21, hsa-miR-126, and hsa-miR-133a are among the most frequently reported miRNAs across multiple conditions, while others such as hsa-miR-155 in DCM appear to be more disease-specific. Figure S3 Diagnostic accuracy, calibration and role of the aPRIORI models. A. AUROC curve for the trade-off between sensitivity and specificity. B. A bar chart is given to summarize key performance measures of the trained model on the test set: specificity, sensitivity, positive predictive value, negative predictive value, F1 score, area under the curve and accuracy. C. The ca [file 12916_2025_4502_MOESM2_ESM.pdf]

Figure S1

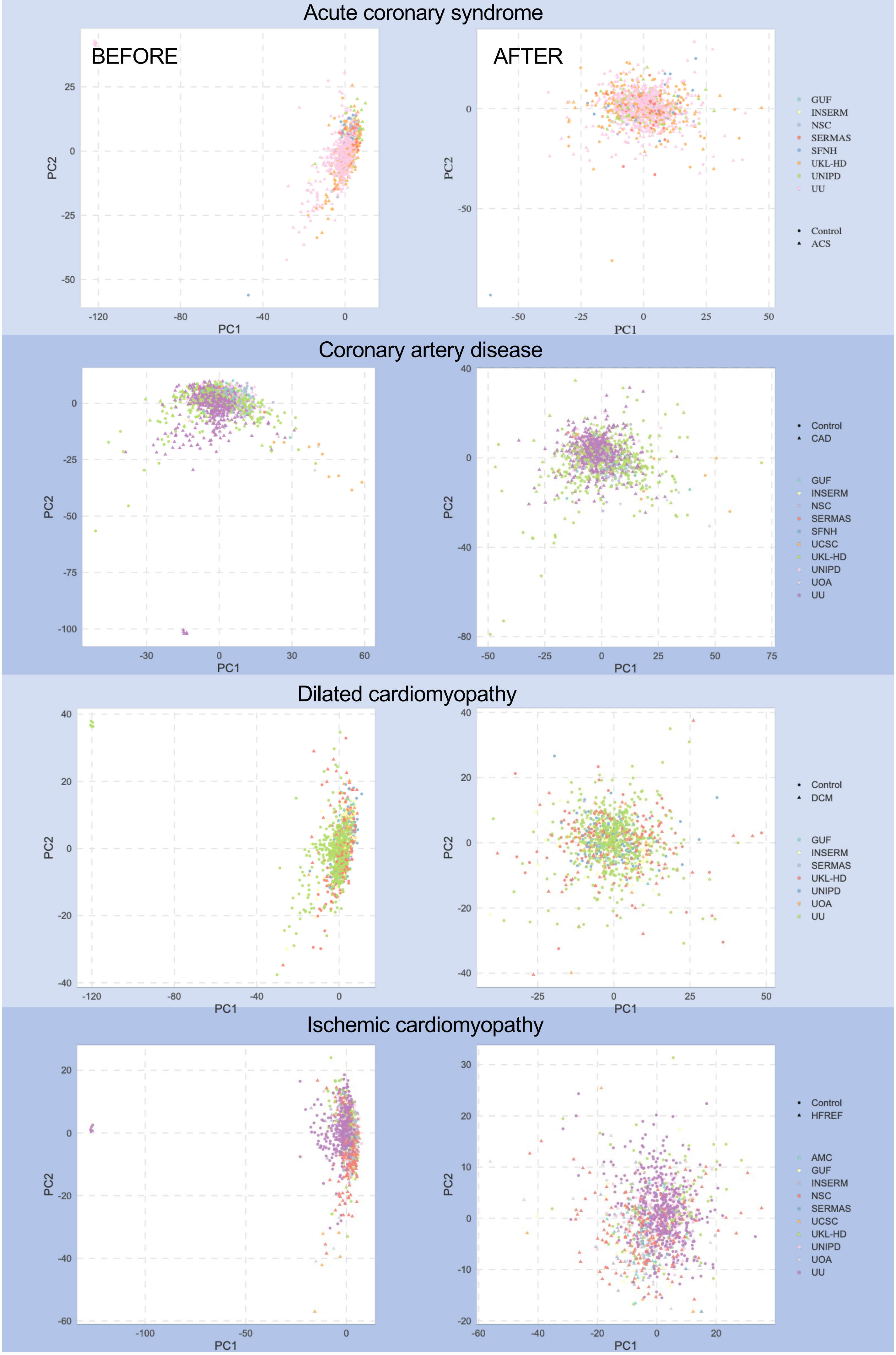

Acute coronary syndrome
BEFORE
AFTER
PC1
PC2
GUF
INSERM
NSC
SERMAS
SFNH
UKL-HD
UNIPD
UU
Control
ACS
Coronary artery disease
Control
CAD
GUF
INSERM
NSC
SERMAS
SFNH
UCSC
UKL-HD
UNIPD
UOA
UU
Dilated cardiomyopathy
Control
DCM
GUF
INSERM
SERMAS
UKL-HD
UNIPD
UOA
UU
Ischemic cardiomyopathy
Control
HFREF
AMC
GUF
INSERM
NSC
SERMAS
UCSC
UKL-HD
UNIPD
UOA
UU

Figure S2

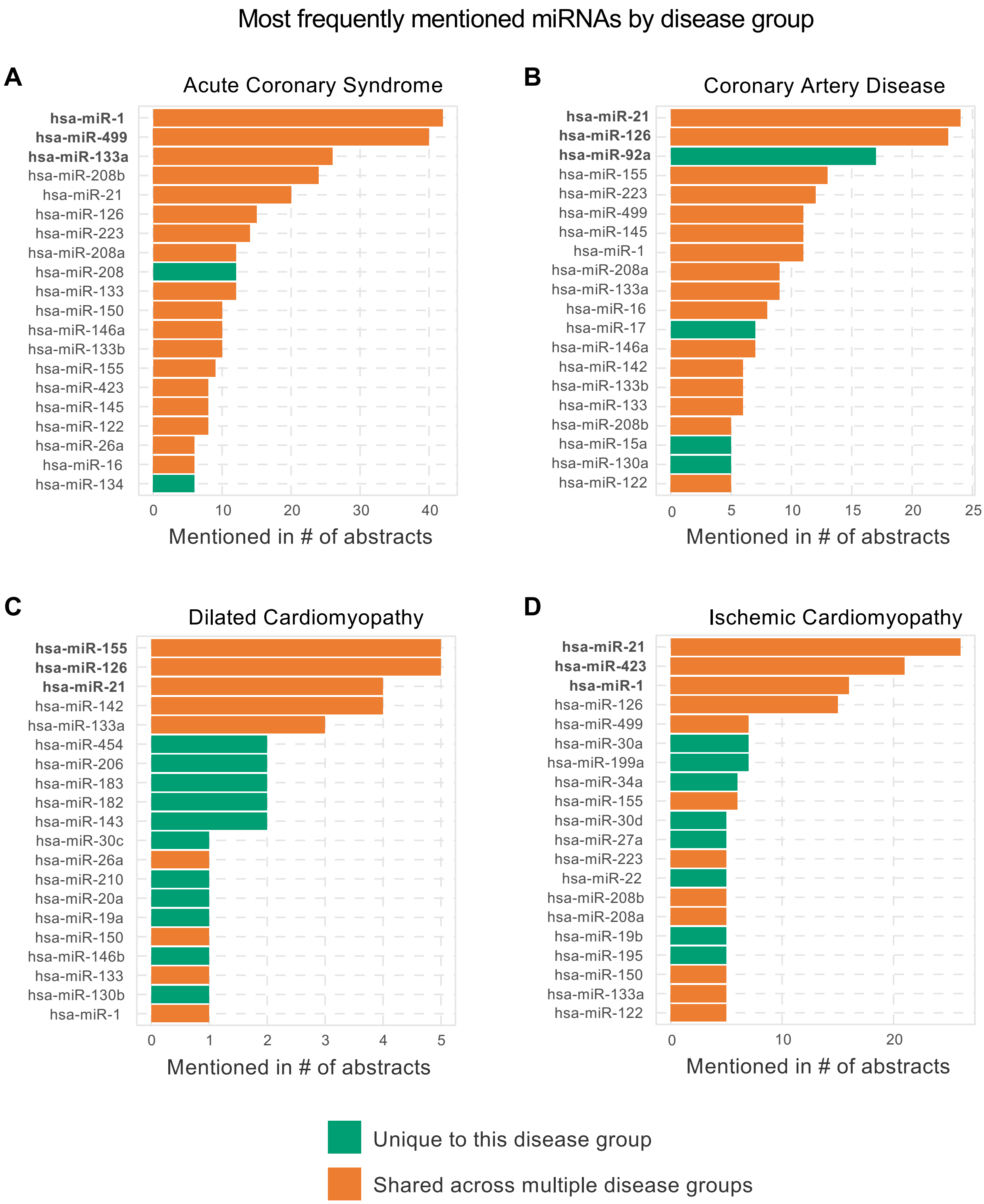

Most frequently mentioned miRNAs by disease group
A
Acute Coronary Syndrome
hsa-miR-1
hsa-miR-499
hsa-miR-133a
hsa-miR-208b
hsa-miR-21
hsa-miR-126
hsa-miR-223
hsa-miR-208a
hsa-miR-208
hsa-miR-133
hsa-miR-150
hsa-miR-146a
hsa-miR-133b
hsa-miR-155
hsa-miR-423
hsa-miR-145
hsa-miR-122
hsa-miR-26a
hsa-miR-16
hsa-miR-134
0
10
20
30
40
Mentioned in # of abstracts
B
Coronary Artery Disease
hsa-miR-21
hsa-miR-126
hsa-miR-92a
hsa-miR-155
hsa-miR-223
hsa-miR-499
hsa-miR-145
hsa-miR-1
hsa-miR-208a
hsa-miR-133a
hsa-miR-16
hsa-miR-17
hsa-miR-146a
hsa-miR-142
hsa-miR-133b
hsa-miR-133
hsa-miR-208b
hsa-miR-15a
hsa-miR-130a
hsa-miR-122
0
5
10
15
20
25
Mentioned in # of abstracts
C
Dilated Cardiomyopathy
hsa-miR-155
hsa-miR-126
hsa-miR-21
hsa-miR-142
hsa-miR-133a
hsa-miR-454
hsa-miR-206
hsa-miR-183
hsa-miR-182
hsa-miR-143
hsa-miR-30c
hsa-miR-26a
hsa-miR-210
hsa-miR-20a
hsa-miR-19a
hsa-miR-150
hsa-miR-146b
hsa-miR-133
hsa-miR-130b
hsa-miR-1
0
1
2
3
4
5
Mentioned in # of abstracts
D
Ischemic Cardiomyopathy
hsa-miR-21
hsa-miR-423
hsa-miR-1
hsa-miR-126
hsa-miR-499
hsa-miR-30a
hsa-miR-199a
hsa-miR-34a
hsa-miR-155
hsa-miR-30d
hsa-miR-27a
hsa-miR-223
hsa-miR-22
hsa-miR-208b
hsa-miR-208a
hsa-miR-19b
hsa-miR-195
hsa-miR-150
hsa-miR-133a
hsa-miR-122
0
10
20
Mentioned in # of abstracts
Unique to this disease group
Shared across multiple disease groups

Figure S3

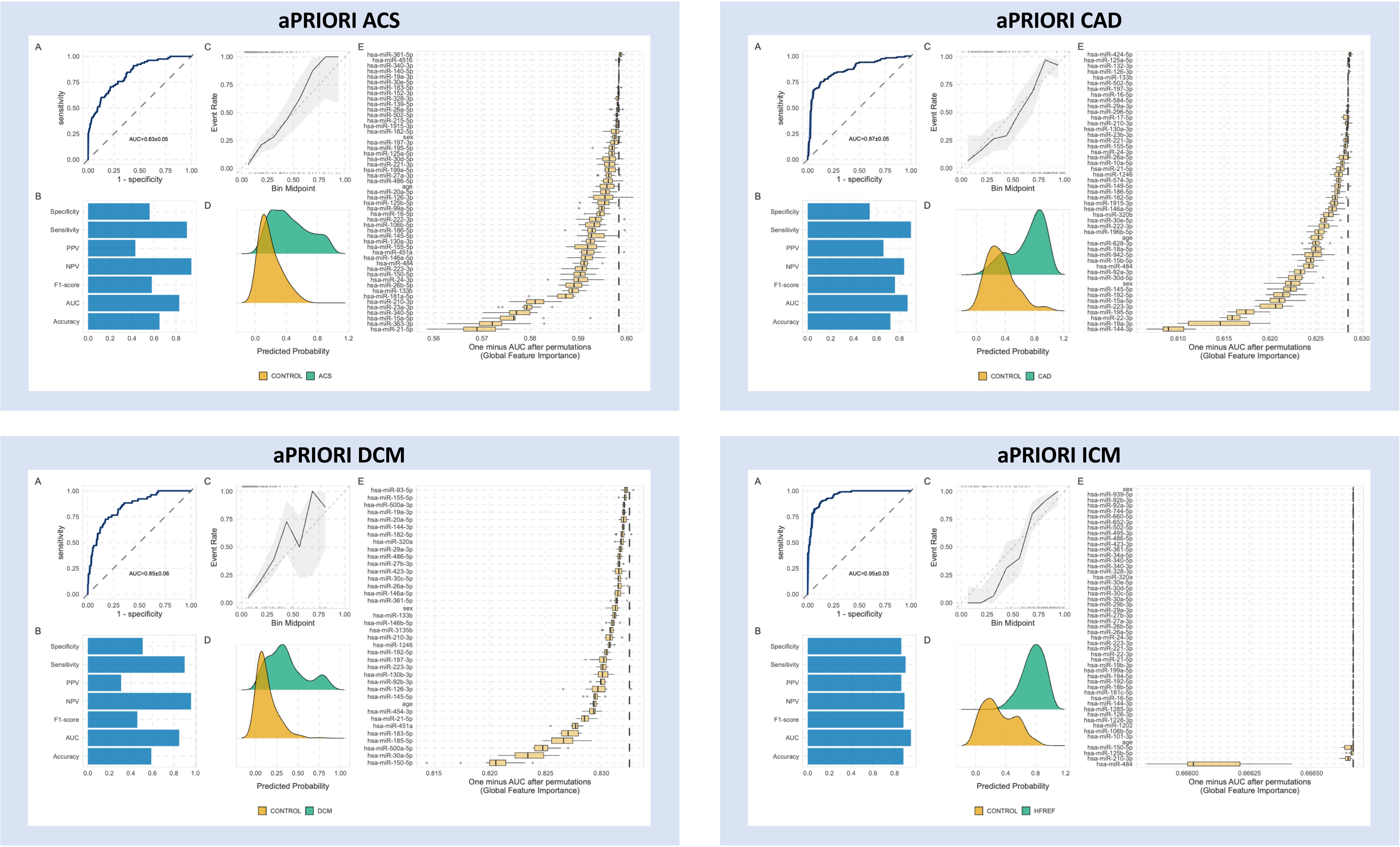

aPRIORI ACS
AUC=0.83±0.05
sensitivity
1 - specificity
Event Rate
Bin Midpoint
Specificity
Sensitivity
PPV
NPV
F1-score
AUC
Accuracy
Predicted Probability
One minus AUC after permutations
(Global Feature Importance)
CONTROL
ACS
aPRIORI CAD
AUC=0.87±0.05
CONTROL
CAD
aPRIORI DCM
AUC=0.85±0.06
CONTROL
DCM
aPRIORI ICM
AUC=0.95±0.03
CONTROL
HFREF

Figure S4

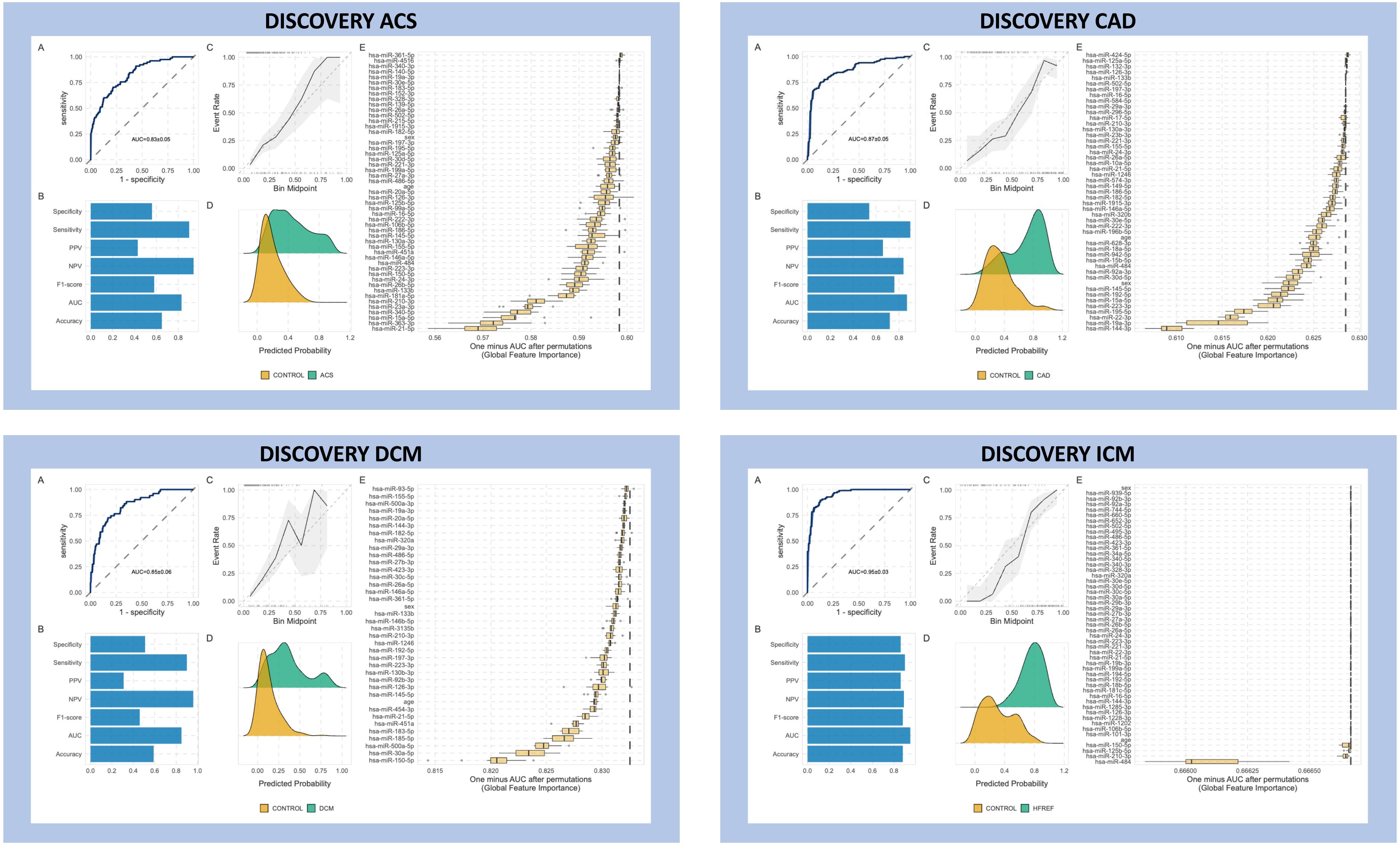

DISCOVERY ACS
AUC=0.83±0.05
DISCOVERY CAD
AUC=0.87±0.05
DISCOVERY DCM
AUC=0.85±0.06
DISCOVERY ICM
AUC=0.95±0.03
A
B
C
D
E
sensitivity
1 - specificity
Event Rate
Bin Midpoint
Specificity
Sensitivity
PPV
NPV
F1-score
AUC
Accuracy
Predicted Probability
One minus AUC after permutations
(Global Feature Importance)
CONTROL
ACS
CAD
DCM
HFREF

Figure S5

Hierarchical clustering heatmap of miRNA expression patterns across cardiovascular diseases

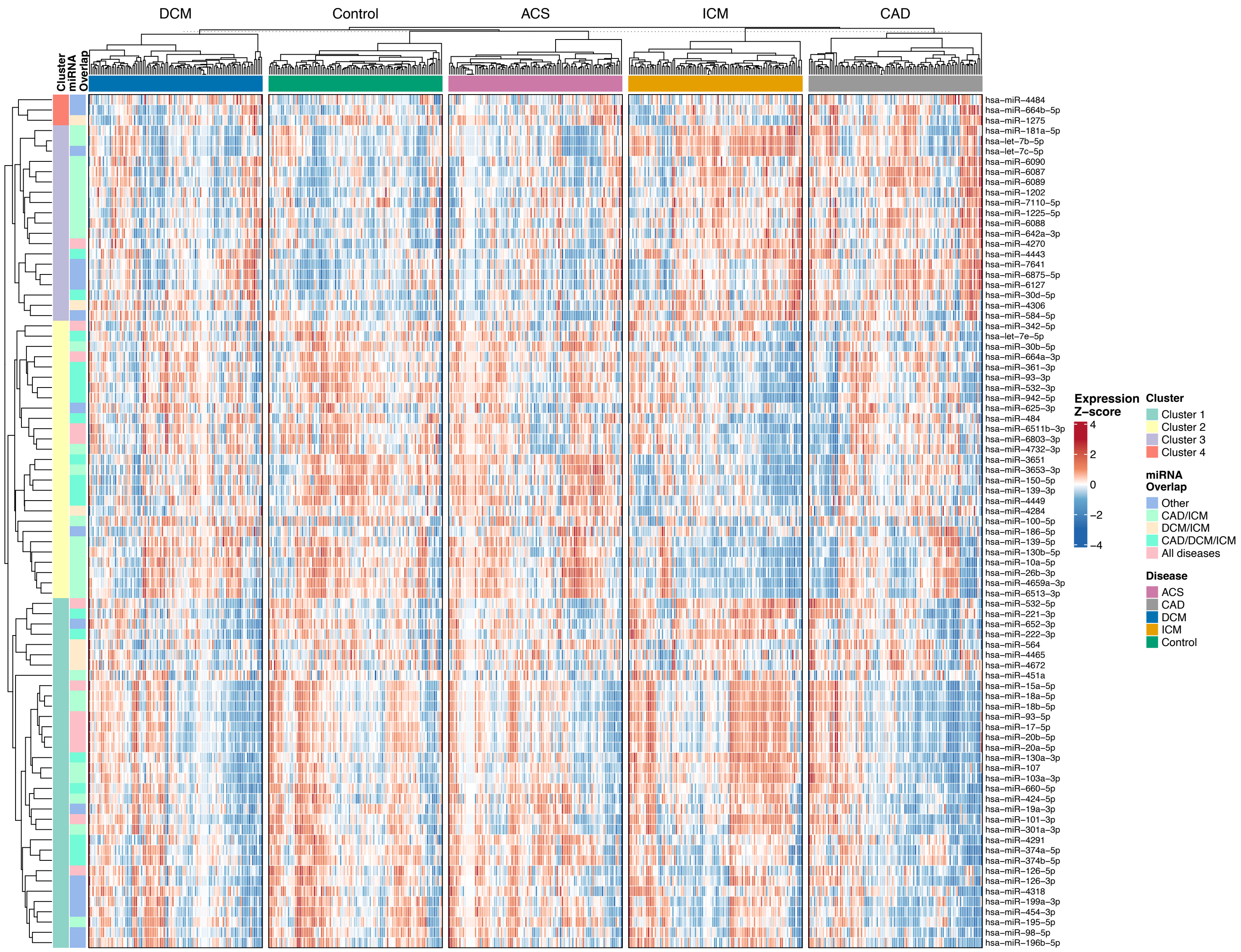

Figure S6

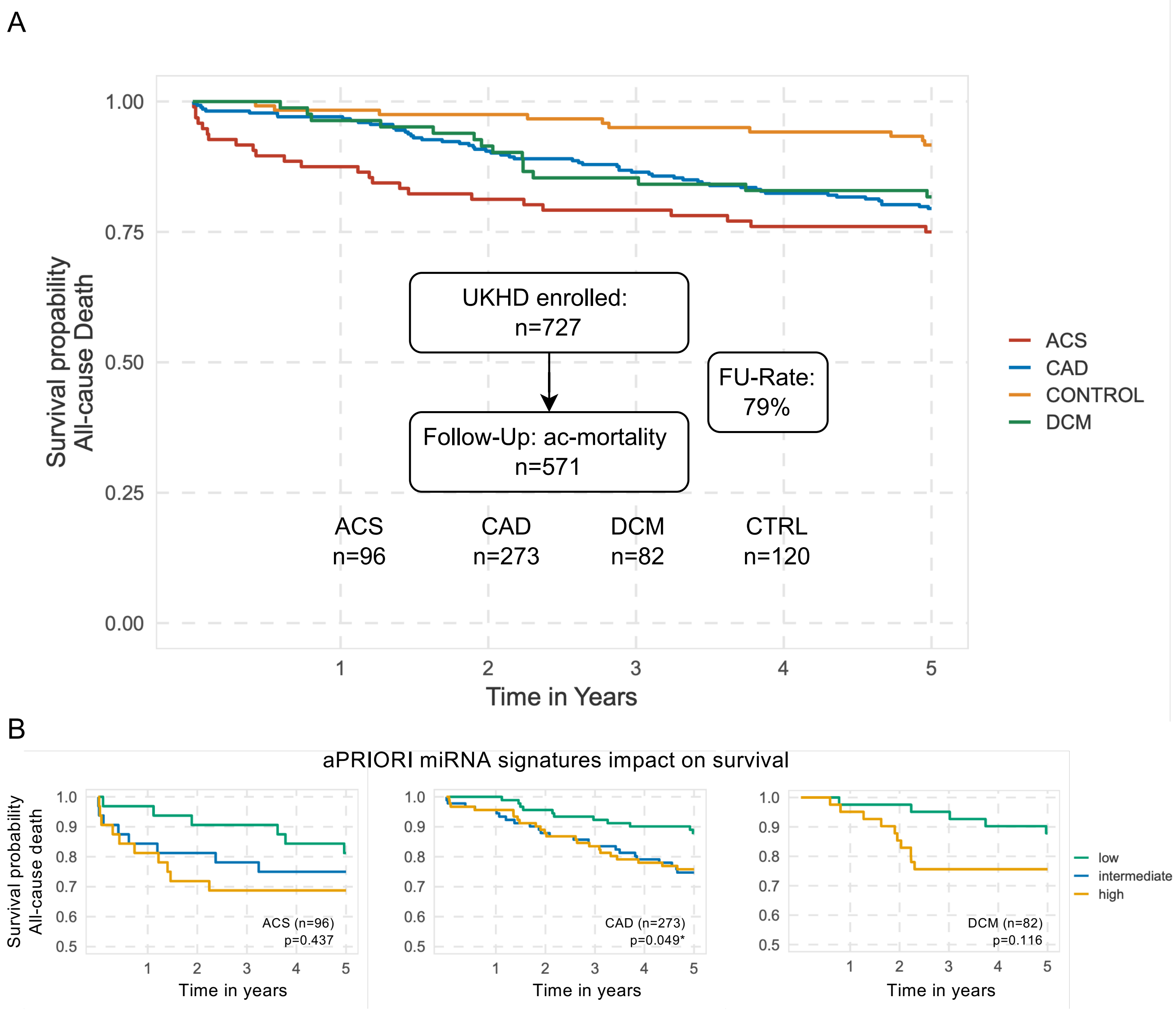
A
1.00
0.75
0.50
0.25
0.00
Survival propability
All-cause Death
UKHD enrolled:
n=727
Follow-Up: ac-mortality
n=571
FU-Rate:
79%
ACS
n=96
CAD
n=273
DCM
n=82
CTRL
n=120
1
2
3
4
5
Time in Years
ACS
CAD
CONTROL
DCM
B
aPRIORI miRNA signatures impact on survival
Survival probability
All-cause death
ACS (n=96)
p=0.437
CAD (n=273)
p=0.049*
DCM (n=82)
p=0.116
Time in years
low
intermediate
high

Figure S7

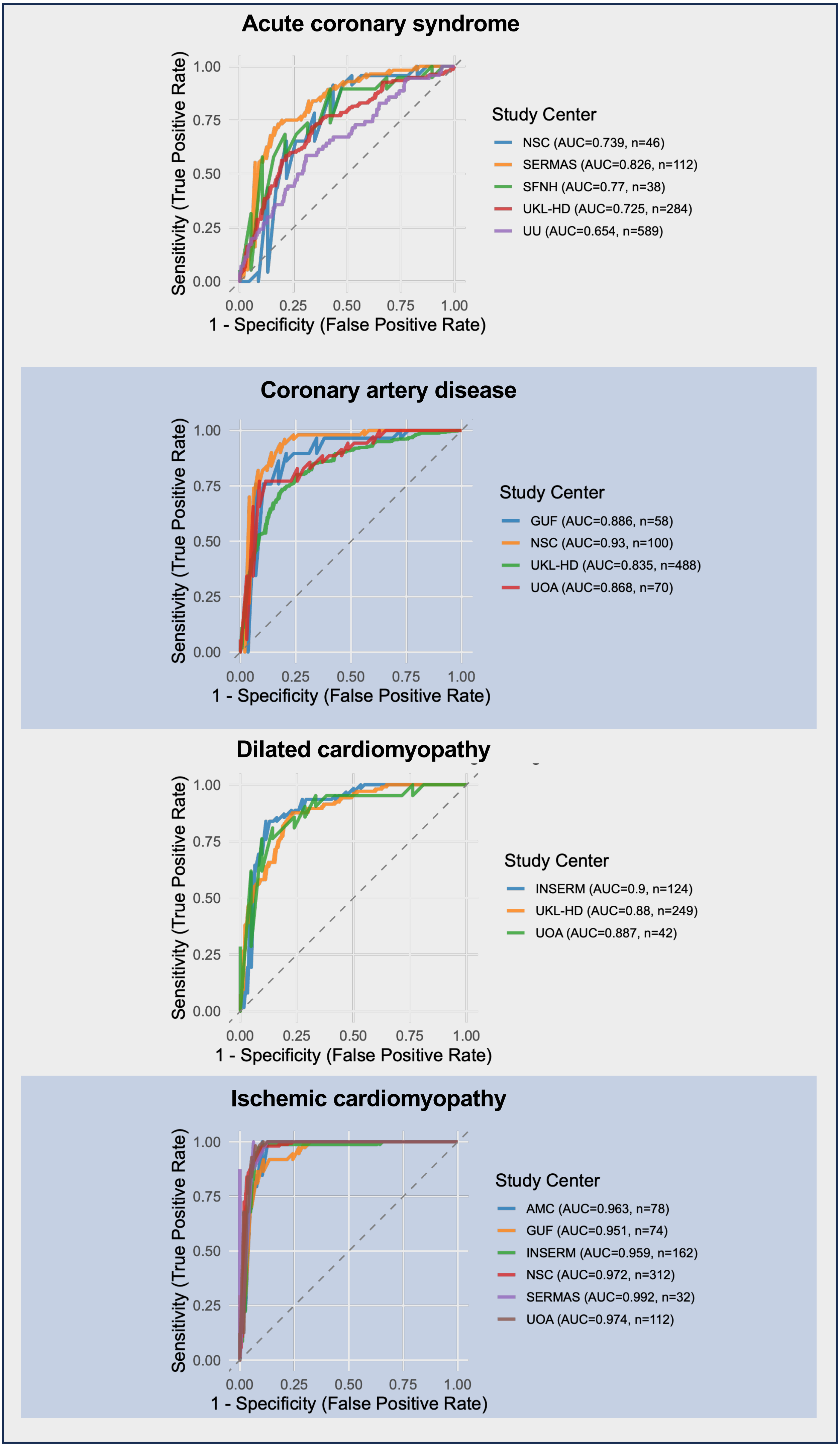

Acute coronary syndrome
Sensitivity (True Positive Rate)
1 - Specificity (False Positive Rate)
0.00
0.25
0.50
0.75
1.00
Study Center
NSC (AUC=0.739, n=46)
SERMAS (AUC=0.826, n=112)
SFNH (AUC=0.77, n=38)
UKL-HD (AUC=0.725, n=284)
UU (AUC=0.654, n=589)
Coronary artery disease
Study Center
GUF (AUC=0.886, n=58)
NSC (AUC=0.93, n=100)
UKL-HD (AUC=0.835, n=488)
UOA (AUC=0.868, n=70)
Dilated cardiomyopathy
Study Center
INSERM (AUC=0.9, n=124)
UKL-HD (AUC=0.88, n=249)
UOA (AUC=0.887, n=42)
Ischemic cardiomyopathy
Study Center
AMC (AUC=0.963, n=78)
GUF (AUC=0.951, n=74)
INSERM (AUC=0.959, n=162)
NSC (AUC=0.972, n=312)
SERMAS (AUC=0.992, n=32)
UOA (AUC=0.974, n=112)

Figure S8

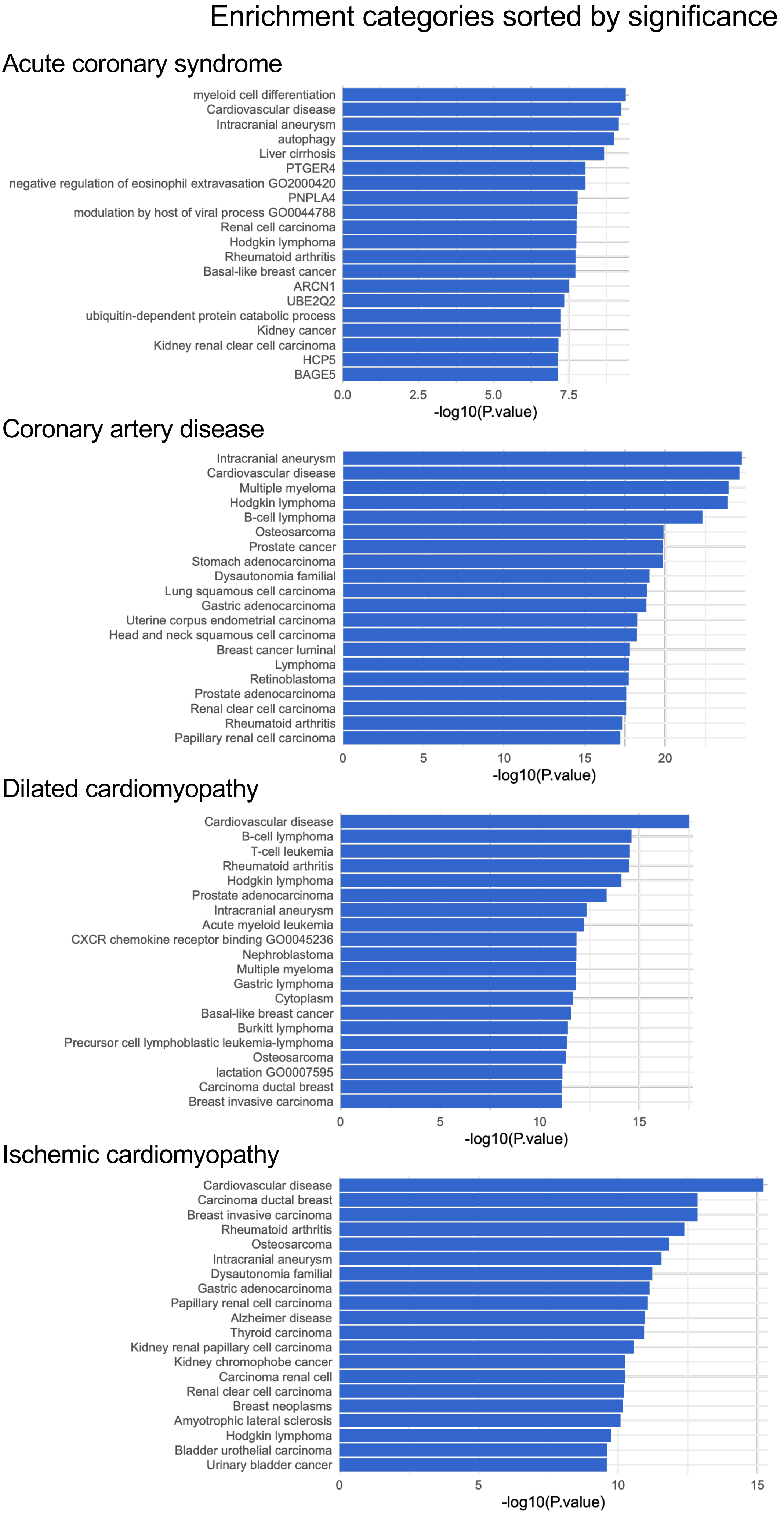

Enrichment categories sorted by significance
Acute coronary syndrome
myeloid cell differentiation
Cardiovascular disease
Intracranial aneurysm
autophagy
Liver cirrhosis
PTGER4
negative regulation of eosinophil extravasation GO2000420
PNPLA4
modulation by host of viral process GO0044788
Renal cell carcinoma
Hodgkin lymphoma
Rheumatoid arthritis
Basal-like breast cancer
ARCN1
UBE2Q2
ubiquitin-dependent protein catabolic process
Kidney cancer
Kidney renal clear cell carcinoma
HCP5
BAGE5
0.0
2.5
5.0
7.5
-log10(P.value)
Coronary artery disease
Intracranial aneurysm
Cardiovascular disease
Multiple myeloma
Hodgkin lymphoma
B-cell lymphoma
Osteosarcoma
Prostate cancer
Stomach adenocarcinoma
Dysautonomia familial
Lung squamous cell carcinoma
Gastric adenocarcinoma
Uterine corpus endometrial carcinoma
Head and neck squamous cell carcinoma
Breast cancer luminal
Lymphoma
Retinoblastoma
Prostate adenocarcinoma
Renal clear cell carcinoma
Rheumatoid arthritis
Papillary renal cell carcinoma
0
5
10
15
20
-log10(P.value)
Dilated cardiomyopathy
Cardiovascular disease
B-cell lymphoma
T-cell leukemia
Rheumatoid arthritis
Hodgkin lymphoma
Prostate adenocarcinoma
Intracranial aneurysm
Acute myeloid leukemia
CXCR chemokine receptor binding GO0045236
Nephroblastoma
Multiple myeloma
Gastric lymphoma
Cytoplasm
Basal-like breast cancer
Burkitt lymphoma
Precursor cell lymphoblastic leukemia-lymphoma
Osteosarcoma
lactation GO0007595
Carcinoma ductal breast
Breast invasive carcinoma
0
5
10
15
-log10(P.value)
Ischemic cardiomyopathy
Cardiovascular disease
Carcinoma ductal breast
Breast invasive carcinoma
Rheumatoid arthritis
Osteosarcoma
Intracranial aneurysm
Dysautonomia familial
Gastric adenocarcinoma
Papillary renal cell carcinoma
Alzheimer disease
Thyroid carcinoma
Kidney renal papillary cell carcinoma
Kidney chromophobe cancer
Carcinoma renal cell
Renal clear cell carcinoma
Breast neoplasms
Amyotrophic lateral sclerosis
Hodgkin lymphoma
Bladder urothelial carcinoma
Urinary bladder cancer
0
5
10
15
-log10(P.value)
