# Supplementary material for: The European BestAgeing Study on microRNA candidates reveals distinct signatures with diagnostic and prognostic potential in cardiovascular disease
Source: BMC Med. 2025 Nov 28;23:670. doi: 10.1186/s12916-025-04502-3 (PMC12670801; doi:10.1186/s12916-025-04502-3)
Supplement: Supplementary file 3 — Additional file 3: Supplementary tables. Table S1 Patient recruitment by center and disease. This table lists the number of patients recruited at each participating centre who underwent miRNA profiling and the specific diseases for which they were recruited. Recruiting centers were Amsterdam UMC- Department of Cardiology, Universitätsklinikum Frankfurt - Department of Cardiology, Frankfurt am Main, Universitätsklinikum Heidelberg - Department of Cardiology, Institut National de la Santé et de la Recherche Médicale (INSERM), National Scientific Center, Institute of Cardiology Named After Academician M.D. Strazhesko of the National Academy of Medical Sciences of Ukraine - Department of Cardiology, Kyiv, Servicio Madrileño de Salud - Department of Cardiology, Madrid, Spain, Azienda Ospedaliera San Filippo Neri - Department of Cardiology, Rome, Italy, Azienda Ospedaliera di Padova (Università degli Studi di Padova) - Department of Cardiology, Padua, Italy, Ethniko kai Kapodistriako Panepistimio Athinon (National and Kapodistrian University of Athens) - Department of Cardiology, Athens, Greece, Uppsala Universitetssjukhus (Uppsala Universitet) - Department of Cardiology, Uppsala, Sweden. Table S2 Summary of abstract extraction and miRNA Identification. The table provides a detailed breakdown of the total number of PubMed abstracts loaded, the count of abstracts successfully extracted using the miRetrieve package, and the subsequent identification of unique miRNAs. Table S3 Intersections of miRNAs in Distinct Cardiovascular Phenotypes. This table details the shared top 50 literature miRNAs per disease as indicated by the weighted biomarker score retrieved via miRetrieve across different cardiovascular conditions. This summary provides insights into the common miRNAs observed in various cardiovascular diseases. Table S4 Selected top 50 miRNAs identified from the literature search and grouped by disease of interest. The weighted biomarker score was calculated using the miRe [file 12916_2025_4502_MOESM3_ESM.docx]

**Additional file 3**

**Supplementary Tables**

**Supplementary Tables**

**Study design and patient characteristics**

**Table 1: Patient recruitment by center and disease.** This table lists the number of patients recruited at each participating centre who underwent miRNA profiling and the specific diseases for which they were recruited. Recruiting centers were Amsterdam UMC- Department of Cardiology, Universitätsklinikum Frankfurt - Department of Cardiology, Frankfurt am Main, Universitätsklinikum Heidelberg - Department of Cardiology, Institut National de la Santé et de la Recherche Médicale (INSERM), National Scientific Center, Institute of Cardiology Named After Academician M.D. Strazhesko of the National Academy of Medical Sciences of Ukraine - Department of Cardiology, Kyiv, Servicio Madrileño de Salud - Department of Cardiology, Madrid, Spain, Azienda Ospedaliera San Filippo Neri - Department of Cardiology, Rome, Italy, Azienda Ospedaliera di Padova (Università degli Studi di Padova) - Department of Cardiology, Padua, Italy, Ethniko kai Kapodistriako Panepistimio Athinon (National and Kapodistrian University of Athens) - Department of Cardiology, Athens, Greece, Uppsala Universitetssjukhus (Uppsala Universitet) - Department of Cardiology, Uppsala, Sweden

| **CENTER** | **CONTROL** | **ACS** | **CAD** | **DCM** | **ICM** | **SUM** |
| --- | --- | --- | --- | --- | --- | --- |
| *ACADEMISCH MEDISCH CENTRUM BIJ DE UNIVERSITEIT VAN AMSTERDAM* |  |  |  |  | 39 | 39 |
| *AZIENDA COMPLESSO OSPEDALIERO SAN FILIPPO NERI* |  | 19 | 1 |  |  | 20 |
| *INSTITUT NATIONAL DE LA SANTE ET DE LA RECHERCHE MEDICALE* |  |  |  | 62 | 19 | 81 |
| *JOHANN WOLFGANG GOETHE UNIVERSITAET FRANKFURT AM MAIN* |  | 1 | 7 | 11 | 26 | 45 |
| *NATIONAL AND KAPODISTRIAN UNIVERSITY OF ATHENS* |  |  |  | 21 | 35 | 56 |
| *NATIONAL SCIENTIFIC CENTER INSTITUTE OF CARDIOLOGY N.A. M.D.STRAZHESKO* |  | 23 | 50 |  | 156 | 229 |
| *SERVICIO MADRILENO DE SALUD* |  | 56 | 3 | 2 | 16 | 77 |
| *UNIVERSITA CATTOLICA DEL SACRO CUORE* |  |  | 9 |  | 5 | 14 |
| *UNIVERSITA DEGLI STUDI DI PADOVA* | 180 |  |  |  |  | 180 |
| *UNIVERSITAETSKLINIKUM HEIDELBERG* | 149 | 135 | 338 | 105 |  | 727 |
| *UPPSALA UNIVERSITET* | 519 | 70 |  |  |  | 589 |
| *SUM* | 848 | 304 | 408 | 201 | 296 | 2057 |

Site Profiling: Heidelberg 2057 samples

**Text mining and literature miRNAs**

**Table 2: Summary of abstract extraction and miRNA Identification.** The table provides a detailed breakdown of the total number of abstracts loaded, the count of abstracts successfully extracted using the miRetrieve package, and the subsequent identification of unique miRNAs.

|  | **ACS** | **CAD** | **DCM** | **ICM** |
| --- | --- | --- | --- | --- |
| **Literature Search Results** | 250 | 186 | 29 | 169 |
| **Subset miRetrieve** | 185 | 111 | 19 | 56 |
| **Unique miRNAs mentioned** | 166 | 181 | 56 | 182 |

**Table 3: Intersections of miRNAs in Distinct Cardiovascular Phenotypes.** This table details the shared literature miRNAs. This summary provides insights into the common miRNAs observed in various cardiovascular diseases.

| **Intersection** | **miRNAs** | **Number of miRNAs** | |
| --- | --- | --- | --- |
| ICM | hsa-mir-1306-3p, hsa-mir-622, hsa-mir-652-3p, hsa-mir-665, hsa-mir-1285-3p, hsa-mir-4491, hsa-mir-216a-5p, hsa-mir-217, hsa-mir-654-5p, hsa-mir-939-5p, hsa-mir-1908-5p, hsa-mir-3615, hsa-mir-7706, hsa-mir-129-5p, hsa-mir-200a-3p, hsa-mir-299-3p, hsa-mir-371a-3p, hsa-mir-18b-5p, hsa-mir-518e-3p, hsa-mir-568, hsa-mir-583, hsa-mir-595, hsa-mir-650, hsa-mir-662, hsa-mir-1228-3p, hsa-mir-1296-5p, hsa-mir-1292-5p, hsa-mir-1825, hsa-mir-3148, hsa-mir-3155a, hsa-mir-3175, hsa-mir-224-5p, hsa-mir-30b-5p, hsa-mir-339-5p, hsa-mir-452-5p, hsa-mir-638, hsa-mir-675-5p, hsa-mir-211-5p, hsa-mir-128-3p, hsa-mir-301a-3p, hsa-mir-671-5p, hsa-mir-550a-5p, hsa-mir-1233-3p, hsa-mir-107, hsa-mir-29c-3p, hsa-mir-302b-3p, hsa-mir-20b-5p, hsa-mir-940, hsa-mir-302f, hsa-mir-320e, hsa-mir-8485 | | 51 |
| ACS:CAD:ICM | hsa-mir-499a-5p, hsa-mir-208b-3p, hsa-mir-208a-3p, hsa-mir-22-3p, hsa-mir-132-3p, hsa-mir-30d-5p, hsa-mir-134-5p, hsa-mir-484, hsa-mir-19b-3p, hsa-mir-27a-3p, hsa-mir-106b-5p, hsa-mir-340-3p, hsa-mir-340-5p, hsa-mir-16-5p, hsa-mir-199a-5p, hsa-mir-34a-5p, hsa-mir-24-3p, hsa-mir-92a-3p, hsa-mir-221-3p, hsa-mir-502-5p, hsa-mir-125a-5p, hsa-mir-29b-3p, hsa-mir-191-5p, hsa-mir-195-5p, hsa-mir-328-3p, hsa-mir-17-5p, hsa-mir-181a-5p, hsa-mir-222-3p, hsa-mir-214-3p, hsa-mir-130a-3p, hsa-mir-483-3p, hsa-mir-23a-3p, hsa-mir-140-5p, hsa-mir-30e-5p, hsa-mir-23b-3p, hsa-mir-103a-3p, hsa-mir-425-3p, hsa-mir-425-5p, hsa-mir-424-5p, hsa-mir-101-3p, hsa-mir-744-5p, hsa-mir-342-3p, hsa-mir-138-5p, hsa-mir-370-3p, hsa-mir-181b-5p | | 45 |
| CAD | hsa-mir-942-5p, hsa-mir-15b-5p, hsa-mir-98-5p, hsa-mir-628-3p, hsa-mir-765, hsa-mir-200b-3p, hsa-mir-135b-5p, hsa-mir-196b-5p, hsa-mir-574-3p, hsa-mir-503-5p, hsa-mir-337-3p, hsa-mir-433-3p, hsa-mir-584-5p, hsa-mir-378a-5p, hsa-mir-378a-3p, hsa-mir-141-3p, hsa-mir-3163, hsa-mir-6090, hsa-mir-382-5p, hsa-mir-326, hsa-mir-432-5p, hsa-mir-615-3p, hsa-mir-624-5p, hsa-mir-624-3p, hsa-mir-3149, hsa-mir-3152-3p, hsa-mir-200c-3p, hsa-mir-548a-3p, hsa-mir-148a-3p, hsa-mir-362-5p, hsa-mir-199b-5p, hsa-mir-212-3p, hsa-mir-548d-3p, hsa-mir-3117-3p, hsa-mir-5683, hsa-mir-5701, hsa-mir-198, hsa-mir-147a | | 38 |
| ACS:CAD | hsa-mir-186-5p, hsa-mir-32-5p, hsa-mir-215-5p, hsa-mir-487a-3p, hsa-mir-152-3p, hsa-mir-507, hsa-mir-656-3p, hsa-mir-1915-3p, hsa-mir-4516, hsa-mir-204-5p, hsa-mir-363-3p, hsa-mir-941, hsa-mir-99a-5p, hsa-mir-139-5p, hsa-mir-15a-5p, hsa-mir-296-5p, hsa-mir-629-3p, hsa-mir-629-5p, hsa-mir-889-3p, hsa-mir-3646, hsa-mir-33a-5p, hsa-mir-188-5p, hsa-mir-148b-3p, hsa-mir-587, hsa-mir-874-3p | | 25 |
| ACS:CAD:DCM:ICM | hsa-mir-1-3p, hsa-mir-133a-3p, hsa-mir-21-5p, hsa-mir-122-5p, hsa-mir-126-3p, hsa-mir-223-3p, hsa-mir-150-5p, hsa-mir-146a-5p, hsa-mir-155-5p, hsa-mir-133b, hsa-mir-423-3p, hsa-mir-26a-5p, hsa-mir-20a-5p, hsa-mir-145-5p, hsa-mir-486-5p, hsa-mir-29a-3p, hsa-mir-143-3p, hsa-mir-210-3p, hsa-mir-451a, hsa-mir-197-3p, hsa-mir-124-3p, hsa-mir-182-5p, hsa-mir-144-3p, hsa-mir-320a | | 24 |
| ACS | hsa-mir-519e-3p, hsa-mir-137, hsa-mir-203a-3p, hsa-mir-492, hsa-mir-193a-3p, hsa-mir-1291, hsa-mir-345-5p, hsa-mir-3681-5p, hsa-mir-4478, hsa-mir-28-5p, hsa-mir-127-3p, hsa-mir-380-3p, hsa-mir-580-3p, hsa-mir-649, hsa-mir-663a, hsa-mir-330-3p, hsa-mir-582-5p, hsa-mir-3162-5p, hsa-mir-1303, hsa-mir-4329 | | 20 |
| CAD:ICM | hsa-mir-149-5p, hsa-mir-205-5p, hsa-mir-18a-5p, hsa-mir-10a-5p, hsa-mir-320b, hsa-mir-31-5p, hsa-mir-190a-5p, hsa-mir-25-3p, hsa-mir-495-3p, hsa-mir-545-3p, hsa-mir-10b-5p, hsa-mir-194-5p, hsa-mir-135a-5p, hsa-mir-9-5p | | 14 |
| ACS:ICM | hsa-mir-497-5p, hsa-mir-663b, hsa-mir-193b-3p, hsa-mir-125b-5p, hsa-mir-26b-5p, hsa-mir-375, hsa-mir-1254, hsa-mir-181c-5p, hsa-mir-660-5p, hsa-mir-1202, hsa-mir-494-3p, hsa-mir-590-5p | | 12 |
| ACS:DCM:ICM | hsa-mir-361-5p, hsa-mir-30a-5p, hsa-mir-183-5p, hsa-let-7a-5p, hsa-mir-3135b, hsa-mir-30c-5p | | 6 |
| CAD:DCM:ICM | hsa-mir-192-5p, hsa-mir-1246, hsa-mir-206, hsa-mir-454-3p, hsa-mir-185-5p | | 5 |
| DCM | hsa-mir-146b-5p, hsa-mir-379-5p, hsa-mir-636, hsa-mir-639, hsa-mir-646 | | 5 |
| DCM:ICM | hsa-mir-92b-3p, hsa-mir-27b-3p, hsa-mir-3908, hsa-mir-500a-3p, hsa-mir-500a-5p | | 5 |
| ACS:CAD:DCM | hsa-mir-19a-3p, hsa-let-7f-5p, hsa-mir-93-5p | | 3 |
| CAD:DCM | hsa-mir-130b-3p | | 1 |

**Table 4: Selected top 50 miRNAs identified from the literature search and grouped by disease of interest.** The weighted biomarker score was calculated using the miRetrieve package, taking into account the number of associated PMIDs and biomarker keywords. Univariate AUCs were calculated for each miRNA and miRNAs ranked accordingly. AUROC; area under the receiver operating characteristic curve, PMID; PubMed unique identifier.

|  |  | Performance | |  |
| --- | --- | --- | --- | --- |
| microRNA | Accession | Biomarker Score | AUROC | PMID |
| Acute Coronary Syndrome | | | | |
| hsa-miR-15a-5p | MIMAT0000068 | 5 | 0.619 (0.568; 0.670) | 29762500, 22052914, 27665403 |
| hsa-miR-20a-5p | MIMAT0000075 | 15 | 0.615 (0.564; 0.665) | 31885737, 30090932 |
| hsa-miR-126-3p | MIMAT0000445 | 33 | 0.609 (0.558; 0.660) | 32595526, 24900964, 28437675, 29030746, 30356307, 27866054, 36416963, 35228564, 34098766, 33580779, 28666417, 26580972, 22011751, 21969012 |
| hsa-miR-451a | MIMAT0001631 | 16 | 0.608 (0.556; 0.660) | 36214846, 28624816 |
| hsa-miR-30d-5p | MIMAT0000245 | 22 | 0.601 (0.550; 0.652) | 31130720, 31092195, 27176713 |
| hsa-miR-140-5p | MIMAT0000431 | 10 | 0.595 (0.543; 0.646) | 27357355 |
| hsa-let-7f-5p | MIMAT0000067 | 16 | 0.586 (0.534; 0.637) | 33866193, 31401194, 24046434 |
| hsa-miR-16-5p | MIMAT0000069 | 19 | 0.585 (0.534; 0.636) | 36198129, 29350392, 27665403, 25350775, 24046434, 23967079 |
| hsa-miR-186-5p | MIMAT0000456 | 23 | 0.584 (0.532; 0.635) | 36139082, 27357355, 26939053, 24727883, 23641832 |
| hsa-miR-23a-3p | MIMAT0000078 | 10 | 0.582 (0.531; 0.634) | 31130720, 31039814, 28213360 |
| hsa-miR-223-3p | MIMAT0000280 | 32 | 0.580 (0.528; 0.632) | 36214592, 36139082, 34098766, 33866193, 32356454, 30623425, 29030746, 28666417, 26720041, 25350775, 24573468, 24345063, 23641832, 21969012 |
| hsa-miR-130a-3p | MIMAT0000425 | 11 | 0.577 (0.525; 0.629) | 36214592, 32356454 |
| hsa-miR-125a-5p | MIMAT0000443 | 16 | 0.575 (0.523; 0.626) | 35751950, 26485305, 24046434 |
| hsa-miR-145-5p | MIMAT0000437 | 22 | 0.569 (0.518; 0.621) | 36723013, 36416963, 33725857, 33261889, 31039814, 27357636, 25465803, 20886220 |
| hsa-miR-210-3p | MIMAT0000267 | 16 | 0.568 (0.516; 0.619) | 33783502, 27357355, 27346801 |
| hsa-miR-222-3p | MIMAT0000279 | 15 | 0.567 (0.515; 0.619) | 36416963, 34330158 |
| hsa-miR-221-3p | MIMAT0000278 | 18 | 0.566 (0.515; 0.618) | 34330158, 34208452, 27374153 |
| hsa-miR-21-5p | MIMAT0000076 | 44 | 0.564 (0.512; 0.615) | 34209965, 36214846, 33580779, 32735867, 32214121, 32033332, 31817254, 31782762, 31721935, 29506703, 29188800, 29030746, 28663047, 27785570, 26875904, 26580972, 26337652, 26248417, 25184815, 23023917, 22330002 |
| hsa-miR-155-5p | MIMAT0000646 | 27 | 0.563 (0.512; 0.615) | 35228564, 35018773, 30623425, 30481637, 28624816, 27866054, 24525789, 22995291, 21969012 |
| hsa-miR-133b | MIMAT0000770 | 27 | 0.554 (0.501; 0.606) | 37165064, 32033332, 31782762, 31672851, 29350392, 27593229, 27484208, 27357636, 25111390, 21806992 |
| hsa-miR-4516 | MIMAT0019053 | 16 | 0.553 (0.501; 0.605) | 37203392 |
| hsa-miR-340-3p | MIMAT0000750 | 22 | 0.547 (0.495; 0.599) | 31885737 |
| hsa-miR-361-5p | MIMAT0000703 | 26 | 0.546 (0.494; 0.598) | 33546604, 25184815 |
| hsa-miR-19a-3p | MIMAT0000073 | 19 | 0.544 (0.492; 0.596) | 32334458, 27357355, 25383678 |
| hsa-miR-363-3p | MIMAT0000707 | 13 | 0.537 (0.485; 0.589) | 28830367 |
| hsa-miR-502-5p | MIMAT0002873 | 18 | 0.537 (0.485; 0.589) | 31782762 |
| hsa-miR-106b-5p | MIMAT0000680 | 21 | 0.536 (0.484; 0.588) | 33670982, 32356454, 30090932 |
| hsa-miR-195-5p | MIMAT0000461 | 16 | 0.535 (0.483; 0.587) | 23236408 |
| hsa-miR-197-3p | MIMAT0000227 | 17 | 0.533 (0.481; 0.585) | 34208452, 26720041 |
| hsa-miR-1915-3p | MIMAT0007892 | 18 | 0.533 (0.481; 0.585) | 31675148, 23255549 |
| hsa-miR-26a-5p | MIMAT0000082 | 25 | 0.532 (0.480; 0.584) | 32735867, 32214121, 31092195, 27192016, 24900964, 24046434 |
| hsa-miR-183-5p | MIMAT0000261 | 16 | 0.531 (0.479; 0.583) | 36723013, 29762500 |
| hsa-miR-486-5p | MIMAT0002177 | 19 | 0.531 (0.479; 0.583) | 36214846, 27190129, 26485305, 26077801, 24900964 |
| hsa-miR-152-3p | MIMAT0000438 | 16 | 0.529 (0.477; 0.582) | 36723013, 35751950 |
| hsa-miR-30e-5p | MIMAT0000692 | 10 | 0.528 (0.476; 0.580) | 35228564 |
| hsa-miR-146a-5p | MIMAT0000449 | 32 | 0.528 (0.476; 0.580) | 34209965, 34786024, 33866193, 33261889, 32356454, 31130720, 31092195, 27665403, 26485305, 26337652, 23023917 |
| hsa-miR-139-5p | MIMAT0000250 | 11 | 0.528 (0.476; 0.580) | 34144190 |
| hsa-miR-215-5p | MIMAT0000272 | 18 | 0.526 (0.474; 0.578) | 31782762 |
| hsa-miR-125b-5p | MIMAT0000423 | 12 | 0.524 (0.472; 0.576) | 27176713 |
| hsa-miR-26b-5p | MIMAT0000083 | 11 | 0.523 (0.471; 0.575) | 26046358 |
| hsa-miR-199a-5p | MIMAT0000231 | 20 | 0.521 (0.426; 0.531) | 31092195 |
| hsa-miR-182-5p | MIMAT0000259 | 13 | 0.520 (0.468; 0.572) | 28830367 |
| hsa-miR-340-5p | MIMAT0004692 | 22 | 0.519 (0.467; 0.572) | 31885737 |
| hsa-miR-150-5p | MIMAT0000451 | 32 | 0.513 (0.461; 0.565) | 31933487, 31817254, 31179325, 30745540, 29030746, 27357355, 26077801, 24900964, 24727883, 23967079 |
| hsa-miR-24-3p | MIMAT0000080 | 18 | 0.509 (0.457; 0.561) | 31039814, 30356307, 26561061 |
| hsa-miR-181a-5p | MIMAT0000256 | 15 | 0.506 (0.454; 0.559) | 27997916 |
| hsa-miR-484 | MIMAT0002174 | 22 | 0.506 (0.453; 0.558) | 36214592, 32356454, 32214121 |
| hsa-miR-99a-5p | MIMAT0000097 | 11 | 0.505 (0.453; 0.557) | 28051249 |
| hsa-miR-27a-3p | MIMAT0000084 | 21 | 0.504 (0.452; 0.556) | 31092195, 23967079 |
| hsa-miR-328-3p | MIMAT0000752 | 17 | 0.502 (0.450; 0.555) | 24833470, 21881276 |
| Coronary Artery Disease | | | | |
| hsa-miR-19a-3p | MIMAT0000073 | 32 | 0.757 (0.726; 0.788) | 21785714, 31495986, 27357355, 37376990, 33352533 |
| hsa-miR-15a-5p | MIMAT0000068 | 16 | 0.716 (0.683; 0.749) | 32776741, 35285386, 32245321, 27665403, 26233505 |
| hsa-miR-424-5p | MIMAT0001341 | 20 | 0.699 (0.665; 0.733) | 33557763, 26017792 |
| hsa-miR-18a-5p | MIMAT0000072 | 11 | 0.697 (0.663; 0.731) | 34689283, 32245321, 25415674 |
| hsa-miR-17-5p | MIMAT0000070 | 18 | 0.692 (0.657; 0.726) | 34827683, 32356454, 32245321, 29979444, 25415674, 25349183, 20595655 |
| hsa-miR-126-3p | MIMAT0000445 | 38 | 0.687 (0.653; 0.721) | 36538237, 22813605, 36214592, 35751015, 33674633, 32404537, 32245321, 31788836, 31495986, 29891858, 28751542, 28666417, 28169059, 27497911, 27005938, 26720041, 26198441, 25349183, 25327597, 24260372, 23316282, 22011751, 21969012, 20595655 |
| hsa-miR-144-3p | MIMAT0000436 | 31 | 0.685 (0.650; 0.719) | 30143484, 33381550, 24733347 |
| hsa-miR-195-5p | MIMAT0000461 | 13 | 0.680 (0.646; 0.714) | 35285386, 26221607 |
| hsa-miR-21-5p | MIMAT0000076 | 41 | 0.676 (0.642; 0.711) | 35751015, 34827683, 33674633, 32772195, 32245321, 32033332, 31797949, 31788836, 31782762, 31109008, 30126654, 29891858, 29777114, 29506703, 29188800, 28751542, 27976798, 27629254, 27005938, 26304936, 26221607, 26198441, 25349183, 23316282 |
| hsa-miR-130a-3p | MIMAT0000425 | 14 | 0.667 (0.632; 0.702) | 36214592, 32356454, 32332683, 31495986, 29891858 |
| hsa-miR-16-5p | MIMAT0000069 | 21 | 0.667 (0.632; 0.702) | 35285386, 32245321, 31813253, 27665403, 26023181, 25385173, 25350775, 21909417 |
| hsa-miR-26a-5p | MIMAT0000082 | 11 | 0.666 (0.631; 0.701) | 32332683, 27005938, 26233505, 26221607 |
| hsa-miR-215-5p | MIMAT0000272 | 19 | 0.660 (0.626; 0.695) | 31782762, 25198728 |
| hsa-miR-132-3p | MIMAT0000426 | 20 | 0.659 (0.625; 0.694) | 33557763, 27357355 |
| hsa-miR-24-3p | MIMAT0000080 | 23 | 0.657 (0.622; 0.692) | 33557763, 27629254, 26304936, 26233505, 26221607 |
| hsa-miR-502-5p | MIMAT0002873 | 19 | 0.656 (0.621; 0.691) | 31782762, 25198728 |
| hsa-miR-155-5p | MIMAT0000646 | 31 | 0.649 (0.614; 0.684) | 35751015, 32245321, 31788836, 28867189, 28624816, 28205634, 27653298, 26233505, 26221607, 24525789, 23316282, 21969012, 20595655 |
| hsa-miR-942-5p | MIMAT0004985 | 31 | 0.648 (0.613; 0.683) | 33381550 |
| hsa-miR-196b-5p | MIMAT0001080 | 15 | 0.646 (0.611; 0.681) | 35751015 |
| hsa-miR-192-5p | MIMAT0000222 | 31 | 0.642 (0.607; 0.677) | 33381550 |
| hsa-miR-182-5p | MIMAT0000259 | 16 | 0.641 (0.606; 0.676) | 35751015, 28830367 |
| hsa-miR-222-3p | MIMAT0000279 | 16 | 0.636 (0.601; 0.672) | 34330158, 29737876, 28751542, 27005938, 25349183 |
| hsa-let-7f-5p | MIMAT0000067 | 31 | 0.630 (0.595; 0.666) | 35562921, 31495986 |
| hsa-miR-30d-5p | MIMAT0000245 | 18 | 0.629 (0.594; 0.665) | 25999658 |
| hsa-miR-628-3p | MIMAT0003297 | 19 | 0.627 (0.591; 0.663) | 27653298 |
| hsa-miR-1246 | MIMAT0005898 | 18 | 0.616 (0.579; 0.652) | 25999658 |
| hsa-miR-15b-5p | MIMAT0000417 | 20 | 0.607 (0.571; 0.643) | 35285386, 33557763 |
| hsa-miR-145-5p | MIMAT0000437 | 25 | 0.606 (0.569; 0.642) | 35751015, 33261889, 33015157, 31788836, 28205634, 27660218, 27357636, 26221607, 25385173, 24260372, 20595655 |
| hsa-miR-10a-5p | MIMAT0000253 | 12 | 0.604 (0.568; 0.640) | 32404537 |
| hsa-miR-221-3p | MIMAT0000278 | 14 | 0.592 (0.555; 0.629) | 34330158, 31788836, 29737876 |
| hsa-miR-133b | MIMAT0000770 | 23 | 0.592 (0.555; 0.628) | 37165064, 32033332, 31782762, 27484208, 27357636, 24260372 |
| hsa-miR-296-5p | MIMAT0000690 | 11 | 0.590 (0.554; 0.627) | 36852944, 31797949, 31495986 |
| hsa-miR-23b-3p | MIMAT0000418 | 14 | 0.590 (0.554; 0.627) | 33015157 |
| hsa-miR-1915-3p | MIMAT0007892 | 17 | 0.587 (0.550; 0.623) | 31675148 |
| hsa-miR-484 | MIMAT0002174 | 11 | 0.578 (0.541; 0.614) | 36214592, 32356454 |
| hsa-miR-125a-5p | MIMAT0000443 | 33 | 0.576 (0.539; 0.612) | 33381550, 26485305, 26233505 |
| hsa-miR-149-5p | MIMAT0000450 | 34 | 0.573 (0.536; 0.610) | 33381550, 32332683, 26017792, 25664324 |
| hsa-miR-30e-5p | MIMAT0000692 | 17 | 0.568 (0.532; 0.605) | 32772195 |
| hsa-miR-146a-5p | MIMAT0000449 | 21 | 0.566 (0.529; 0.602) | 34209965, 35751015, 33261889, 32356454, 27665403, 26485305, 26233505, 23316282 |
| hsa-miR-574-3p | MIMAT0003239 | 13 | 0.553 (0.517; 0.590) | 31709737, 29091861, 26685009 |
| hsa-miR-320b | MIMAT0005792 | 12 | 0.546 (0.509; 0.583) | 35285386 |
| hsa-miR-92a-3p | MIMAT0000092 | 33 | 0.545 (0.508; 0.582) | 35285386, 32772195, 32404537, 31495986, 29777114, 28867189, 28751542, 28213360, 28205634, 27981487, 27005938, 26485305, 26198441, 25415674, 25349183, 21969012, 20595655 |
| hsa-miR-223-3p | MIMAT0000280 | 27 | 0.544 (0.507; 0.581) | 36214592, 35751015, 32356454, 32245321, 29888618, 28751542, 28666417, 26720041, 26233505, 25350775, 23316282, 21969012 |
| hsa-miR-320a | MIMAT0000510 | 12 | 0.538 (0.501; 0.575) | 35285386 |
| hsa-miR-210-3p | MIMAT0000267 | 15 | 0.521 (0.483; 0.559) | 32404537, 31495986, 27357355, 26198441 |
| hsa-miR-29a-3p | MIMAT0000086 | 16 | 0.520 (0.483; 0.557) | 33674633, 31709737, 27629254 |
| hsa-miR-197-3p | MIMAT0000227 | 16 | 0.515 (0.478; 0.552) | 26720041 |
| hsa-miR-186-5p | MIMAT0000456 | 31 | 0.514 (0.477; 0.551) | 35562921, 27357355 |
| hsa-miR-22-3p | MIMAT0000077 | 15 | 0.508 (0.455; 0.529) | 27484208, 26304936 |
| hsa-miR-584-5p | MIMAT0003249 | 12 | 0.504 (0.467; 0.541) | 31863331 |
| Dilated Cardiomyopathy | | | | |
| hsa-miR-126-3p | MIMAT0000445 | 21 | 0.669 (0.616; 0.722) | 37138538, 36187500, 34797300, 35726831, 26313139, 30054123, 29314582, 29335596, 23438607, 22041329 |
| hsa-miR-93-5p | MIMAT0000093 | 9 | 0.668 (0.615; 0.721) | 33215881 |
| hsa-miR-150-5p | MIMAT0000451 | 10 | 0.658 (0.605; 0.711) | 33824379, 36187500, 33443726, 28259597 |
| hsa-let-7a-5p | MIMAT0000062 | 8 | 0.632 (0.577; 0.686) | 30008018 |
| hsa-let-7f-5p | MIMAT0000067 | 9 | 0.627 (0.572; 0.682) | 33215881 |
| hsa-miR-185-5p | MIMAT0000455 | 3 | 0.625 (0.570; 0.680) | 27645404, 34973276, 34685557 |
| hsa-miR-20a-5p | MIMAT0000075 | 7 | 0.623 (0.568; 0.678) | 34187571 |
| hsa-miR-144-3p | MIMAT0000436 | 6 | 0.622 (0.567; 0.677) | 32598816 |
| hsa-miR-155-5p | MIMAT0000646 | 21 | 0.622 (0.567; 0.677) | 25840506, 36291684, 33830643, 22041329 |
| hsa-miR-30a-5p | MIMAT0000087 | 9 | 0.617 (0.562; 0.673) | 33215881, 35690861, 32124924, 29314582, 23660476 |
| hsa-miR-3135b | MIMAT0018985 | 13 | 0.617 (0.562; 0.672) | 27748501 |
| hsa-miR-454-3p | MIMAT0003885 | 10 | 0.609 (0.554; 0.665) | 30008018, 23247724 |
| hsa-miR-92b-3p | MIMAT0003218 | 17 | 0.592 (0.536; 0.648) | 29719295 |
| hsa-miR-145-5p | MIMAT0000437 | 8 | 0.590 (0.534; 0.645) | 30008018 |
| hsa-miR-183-5p | MIMAT0000261 | 10 | 0.586 (0.529; 0.642) | 32598816, 32102357 |
| hsa-miR-197-3p | MIMAT0000227 | 9 | 0.579 (0.523; 0.635) | 33215881 |
| hsa-miR-320a | MIMAT0000510 | 17 | 0.576 (0.520; 0.632) | 29719295 |
| hsa-miR-486-5p | MIMAT0002177 | 6 | 0.571 (0.515; 0.627) | 32598816 |
| hsa-miR-500a-5p | MIMAT0004773 | 9 | 0.566 (0.510; 0.622) | 23247724 |
| hsa-miR-19a-3p | MIMAT0000073 | 7 | 0.565 (0.509; 0.621) | 34187571 |
| hsa-miR-30c-5p | MIMAT0000244 | 10 | 0.561 (0.505; 0.618) | 37138538 |
| hsa-miR-29a-3p | MIMAT0000086 | 15 | 0.558 (0.502; 0.615) | 33820510 |
| hsa-miR-210-3p | MIMAT0000267 | 10 | 0.556 (0.500; 0.613) | 33824379 |
| hsa-miR-451a | MIMAT0001631 | 1 | 0.544 (0.487; 0.601) | 27974462 |
| hsa-miR-223-3p | MIMAT0000280 | 9 | 0.543 (0.487; 0.600) | 33215881 |
| hsa-miR-146a-5p | MIMAT0000449 | 17 | 0.539 (0.483; 0.596) | 23438607 |
| hsa-miR-146b-5p | MIMAT0002809 | 10 | 0.538 (0.481; 0.594) | 33830643 |
| hsa-miR-192-5p | MIMAT0000222 | 5 | 0.523 (0.466; 0.579) | 29719295 |
| hsa-miR-21-5p | MIMAT0000076 | 13 | 0.516 (0.459; 0.573) | 33830643, 33215881, 29377565, 22041329 |
| hsa-miR-423-3p | MIMAT0001340 | 17 | 0.516 (0.459; 0.573) | 23438607, 26580972 |
| hsa-miR-182-5p | MIMAT0000259 | 10 | 0.512 (0.431; 0.545) | 32598816, 32102357 |
| hsa-miR-26a-5p | MIMAT0000082 | 10 | 0.512 (0.431; 0.545) | 37138538 |
| hsa-miR-133b | MIMAT0000770 | 9 | 0.510 (0.453; 0.567) | 32102357 |
| hsa-miR-1246 | MIMAT0005898 | 9 | 0.510 (0.453; 0.567) | 23247724 |
| hsa-miR-27b-3p | MIMAT0000419 | 14 | 0.506 (0.449; 0.563) | 29335596 |
| hsa-miR-130b-3p | MIMAT0000691 | 10 | 0.505 (0.438; 0.552) | 33824379 |
| hsa-miR-500a-3p | MIMAT0002871 | 9 | 0.504 (0.447; 0.560) | 23247724 |
| hsa-miR-361-5p | MIMAT0000703 | 17 | 0.502 (0.441; 0.555) | 23438607 |
| Ischemic cardiomyopathy | | | | |
| hsa-miR-150-5p | MIMAT0000451 | 20 | 0.772 (0.739; 0.805) | 36187500, 33443726, 31933487, 30745540, 28259597 |
| hsa-miR-484 | MIMAT0002174 | 14 | 0.771 (0.738; 0.805) | 34612058 |
| hsa-miR-210-3p | MIMAT0000267 | 16 | 0.754 (0.718; 0.789) | 33934544, 33783502, 23660476 |
| hsa-miR-106b-5p | MIMAT0000680 | 18 | 0.737 (0.701; 0.773) | 30090932, 37234747, 23388090 |
| hsa-miR-221-3p | MIMAT0000278 | 16 | 0.736 (0.700; 0.772) | 34247489, 30523241, 25739750 |
| hsa-miR-22-3p | MIMAT0000077 | 27 | 0.727 (0.691; 0.762) | 36207069, 36161443, 35473531, 33934544, 28274577 |
| hsa-miR-125b-5p | MIMAT0000423 | 11 | 0.720 (0.685; 0.755) | 35096206, 27072074 |
| hsa-miR-199a-5p | MIMAT0000231 | 24 | 0.713 (0.677; 0.749) | 29378531, 28293796, 27072074, 26569364, 26345695, 23696613, 34247489 |
| hsa-miR-30c-5p | MIMAT0000244 | 20 | 0.713 (0.676; 0.749) | 37138538, 36161443, 25739750 |
| hsa-miR-34a-5p | MIMAT0000255 | 14 | 0.702 (0.665; 0.739) | 33053610, 29378531, 29314582, 24714087 |
| hsa-miR-361-5p | MIMAT0000703 | 17 | 0.685 (0.647; 0.722) | 23438607 |
| hsa-miR-18b-5p | MIMAT0001412 | 10 | 0.684 (0.647; 0.721) | 36161443, 34797300 |
| hsa-miR-101-3p | MIMAT0000099 | 14 | 0.680 (0.642; 0.718) | 34612058, 27058529, 37752740 |
| hsa-miR-16-5p | MIMAT0000069 | 12 | 0.657 (0.618; 0.696) | 34797300, 33443726, 27072074 |
| hsa-miR-30a-5p | MIMAT0000087 | 23 | 0.653 (0.614; 0.692) | 35690861, 32124924, 31933487, 29959359, 29314582, 27072074, 23660476 |
| hsa-miR-223-3p | MIMAT0000280 | 27 | 0.653 (0.614; 0.691) | 36207069, 30054123, 28666417, 28293796, 26569364 |
| hsa-miR-495-3p | MIMAT0002817 | 35 | 0.651 (0.612; 0.689) | 33053610 |
| hsa-miR-29b-3p | MIMAT0000100 | 12 | 0.640 (0.601; 0.679) | 33443726, 31933487, 27072074 |
| hsa-miR-92a-3p | MIMAT0000092 | 10 | 0.639 (0.600; 0.678) | 36549862, 32361531 |
| hsa-let-7a-5p | MIMAT0000062 | 15 | 0.638 (0.599; 0.677) | 32400052, 27072074 |
| hsa-miR-320a | MIMAT0000510 | 20 | 0.633 (0.594; 0.673) | 36161443, 29719295, 28948688, 27072074 |
| hsa-miR-423-3p | MIMAT0001340 | 43 | 0.629 (0.589; 0.669) | 36207069, 36187500, 36161443, 36149935, 35552172, 34797300, 32984995, 31757654, 29378531, 29314582, 28948688, 28562533, 26580972, 26569364, 25776937, 23438607, 23347612, 23242657, 22858654, 22735911, 20185794 |
| hsa-miR-939-5p | MIMAT0004982 | 14 | 0.627 (0.588; 0.666) | 34612058 |
| hsa-miR-1202 | MIMAT0005865 | 18 | 0.624 (0.584; 0.663) | 24961598, 29332918 |
| hsa-miR-30d-5p | MIMAT0000245 | 17 | 0.623 (0.584; 0.663) | 34797300, 28214846, 27072074, 25995320, 25643195 |
| hsa-miR-328-3p | MIMAT0000752 | 20 | 0.619 (0.580; 0.659) | 36161443, 25739750, 24833470 |
| hsa-miR-29a-3p | MIMAT0000086 | 18 | 0.614 (0.575; 0.654) | 33443726, 31933487, 30356307 |
| hsa-miR-660-5p | MIMAT0003338 | 19 | 0.605 (0.564; 0.645) | 26683101 |
| hsa-miR-502-5p | MIMAT0002873 | 11 | 0.604 (0.564; 0.644) | 25643195 |
| hsa-miR-92b-3p | MIMAT0003218 | 37 | 0.597 (0.557; 0.637) | 36161443, 33053610, 29719295 |
| hsa-miR-21-5p | MIMAT0000076 | 48 | 0.596 (0.556; 0.636) | 36549862, 36207069, 36197244, 36161443, 36149935, 35726831, 34797300, 33934544, 33877895, 33443726, 32735867, 32400052, 32184430, 32176569, 32124924, 31109008, 29506053, 29314582, 28944900, 27072074, 26580972, 26498537, 26221607, 26188824, 25643195, 22735911 |
| hsa-miR-181c-5p | MIMAT0000258 | 35 | 0.594 (0.554; 0.634) | 33053610 |
| hsa-miR-27a-3p | MIMAT0000084 | 22 | 0.593 (0.553; 0.633) | 34797300, 28293796, 27072074, 26569364, 26345695 |
| hsa-miR-144-3p | MIMAT0000436 | 16 | 0.592 (0.551; 0.632) | 35235759, 34612058, 32598816 |
| hsa-miR-26b-5p | MIMAT0000083 | 10 | 0.588 (0.547; 0.628) | 34797300, 29506053, 26345695 |
| hsa-miR-192-5p | MIMAT0000222 | 37 | 0.580 (0.539; 0.620) | 33053610, 29927310, 29719295 |
| hsa-miR-194-5p | MIMAT0000460 | 35 | 0.572 (0.531; 0.612) | 33053610 |
| hsa-miR-1228-3p | MIMAT0005583 | 11 | 0.565 (0.524; 0.605) | 25643195 |
| hsa-miR-19b-3p | MIMAT0000074 | 18 | 0.563 (0.522; 0.604) | 36161443, 34612058, 34247489, 30230713, 28091585 |
| hsa-miR-24-3p | MIMAT0000080 | 17 | 0.561 (0.520; 0.602) | 30356307, 26221607 |
| hsa-miR-744-5p | MIMAT0004945 | 36 | 0.555 (0.514; 0.596) | 33053610, 25643195 |
| hsa-miR-486-5p | MIMAT0002177 | 12 | 0.553 (0.513; 0.594) | 33443726, 32598816, 28949058 |
| hsa-miR-30e-5p | MIMAT0000692 | 13 | 0.551 (0.510; 0.591) | 34797300, 33443726, 27072074, 26345695 |
| hsa-miR-26a-5p | MIMAT0000082 | 13 | 0.550 (0.509; 0.591) | 37138538, 32735867, 28259597, 26221607 |
| hsa-miR-652-3p | MIMAT0003322 | 21 | 0.541 (0.501; 0.582) | 33249927, 28293796, 26569364, 26345695 |
| hsa-miR-27b-3p | MIMAT0000419 | 12 | 0.538 (0.497; 0.579) | 33443726, 27072074, 23696613 |
| hsa-miR-1285-3p | MIMAT0005876 | 19 | 0.518 (0.477; 0.559) | 26683101 |
| hsa-miR-126-3p | MIMAT0000445 | 31 | 0.514 (0.473; 0.555) | 37138538, 36549862, 36187500, 35726831, 34797300, 31757654, 30356307, 30054123, 29378531, 29314582, 28666417, 26580972, 26313139, 23438607, 22011751 |
| hsa-miR-340-5p | MIMAT0004692 | 22 | 0.511 (0.470; 0.552) | 31885737 |
|  | | | | |
|  | | | | |
|  | | | | |

**Table S5: Result metrics for the comparison of ACS versus control.** This table shows the statistical results from matched analysis and logistic regression, adjusted for age and sex. The 'ttest_rawp' column shows the raw p-values from the matched analysis t-test, 'ttest_adjp' shows the Bonferroni-Holm adjusted p-values, 'glm_rawp' shows the raw p-values from the logistic regression model, 'AUC' shows the area under the curve values reflecting the diagnostic accuracy, and 'log2FoldChange' shows the magnitude of differential expression on a logarithmic scale.

| miRNA | ttest_rawp | ttest_adjp | glm_rawp | AUC | log2FoldChange |
| --- | --- | --- | --- | --- | --- |
| hsa-miR-4318 | 2.01E-08 | 8.85E-06 | 1.89E-06 | 0.614 (0.563; 0.665) | -0.149 |
| hsa-miR-652-3p | 4.06E-08 | 1.78E-05 | 2.81E-06 | 0.601 (0.55; 0.652) | -0.126 |
| hsa-miR-451a | 1.62E-07 | 7.11E-05 | 8.37E-05 | 0.608 (0.556; 0.66) | -0.030 |
| hsa-miR-17-5p | 1.22E-06 | 5.34E-04 | 1.05E-04 | 0.622 (0.571; 0.673) | -0.181 |
| hsa-miR-342-5p | 4.81E-06 | 2.10E-03 | 5.19E-04 | 0.586 (0.535; 0.638) | -0.125 |
| hsa-miR-1202 | 5.73E-06 | 2.49E-03 | 1.24E-03 | 0.598 (0.547; 0.649) | 0.131 |
| hsa-miR-93-5p | 9.12E-06 | 3.96E-03 | 2.23E-04 | 0.618 (0.567; 0.668) | -0.170 |
| hsa-miR-7110-5p | 9.37E-06 | 4.06E-03 | 3.80E-04 | 0.571 (0.519; 0.623) | 0.150 |
| hsa-miR-664b-5p | 1.11E-05 | 4.81E-03 | 2.49E-03 | 0.577 (0.526; 0.629) | 0.239 |
| hsa-miR-126-5p | 1.51E-05 | 6.51E-03 | 1.84E-03 | 0.625 (0.574; 0.675) | -0.160 |
| hsa-miR-6511b-3p | 1.81E-05 | 7.79E-03 | 1.16E-04 | 0.583 (0.531; 0.634) | -0.108 |
| hsa-miR-1225-5p | 2.03E-05 | 8.72E-03 | 1.04E-03 | 0.604 (0.553; 0.655) | 0.077 |
| hsa-miR-20b-5p | 2.47E-05 | 1.06E-02 | 1.20E-03 | 0.612 (0.561; 0.663) | -0.155 |
| hsa-miR-20a-5p | 2.71E-05 | 1.16E-02 | 5.71E-03 | 0.615 (0.564; 0.665) | -0.175 |
| hsa-miR-625-3p | 3.63E-05 | 1.55E-02 | 8.45E-04 | 0.554 (0.502; 0.606) | -0.053 |
| hsa-miR-6803-3p | 5.46E-05 | 2.32E-02 | 3.16E-04 | 0.561 (0.509; 0.612) | -0.071 |
| hsa-miR-126-3p | 5.59E-05 | 2.37E-02 | 1.89E-03 | 0.609 (0.558; 0.66) | -0.186 |
| hsa-miR-101-3p | 6.38E-05 | 2.70E-02 | 2.76E-05 | 0.617 (0.566; 0.667) | -0.238 |
| hsa-miR-4732-3p | 7.24E-05 | 3.06E-02 | 1.88E-03 | 0.535 (0.483; 0.587) | -0.129 |
| hsa-miR-15a-5p | 8.41E-05 | 3.54E-02 | 8.46E-04 | 0.619 (0.568; 0.67) | -0.141 |
| hsa-miR-18a-5p | 9.86E-05 | 4.14E-02 | 9.61E-04 | 0.607 (0.556; 0.658) | -0.205 |
| hsa-miR-532-5p | 9.98E-05 | 4.18E-02 | 1.54E-05 | 0.591 (0.54; 0.642) | -0.108 |
| hsa-miR-642a-3p | 1.04E-04 | 4.35E-02 | 1.78E-03 | 0.571 (0.52; 0.623) | 0.126 |
| hsa-miR-664a-3p | 1.26E-04 | 5.26E-02 | 4.42E-04 | 0.595 (0.543; 0.646) | -0.080 |
| hsa-miR-18b-5p | 1.59E-04 | 6.63E-02 | 5.18E-04 | 0.612 (0.561; 0.662) | -0.183 |
| hsa-miR-4270 | 1.95E-04 | 8.11E-02 | 4.37E-03 | 0.588 (0.536; 0.64) | 0.092 |
| hsa-miR-301a-3p | 2.37E-04 | 9.81E-02 | 3.29E-03 | 0.598 (0.547; 0.649) | -0.182 |
| hsa-miR-7-5p | 2.71E-04 | 1.12E-01 | 5.52E-05 | 0.608 (0.557; 0.659) | -0.158 |
| hsa-miR-144-5p | 3.24E-04 | 1.34E-01 | 3.20E-04 | 0.632 (0.581; 0.682) | -0.241 |
| hsa-miR-17-3p | 3.35E-04 | 1.38E-01 | 1.17E-02 | 0.572 (0.521; 0.624) | -0.138 |
| hsa-miR-762 | 3.38E-04 | 1.38E-01 | 2.19E-02 | 0.567 (0.515; 0.619) | 0.085 |
| hsa-miR-4284 | 3.64E-04 | 1.49E-01 | 1.63E-02 | 0.566 (0.514; 0.618) | 0.103 |
| hsa-miR-505-5p | 3.81E-04 | 1.56E-01 | 4.83E-04 | 0.565 (0.514; 0.617) | -0.099 |
| hsa-miR-501-3p | 4.40E-04 | 1.79E-01 | 3.39E-04 | 0.558 (0.506; 0.61) | -0.080 |
| hsa-miR-629-5p | 4.53E-04 | 1.84E-01 | 1.01E-03 | 0.578 (0.527; 0.63) | -0.099 |
| hsa-miR-628-3p | 4.62E-04 | 1.87E-01 | 4.83E-04 | 0.577 (0.526; 0.628) | -0.071 |
| hsa-miR-16-5p | 4.78E-04 | 1.93E-01 | 1.78E-02 | 0.585 (0.534; 0.636) | -0.076 |
| hsa-miR-4516 | 4.82E-04 | 1.94E-01 | 6.98E-03 | 0.553 (0.501; 0.605) | 0.119 |
| hsa-miR-3656 | 5.00E-04 | 2.01E-01 | 2.78E-03 | 0.557 (0.505; 0.609) | -0.129 |
| hsa-miR-550a-3-5p | 5.10E-04 | 2.04E-01 | 1.02E-01 | 0.537 (0.485; 0.589) | -0.088 |
| hsa-miR-186-5p | 5.17E-04 | 2.07E-01 | 1.41E-03 | 0.584 (0.532; 0.635) | 0.089 |
| hsa-miR-4669 | 5.30E-04 | 2.12E-01 | 1.73E-02 | 0.551 (0.499; 0.604) | 0.110 |
| hsa-miR-361-3p | 7.35E-04 | 2.92E-01 | 9.73E-03 | 0.545 (0.493; 0.597) | -0.089 |
| hsa-miR-942-3p | 8.01E-04 | 3.18E-01 | 2.16E-03 | 0.567 (0.516; 0.619) | -0.094 |
| hsa-miR-6749-5p | 8.07E-04 | 3.19E-01 | 1.13E-01 | 0.563 (0.511; 0.614) | 0.126 |
| hsa-miR-144-3p | 8.88E-04 | 3.51E-01 | 1.59E-03 | 0.582 (0.53; 0.633) | -0.262 |
| hsa-let-7a-5p | 9.36E-04 | 3.69E-01 | 3.61E-02 | 0.572 (0.521; 0.624) | -0.079 |
| hsa-miR-532-3p | 1.00E-03 | 3.93E-01 | 3.30E-02 | 0.524 (0.472; 0.576) | -0.093 |
| hsa-miR-140-5p | 1.05E-03 | 4.10E-01 | 9.25E-03 | 0.595 (0.543; 0.646) | -0.142 |
| hsa-miR-107 | 1.07E-03 | 4.18E-01 | 4.49E-04 | 0.608 (0.557; 0.658) | -0.070 |
| hsa-miR-5787 | 1.07E-03 | 4.18E-01 | 4.16E-02 | 0.599 (0.547; 0.65) | 0.132 |
| hsa-miR-96-5p | 1.10E-03 | 4.27E-01 | 5.12E-03 | 0.593 (0.542; 0.644) | -0.161 |
| hsa-miR-196b-5p | 1.17E-03 | 4.55E-01 | 4.18E-01 | 0.552 (0.5; 0.604) | -0.125 |
| hsa-let-7f-5p | 1.20E-03 | 4.63E-01 | 3.31E-02 | 0.586 (0.534; 0.637) | -0.088 |
| hsa-miR-100-5p | 1.65E-03 | 6.36E-01 | 2.86E-03 | 0.53 (0.418; 0.523) | 0.429 |
| hsa-miR-200c-3p | 1.70E-03 | 6.54E-01 | 1.23E-02 | 0.589 (0.538; 0.641) | -0.069 |
| hsa-miR-15b-5p | 2.05E-03 | 7.87E-01 | 1.74E-01 | 0.533 (0.481; 0.585) | -0.056 |
| hsa-miR-30d-5p | 2.30E-03 | 8.82E-01 | 1.32E-02 | 0.601 (0.55; 0.652) | 0.071 |
| hsa-miR-629-3p | 2.38E-03 | 9.10E-01 | 8.03E-02 | 0.525 (0.473; 0.577) | -0.070 |
| hsa-miR-625-5p | 2.60E-03 | 9.90E-01 | 1.53E-01 | 0.518 (0.465; 0.57) | -0.094 |
| hsa-let-7i-5p | 2.67E-03 | 1.00E+00 | 4.66E-04 | 0.605 (0.554; 0.656) | -0.110 |
| hsa-miR-4672 | 2.91E-03 | 1.00E+00 | 1.04E-02 | 0.553 (0.502; 0.605) | -0.081 |
| hsa-miR-98-5p | 3.70E-03 | 1.00E+00 | 4.00E-02 | 0.581 (0.53; 0.633) | -0.111 |
| hsa-miR-454-3p | 3.70E-03 | 1.00E+00 | 1.59E-01 | 0.581 (0.529; 0.632) | -0.147 |
| hsa-miR-320c | 3.72E-03 | 1.00E+00 | 1.29E-03 | 0.553 (0.501; 0.605) | -0.078 |
| hsa-miR-371b-5p | 4.11E-03 | 1.00E+00 | 1.34E-02 | 0.54 (0.488; 0.592) | -0.073 |
| hsa-miR-222-3p | 4.31E-03 | 1.00E+00 | 5.86E-03 | 0.567 (0.515; 0.619) | -0.098 |
| hsa-miR-1246 | 4.85E-03 | 1.00E+00 | 2.66E-01 | 0.562 (0.51; 0.614) | 0.106 |
| hsa-miR-4436b-5p | 4.87E-03 | 1.00E+00 | 5.35E-02 | 0.597 (0.546; 0.649) | 0.071 |
| hsa-miR-23a-3p | 4.88E-03 | 1.00E+00 | 7.04E-04 | 0.582 (0.531; 0.634) | 0.090 |
| hsa-miR-4485-5p | 4.89E-03 | 1.00E+00 | 2.61E-02 | 0.576 (0.524; 0.628) | 0.109 |
| hsa-miR-324-3p | 5.14E-03 | 1.00E+00 | 1.09E-02 | 0.549 (0.497; 0.601) | -0.054 |
| hsa-miR-5739 | 5.63E-03 | 1.00E+00 | 3.31E-01 | 0.541 (0.489; 0.593) | 0.126 |
| hsa-miR-590-5p | 5.66E-03 | 1.00E+00 | 1.67E-03 | 0.581 (0.53; 0.633) | -0.159 |
| hsa-miR-598-3p | 5.75E-03 | 1.00E+00 | 5.94E-02 | 0.553 (0.501; 0.605) | -0.073 |
| hsa-miR-2861 | 5.81E-03 | 1.00E+00 | 5.30E-03 | 0.55 (0.498; 0.602) | -0.078 |
| hsa-miR-664a-5p | 5.81E-03 | 1.00E+00 | 1.67E-01 | 0.543 (0.49; 0.595) | 0.082 |
| hsa-miR-6127 | 5.91E-03 | 1.00E+00 | 1.92E-01 | 0.562 (0.511; 0.614) | 0.074 |
| hsa-miR-8485 | 6.19E-03 | 1.00E+00 | 6.34E-02 | 0.566 (0.514; 0.618) | 0.131 |
| hsa-miR-199b-5p | 6.56E-03 | 1.00E+00 | 6.83E-04 | 0.577 (0.526; 0.629) | 0.149 |
| hsa-miR-550b-2-5p | 6.88E-03 | 1.00E+00 | 4.66E-01 | 0.516 (0.464; 0.568) | -0.057 |
| hsa-miR-93-3p | 6.89E-03 | 1.00E+00 | 4.61E-02 | 0.53 (0.477; 0.582) | -0.065 |
| hsa-miR-610 | 7.06E-03 | 1.00E+00 | 1.63E-02 | 0.55 (0.498; 0.602) | -0.050 |
| hsa-miR-7641 | 7.46E-03 | 1.00E+00 | 2.34E-01 | 0.557 (0.506; 0.609) | 0.106 |
| hsa-miR-338-3p | 7.52E-03 | 1.00E+00 | 9.18E-03 | 0.551 (0.499; 0.603) | 0.121 |
| hsa-miR-1973 | 8.68E-03 | 1.00E+00 | 8.21E-02 | 0.557 (0.505; 0.609) | 0.089 |
| hsa-miR-6803-5p | 9.09E-03 | 1.00E+00 | 2.90E-02 | 0.54 (0.488; 0.592) | -0.108 |
| hsa-miR-5001-5p | 9.86E-03 | 1.00E+00 | 9.25E-03 | 0.553 (0.501; 0.605) | -0.058 |
| hsa-miR-3651 | 9.91E-03 | 1.00E+00 | 5.11E-02 | 0.534 (0.482; 0.586) | 0.083 |
| hsa-miR-1908-3p | 9.99E-03 | 1.00E+00 | 4.26E-02 | 0.55 (0.498; 0.602) | 0.042 |
| hsa-miR-6891-5p | 1.02E-02 | 1.00E+00 | 2.34E-01 | 0.535 (0.483; 0.587) | 0.060 |
| hsa-miR-125b-5p | 1.04E-02 | 1.00E+00 | 2.38E-02 | 0.524 (0.472; 0.576) | 0.171 |
| hsa-miR-660-5p | 1.12E-02 | 1.00E+00 | 5.29E-02 | 0.578 (0.526; 0.63) | -0.097 |
| hsa-let-7g-5p | 1.16E-02 | 1.00E+00 | 4.05E-02 | 0.605 (0.554; 0.656) | -0.078 |
| hsa-miR-7107-5p | 1.17E-02 | 1.00E+00 | 8.79E-02 | 0.535 (0.483; 0.588) | 0.085 |
| hsa-miR-103a-3p | 1.19E-02 | 1.00E+00 | 2.94E-03 | 0.58 (0.529; 0.631) | -0.053 |
| hsa-miR-4732-5p | 1.24E-02 | 1.00E+00 | 2.12E-03 | 0.557 (0.505; 0.609) | -0.082 |
| hsa-miR-6125 | 1.33E-02 | 1.00E+00 | 1.23E-02 | 0.549 (0.497; 0.601) | -0.067 |
| hsa-miR-505-3p | 1.37E-02 | 1.00E+00 | 1.21E-02 | 0.58 (0.528; 0.631) | 0.072 |
| hsa-miR-378d | 1.45E-02 | 1.00E+00 | 1.91E-03 | 0.568 (0.516; 0.619) | -0.062 |
| hsa-miR-4505 | 1.46E-02 | 1.00E+00 | 2.12E-02 | 0.549 (0.497; 0.601) | -0.065 |
| hsa-let-7d-5p | 1.46E-02 | 1.00E+00 | 2.21E-01 | 0.549 (0.497; 0.601) | -0.057 |
| hsa-miR-1255a | 1.55E-02 | 1.00E+00 | 4.15E-02 | 0.579 (0.527; 0.631) | -0.040 |
| hsa-miR-29a-3p | 1.57E-02 | 1.00E+00 | 2.28E-02 | 0.549 (0.498; 0.601) | 0.082 |
| hsa-miR-7704 | 1.62E-02 | 1.00E+00 | 1.53E-02 | 0.537 (0.485; 0.589) | -0.056 |
| hsa-miR-4739 | 1.67E-02 | 1.00E+00 | 1.07E-01 | 0.544 (0.492; 0.596) | 0.057 |
| hsa-miR-6090 | 1.69E-02 | 1.00E+00 | 1.46E-01 | 0.551 (0.498; 0.603) | 0.062 |
| hsa-miR-4746-3p | 1.69E-02 | 1.00E+00 | 5.01E-02 | 0.575 (0.524; 0.627) | 0.041 |
| hsa-miR-130a-3p | 1.74E-02 | 1.00E+00 | 5.68E-02 | 0.577 (0.525; 0.629) | -0.089 |
| hsa-miR-6826-5p | 1.81E-02 | 1.00E+00 | 2.29E-01 | 0.545 (0.493; 0.597) | 0.095 |
| hsa-miR-6124 | 1.82E-02 | 1.00E+00 | 4.36E-01 | 0.54 (0.488; 0.593) | 0.054 |
| hsa-miR-584-5p | 1.84E-02 | 1.00E+00 | 7.04E-04 | 0.553 (0.502; 0.605) | -0.082 |
| hsa-miR-374c-5p | 1.85E-02 | 1.00E+00 | 3.86E-02 | 0.557 (0.505; 0.608) | -0.091 |
| hsa-miR-197-5p | 1.89E-02 | 1.00E+00 | 3.71E-01 | 0.514 (0.461; 0.566) | 0.078 |
| hsa-miR-210-3p | 1.93E-02 | 1.00E+00 | 2.41E-03 | 0.568 (0.516; 0.619) | -0.107 |
| hsa-miR-486-5p | 2.03E-02 | 1.00E+00 | 3.16E-02 | 0.531 (0.479; 0.583) | -0.030 |
| hsa-miR-221-3p | 2.10E-02 | 1.00E+00 | 1.34E-02 | 0.566 (0.515; 0.618) | -0.092 |
| hsa-miR-6819-3p | 2.16E-02 | 1.00E+00 | 2.43E-02 | 0.562 (0.511; 0.614) | 0.043 |
| hsa-miR-374a-5p | 2.24E-02 | 1.00E+00 | 2.64E-01 | 0.549 (0.497; 0.601) | -0.134 |
| hsa-miR-1285-3p | 2.38E-02 | 1.00E+00 | 1.82E-01 | 0.512 (0.46; 0.564) | -0.040 |
| hsa-miR-199a-3p | 2.47E-02 | 1.00E+00 | 8.69E-01 | 0.559 (0.507; 0.61) | -0.110 |
| hsa-miR-150-5p | 2.65E-02 | 1.00E+00 | 7.56E-01 | 0.513 (0.461; 0.565) | -0.092 |
| hsa-miR-92a-3p | 2.86E-02 | 1.00E+00 | 1.12E-02 | 0.531 (0.478; 0.583) | -0.041 |
| hsa-miR-3163 | 2.93E-02 | 1.00E+00 | 1.40E-01 | 0.537 (0.485; 0.589) | -0.057 |
| hsa-miR-423-5p | 3.02E-02 | 1.00E+00 | 1.28E-03 | 0.532 (0.479; 0.584) | -0.055 |
| hsa-miR-3665 | 3.05E-02 | 1.00E+00 | 4.26E-02 | 0.547 (0.495; 0.599) | -0.055 |
| hsa-miR-129-1-3p | 3.08E-02 | 1.00E+00 | 3.63E-01 | 0.506 (0.453; 0.558) | -0.035 |
| hsa-miR-21-5p | 3.20E-02 | 1.00E+00 | 3.88E-02 | 0.564 (0.512; 0.615) | -0.074 |
| hsa-miR-1207-5p | 3.31E-02 | 1.00E+00 | 3.18E-01 | 0.525 (0.473; 0.578) | 0.053 |
| hsa-miR-942-5p | 3.50E-02 | 1.00E+00 | 8.44E-02 | 0.515 (0.463; 0.568) | -0.074 |
| hsa-miR-5194 | 3.51E-02 | 1.00E+00 | 4.68E-01 | 0.533 (0.415; 0.519) | 0.052 |
| hsa-miR-125a-5p | 3.55E-02 | 1.00E+00 | 1.34E-02 | 0.575 (0.523; 0.626) | 0.082 |
| hsa-miR-33b-3p | 3.84E-02 | 1.00E+00 | 6.06E-02 | 0.571 (0.52; 0.623) | 0.040 |
| hsa-miR-378g | 3.88E-02 | 1.00E+00 | 2.93E-03 | 0.571 (0.519; 0.622) | -0.061 |
| hsa-miR-574-5p | 3.99E-02 | 1.00E+00 | 4.19E-01 | 0.522 (0.47; 0.574) | 0.085 |
| hsa-miR-6165 | 4.12E-02 | 1.00E+00 | 4.76E-01 | 0.516 (0.463; 0.568) | 0.071 |
| hsa-miR-4787-3p | 4.18E-02 | 1.00E+00 | 6.04E-02 | 0.562 (0.51; 0.614) | 0.038 |
| hsa-miR-940 | 4.22E-02 | 1.00E+00 | 9.30E-02 | 0.529 (0.477; 0.581) | 0.046 |
| hsa-miR-4507 | 4.39E-02 | 1.00E+00 | 6.18E-02 | 0.533 (0.481; 0.585) | -0.073 |
| hsa-miR-4484 | 4.43E-02 | 1.00E+00 | 4.38E-02 | 0.59 (0.538; 0.641) | 0.030 |
| hsa-miR-4664-3p | 4.45E-02 | 1.00E+00 | 4.72E-02 | 0.558 (0.506; 0.61) | 0.035 |
| hsa-miR-6779-5p | 4.48E-02 | 1.00E+00 | 5.41E-02 | 0.523 (0.471; 0.575) | -0.039 |
| hsa-miR-99b-5p | 4.51E-02 | 1.00E+00 | 3.81E-03 | 0.578 (0.527; 0.63) | 0.076 |
| hsa-miR-4685-5p | 4.62E-02 | 1.00E+00 | 3.25E-03 | 0.547 (0.495; 0.599) | -0.096 |
| hsa-miR-4763-3p | 4.79E-02 | 1.00E+00 | 9.41E-02 | 0.544 (0.492; 0.596) | 0.054 |
| hsa-miR-939-5p | 4.90E-02 | 1.00E+00 | 6.54E-03 | 0.565 (0.513; 0.617) | -0.056 |
| hsa-miR-502-5p | 5.07E-02 | 1.00E+00 | 3.67E-01 | 0.537 (0.485; 0.589) | -0.071 |
| hsa-miR-223-3p | 5.08E-02 | 1.00E+00 | 2.44E-03 | 0.58 (0.528; 0.632) | 0.037 |
| hsa-miR-1539 | 5.17E-02 | 1.00E+00 | 5.58E-02 | 0.564 (0.513; 0.616) | 0.036 |
| hsa-miR-155-5p | 5.41E-02 | 1.00E+00 | 2.06E-01 | 0.563 (0.512; 0.615) | -0.051 |
| hsa-miR-8069 | 5.46E-02 | 1.00E+00 | 1.32E-01 | 0.552 (0.5; 0.604) | 0.050 |
| hsa-miR-149-5p | 5.58E-02 | 1.00E+00 | 3.58E-02 | 0.585 (0.533; 0.636) | 0.035 |
| hsa-miR-6068 | 5.59E-02 | 1.00E+00 | 1.08E-01 | 0.522 (0.47; 0.574) | -0.047 |
| hsa-miR-296-5p | 5.61E-02 | 1.00E+00 | 1.43E-02 | 0.529 (0.477; 0.582) | -0.048 |
| hsa-miR-22-5p | 5.65E-02 | 1.00E+00 | 5.70E-02 | 0.541 (0.488; 0.593) | -0.061 |
| hsa-miR-4725-5p | 5.69E-02 | 1.00E+00 | 9.66E-02 | 0.534 (0.482; 0.587) | 0.041 |
| hsa-miR-3200-5p | 5.69E-02 | 1.00E+00 | 1.91E-01 | 0.533 (0.481; 0.585) | -0.073 |
| hsa-miR-6777-3p | 5.71E-02 | 1.00E+00 | 6.44E-02 | 0.564 (0.512; 0.616) | 0.030 |
| hsa-miR-3162-5p | 5.82E-02 | 1.00E+00 | 3.48E-01 | 0.525 (0.473; 0.577) | 0.048 |
| hsa-miR-6088 | 5.88E-02 | 1.00E+00 | 1.47E-01 | 0.543 (0.491; 0.595) | 0.033 |
| hsa-miR-6131 | 6.34E-02 | 1.00E+00 | 6.05E-01 | 0.561 (0.509; 0.613) | 0.053 |
| hsa-miR-502-3p | 6.34E-02 | 1.00E+00 | 3.55E-02 | 0.538 (0.486; 0.59) | -0.053 |
| hsa-miR-6717-5p | 6.37E-02 | 1.00E+00 | 5.57E-01 | 0.546 (0.494; 0.598) | 0.052 |
| hsa-miR-642b-3p | 6.45E-02 | 1.00E+00 | 3.98E-01 | 0.53 (0.478; 0.582) | 0.045 |
| hsa-miR-6737-3p | 6.45E-02 | 1.00E+00 | 5.79E-02 | 0.556 (0.504; 0.608) | 0.039 |
| hsa-miR-3940-3p | 6.54E-02 | 1.00E+00 | 1.49E-01 | 0.559 (0.507; 0.611) | 0.032 |
| hsa-miR-320e | 6.67E-02 | 1.00E+00 | 2.42E-02 | 0.533 (0.481; 0.585) | -0.050 |
| hsa-miR-362-5p | 6.67E-02 | 1.00E+00 | 4.58E-02 | 0.537 (0.485; 0.59) | -0.047 |
| hsa-miR-6789-5p | 6.89E-02 | 1.00E+00 | 2.08E-01 | 0.524 (0.472; 0.576) | -0.070 |
| hsa-miR-4515 | 7.11E-02 | 1.00E+00 | 2.72E-01 | 0.568 (0.517; 0.62) | 0.041 |
| hsa-miR-500b-5p | 7.39E-02 | 1.00E+00 | 1.08E-01 | 0.547 (0.494; 0.599) | -0.049 |
| hsa-miR-769-5p | 7.40E-02 | 1.00E+00 | 1.71E-01 | 0.56 (0.508; 0.611) | 0.045 |
| hsa-miR-3135b | 7.46E-02 | 1.00E+00 | 2.95E-01 | 0.52 (0.468; 0.572) | 0.127 |
| hsa-miR-4758-3p | 7.52E-02 | 1.00E+00 | 5.18E-02 | 0.555 (0.503; 0.606) | 0.032 |
| hsa-miR-3162-3p | 7.70E-02 | 1.00E+00 | 1.20E-01 | 0.552 (0.499; 0.604) | 0.043 |
| hsa-miR-3679-5p | 7.76E-02 | 1.00E+00 | 3.90E-01 | 0.527 (0.474; 0.58) | 0.045 |
| hsa-miR-4286 | 7.76E-02 | 1.00E+00 | 5.03E-01 | 0.528 (0.475; 0.58) | 0.081 |
| hsa-miR-5100 | 7.87E-02 | 1.00E+00 | 8.81E-01 | 0.541 (0.489; 0.593) | 0.084 |
| hsa-miR-6085 | 7.91E-02 | 1.00E+00 | 7.06E-01 | 0.523 (0.471; 0.575) | 0.071 |
| hsa-miR-4306 | 8.07E-02 | 1.00E+00 | 3.77E-02 | 0.53 (0.478; 0.583) | -0.028 |
| hsa-miR-6069 | 8.08E-02 | 1.00E+00 | 7.76E-02 | 0.558 (0.506; 0.61) | 0.037 |
| hsa-miR-6763-3p | 8.12E-02 | 1.00E+00 | 9.40E-02 | 0.548 (0.496; 0.6) | 0.026 |
| hsa-miR-500a-3p | 8.13E-02 | 1.00E+00 | 2.01E-02 | 0.544 (0.492; 0.596) | -0.051 |
| hsa-miR-6780b-5p | 8.40E-02 | 1.00E+00 | 7.39E-01 | 0.545 (0.493; 0.597) | 0.048 |
| hsa-miR-3653-3p | 8.51E-02 | 1.00E+00 | 1.71E-02 | 0.524 (0.472; 0.576) | 0.077 |
| hsa-miR-575 | 8.71E-02 | 1.00E+00 | 6.85E-01 | 0.541 (0.489; 0.593) | 0.041 |
| hsa-miR-1304-3p | 8.71E-02 | 1.00E+00 | 7.19E-02 | 0.555 (0.503; 0.607) | 0.038 |
| hsa-miR-4665-3p | 9.06E-02 | 1.00E+00 | 1.28E-01 | 0.554 (0.502; 0.606) | 0.036 |
| hsa-miR-4749-3p | 9.28E-02 | 1.00E+00 | 7.24E-02 | 0.549 (0.497; 0.601) | 0.025 |
| hsa-miR-4433a-5p | 9.43E-02 | 1.00E+00 | 7.73E-02 | 0.559 (0.507; 0.611) | 0.028 |
| hsa-miR-29c-5p | 9.58E-02 | 1.00E+00 | 5.45E-01 | 0.521 (0.469; 0.573) | 0.053 |
| hsa-miR-378i | 9.77E-02 | 1.00E+00 | 5.56E-03 | 0.552 (0.5; 0.604) | -0.040 |
| hsa-miR-320d | 9.82E-02 | 1.00E+00 | 4.41E-02 | 0.526 (0.473; 0.578) | -0.045 |
| hsa-miR-142-5p | 1.04E-01 | 1.00E+00 | 1.67E-01 | 0.518 (0.466; 0.57) | -0.061 |
| hsa-miR-320b | 1.05E-01 | 1.00E+00 | 1.72E-02 | 0.535 (0.483; 0.587) | -0.045 |
| hsa-miR-181a-3p | 1.07E-01 | 1.00E+00 | 7.61E-02 | 0.555 (0.503; 0.607) | 0.052 |
| hsa-miR-363-3p | 1.08E-01 | 1.00E+00 | 1.20E-02 | 0.537 (0.485; 0.589) | 0.047 |
| hsa-miR-7974 | 1.08E-01 | 1.00E+00 | 8.76E-02 | 0.553 (0.501; 0.605) | 0.027 |
| hsa-miR-484 | 1.10E-01 | 1.00E+00 | 5.66E-02 | 0.506 (0.453; 0.558) | -0.042 |
| hsa-miR-6875-5p | 1.11E-01 | 1.00E+00 | 5.84E-01 | 0.557 (0.505; 0.609) | 0.052 |
| hsa-miR-4649-3p | 1.11E-01 | 1.00E+00 | 1.23E-01 | 0.552 (0.5; 0.604) | 0.034 |
| hsa-miR-30e-3p | 1.15E-01 | 1.00E+00 | 1.80E-02 | 0.55 (0.498; 0.602) | 0.068 |
| hsa-miR-92b-3p | 1.17E-01 | 1.00E+00 | 1.49E-01 | 0.551 (0.499; 0.603) | 0.024 |
| hsa-miR-1234-3p | 1.17E-01 | 1.00E+00 | 1.36E-01 | 0.544 (0.492; 0.596) | 0.032 |
| hsa-miR-423-3p | 1.17E-01 | 1.00E+00 | 4.26E-01 | 0.502 (0.445; 0.55) | -0.045 |
| hsa-miR-19a-3p | 1.18E-01 | 1.00E+00 | 1.50E-01 | 0.544 (0.492; 0.596) | -0.067 |
| hsa-miR-7977 | 1.21E-01 | 1.00E+00 | 7.35E-01 | 0.521 (0.469; 0.573) | 0.087 |
| hsa-miR-23c | 1.26E-01 | 1.00E+00 | 8.61E-02 | 0.558 (0.506; 0.609) | 0.028 |
| hsa-let-7f-1-3p | 1.32E-01 | 1.00E+00 | 8.26E-02 | 0.581 (0.529; 0.632) | 0.027 |
| hsa-let-7d-3p | 1.34E-01 | 1.00E+00 | 4.72E-02 | 0.57 (0.519; 0.622) | 0.030 |
| hsa-miR-1825 | 1.35E-01 | 1.00E+00 | 1.37E-01 | 0.555 (0.503; 0.607) | 0.032 |
| hsa-miR-2116-3p | 1.37E-01 | 1.00E+00 | 1.19E-01 | 0.551 (0.499; 0.603) | 0.029 |
| hsa-miR-130b-5p | 1.39E-01 | 1.00E+00 | 6.54E-01 | 0.509 (0.439; 0.543) | -0.028 |
| hsa-miR-4465 | 1.39E-01 | 1.00E+00 | 1.34E-01 | 0.543 (0.491; 0.595) | -0.059 |
| hsa-miR-5010-3p | 1.39E-01 | 1.00E+00 | 1.44E-01 | 0.544 (0.492; 0.596) | 0.023 |
| hsa-miR-4485-3p | 1.41E-01 | 1.00E+00 | 4.82E-01 | 0.544 (0.492; 0.596) | 0.044 |
| hsa-miR-4532 | 1.44E-01 | 1.00E+00 | 4.82E-01 | 0.555 (0.503; 0.606) | 0.020 |
| hsa-miR-183-3p | 1.46E-01 | 1.00E+00 | 4.22E-01 | 0.533 (0.481; 0.585) | -0.042 |
| hsa-miR-501-5p | 1.47E-01 | 1.00E+00 | 1.20E-01 | 0.543 (0.491; 0.595) | -0.041 |
| hsa-miR-6740-5p | 1.49E-01 | 1.00E+00 | 8.35E-01 | 0.531 (0.479; 0.583) | 0.038 |
| hsa-miR-6893-5p | 1.52E-01 | 1.00E+00 | 3.38E-01 | 0.528 (0.476; 0.58) | 0.038 |
| hsa-miR-7150 | 1.52E-01 | 1.00E+00 | 5.59E-01 | 0.544 (0.492; 0.596) | 0.037 |
| hsa-miR-4793-5p | 1.58E-01 | 1.00E+00 | 4.61E-01 | 0.532 (0.48; 0.584) | 0.024 |
| hsa-miR-1306-5p | 1.59E-01 | 1.00E+00 | 1.37E-01 | 0.517 (0.465; 0.57) | -0.035 |
| hsa-miR-1281 | 1.61E-01 | 1.00E+00 | 2.12E-01 | 0.551 (0.499; 0.603) | 0.031 |
| hsa-miR-4310 | 1.61E-01 | 1.00E+00 | 1.10E-01 | 0.55 (0.498; 0.602) | 0.028 |
| hsa-miR-1260b | 1.62E-01 | 1.00E+00 | 5.01E-01 | 0.543 (0.491; 0.595) | 0.047 |
| hsa-miR-6879-5p | 1.65E-01 | 1.00E+00 | 9.12E-01 | 0.516 (0.464; 0.568) | 0.035 |
| hsa-miR-4281 | 1.66E-01 | 1.00E+00 | 5.58E-01 | 0.523 (0.47; 0.575) | 0.039 |
| hsa-miR-27b-3p | 1.67E-01 | 1.00E+00 | 9.00E-01 | 0.52 (0.468; 0.573) | -0.062 |
| hsa-miR-624-5p | 1.69E-01 | 1.00E+00 | 5.36E-01 | 0.512 (0.46; 0.565) | -0.058 |
| hsa-miR-6132 | 1.70E-01 | 1.00E+00 | 7.01E-01 | 0.55 (0.498; 0.602) | 0.047 |
| hsa-miR-6858-3p | 1.79E-01 | 1.00E+00 | 1.87E-01 | 0.567 (0.515; 0.619) | 0.023 |
| hsa-miR-7976 | 1.79E-01 | 1.00E+00 | 6.57E-01 | 0.503 (0.451; 0.555) | -0.023 |
| hsa-miR-1228-3p | 1.79E-01 | 1.00E+00 | 1.74E-01 | 0.539 (0.487; 0.591) | 0.026 |
| hsa-miR-145-5p | 1.79E-01 | 1.00E+00 | 1.03E-01 | 0.569 (0.518; 0.621) | 0.050 |
| hsa-miR-6870-3p | 1.80E-01 | 1.00E+00 | 1.89E-01 | 0.519 (0.467; 0.571) | 0.015 |
| hsa-miR-1260a | 1.80E-01 | 1.00E+00 | 7.28E-01 | 0.53 (0.478; 0.582) | 0.053 |
| hsa-miR-1587 | 1.84E-01 | 1.00E+00 | 1.10E-01 | 0.546 (0.494; 0.598) | -0.028 |
| hsa-miR-6785-5p | 1.87E-01 | 1.00E+00 | 7.30E-01 | 0.552 (0.5; 0.604) | 0.063 |
| hsa-miR-4687-3p | 1.94E-01 | 1.00E+00 | 7.18E-01 | 0.524 (0.472; 0.576) | 0.024 |
| hsa-miR-6073 | 1.98E-01 | 1.00E+00 | 1.16E-01 | 0.553 (0.501; 0.605) | -0.043 |
| hsa-miR-342-3p | 1.98E-01 | 1.00E+00 | 4.31E-01 | 0.5 (0.448; 0.553) | -0.036 |
| hsa-miR-3195 | 2.00E-01 | 1.00E+00 | 3.93E-01 | 0.51 (0.458; 0.562) | 0.039 |
| hsa-miR-494-3p | 2.02E-01 | 1.00E+00 | 7.93E-01 | 0.526 (0.474; 0.578) | 0.062 |
| hsa-miR-1915-3p | 2.05E-01 | 1.00E+00 | 9.81E-02 | 0.533 (0.481; 0.585) | -0.034 |
| hsa-miR-6508-5p | 2.05E-01 | 1.00E+00 | 1.49E-01 | 0.545 (0.493; 0.597) | 0.028 |
| hsa-let-7c-5p | 2.17E-01 | 1.00E+00 | 2.32E-02 | 0.543 (0.491; 0.595) | -0.055 |
| hsa-miR-4428 | 2.22E-01 | 1.00E+00 | 7.92E-01 | 0.531 (0.479; 0.584) | 0.037 |
| hsa-miR-3652 | 2.22E-01 | 1.00E+00 | 4.79E-02 | 0.553 (0.501; 0.605) | -0.029 |
| hsa-miR-6731-3p | 2.25E-01 | 1.00E+00 | 1.54E-01 | 0.565 (0.513; 0.616) | 0.018 |
| hsa-miR-6824-3p | 2.26E-01 | 1.00E+00 | 2.15E-01 | 0.546 (0.494; 0.598) | 0.019 |
| hsa-miR-3198 | 2.26E-01 | 1.00E+00 | 8.51E-01 | 0.543 (0.491; 0.595) | 0.034 |
| hsa-miR-326 | 2.30E-01 | 1.00E+00 | 7.33E-01 | 0.517 (0.431; 0.535) | -0.039 |
| hsa-miR-195-5p | 2.30E-01 | 1.00E+00 | 8.18E-01 | 0.535 (0.483; 0.587) | -0.046 |
| hsa-miR-4459 | 2.31E-01 | 1.00E+00 | 6.94E-01 | 0.502 (0.445; 0.55) | 0.037 |
| hsa-miR-744-5p | 2.33E-01 | 1.00E+00 | 4.13E-01 | 0.522 (0.47; 0.574) | -0.028 |
| hsa-miR-103a-2-5p | 2.40E-01 | 1.00E+00 | 6.81E-01 | 0.523 (0.471; 0.575) | 0.030 |
| hsa-miR-6767-5p | 2.41E-01 | 1.00E+00 | 9.10E-01 | 0.528 (0.476; 0.581) | 0.028 |
| hsa-miR-378a-3p | 2.41E-01 | 1.00E+00 | 1.50E-02 | 0.552 (0.5; 0.604) | -0.029 |
| hsa-miR-30b-5p | 2.42E-01 | 1.00E+00 | 7.05E-02 | 0.564 (0.513; 0.616) | 0.030 |
| hsa-miR-550a-5p | 2.45E-01 | 1.00E+00 | 1.17E-01 | 0.564 (0.512; 0.616) | 0.020 |
| hsa-miR-4659a-3p | 2.45E-01 | 1.00E+00 | 8.94E-01 | 0.502 (0.45; 0.554) | -0.042 |
| hsa-miR-6813-3p | 2.51E-01 | 1.00E+00 | 2.01E-01 | 0.529 (0.476; 0.581) | 0.020 |
| hsa-miR-4721 | 2.52E-01 | 1.00E+00 | 8.40E-01 | 0.528 (0.476; 0.581) | 0.042 |
| hsa-miR-4449 | 2.56E-01 | 1.00E+00 | 3.70E-01 | 0.517 (0.465; 0.569) | 0.028 |
| hsa-miR-4769-3p | 2.58E-01 | 1.00E+00 | 2.52E-01 | 0.535 (0.483; 0.587) | 0.018 |
| hsa-miR-500a-5p | 2.59E-01 | 1.00E+00 | 4.01E-02 | 0.539 (0.487; 0.591) | -0.031 |
| hsa-let-7b-3p | 2.61E-01 | 1.00E+00 | 1.49E-01 | 0.555 (0.503; 0.607) | 0.021 |
| hsa-miR-6812-3p | 2.66E-01 | 1.00E+00 | 2.35E-01 | 0.54 (0.488; 0.592) | 0.016 |
| hsa-miR-1238-3p | 2.76E-01 | 1.00E+00 | 2.16E-01 | 0.536 (0.484; 0.588) | 0.024 |
| hsa-miR-4324 | 2.81E-01 | 1.00E+00 | 2.80E-01 | 0.532 (0.48; 0.584) | 0.018 |
| hsa-miR-191-3p | 2.88E-01 | 1.00E+00 | 2.51E-01 | 0.533 (0.481; 0.585) | 0.022 |
| hsa-miR-937-5p | 2.90E-01 | 1.00E+00 | 2.32E-01 | 0.528 (0.476; 0.58) | -0.026 |
| hsa-miR-4443 | 2.92E-01 | 1.00E+00 | 7.74E-01 | 0.507 (0.441; 0.546) | 0.036 |
| hsa-miR-331-3p | 2.93E-01 | 1.00E+00 | 8.88E-01 | 0.514 (0.434; 0.538) | -0.035 |
| hsa-miR-4713-3p | 2.96E-01 | 1.00E+00 | 9.23E-01 | 0.539 (0.487; 0.591) | 0.030 |
| hsa-miR-4299 | 3.00E-01 | 1.00E+00 | 5.82E-01 | 0.523 (0.471; 0.575) | -0.030 |
| hsa-miR-193a-5p | 3.04E-01 | 1.00E+00 | 5.23E-01 | 0.548 (0.496; 0.6) | 0.022 |
| hsa-miR-628-5p | 3.05E-01 | 1.00E+00 | 1.49E-01 | 0.511 (0.459; 0.563) | 0.026 |
| hsa-miR-6727-5p | 3.07E-01 | 1.00E+00 | 2.33E-01 | 0.527 (0.475; 0.579) | -0.032 |
| hsa-miR-1288-3p | 3.10E-01 | 1.00E+00 | 9.52E-01 | 0.526 (0.473; 0.578) | 0.025 |
| hsa-miR-1237-3p | 3.15E-01 | 1.00E+00 | 2.87E-01 | 0.547 (0.495; 0.599) | 0.015 |
| hsa-miR-6797-3p | 3.18E-01 | 1.00E+00 | 2.91E-01 | 0.533 (0.481; 0.585) | 0.020 |
| hsa-miR-30a-5p | 3.23E-01 | 1.00E+00 | 5.40E-01 | 0.571 (0.519; 0.623) | 0.034 |
| hsa-miR-6800-3p | 3.24E-01 | 1.00E+00 | 2.34E-01 | 0.539 (0.487; 0.591) | 0.021 |
| hsa-let-7e-5p | 3.29E-01 | 1.00E+00 | 8.35E-01 | 0.528 (0.475; 0.58) | -0.031 |
| hsa-miR-132-3p | 3.30E-01 | 1.00E+00 | 1.10E-01 | 0.542 (0.49; 0.594) | -0.025 |
| hsa-miR-4728-5p | 3.30E-01 | 1.00E+00 | 8.10E-01 | 0.531 (0.479; 0.583) | 0.028 |
| hsa-miR-223-5p | 3.32E-01 | 1.00E+00 | 4.24E-01 | 0.52 (0.428; 0.532) | 0.021 |
| hsa-miR-5006-5p | 3.32E-01 | 1.00E+00 | 7.97E-01 | 0.529 (0.477; 0.581) | 0.026 |
| hsa-miR-3196 | 3.33E-01 | 1.00E+00 | 2.06E-01 | 0.521 (0.469; 0.573) | -0.024 |
| hsa-miR-340-5p | 3.38E-01 | 1.00E+00 | 8.71E-01 | 0.519 (0.467; 0.572) | 0.045 |
| hsa-miR-148b-3p | 3.39E-01 | 1.00E+00 | 8.23E-01 | 0.512 (0.46; 0.564) | -0.031 |
| hsa-miR-7847-3p | 3.39E-01 | 1.00E+00 | 9.97E-01 | 0.52 (0.468; 0.572) | 0.027 |
| hsa-miR-6785-3p | 3.41E-01 | 1.00E+00 | 3.50E-01 | 0.531 (0.479; 0.584) | 0.014 |
| hsa-miR-320a | 3.47E-01 | 1.00E+00 | 7.66E-02 | 0.519 (0.466; 0.571) | -0.030 |
| hsa-miR-1273g-3p | 3.48E-01 | 1.00E+00 | 9.36E-01 | 0.526 (0.474; 0.578) | 0.035 |
| hsa-miR-324-5p | 3.53E-01 | 1.00E+00 | 5.05E-01 | 0.531 (0.479; 0.583) | -0.021 |
| hsa-miR-6763-5p | 3.54E-01 | 1.00E+00 | 8.19E-01 | 0.506 (0.454; 0.558) | 0.025 |
| hsa-miR-4530 | 3.55E-01 | 1.00E+00 | 1.73E-01 | 0.531 (0.479; 0.583) | -0.027 |
| hsa-miR-4323 | 3.60E-01 | 1.00E+00 | 3.44E-01 | 0.532 (0.48; 0.584) | -0.028 |
| hsa-miR-26b-3p | 3.62E-01 | 1.00E+00 | 8.23E-02 | 0.527 (0.475; 0.579) | 0.017 |
| hsa-miR-3200-3p | 3.67E-01 | 1.00E+00 | 8.32E-01 | 0.5 (0.448; 0.553) | -0.029 |
| hsa-miR-1227-3p | 3.69E-01 | 1.00E+00 | 1.99E-01 | 0.552 (0.5; 0.604) | 0.009 |
| hsa-miR-627-5p | 3.74E-01 | 1.00E+00 | 2.73E-01 | 0.514 (0.462; 0.567) | -0.029 |
| hsa-miR-4442 | 3.76E-01 | 1.00E+00 | 8.19E-01 | 0.523 (0.471; 0.576) | 0.025 |
| hsa-miR-6865-3p | 3.77E-01 | 1.00E+00 | 2.62E-01 | 0.534 (0.482; 0.586) | 0.018 |
| hsa-miR-197-3p | 3.78E-01 | 1.00E+00 | 3.72E-01 | 0.533 (0.481; 0.585) | 0.023 |
| hsa-miR-8063 | 3.89E-01 | 1.00E+00 | 9.25E-01 | 0.508 (0.455; 0.56) | 0.025 |
| hsa-miR-4716-3p | 3.90E-01 | 1.00E+00 | 7.04E-01 | 0.525 (0.423; 0.527) | 0.023 |
| hsa-miR-106b-5p | 3.92E-01 | 1.00E+00 | 4.58E-02 | 0.536 (0.484; 0.588) | -0.034 |
| hsa-miR-3605-3p | 3.92E-01 | 1.00E+00 | 7.88E-01 | 0.501 (0.449; 0.554) | -0.017 |
| hsa-miR-6752-3p | 3.98E-01 | 1.00E+00 | 2.80E-01 | 0.537 (0.485; 0.589) | 0.013 |
| hsa-miR-183-5p | 4.02E-01 | 1.00E+00 | 4.86E-01 | 0.531 (0.479; 0.583) | -0.032 |
| hsa-miR-30e-5p | 4.03E-01 | 1.00E+00 | 2.43E-01 | 0.528 (0.476; 0.58) | -0.026 |
| hsa-miR-6760-3p | 4.05E-01 | 1.00E+00 | 3.17E-01 | 0.52 (0.468; 0.572) | 0.012 |
| hsa-miR-1305 | 4.05E-01 | 1.00E+00 | 7.28E-01 | 0.523 (0.425; 0.53) | 0.021 |
| hsa-miR-148a-3p | 4.05E-01 | 1.00E+00 | 2.27E-01 | 0.527 (0.474; 0.579) | -0.039 |
| hsa-miR-151a-3p | 4.11E-01 | 1.00E+00 | 5.33E-01 | 0.543 (0.491; 0.595) | 0.025 |
| hsa-miR-454-5p | 4.12E-01 | 1.00E+00 | 9.44E-01 | 0.51 (0.438; 0.542) | -0.022 |
| hsa-miR-26a-5p | 4.16E-01 | 1.00E+00 | 2.69E-01 | 0.532 (0.48; 0.584) | 0.023 |
| hsa-miR-4741 | 4.16E-01 | 1.00E+00 | 9.80E-01 | 0.522 (0.47; 0.574) | 0.027 |
| hsa-miR-374b-5p | 4.18E-01 | 1.00E+00 | 8.78E-01 | 0.514 (0.462; 0.567) | -0.032 |
| hsa-miR-424-5p | 4.18E-01 | 1.00E+00 | 2.13E-01 | 0.542 (0.49; 0.594) | -0.042 |
| hsa-miR-378a-5p | 4.25E-01 | 1.00E+00 | 3.08E-01 | 0.561 (0.509; 0.613) | 0.028 |
| hsa-miR-933 | 4.27E-01 | 1.00E+00 | 2.77E-01 | 0.527 (0.475; 0.579) | 0.010 |
| hsa-miR-1914-3p | 4.37E-01 | 1.00E+00 | 7.42E-01 | 0.529 (0.477; 0.581) | 0.025 |
| hsa-miR-5088-5p | 4.44E-01 | 1.00E+00 | 6.39E-01 | 0.521 (0.469; 0.574) | 0.019 |
| hsa-miR-1249-3p | 4.44E-01 | 1.00E+00 | 9.24E-01 | 0.513 (0.461; 0.566) | 0.012 |
| hsa-miR-182-5p | 4.58E-01 | 1.00E+00 | 4.58E-01 | 0.52 (0.468; 0.572) | -0.030 |
| hsa-miR-425-3p | 4.59E-01 | 1.00E+00 | 3.48E-01 | 0.547 (0.495; 0.599) | 0.013 |
| hsa-miR-3180-3p | 4.64E-01 | 1.00E+00 | 1.88E-01 | 0.51 (0.458; 0.562) | -0.030 |
| hsa-miR-365a-3p | 4.67E-01 | 1.00E+00 | 4.81E-01 | 0.53 (0.477; 0.582) | 0.022 |
| hsa-miR-485-3p | 4.71E-01 | 1.00E+00 | 3.58E-01 | 0.501 (0.448; 0.553) | -0.031 |
| hsa-miR-550a-3p | 4.73E-01 | 1.00E+00 | 1.97E-01 | 0.557 (0.506; 0.609) | 0.018 |
| hsa-miR-24-3p | 4.76E-01 | 1.00E+00 | 6.23E-01 | 0.509 (0.457; 0.561) | -0.014 |
| hsa-miR-339-3p | 4.87E-01 | 1.00E+00 | 7.42E-01 | 0.509 (0.457; 0.561) | 0.016 |
| hsa-miR-151a-5p | 4.92E-01 | 1.00E+00 | 7.65E-01 | 0.515 (0.433; 0.538) | -0.016 |
| hsa-miR-181a-5p | 4.94E-01 | 1.00E+00 | 1.45E-01 | 0.506 (0.454; 0.559) | -0.031 |
| hsa-miR-638 | 4.96E-01 | 1.00E+00 | 3.73E-01 | 0.522 (0.47; 0.574) | -0.015 |
| hsa-miR-148b-5p | 4.99E-01 | 1.00E+00 | 9.85E-01 | 0.523 (0.471; 0.575) | -0.018 |
| hsa-miR-6851-3p | 5.01E-01 | 1.00E+00 | 3.29E-01 | 0.534 (0.482; 0.586) | 0.012 |
| hsa-miR-6734-5p | 5.01E-01 | 1.00E+00 | 5.29E-01 | 0.521 (0.469; 0.573) | 0.016 |
| hsa-miR-133b | 5.03E-01 | 1.00E+00 | 1.03E-01 | 0.554 (0.501; 0.606) | 0.035 |
| hsa-miR-6821-5p | 5.05E-01 | 1.00E+00 | 8.19E-01 | 0.505 (0.453; 0.558) | 0.014 |
| hsa-miR-1268a | 5.13E-01 | 1.00E+00 | 8.76E-01 | 0.528 (0.42; 0.524) | 0.020 |
| hsa-miR-4291 | 5.34E-01 | 1.00E+00 | 5.27E-01 | 0.527 (0.475; 0.579) | -0.018 |
| hsa-miR-6087 | 5.39E-01 | 1.00E+00 | 9.83E-01 | 0.51 (0.438; 0.543) | 0.014 |
| hsa-miR-6126 | 5.45E-01 | 1.00E+00 | 2.91E-01 | 0.508 (0.439; 0.544) | -0.017 |
| hsa-miR-140-3p | 5.49E-01 | 1.00E+00 | 5.18E-01 | 0.551 (0.499; 0.603) | -0.013 |
| hsa-miR-6889-3p | 5.54E-01 | 1.00E+00 | 3.82E-01 | 0.52 (0.468; 0.573) | 0.012 |
| hsa-miR-330-3p | 5.55E-01 | 1.00E+00 | 8.48E-01 | 0.517 (0.431; 0.535) | -0.015 |
| hsa-miR-1275 | 5.59E-01 | 1.00E+00 | 7.92E-02 | 0.514 (0.462; 0.566) | 0.019 |
| hsa-miR-181b-5p | 5.59E-01 | 1.00E+00 | 5.51E-01 | 0.527 (0.474; 0.579) | 0.019 |
| hsa-miR-362-3p | 5.61E-01 | 1.00E+00 | 9.47E-01 | 0.512 (0.436; 0.54) | -0.029 |
| hsa-miR-340-3p | 5.70E-01 | 1.00E+00 | 1.25E-01 | 0.547 (0.495; 0.599) | 0.030 |
| hsa-miR-4317 | 5.71E-01 | 1.00E+00 | 2.74E-01 | 0.535 (0.483; 0.587) | 0.015 |
| hsa-miR-6798-3p | 5.91E-01 | 1.00E+00 | 8.50E-01 | 0.518 (0.466; 0.571) | -0.005 |
| hsa-miR-146b-5p | 6.00E-01 | 1.00E+00 | 8.05E-02 | 0.517 (0.465; 0.569) | -0.025 |
| hsa-miR-23b-3p | 6.04E-01 | 1.00E+00 | 4.36E-01 | 0.528 (0.42; 0.524) | -0.016 |
| hsa-miR-6515-3p | 6.09E-01 | 1.00E+00 | 5.58E-01 | 0.522 (0.47; 0.574) | 0.008 |
| hsa-miR-6780a-5p | 6.11E-01 | 1.00E+00 | 7.11E-01 | 0.518 (0.43; 0.534) | 0.012 |
| hsa-miR-181a-2-3p | 6.13E-01 | 1.00E+00 | 4.00E-01 | 0.534 (0.482; 0.586) | 0.010 |
| hsa-miR-361-5p | 6.14E-01 | 1.00E+00 | 7.33E-02 | 0.546 (0.494; 0.598) | 0.012 |
| hsa-let-7b-5p | 6.29E-01 | 1.00E+00 | 9.63E-02 | 0.531 (0.479; 0.583) | -0.021 |
| hsa-miR-328-3p | 6.32E-01 | 1.00E+00 | 8.70E-01 | 0.502 (0.45; 0.555) | -0.013 |
| hsa-miR-99a-5p | 6.45E-01 | 1.00E+00 | 5.08E-01 | 0.505 (0.453; 0.557) | 0.021 |
| hsa-miR-3125 | 6.48E-01 | 1.00E+00 | 5.66E-01 | 0.514 (0.434; 0.538) | 0.011 |
| hsa-miR-6791-5p | 6.58E-01 | 1.00E+00 | 8.55E-01 | 0.503 (0.451; 0.555) | 0.012 |
| hsa-miR-19b-3p | 6.62E-01 | 1.00E+00 | 7.66E-01 | 0.515 (0.463; 0.567) | 0.014 |
| hsa-miR-139-3p | 6.67E-01 | 1.00E+00 | 3.91E-01 | 0.502 (0.446; 0.55) | -0.006 |
| hsa-miR-4313 | 6.71E-01 | 1.00E+00 | 5.15E-01 | 0.534 (0.481; 0.586) | 0.010 |
| hsa-miR-22-3p | 6.72E-01 | 1.00E+00 | 2.23E-01 | 0.502 (0.445; 0.55) | 0.010 |
| hsa-miR-199a-5p | 6.72E-01 | 1.00E+00 | 2.89E-01 | 0.521 (0.426; 0.531) | -0.020 |
| hsa-miR-4466 | 6.74E-01 | 1.00E+00 | 4.10E-01 | 0.505 (0.453; 0.558) | -0.010 |
| hsa-miR-766-3p | 6.74E-01 | 1.00E+00 | 3.24E-01 | 0.526 (0.474; 0.578) | -0.012 |
| hsa-miR-6513-3p | 6.75E-01 | 1.00E+00 | 7.27E-01 | 0.503 (0.451; 0.555) | -0.012 |
| hsa-miR-6724-5p | 6.78E-01 | 1.00E+00 | 4.93E-01 | 0.526 (0.474; 0.578) | -0.011 |
| hsa-miR-564 | 6.85E-01 | 1.00E+00 | 6.73E-01 | 0.531 (0.479; 0.583) | 0.009 |
| hsa-miR-1255b-5p | 6.87E-01 | 1.00E+00 | 7.90E-01 | 0.506 (0.454; 0.559) | 0.010 |
| hsa-miR-3960 | 6.87E-01 | 1.00E+00 | 7.68E-01 | 0.509 (0.438; 0.543) | 0.011 |
| hsa-miR-146a-5p | 6.90E-01 | 1.00E+00 | 1.32E-01 | 0.528 (0.476; 0.58) | 0.011 |
| hsa-miR-27a-3p | 7.00E-01 | 1.00E+00 | 6.38E-01 | 0.504 (0.452; 0.556) | -0.017 |
| hsa-miR-5581-5p | 7.03E-01 | 1.00E+00 | 4.76E-01 | 0.512 (0.46; 0.565) | 0.010 |
| hsa-miR-152-3p | 7.07E-01 | 1.00E+00 | 9.36E-01 | 0.529 (0.477; 0.582) | -0.008 |
| hsa-miR-491-5p | 7.07E-01 | 1.00E+00 | 5.51E-01 | 0.51 (0.457; 0.562) | 0.002 |
| hsa-miR-192-5p | 7.08E-01 | 1.00E+00 | 6.51E-01 | 0.541 (0.489; 0.593) | 0.013 |
| hsa-miR-641 | 7.09E-01 | 1.00E+00 | 9.26E-01 | 0.522 (0.47; 0.574) | -0.007 |
| hsa-miR-30c-5p | 7.17E-01 | 1.00E+00 | 6.36E-01 | 0.561 (0.51; 0.613) | 0.009 |
| hsa-miR-4497 | 7.17E-01 | 1.00E+00 | 5.51E-01 | 0.521 (0.469; 0.573) | -0.009 |
| hsa-miR-425-5p | 7.21E-01 | 1.00E+00 | 7.24E-01 | 0.554 (0.503; 0.606) | 0.007 |
| hsa-miR-7108-5p | 7.24E-01 | 1.00E+00 | 9.12E-01 | 0.521 (0.469; 0.573) | 0.010 |
| hsa-miR-130b-3p | 7.29E-01 | 1.00E+00 | 4.10E-01 | 0.525 (0.473; 0.577) | -0.008 |
| hsa-miR-3940-5p | 7.32E-01 | 1.00E+00 | 8.18E-01 | 0.502 (0.45; 0.554) | -0.007 |
| hsa-miR-28-5p | 7.33E-01 | 1.00E+00 | 2.27E-01 | 0.517 (0.431; 0.535) | 0.010 |
| hsa-miR-339-5p | 7.34E-01 | 1.00E+00 | 9.60E-01 | 0.504 (0.452; 0.556) | 0.012 |
| hsa-miR-6512-5p | 7.34E-01 | 1.00E+00 | 6.43E-01 | 0.527 (0.475; 0.579) | 0.008 |
| hsa-miR-874-3p | 7.35E-01 | 1.00E+00 | 7.37E-01 | 0.522 (0.47; 0.574) | -0.006 |
| hsa-miR-151b | 7.48E-01 | 1.00E+00 | 5.23E-01 | 0.516 (0.464; 0.568) | 0.007 |
| hsa-miR-654-3p | 7.54E-01 | 1.00E+00 | 1.71E-01 | 0.511 (0.437; 0.541) | -0.017 |
| hsa-miR-29c-3p | 7.55E-01 | 1.00E+00 | 3.54E-01 | 0.51 (0.458; 0.562) | 0.014 |
| hsa-miR-1271-5p | 7.73E-01 | 1.00E+00 | 4.63E-02 | 0.548 (0.496; 0.599) | 0.010 |
| hsa-miR-7-1-3p | 7.73E-01 | 1.00E+00 | 4.71E-01 | 0.531 (0.417; 0.521) | -0.013 |
| hsa-miR-139-5p | 7.77E-01 | 1.00E+00 | 1.92E-01 | 0.528 (0.476; 0.58) | 0.007 |
| hsa-miR-30c-1-3p | 7.77E-01 | 1.00E+00 | 3.24E-01 | 0.537 (0.484; 0.589) | 0.002 |
| hsa-miR-335-5p | 7.82E-01 | 1.00E+00 | 3.24E-01 | 0.542 (0.49; 0.594) | 0.011 |
| hsa-miR-128-3p | 7.85E-01 | 1.00E+00 | 3.20E-01 | 0.557 (0.506; 0.609) | 0.008 |
| hsa-miR-6757-5p | 7.88E-01 | 1.00E+00 | 6.87E-01 | 0.509 (0.439; 0.544) | 0.005 |
| hsa-miR-7975 | 7.99E-01 | 1.00E+00 | 2.33E-01 | 0.505 (0.453; 0.557) | 0.007 |
| hsa-miR-129-2-3p | 8.05E-01 | 1.00E+00 | 4.61E-01 | 0.524 (0.471; 0.576) | -0.005 |
| hsa-miR-191-5p | 8.14E-01 | 1.00E+00 | 6.91E-01 | 0.56 (0.508; 0.612) | 0.005 |
| hsa-miR-16-2-3p | 8.16E-01 | 1.00E+00 | 1.08E-01 | 0.522 (0.47; 0.574) | -0.008 |
| hsa-miR-6089 | 8.20E-01 | 1.00E+00 | 9.30E-01 | 0.516 (0.464; 0.568) | 0.006 |
| hsa-miR-4788 | 8.28E-01 | 1.00E+00 | 3.93E-01 | 0.511 (0.459; 0.563) | -0.009 |
| hsa-miR-142-3p | 8.29E-01 | 1.00E+00 | 3.02E-01 | 0.512 (0.459; 0.565) | 0.011 |
| hsa-miR-7152-3p | 8.45E-01 | 1.00E+00 | 3.71E-01 | 0.505 (0.453; 0.557) | -0.006 |
| hsa-miR-5690 | 8.49E-01 | 1.00E+00 | 5.04E-01 | 0.505 (0.453; 0.557) | -0.009 |
| hsa-miR-1268b | 8.61E-01 | 1.00E+00 | 7.53E-01 | 0.509 (0.457; 0.562) | 0.005 |
| hsa-miR-7114-5p | 8.63E-01 | 1.00E+00 | 1.94E-01 | 0.509 (0.457; 0.561) | 0.007 |
| hsa-miR-6880-3p | 8.65E-01 | 1.00E+00 | 7.26E-01 | 0.515 (0.463; 0.567) | 0.003 |
| hsa-miR-6800-5p | 8.75E-01 | 1.00E+00 | 8.65E-01 | 0.5 (0.448; 0.552) | 0.004 |
| hsa-miR-409-3p | 8.82E-01 | 1.00E+00 | 2.58E-01 | 0.503 (0.445; 0.549) | -0.009 |
| hsa-miR-6869-5p | 8.86E-01 | 1.00E+00 | 2.17E-01 | 0.508 (0.455; 0.56) | -0.004 |
| hsa-miR-15b-3p | 8.88E-01 | 1.00E+00 | 5.14E-01 | 0.509 (0.456; 0.561) | -0.004 |
| hsa-miR-29b-3p | 9.02E-01 | 1.00E+00 | 2.97E-02 | 0.543 (0.491; 0.595) | -0.005 |
| hsa-miR-3614-5p | 9.29E-01 | 1.00E+00 | 7.77E-01 | 0.526 (0.474; 0.578) | -0.001 |
| hsa-miR-25-3p | 9.33E-01 | 1.00E+00 | 7.81E-01 | 0.516 (0.463; 0.569) | -0.001 |
| hsa-miR-574-3p | 9.44E-01 | 1.00E+00 | 5.71E-01 | 0.51 (0.458; 0.562) | -0.002 |
| hsa-miR-10a-5p | 9.47E-01 | 1.00E+00 | 3.39E-02 | 0.513 (0.435; 0.539) | -0.003 |
| hsa-miR-185-5p | 9.52E-01 | 1.00E+00 | 3.24E-01 | 0.506 (0.454; 0.558) | 0.001 |
| hsa-miR-664b-3p | 9.52E-01 | 1.00E+00 | 4.82E-01 | 0.516 (0.464; 0.569) | -0.001 |
| hsa-miR-4787-5p | 9.57E-01 | 1.00E+00 | 8.34E-01 | 0.505 (0.453; 0.558) | -0.001 |
| hsa-miR-215-5p | 9.59E-01 | 1.00E+00 | 8.43E-01 | 0.526 (0.474; 0.578) | 0.002 |
| hsa-miR-194-5p | 9.61E-01 | 1.00E+00 | 4.17E-01 | 0.508 (0.455; 0.56) | -0.002 |
| hsa-miR-495-3p | 9.67E-01 | 1.00E+00 | 1.13E-01 | 0.511 (0.459; 0.563) | -0.002 |
| hsa-miR-4653-3p | 9.69E-01 | 1.00E+00 | 3.94E-01 | 0.501 (0.446; 0.551) | -0.001 |
| hsa-miR-6848-3p | 9.82E-01 | 1.00E+00 | 8.59E-01 | 0.502 (0.45; 0.554) | 0.000 |
| hsa-miR-3613-3p | 9.87E-01 | 1.00E+00 | 7.76E-01 | 0.511 (0.459; 0.564) | -0.000 |
| hsa-miR-26b-5p | 9.90E-01 | 1.00E+00 | 3.13E-01 | 0.523 (0.471; 0.575) | 0.001 |

**Table 6: Result metrics for the comparison of CAD versus control**

| miRNA | ttest_rawp | ttest_adjp | glm_rawp | AUC | log2FoldChange |
| --- | --- | --- | --- | --- | --- |
| hsa-miR-454-3p | 6.49E-45 | 2.83E-42 | 1.13E-25 | 0.71 (0.677; 0.743) | -0.716 |
| hsa-miR-19a-3p | 1.08E-44 | 4.72E-42 | 1.11E-29 | 0.757 (0.726; 0.788) | -0.598 |
| hsa-miR-374a-5p | 4.25E-38 | 1.85E-35 | 6.16E-24 | 0.712 (0.679; 0.745) | -0.793 |
| hsa-miR-7641 | 4.85E-37 | 2.10E-34 | 3.24E-20 | 0.688 (0.654; 0.722) | 0.505 |
| hsa-miR-301a-3p | 8.74E-36 | 3.78E-33 | 2.71E-21 | 0.7 (0.667; 0.734) | -0.550 |
| hsa-miR-374b-5p | 6.71E-35 | 2.89E-32 | 1.89E-23 | 0.714 (0.68; 0.747) | -0.534 |
| hsa-miR-6090 | 2.17E-34 | 9.34E-32 | 1.54E-21 | 0.697 (0.664; 0.731) | 0.281 |
| hsa-miR-195-5p | 4.06E-34 | 1.74E-31 | 1.29E-22 | 0.68 (0.646; 0.714) | -0.487 |
| hsa-miR-199a-3p | 1.07E-32 | 4.60E-30 | 1.93E-14 | 0.656 (0.621; 0.691) | -0.565 |
| hsa-miR-6875-5p | 1.23E-32 | 5.26E-30 | 4.25E-19 | 0.688 (0.654; 0.722) | 0.361 |
| hsa-miR-6087 | 2.15E-32 | 9.16E-30 | 5.81E-25 | 0.712 (0.679; 0.745) | 0.304 |
| hsa-miR-15a-5p | 6.53E-32 | 2.78E-29 | 4.32E-22 | 0.716 (0.683; 0.749) | -0.456 |
| hsa-miR-660-5p | 7.44E-32 | 3.15E-29 | 4.28E-21 | 0.718 (0.685; 0.751) | -0.439 |
| hsa-miR-6127 | 7.80E-32 | 3.30E-29 | 1.09E-17 | 0.676 (0.642; 0.711) | 0.323 |
| hsa-miR-101-3p | 2.86E-31 | 1.20E-28 | 1.81E-24 | 0.72 (0.687; 0.753) | -0.682 |
| hsa-miR-98-5p | 7.59E-31 | 3.20E-28 | 3.95E-19 | 0.672 (0.638; 0.707) | -0.404 |
| hsa-miR-424-5p | 2.36E-30 | 9.92E-28 | 3.59E-22 | 0.699 (0.665; 0.733) | -0.535 |
| hsa-miR-6089 | 3.46E-30 | 1.45E-27 | 7.23E-23 | 0.691 (0.657; 0.725) | 0.347 |
| hsa-miR-4443 | 3.57E-30 | 1.49E-27 | 4.11E-18 | 0.663 (0.628; 0.698) | 0.400 |
| hsa-miR-126-3p | 4.30E-30 | 1.79E-27 | 3.24E-20 | 0.687 (0.653; 0.721) | -0.529 |
| hsa-miR-6088 | 1.04E-29 | 4.31E-27 | 2.46E-21 | 0.698 (0.665; 0.732) | 0.232 |
| hsa-miR-17-5p | 1.91E-29 | 7.93E-27 | 1.03E-19 | 0.692 (0.657; 0.726) | -0.458 |
| hsa-miR-196b-5p | 7.19E-29 | 2.98E-26 | 2.13E-12 | 0.646 (0.611; 0.681) | -0.395 |
| hsa-miR-20a-5p | 8.58E-29 | 3.54E-26 | 1.49E-16 | 0.679 (0.645; 0.713) | -0.494 |
| hsa-miR-4291 | 1.88E-28 | 7.76E-26 | 1.21E-18 | 0.697 (0.663; 0.731) | -0.346 |
| hsa-miR-629-3p | 1.96E-28 | 8.05E-26 | 2.15E-20 | 0.651 (0.616; 0.686) | -0.266 |
| hsa-miR-93-5p | 2.20E-28 | 9.03E-26 | 3.71E-19 | 0.694 (0.66; 0.728) | -0.448 |
| hsa-miR-18a-5p | 2.71E-28 | 1.11E-25 | 1.08E-18 | 0.697 (0.663; 0.731) | -0.576 |
| hsa-miR-20b-5p | 6.30E-28 | 2.57E-25 | 3.75E-17 | 0.684 (0.649; 0.718) | -0.430 |
| hsa-miR-6800-5p | 1.48E-27 | 6.01E-25 | 5.97E-20 | 0.675 (0.64; 0.709) | 0.263 |
| hsa-miR-6869-5p | 2.28E-27 | 9.28E-25 | 2.76E-21 | 0.697 (0.663; 0.73) | 0.397 |
| hsa-miR-4507 | 4.65E-27 | 1.88E-24 | 2.18E-19 | 0.672 (0.638; 0.706) | 0.374 |
| hsa-miR-6125 | 5.16E-27 | 2.08E-24 | 8.36E-21 | 0.673 (0.638; 0.707) | 0.294 |
| hsa-miR-4516 | 6.95E-27 | 2.80E-24 | 1.11E-16 | 0.655 (0.62; 0.69) | 0.308 |
| hsa-miR-1285-3p | 1.71E-26 | 6.86E-24 | 5.81E-21 | 0.66 (0.625; 0.695) | -0.201 |
| hsa-miR-3960 | 2.85E-26 | 1.14E-23 | 9.38E-20 | 0.688 (0.654; 0.722) | 0.288 |
| hsa-miR-502-5p | 3.80E-26 | 1.52E-23 | 3.74E-17 | 0.656 (0.621; 0.691) | -0.392 |
| hsa-miR-200c-3p | 5.15E-26 | 2.05E-23 | 1.77E-18 | 0.688 (0.654; 0.722) | -0.218 |
| hsa-miR-96-5p | 7.50E-26 | 2.99E-23 | 2.39E-17 | 0.674 (0.64; 0.709) | -0.496 |
| hsa-miR-4787-5p | 1.06E-25 | 4.21E-23 | 1.07E-19 | 0.688 (0.654; 0.722) | 0.178 |
| hsa-miR-3162-5p | 1.46E-25 | 5.79E-23 | 1.32E-17 | 0.648 (0.613; 0.684) | 0.286 |
| hsa-miR-93-3p | 1.86E-25 | 7.36E-23 | 7.17E-18 | 0.666 (0.631; 0.701) | -0.291 |
| hsa-miR-652-3p | 2.04E-25 | 8.02E-23 | 1.92E-20 | 0.694 (0.659; 0.728) | -0.277 |
| hsa-miR-6821-5p | 3.36E-25 | 1.32E-22 | 2.00E-15 | 0.656 (0.621; 0.691) | 0.206 |
| hsa-miR-144-3p | 3.63E-25 | 1.42E-22 | 4.28E-18 | 0.685 (0.65; 0.719) | -0.750 |
| hsa-miR-4485-5p | 4.19E-25 | 1.64E-22 | 2.06E-18 | 0.678 (0.644; 0.712) | 0.385 |
| hsa-miR-4530 | 6.06E-25 | 2.36E-22 | 7.44E-19 | 0.674 (0.639; 0.708) | 0.334 |
| hsa-miR-18b-5p | 6.93E-25 | 2.70E-22 | 7.21E-17 | 0.681 (0.647; 0.715) | -0.469 |
| hsa-miR-27b-3p | 9.84E-25 | 3.82E-22 | 8.65E-16 | 0.647 (0.611; 0.682) | -0.493 |
| hsa-miR-148b-3p | 2.12E-24 | 8.22E-22 | 9.81E-17 | 0.662 (0.628; 0.697) | -0.361 |
| hsa-miR-126-5p | 3.19E-24 | 1.23E-21 | 3.38E-13 | 0.652 (0.617; 0.687) | -0.347 |
| hsa-miR-4687-3p | 3.34E-24 | 1.28E-21 | 2.67E-18 | 0.69 (0.656; 0.724) | 0.249 |
| hsa-miR-374c-5p | 3.91E-24 | 1.50E-21 | 3.32E-17 | 0.669 (0.634; 0.703) | -0.420 |
| hsa-miR-139-3p | 4.93E-24 | 1.89E-21 | 5.20E-11 | 0.637 (0.602; 0.673) | -0.125 |
| hsa-miR-4659a-3p | 1.07E-23 | 4.09E-21 | 1.59E-14 | 0.624 (0.589; 0.66) | -0.372 |
| hsa-miR-3651 | 1.15E-23 | 4.39E-21 | 2.77E-17 | 0.672 (0.637; 0.706) | -0.304 |
| hsa-miR-500b-5p | 1.34E-23 | 5.10E-21 | 3.29E-18 | 0.672 (0.638; 0.707) | -0.269 |
| hsa-miR-6513-3p | 1.46E-23 | 5.52E-21 | 5.62E-17 | 0.635 (0.6; 0.671) | -0.317 |
| hsa-miR-744-5p | 2.38E-23 | 9.01E-21 | 2.79E-19 | 0.694 (0.66; 0.728) | -0.264 |
| hsa-miR-130b-5p | 2.63E-23 | 9.93E-21 | 8.92E-16 | 0.637 (0.602; 0.673) | -0.180 |
| hsa-miR-4505 | 3.90E-23 | 1.47E-20 | 1.27E-15 | 0.647 (0.611; 0.682) | 0.250 |
| hsa-miR-215-5p | 4.01E-23 | 1.50E-20 | 2.64E-18 | 0.66 (0.626; 0.695) | -0.397 |
| hsa-miR-22-5p | 4.99E-23 | 1.87E-20 | 5.42E-20 | 0.67 (0.636; 0.705) | -0.315 |
| hsa-miR-16-5p | 5.03E-23 | 1.88E-20 | 1.97E-14 | 0.667 (0.632; 0.702) | -0.237 |
| hsa-miR-140-5p | 6.93E-23 | 2.58E-20 | 2.68E-14 | 0.674 (0.639; 0.708) | -0.383 |
| hsa-miR-6803-5p | 7.26E-23 | 2.69E-20 | 2.54E-16 | 0.654 (0.62; 0.689) | 0.373 |
| hsa-miR-454-5p | 1.08E-22 | 4.01E-20 | 3.75E-15 | 0.638 (0.602; 0.673) | -0.284 |
| hsa-miR-942-5p | 1.57E-22 | 5.81E-20 | 3.04E-17 | 0.648 (0.613; 0.683) | -0.362 |
| hsa-miR-624-5p | 2.25E-22 | 8.27E-20 | 8.44E-15 | 0.647 (0.612; 0.682) | -0.403 |
| hsa-miR-494-3p | 2.67E-22 | 9.80E-20 | 2.23E-15 | 0.671 (0.637; 0.706) | 0.511 |
| hsa-miR-6749-5p | 3.80E-22 | 1.39E-19 | 8.69E-14 | 0.649 (0.613; 0.685) | 0.434 |
| hsa-miR-21-5p | 4.78E-22 | 1.74E-19 | 1.85E-16 | 0.676 (0.642; 0.711) | -0.333 |
| hsa-miR-3200-5p | 5.08E-22 | 1.85E-19 | 7.48E-16 | 0.65 (0.615; 0.686) | -0.367 |
| hsa-miR-6780b-5p | 7.50E-22 | 2.72E-19 | 1.72E-12 | 0.67 (0.635; 0.704) | 0.302 |
| hsa-miR-17-3p | 1.26E-21 | 4.56E-19 | 1.57E-12 | 0.655 (0.62; 0.69) | -0.353 |
| hsa-miR-3163 | 1.66E-21 | 5.98E-19 | 3.09E-15 | 0.629 (0.594; 0.665) | -0.253 |
| hsa-miR-130a-3p | 2.08E-21 | 7.49E-19 | 1.64E-15 | 0.667 (0.632; 0.702) | -0.368 |
| hsa-miR-199a-5p | 2.38E-21 | 8.54E-19 | 3.47E-11 | 0.619 (0.584; 0.655) | -0.447 |
| hsa-let-7e-5p | 2.74E-21 | 9.81E-19 | 2.23E-13 | 0.635 (0.6; 0.671) | -0.280 |
| hsa-miR-4515 | 3.75E-21 | 1.34E-18 | 5.01E-14 | 0.676 (0.641; 0.711) | 0.207 |
| hsa-miR-28-5p | 4.14E-21 | 1.47E-18 | 1.79E-14 | 0.647 (0.612; 0.682) | -0.292 |
| hsa-miR-4318 | 4.49E-21 | 1.59E-18 | 5.07E-16 | 0.652 (0.617; 0.687) | -0.229 |
| hsa-miR-2861 | 5.39E-21 | 1.91E-18 | 3.27E-15 | 0.645 (0.609; 0.68) | 0.259 |
| hsa-miR-1273g-3p | 7.11E-21 | 2.51E-18 | 1.63E-11 | 0.634 (0.599; 0.67) | 0.318 |
| hsa-miR-4449 | 1.06E-20 | 3.72E-18 | 6.77E-15 | 0.643 (0.607; 0.678) | -0.220 |
| hsa-miR-1973 | 1.09E-20 | 3.82E-18 | 3.93E-15 | 0.675 (0.64; 0.709) | 0.332 |
| hsa-miR-183-5p | 1.26E-20 | 4.42E-18 | 3.48E-16 | 0.648 (0.613; 0.683) | -0.343 |
| hsa-miR-7704 | 1.43E-20 | 4.97E-18 | 9.00E-16 | 0.649 (0.614; 0.684) | 0.238 |
| hsa-miR-10a-5p | 1.48E-20 | 5.16E-18 | 3.38E-10 | 0.604 (0.568; 0.64) | -0.416 |
| hsa-miR-362-3p | 1.97E-20 | 6.84E-18 | 3.95E-14 | 0.642 (0.607; 0.677) | -0.447 |
| hsa-miR-550b-2-5p | 2.08E-20 | 7.21E-18 | 1.30E-12 | 0.627 (0.591; 0.663) | -0.212 |
| hsa-miR-148b-5p | 3.81E-20 | 1.31E-17 | 5.43E-15 | 0.645 (0.61; 0.681) | -0.246 |
| hsa-miR-7150 | 7.78E-20 | 2.67E-17 | 2.82E-14 | 0.694 (0.66; 0.728) | 0.300 |
| hsa-miR-4459 | 9.99E-20 | 3.43E-17 | 1.17E-15 | 0.664 (0.629; 0.699) | 0.329 |
| hsa-miR-27a-3p | 1.01E-19 | 3.45E-17 | 6.02E-13 | 0.649 (0.614; 0.685) | -0.356 |
| hsa-miR-7-5p | 1.04E-19 | 3.53E-17 | 6.53E-19 | 0.667 (0.632; 0.702) | -0.441 |
| hsa-miR-194-5p | 1.34E-19 | 4.54E-17 | 1.20E-17 | 0.661 (0.627; 0.696) | -0.334 |
| hsa-miR-6803-3p | 1.49E-19 | 5.07E-17 | 7.14E-18 | 0.656 (0.621; 0.69) | -0.153 |
| hsa-miR-192-5p | 1.82E-19 | 6.16E-17 | 6.93E-16 | 0.642 (0.607; 0.677) | -0.341 |
| hsa-miR-4741 | 2.29E-19 | 7.72E-17 | 5.28E-14 | 0.665 (0.63; 0.7) | 0.353 |
| hsa-miR-642b-3p | 5.18E-19 | 1.74E-16 | 1.54E-12 | 0.638 (0.603; 0.674) | 0.237 |
| hsa-miR-4323 | 7.19E-19 | 2.41E-16 | 5.69E-11 | 0.614 (0.577; 0.65) | -0.305 |
| hsa-miR-6511b-3p | 8.50E-19 | 2.84E-16 | 1.88E-16 | 0.64 (0.605; 0.676) | -0.210 |
| hsa-miR-6879-5p | 9.75E-19 | 3.25E-16 | 7.71E-10 | 0.633 (0.598; 0.669) | 0.225 |
| hsa-miR-664b-5p | 1.25E-18 | 4.15E-16 | 2.10E-12 | 0.637 (0.601; 0.672) | 0.522 |
| hsa-miR-30b-5p | 1.54E-18 | 5.09E-16 | 1.53E-12 | 0.639 (0.604; 0.675) | -0.240 |
| hsa-miR-7976 | 1.99E-18 | 6.56E-16 | 5.81E-13 | 0.619 (0.583; 0.654) | -0.140 |
| hsa-miR-144-5p | 2.17E-18 | 7.14E-16 | 2.71E-13 | 0.647 (0.611; 0.682) | -0.514 |
| hsa-let-7d-5p | 2.26E-18 | 7.40E-16 | 4.07E-13 | 0.627 (0.591; 0.662) | -0.210 |
| hsa-miR-532-3p | 3.13E-18 | 1.02E-15 | 6.79E-14 | 0.633 (0.598; 0.669) | -0.272 |
| hsa-miR-26a-5p | 3.19E-18 | 1.04E-15 | 8.58E-14 | 0.666 (0.631; 0.701) | -0.282 |
| hsa-miR-423-3p | 4.14E-18 | 1.34E-15 | 2.85E-14 | 0.633 (0.597; 0.668) | -0.286 |
| hsa-miR-132-3p | 4.51E-18 | 1.46E-15 | 1.58E-17 | 0.659 (0.625; 0.694) | -0.227 |
| hsa-miR-24-3p | 4.72E-18 | 1.53E-15 | 7.88E-14 | 0.657 (0.622; 0.692) | -0.178 |
| hsa-miR-642a-3p | 5.62E-18 | 1.81E-15 | 6.17E-14 | 0.67 (0.635; 0.705) | 0.304 |
| hsa-miR-326 | 5.97E-18 | 1.92E-15 | 1.41E-10 | 0.606 (0.57; 0.642) | -0.274 |
| hsa-miR-99a-5p | 5.99E-18 | 1.92E-15 | 1.77E-13 | 0.625 (0.589; 0.661) | -0.421 |
| hsa-miR-1255b-5p | 6.11E-18 | 1.95E-15 | 1.45E-15 | 0.643 (0.608; 0.679) | -0.203 |
| hsa-miR-340-3p | 6.80E-18 | 2.16E-15 | 7.78E-12 | 0.603 (0.567; 0.639) | -0.513 |
| hsa-miR-6512-5p | 8.81E-18 | 2.79E-15 | 7.86E-10 | 0.642 (0.606; 0.677) | 0.222 |
| hsa-miR-762 | 1.27E-17 | 4.00E-15 | 3.03E-12 | 0.657 (0.622; 0.692) | 0.234 |
| hsa-miR-7-1-3p | 1.50E-17 | 4.73E-15 | 2.41E-12 | 0.607 (0.571; 0.644) | -0.430 |
| hsa-miR-142-3p | 1.55E-17 | 4.86E-15 | 2.42E-13 | 0.64 (0.604; 0.676) | -0.429 |
| hsa-let-7f-5p | 2.01E-17 | 6.30E-15 | 5.44E-12 | 0.63 (0.595; 0.666) | -0.230 |
| hsa-miR-150-5p | 3.19E-17 | 9.95E-15 | 4.64E-05 | 0.599 (0.563; 0.635) | -0.300 |
| hsa-miR-342-5p | 3.47E-17 | 1.08E-14 | 1.09E-11 | 0.639 (0.604; 0.675) | -0.212 |
| hsa-miR-5194 | 8.20E-17 | 2.54E-14 | 5.65E-09 | 0.635 (0.599; 0.67) | 0.220 |
| hsa-miR-1225-5p | 1.10E-16 | 3.40E-14 | 1.89E-11 | 0.643 (0.608; 0.679) | 0.174 |
| hsa-miR-328-3p | 1.36E-16 | 4.19E-14 | 1.08E-13 | 0.629 (0.593; 0.665) | -0.224 |
| hsa-miR-152-3p | 1.75E-16 | 5.36E-14 | 4.07E-11 | 0.642 (0.606; 0.677) | -0.176 |
| hsa-miR-182-5p | 2.46E-16 | 7.52E-14 | 1.21E-13 | 0.641 (0.606; 0.676) | -0.350 |
| hsa-let-7g-5p | 3.06E-16 | 9.33E-14 | 2.48E-11 | 0.635 (0.599; 0.671) | -0.250 |
| hsa-miR-590-5p | 3.24E-16 | 9.86E-14 | 7.97E-14 | 0.652 (0.617; 0.687) | -0.430 |
| hsa-miR-26b-3p | 3.31E-16 | 1.00E-13 | 9.73E-11 | 0.609 (0.573; 0.645) | -0.142 |
| hsa-miR-4485-3p | 3.49E-16 | 1.05E-13 | 2.12E-11 | 0.664 (0.629; 0.699) | 0.275 |
| hsa-miR-1587 | 3.77E-16 | 1.14E-13 | 3.05E-12 | 0.63 (0.594; 0.665) | 0.183 |
| hsa-miR-3665 | 4.85E-16 | 1.45E-13 | 2.04E-12 | 0.635 (0.599; 0.67) | 0.212 |
| hsa-miR-15b-5p | 4.87E-16 | 1.45E-13 | 1.47E-09 | 0.607 (0.571; 0.643) | -0.154 |
| hsa-miR-146b-5p | 5.70E-16 | 1.70E-13 | 1.33E-07 | 0.594 (0.557; 0.631) | -0.397 |
| hsa-miR-30d-5p | 6.08E-16 | 1.81E-13 | 2.43E-11 | 0.629 (0.594; 0.665) | 0.200 |
| hsa-miR-1271-5p | 9.29E-16 | 2.75E-13 | 9.35E-09 | 0.587 (0.551; 0.623) | -0.285 |
| hsa-miR-5739 | 9.97E-16 | 2.94E-13 | 1.96E-09 | 0.606 (0.57; 0.642) | 0.431 |
| hsa-miR-550a-3-5p | 1.30E-15 | 3.82E-13 | 3.72E-10 | 0.61 (0.573; 0.646) | -0.233 |
| hsa-miR-155-5p | 1.31E-15 | 3.83E-13 | 2.17E-11 | 0.649 (0.614; 0.684) | -0.231 |
| hsa-miR-339-5p | 1.42E-15 | 4.14E-13 | 3.54E-13 | 0.634 (0.598; 0.669) | -0.282 |
| hsa-miR-6740-5p | 1.54E-15 | 4.47E-13 | 1.72E-08 | 0.626 (0.59; 0.661) | 0.219 |
| hsa-miR-331-3p | 1.59E-15 | 4.61E-13 | 9.04E-12 | 0.607 (0.571; 0.644) | -0.302 |
| hsa-miR-4728-5p | 1.90E-15 | 5.48E-13 | 1.27E-12 | 0.668 (0.633; 0.703) | 0.292 |
| hsa-miR-6717-5p | 2.36E-15 | 6.79E-13 | 2.57E-07 | 0.625 (0.59; 0.661) | 0.216 |
| hsa-miR-222-3p | 3.09E-15 | 8.88E-13 | 1.36E-11 | 0.636 (0.601; 0.672) | -0.305 |
| hsa-miR-6132 | 3.23E-15 | 9.23E-13 | 7.43E-12 | 0.648 (0.612; 0.683) | 0.312 |
| hsa-miR-335-5p | 3.66E-15 | 1.04E-12 | 6.47E-10 | 0.604 (0.568; 0.64) | -0.308 |
| hsa-miR-598-3p | 3.77E-15 | 1.07E-12 | 2.63E-09 | 0.624 (0.589; 0.66) | -0.193 |
| hsa-miR-5690 | 4.30E-15 | 1.22E-12 | 1.04E-11 | 0.608 (0.571; 0.645) | -0.405 |
| hsa-miR-766-3p | 5.66E-15 | 1.60E-12 | 4.40E-09 | 0.608 (0.571; 0.644) | -0.263 |
| hsa-miR-197-5p | 6.07E-15 | 1.70E-12 | 1.77E-08 | 0.609 (0.573; 0.646) | 0.215 |
| hsa-miR-6785-5p | 1.06E-14 | 2.97E-12 | 6.53E-11 | 0.672 (0.637; 0.707) | 0.449 |
| hsa-miR-145-5p | 1.15E-14 | 3.22E-12 | 1.50E-10 | 0.606 (0.569; 0.642) | -0.256 |
| hsa-miR-8069 | 3.69E-14 | 1.03E-11 | 3.06E-11 | 0.629 (0.593; 0.665) | 0.189 |
| hsa-miR-575 | 4.21E-14 | 1.17E-11 | 1.55E-08 | 0.644 (0.609; 0.679) | 0.221 |
| hsa-miR-3605-3p | 4.43E-14 | 1.22E-11 | 3.53E-11 | 0.605 (0.568; 0.641) | -0.160 |
| hsa-miR-3200-3p | 4.70E-14 | 1.29E-11 | 5.02E-08 | 0.621 (0.585; 0.657) | -0.239 |
| hsa-miR-4721 | 6.70E-14 | 1.84E-11 | 9.92E-11 | 0.654 (0.619; 0.689) | 0.326 |
| hsa-miR-4317 | 7.25E-14 | 1.98E-11 | 7.16E-10 | 0.602 (0.566; 0.639) | -0.171 |
| hsa-miR-4281 | 9.25E-14 | 2.52E-11 | 2.79E-08 | 0.606 (0.569; 0.642) | 0.193 |
| hsa-miR-628-5p | 1.18E-13 | 3.20E-11 | 1.64E-08 | 0.598 (0.562; 0.634) | -0.177 |
| hsa-miR-1202 | 1.33E-13 | 3.58E-11 | 1.45E-08 | 0.618 (0.581; 0.654) | 0.244 |
| hsa-miR-361-3p | 1.80E-13 | 4.83E-11 | 8.84E-10 | 0.604 (0.567; 0.64) | -0.180 |
| hsa-miR-4713-3p | 1.82E-13 | 4.87E-11 | 2.05E-06 | 0.613 (0.577; 0.649) | 0.198 |
| hsa-miR-183-3p | 1.84E-13 | 4.92E-11 | 1.38E-10 | 0.619 (0.583; 0.655) | -0.217 |
| hsa-miR-6124 | 1.85E-13 | 4.93E-11 | 2.81E-07 | 0.632 (0.596; 0.668) | 0.203 |
| hsa-miR-628-3p | 2.86E-13 | 7.57E-11 | 6.01E-11 | 0.627 (0.591; 0.663) | -0.155 |
| hsa-miR-330-3p | 4.23E-13 | 1.12E-10 | 2.79E-09 | 0.59 (0.553; 0.626) | -0.176 |
| hsa-miR-638 | 4.35E-13 | 1.14E-10 | 6.55E-11 | 0.625 (0.589; 0.661) | 0.185 |
| hsa-let-7a-5p | 4.47E-13 | 1.17E-10 | 1.09E-08 | 0.6 (0.564; 0.636) | -0.173 |
| hsa-miR-103a-3p | 5.19E-13 | 1.36E-10 | 6.25E-12 | 0.646 (0.611; 0.681) | -0.190 |
| hsa-miR-6780a-5p | 6.91E-13 | 1.80E-10 | 2.57E-06 | 0.613 (0.577; 0.65) | 0.181 |
| hsa-miR-133b | 7.34E-13 | 1.90E-10 | 3.33E-07 | 0.592 (0.555; 0.628) | -0.349 |
| hsa-miR-107 | 7.51E-13 | 1.94E-10 | 8.47E-12 | 0.64 (0.604; 0.675) | -0.179 |
| hsa-miR-26b-5p | 1.06E-12 | 2.71E-10 | 3.82E-09 | 0.601 (0.564; 0.638) | -0.374 |
| hsa-miR-19b-3p | 1.48E-12 | 3.78E-10 | 3.10E-09 | 0.633 (0.597; 0.668) | -0.207 |
| hsa-miR-128-3p | 1.55E-12 | 3.95E-10 | 7.41E-10 | 0.603 (0.566; 0.639) | -0.257 |
| hsa-miR-142-5p | 1.73E-12 | 4.40E-10 | 3.00E-09 | 0.612 (0.576; 0.648) | -0.261 |
| hsa-miR-8063 | 1.87E-12 | 4.73E-10 | 2.26E-09 | 0.624 (0.588; 0.66) | 0.227 |
| hsa-miR-3940-5p | 2.47E-12 | 6.23E-10 | 1.40E-09 | 0.619 (0.584; 0.655) | 0.136 |
| hsa-miR-1246 | 3.16E-12 | 7.93E-10 | 1.38E-07 | 0.616 (0.579; 0.652) | 0.295 |
| hsa-miR-4763-3p | 6.23E-12 | 1.56E-09 | 1.12E-07 | 0.604 (0.568; 0.64) | 0.163 |
| hsa-miR-4270 | 7.51E-12 | 1.87E-09 | 4.97E-07 | 0.617 (0.581; 0.653) | 0.171 |
| hsa-miR-6131 | 7.62E-12 | 1.89E-09 | 2.59E-05 | 0.606 (0.569; 0.642) | 0.192 |
| hsa-miR-5001-5p | 9.07E-12 | 2.24E-09 | 2.14E-08 | 0.607 (0.571; 0.643) | 0.156 |
| hsa-miR-23b-3p | 9.78E-12 | 2.41E-09 | 4.70E-08 | 0.59 (0.554; 0.627) | -0.265 |
| hsa-miR-6727-5p | 1.43E-11 | 3.50E-09 | 1.79E-08 | 0.606 (0.57; 0.643) | 0.211 |
| hsa-miR-30e-3p | 2.08E-11 | 5.07E-09 | 1.28E-08 | 0.578 (0.54; 0.615) | -0.315 |
| hsa-miR-181a-2-3p | 2.26E-11 | 5.49E-09 | 1.90E-08 | 0.592 (0.556; 0.628) | -0.128 |
| hsa-miR-942-3p | 2.43E-11 | 5.87E-09 | 2.27E-10 | 0.598 (0.562; 0.635) | -0.176 |
| hsa-miR-361-5p | 2.76E-11 | 6.65E-09 | 4.39E-07 | 0.574 (0.537; 0.611) | -0.191 |
| hsa-miR-3656 | 5.43E-11 | 1.30E-08 | 1.46E-08 | 0.606 (0.569; 0.642) | 0.232 |
| hsa-miR-664a-3p | 5.59E-11 | 1.34E-08 | 1.58E-09 | 0.621 (0.585; 0.658) | -0.174 |
| hsa-let-7i-5p | 9.94E-11 | 2.36E-08 | 2.32E-09 | 0.62 (0.583; 0.656) | -0.253 |
| hsa-miR-365a-3p | 1.37E-10 | 3.26E-08 | 3.21E-08 | 0.587 (0.551; 0.623) | -0.201 |
| hsa-miR-6734-5p | 1.40E-10 | 3.29E-08 | 8.21E-05 | 0.606 (0.57; 0.642) | 0.167 |
| hsa-miR-199b-5p | 1.78E-10 | 4.18E-08 | 2.84E-07 | 0.586 (0.55; 0.623) | -0.314 |
| hsa-miR-140-3p | 2.55E-10 | 5.96E-08 | 6.11E-07 | 0.563 (0.526; 0.6) | -0.144 |
| hsa-miR-378a-5p | 3.45E-10 | 8.04E-08 | 1.29E-08 | 0.585 (0.548; 0.621) | -0.220 |
| hsa-miR-5088-5p | 3.99E-10 | 9.26E-08 | 2.23E-05 | 0.601 (0.564; 0.637) | 0.165 |
| hsa-miR-1915-3p | 5.05E-10 | 1.17E-07 | 3.22E-07 | 0.587 (0.55; 0.623) | 0.179 |
| hsa-miR-5581-5p | 6.20E-10 | 1.43E-07 | 6.18E-05 | 0.601 (0.565; 0.637) | 0.166 |
| hsa-miR-5787 | 8.48E-10 | 1.94E-07 | 3.22E-06 | 0.632 (0.596; 0.668) | 0.303 |
| hsa-miR-15b-3p | 1.41E-09 | 3.22E-07 | 1.70E-07 | 0.585 (0.549; 0.622) | -0.163 |
| hsa-miR-4442 | 1.58E-09 | 3.58E-07 | 1.64E-05 | 0.586 (0.55; 0.623) | 0.176 |
| hsa-miR-625-5p | 1.72E-09 | 3.90E-07 | 6.58E-07 | 0.589 (0.552; 0.625) | -0.214 |
| hsa-miR-342-3p | 2.00E-09 | 4.50E-07 | 1.39E-03 | 0.554 (0.517; 0.591) | -0.152 |
| hsa-miR-664a-5p | 2.46E-09 | 5.50E-07 | 3.51E-06 | 0.595 (0.558; 0.632) | 0.206 |
| hsa-miR-6724-5p | 2.52E-09 | 5.61E-07 | 9.25E-08 | 0.587 (0.55; 0.623) | 0.172 |
| hsa-miR-4746-3p | 2.59E-09 | 5.75E-07 | 3.60E-08 | 0.607 (0.571; 0.643) | 0.099 |
| hsa-miR-6779-5p | 3.14E-09 | 6.94E-07 | 7.84E-07 | 0.603 (0.567; 0.64) | 0.107 |
| hsa-miR-664b-3p | 6.66E-09 | 1.46E-06 | 9.74E-08 | 0.581 (0.544; 0.617) | -0.126 |
| hsa-miR-4484 | 6.94E-09 | 1.52E-06 | 4.35E-08 | 0.614 (0.578; 0.65) | 0.080 |
| hsa-miR-627-5p | 7.17E-09 | 1.56E-06 | 6.73E-08 | 0.61 (0.574; 0.646) | -0.190 |
| hsa-miR-3198 | 7.33E-09 | 1.59E-06 | 2.08E-04 | 0.602 (0.565; 0.638) | 0.170 |
| hsa-miR-1305 | 7.80E-09 | 1.68E-06 | 8.68E-04 | 0.586 (0.55; 0.623) | 0.140 |
| hsa-miR-3196 | 9.09E-09 | 1.95E-06 | 1.31E-06 | 0.594 (0.558; 0.631) | 0.147 |
| hsa-miR-3195 | 1.39E-08 | 2.98E-06 | 3.68E-04 | 0.591 (0.554; 0.628) | 0.197 |
| hsa-miR-371b-5p | 1.43E-08 | 3.05E-06 | 1.60E-07 | 0.598 (0.562; 0.635) | 0.145 |
| hsa-miR-6085 | 1.52E-08 | 3.22E-06 | 2.23E-04 | 0.56 (0.523; 0.597) | 0.247 |
| hsa-miR-5006-5p | 1.63E-08 | 3.43E-06 | 2.09E-07 | 0.605 (0.569; 0.642) | 0.182 |
| hsa-miR-1255a | 1.80E-08 | 3.77E-06 | 3.95E-08 | 0.604 (0.568; 0.64) | -0.087 |
| hsa-miR-641 | 1.87E-08 | 3.92E-06 | 3.22E-05 | 0.571 (0.535; 0.608) | -0.099 |
| hsa-miR-1288-3p | 1.95E-08 | 4.06E-06 | 6.78E-04 | 0.585 (0.548; 0.621) | 0.135 |
| hsa-miR-550a-3p | 2.40E-08 | 4.96E-06 | 7.52E-06 | 0.587 (0.55; 0.623) | -0.170 |
| hsa-miR-6893-5p | 2.56E-08 | 5.27E-06 | 4.66E-07 | 0.6 (0.563; 0.636) | 0.161 |
| hsa-miR-495-3p | 2.69E-08 | 5.51E-06 | 9.19E-03 | 0.553 (0.516; 0.59) | -0.274 |
| hsa-miR-6165 | 3.77E-08 | 7.70E-06 | 2.37E-04 | 0.556 (0.519; 0.593) | 0.207 |
| hsa-miR-3125 | 4.20E-08 | 8.53E-06 | 8.92E-04 | 0.578 (0.542; 0.615) | 0.139 |
| hsa-miR-4428 | 4.26E-08 | 8.61E-06 | 3.11E-06 | 0.609 (0.573; 0.645) | 0.191 |
| hsa-miR-363-3p | 4.48E-08 | 9.00E-06 | 2.72E-03 | 0.576 (0.539; 0.612) | -0.162 |
| hsa-miR-4716-3p | 5.03E-08 | 1.01E-05 | 1.61E-03 | 0.587 (0.551; 0.624) | 0.148 |
| hsa-miR-3679-5p | 6.04E-08 | 1.20E-05 | 3.77E-05 | 0.584 (0.547; 0.62) | 0.152 |
| hsa-let-7b-5p | 6.23E-08 | 1.23E-05 | 4.37E-04 | 0.564 (0.527; 0.601) | 0.227 |
| hsa-miR-125a-5p | 6.49E-08 | 1.28E-05 | 2.44E-05 | 0.576 (0.539; 0.612) | -0.222 |
| hsa-miR-139-5p | 7.11E-08 | 1.39E-05 | 3.03E-04 | 0.571 (0.534; 0.608) | -0.134 |
| hsa-miR-5100 | 7.70E-08 | 1.50E-05 | 3.09E-03 | 0.548 (0.511; 0.585) | 0.266 |
| hsa-miR-3652 | 8.95E-08 | 1.74E-05 | 1.93E-04 | 0.566 (0.53; 0.603) | 0.139 |
| hsa-miR-221-3p | 1.08E-07 | 2.08E-05 | 1.48E-06 | 0.592 (0.555; 0.629) | -0.219 |
| hsa-miR-937-5p | 1.13E-07 | 2.18E-05 | 4.18E-05 | 0.574 (0.538; 0.611) | 0.124 |
| hsa-miR-191-5p | 1.37E-07 | 2.61E-05 | 1.51E-06 | 0.574 (0.537; 0.611) | -0.117 |
| hsa-miR-338-3p | 1.41E-07 | 2.67E-05 | 2.62E-06 | 0.584 (0.548; 0.621) | -0.194 |
| hsa-miR-654-3p | 1.65E-07 | 3.12E-05 | 2.75E-02 | 0.548 (0.512; 0.585) | -0.259 |
| hsa-miR-629-5p | 1.71E-07 | 3.22E-05 | 3.15E-07 | 0.585 (0.549; 0.622) | -0.145 |
| hsa-miR-99b-5p | 1.88E-07 | 3.51E-05 | 2.22E-03 | 0.558 (0.522; 0.595) | -0.197 |
| hsa-miR-6068 | 1.94E-07 | 3.61E-05 | 4.84E-07 | 0.598 (0.562; 0.634) | 0.136 |
| hsa-miR-423-5p | 1.98E-07 | 3.67E-05 | 5.04E-03 | 0.562 (0.525; 0.598) | 0.126 |
| hsa-miR-106b-5p | 2.47E-07 | 4.54E-05 | 6.08E-08 | 0.61 (0.573; 0.647) | -0.238 |
| hsa-miR-532-5p | 2.58E-07 | 4.71E-05 | 7.35E-08 | 0.597 (0.56; 0.633) | -0.168 |
| hsa-miR-6763-5p | 3.19E-07 | 5.80E-05 | 3.84E-05 | 0.574 (0.537; 0.611) | 0.144 |
| hsa-miR-501-3p | 3.24E-07 | 5.87E-05 | 9.50E-09 | 0.6 (0.564; 0.636) | -0.103 |
| hsa-miR-505-5p | 3.89E-07 | 7.01E-05 | 9.30E-09 | 0.591 (0.554; 0.627) | -0.149 |
| hsa-miR-610 | 3.93E-07 | 7.04E-05 | 2.09E-05 | 0.569 (0.532; 0.606) | -0.084 |
| hsa-miR-7110-5p | 3.95E-07 | 7.04E-05 | 2.90E-05 | 0.57 (0.533; 0.607) | 0.175 |
| hsa-miR-7107-5p | 4.47E-07 | 7.90E-05 | 1.09E-04 | 0.568 (0.531; 0.604) | 0.177 |
| hsa-miR-505-3p | 4.93E-07 | 8.67E-05 | 2.98E-06 | 0.564 (0.527; 0.601) | -0.155 |
| hsa-miR-502-3p | 5.79E-07 | 1.01E-04 | 1.72E-07 | 0.59 (0.554; 0.626) | -0.146 |
| hsa-miR-296-5p | 1.26E-06 | 2.19E-04 | 1.61E-07 | 0.59 (0.554; 0.627) | -0.120 |
| hsa-miR-4466 | 1.31E-06 | 2.27E-04 | 2.12E-04 | 0.574 (0.537; 0.61) | 0.109 |
| hsa-miR-6880-3p | 2.19E-06 | 3.77E-04 | 7.77E-06 | 0.598 (0.561; 0.634) | 0.072 |
| hsa-miR-1207-5p | 2.60E-06 | 4.45E-04 | 1.54E-03 | 0.557 (0.52; 0.594) | 0.122 |
| hsa-miR-3940-3p | 2.90E-06 | 4.94E-04 | 6.71E-07 | 0.56 (0.523; 0.597) | -0.082 |
| hsa-miR-223-3p | 3.94E-06 | 6.65E-04 | 5.92E-03 | 0.544 (0.507; 0.581) | -0.084 |
| hsa-miR-7108-5p | 4.36E-06 | 7.33E-04 | 1.97E-04 | 0.57 (0.533; 0.606) | 0.128 |
| hsa-miR-320d | 5.19E-06 | 8.66E-04 | 3.83E-04 | 0.573 (0.536; 0.609) | 0.115 |
| hsa-miR-146a-5p | 5.20E-06 | 8.66E-04 | 7.68E-03 | 0.566 (0.529; 0.602) | -0.121 |
| hsa-miR-125b-5p | 6.57E-06 | 1.08E-03 | 3.21E-05 | 0.569 (0.533; 0.606) | -0.299 |
| hsa-miR-181a-5p | 1.34E-05 | 2.19E-03 | 3.07E-04 | 0.568 (0.531; 0.606) | 0.219 |
| hsa-miR-151a-5p | 1.56E-05 | 2.54E-03 | 1.48E-05 | 0.554 (0.517; 0.591) | -0.113 |
| hsa-miR-485-3p | 1.76E-05 | 2.85E-03 | 5.57E-02 | 0.533 (0.496; 0.57) | -0.175 |
| hsa-miR-6763-3p | 1.90E-05 | 3.07E-03 | 1.60E-04 | 0.586 (0.55; 0.623) | 0.065 |
| hsa-miR-4433a-5p | 1.92E-05 | 3.07E-03 | 1.10E-04 | 0.578 (0.542; 0.615) | 0.069 |
| hsa-miR-6757-5p | 2.06E-05 | 3.27E-03 | 3.94E-04 | 0.566 (0.53; 0.603) | 0.086 |
| hsa-miR-500a-3p | 2.76E-05 | 4.36E-03 | 1.51E-06 | 0.579 (0.542; 0.615) | -0.131 |
| hsa-miR-501-5p | 3.38E-05 | 5.31E-03 | 1.13E-05 | 0.569 (0.532; 0.606) | -0.105 |
| hsa-miR-148a-3p | 4.12E-05 | 6.42E-03 | 2.53E-04 | 0.567 (0.53; 0.604) | -0.181 |
| hsa-miR-484 | 4.34E-05 | 6.72E-03 | 3.10E-06 | 0.578 (0.541; 0.614) | -0.104 |
| hsa-miR-4299 | 4.75E-05 | 7.31E-03 | 7.34E-05 | 0.562 (0.525; 0.599) | 0.112 |
| hsa-miR-4497 | 4.97E-05 | 7.61E-03 | 1.01E-04 | 0.578 (0.541; 0.615) | 0.101 |
| hsa-miR-130b-3p | 5.09E-05 | 7.74E-03 | 6.69E-06 | 0.572 (0.535; 0.608) | -0.105 |
| hsa-miR-933 | 5.42E-05 | 8.18E-03 | 2.83E-04 | 0.57 (0.533; 0.607) | 0.051 |
| hsa-miR-320c | 5.92E-05 | 8.89E-03 | 2.16E-03 | 0.562 (0.525; 0.599) | 0.099 |
| hsa-miR-6073 | 5.98E-05 | 8.92E-03 | 1.89E-03 | 0.541 (0.504; 0.578) | 0.120 |
| hsa-miR-4769-3p | 8.88E-05 | 1.31E-02 | 9.86E-04 | 0.57 (0.533; 0.607) | 0.057 |
| hsa-miR-7152-3p | 9.22E-05 | 1.36E-02 | 4.08E-03 | 0.56 (0.524; 0.597) | 0.126 |
| hsa-miR-4653-3p | 9.38E-05 | 1.37E-02 | 3.09E-02 | 0.562 (0.526; 0.599) | 0.096 |
| hsa-miR-4685-5p | 9.96E-05 | 1.44E-02 | 1.50E-01 | 0.542 (0.505; 0.579) | 0.175 |
| hsa-miR-30c-1-3p | 1.01E-04 | 1.45E-02 | 1.30E-03 | 0.565 (0.528; 0.602) | -0.027 |
| hsa-miR-16-2-3p | 1.03E-04 | 1.47E-02 | 1.02E-01 | 0.523 (0.486; 0.561) | 0.121 |
| hsa-miR-1914-3p | 1.08E-04 | 1.53E-02 | 2.46E-02 | 0.555 (0.518; 0.591) | 0.119 |
| hsa-miR-1249-3p | 1.12E-04 | 1.58E-02 | 7.22E-03 | 0.547 (0.51; 0.584) | 0.060 |
| hsa-miR-4739 | 1.39E-04 | 1.95E-02 | 4.80E-03 | 0.556 (0.519; 0.593) | 0.085 |
| hsa-miR-4749-3p | 1.56E-04 | 2.18E-02 | 6.94E-04 | 0.578 (0.542; 0.615) | 0.056 |
| hsa-let-7f-1-3p | 1.79E-04 | 2.48E-02 | 1.76E-04 | 0.588 (0.551; 0.624) | 0.067 |
| hsa-miR-6767-5p | 1.80E-04 | 2.48E-02 | 7.46E-02 | 0.553 (0.516; 0.59) | 0.092 |
| hsa-miR-6515-3p | 1.99E-04 | 2.70E-02 | 1.20E-03 | 0.573 (0.537; 0.61) | 0.060 |
| hsa-miR-4732-3p | 2.47E-04 | 3.33E-02 | 2.54E-04 | 0.553 (0.516; 0.59) | -0.114 |
| hsa-miR-4669 | 2.47E-04 | 3.33E-02 | 8.69E-03 | 0.548 (0.511; 0.585) | 0.109 |
| hsa-miR-103a-2-5p | 2.57E-04 | 3.41E-02 | 1.11E-06 | 0.591 (0.554; 0.627) | -0.093 |
| hsa-miR-4788 | 2.60E-04 | 3.43E-02 | 1.00E-02 | 0.537 (0.5; 0.574) | 0.142 |
| hsa-miR-6789-5p | 2.71E-04 | 3.55E-02 | 6.16E-04 | 0.563 (0.527; 0.6) | 0.137 |
| hsa-miR-6891-5p | 3.00E-04 | 3.90E-02 | 2.26E-02 | 0.549 (0.512; 0.586) | 0.089 |
| hsa-miR-6870-3p | 3.27E-04 | 4.22E-02 | 1.15E-03 | 0.566 (0.529; 0.602) | 0.038 |
| hsa-miR-451a | 3.31E-04 | 4.24E-02 | 1.26E-03 | 0.594 (0.557; 0.631) | -0.020 |
| hsa-miR-100-5p | 5.00E-04 | 6.35E-02 | 1.71E-03 | 0.557 (0.521; 0.594) | -0.469 |
| hsa-miR-574-3p | 5.26E-04 | 6.63E-02 | 1.23E-05 | 0.553 (0.517; 0.59) | -0.095 |
| hsa-miR-149-5p | 5.93E-04 | 7.41E-02 | 6.49E-04 | 0.573 (0.536; 0.61) | 0.059 |
| hsa-miR-939-5p | 6.12E-04 | 7.59E-02 | 2.23E-02 | 0.543 (0.506; 0.58) | 0.103 |
| hsa-miR-4324 | 6.30E-04 | 7.74E-02 | 1.53E-03 | 0.559 (0.522; 0.595) | -0.051 |
| hsa-miR-320e | 6.46E-04 | 7.88E-02 | 1.09E-02 | 0.551 (0.514; 0.588) | 0.084 |
| hsa-miR-3653-3p | 7.20E-04 | 8.72E-02 | 6.93E-02 | 0.53 (0.493; 0.567) | -0.133 |
| hsa-miR-320b | 7.38E-04 | 8.86E-02 | 1.41E-02 | 0.546 (0.509; 0.583) | 0.090 |
| hsa-miR-409-3p | 8.58E-04 | 1.02E-01 | 3.41E-01 | 0.526 (0.489; 0.563) | -0.191 |
| hsa-miR-3614-5p | 9.09E-04 | 1.07E-01 | 2.68E-03 | 0.565 (0.529; 0.602) | 0.048 |
| hsa-let-7c-5p | 1.71E-03 | 2.00E-01 | 1.02E-01 | 0.521 (0.484; 0.558) | 0.130 |
| hsa-miR-7847-3p | 1.75E-03 | 2.03E-01 | 1.92E-02 | 0.545 (0.508; 0.582) | 0.096 |
| hsa-miR-92a-3p | 1.88E-03 | 2.16E-01 | 1.04E-01 | 0.545 (0.508; 0.582) | 0.051 |
| hsa-miR-7974 | 1.88E-03 | 2.16E-01 | 2.33E-03 | 0.562 (0.526; 0.599) | 0.052 |
| hsa-miR-6069 | 1.89E-03 | 2.16E-01 | 5.73E-03 | 0.557 (0.52; 0.594) | 0.060 |
| hsa-miR-6797-3p | 2.15E-03 | 2.41E-01 | 6.97E-03 | 0.556 (0.519; 0.592) | 0.058 |
| hsa-miR-1306-5p | 2.23E-03 | 2.48E-01 | 9.88E-05 | 0.559 (0.522; 0.596) | -0.074 |
| hsa-miR-378a-3p | 2.35E-03 | 2.59E-01 | 2.00E-01 | 0.524 (0.486; 0.561) | 0.087 |
| hsa-miR-29c-5p | 2.39E-03 | 2.60E-01 | 1.46E-04 | 0.548 (0.511; 0.585) | -0.082 |
| hsa-miR-30e-5p | 2.53E-03 | 2.73E-01 | 3.18E-03 | 0.568 (0.532; 0.605) | -0.097 |
| hsa-miR-1268b | 2.62E-03 | 2.81E-01 | 2.73E-02 | 0.541 (0.504; 0.578) | 0.091 |
| hsa-miR-6777-3p | 2.72E-03 | 2.89E-01 | 4.75E-03 | 0.554 (0.517; 0.59) | 0.043 |
| hsa-miR-491-5p | 3.14E-03 | 3.30E-01 | 1.75E-02 | 0.546 (0.509; 0.583) | -0.017 |
| hsa-miR-1228-3p | 3.32E-03 | 3.45E-01 | 1.52E-02 | 0.55 (0.513; 0.587) | 0.053 |
| hsa-miR-6785-3p | 3.39E-03 | 3.49E-01 | 1.31E-02 | 0.555 (0.518; 0.591) | 0.045 |
| hsa-miR-2116-3p | 3.48E-03 | 3.55E-01 | 4.51E-03 | 0.566 (0.529; 0.603) | 0.054 |
| hsa-miR-1268a | 3.55E-03 | 3.58E-01 | 7.25E-03 | 0.568 (0.531; 0.604) | -0.080 |
| hsa-miR-193a-5p | 5.00E-03 | 5.00E-01 | 1.95E-03 | 0.533 (0.496; 0.57) | -0.050 |
| hsa-let-7b-3p | 5.46E-03 | 5.40E-01 | 5.41E-03 | 0.56 (0.523; 0.596) | 0.051 |
| hsa-miR-340-5p | 5.52E-03 | 5.41E-01 | 4.74E-03 | 0.55 (0.513; 0.588) | -0.139 |
| hsa-miR-564 | 5.95E-03 | 5.77E-01 | 8.32E-02 | 0.53 (0.493; 0.567) | -0.061 |
| hsa-miR-6826-5p | 6.15E-03 | 5.91E-01 | 5.98E-02 | 0.537 (0.5; 0.575) | 0.128 |
| hsa-miR-4664-3p | 6.22E-03 | 5.91E-01 | 8.53E-03 | 0.551 (0.514; 0.588) | 0.049 |
| hsa-miR-574-5p | 6.32E-03 | 5.94E-01 | 7.59E-02 | 0.52 (0.483; 0.557) | 0.118 |
| hsa-miR-6760-3p | 6.57E-03 | 6.11E-01 | 5.23E-03 | 0.549 (0.512; 0.586) | 0.038 |
| hsa-let-7d-3p | 6.82E-03 | 6.28E-01 | 4.06E-03 | 0.563 (0.526; 0.599) | -0.059 |
| hsa-miR-4310 | 6.85E-03 | 6.28E-01 | 7.14E-03 | 0.553 (0.516; 0.59) | 0.053 |
| hsa-miR-4758-3p | 6.96E-03 | 6.28E-01 | 9.87E-03 | 0.548 (0.511; 0.585) | 0.046 |
| hsa-miR-320a | 8.18E-03 | 7.28E-01 | 4.51E-02 | 0.538 (0.501; 0.575) | 0.088 |
| hsa-miR-6752-3p | 1.07E-02 | 9.42E-01 | 2.45E-02 | 0.554 (0.517; 0.591) | 0.037 |
| hsa-miR-181b-5p | 1.23E-02 | 1.00E+00 | 6.09E-04 | 0.533 (0.496; 0.57) | -0.079 |
| hsa-miR-1238-3p | 1.31E-02 | 1.00E+00 | 2.63E-02 | 0.547 (0.51; 0.584) | 0.051 |
| hsa-miR-1539 | 1.32E-02 | 1.00E+00 | 2.95E-02 | 0.552 (0.515; 0.589) | 0.047 |
| hsa-miR-4793-5p | 1.42E-02 | 1.00E+00 | 1.92E-02 | 0.542 (0.505; 0.579) | 0.050 |
| hsa-miR-1234-3p | 1.47E-02 | 1.00E+00 | 4.63E-02 | 0.533 (0.496; 0.57) | 0.048 |
| hsa-miR-6851-3p | 1.63E-02 | 1.00E+00 | 2.42E-02 | 0.557 (0.52; 0.594) | 0.042 |
| hsa-miR-4665-3p | 1.64E-02 | 1.00E+00 | 9.49E-02 | 0.535 (0.498; 0.572) | 0.051 |
| hsa-miR-191-3p | 1.67E-02 | 1.00E+00 | 3.05E-02 | 0.538 (0.501; 0.575) | 0.047 |
| hsa-miR-378i | 2.14E-02 | 1.00E+00 | 4.93E-01 | 0.508 (0.471; 0.545) | 0.064 |
| hsa-miR-5010-3p | 2.29E-02 | 1.00E+00 | 2.35E-02 | 0.552 (0.515; 0.588) | 0.033 |
| hsa-miR-425-5p | 2.31E-02 | 1.00E+00 | 3.12E-02 | 0.516 (0.479; 0.553) | -0.042 |
| hsa-miR-29a-3p | 2.76E-02 | 1.00E+00 | 1.97E-01 | 0.52 (0.483; 0.557) | -0.077 |
| hsa-miR-129-2-3p | 2.81E-02 | 1.00E+00 | 8.91E-04 | 0.538 (0.501; 0.575) | -0.043 |
| hsa-miR-6731-3p | 3.14E-02 | 1.00E+00 | 3.49E-02 | 0.54 (0.503; 0.576) | 0.034 |
| hsa-miR-6865-3p | 3.29E-02 | 1.00E+00 | 4.71E-02 | 0.538 (0.501; 0.575) | 0.044 |
| hsa-miR-223-5p | 3.30E-02 | 1.00E+00 | 2.71E-02 | 0.537 (0.5; 0.574) | 0.043 |
| hsa-miR-6800-3p | 3.60E-02 | 1.00E+00 | 5.40E-02 | 0.541 (0.504; 0.578) | 0.043 |
| hsa-miR-874-3p | 3.62E-02 | 1.00E+00 | 7.81E-02 | 0.523 (0.486; 0.56) | -0.044 |
| hsa-miR-6791-5p | 3.64E-02 | 1.00E+00 | 1.08E-01 | 0.528 (0.491; 0.565) | 0.055 |
| hsa-miR-1825 | 3.93E-02 | 1.00E+00 | 7.82E-02 | 0.537 (0.5; 0.574) | 0.042 |
| hsa-miR-25-3p | 3.98E-02 | 1.00E+00 | 3.93E-01 | 0.515 (0.478; 0.553) | 0.029 |
| hsa-miR-6508-5p | 4.01E-02 | 1.00E+00 | 6.12E-02 | 0.536 (0.499; 0.573) | 0.044 |
| hsa-miR-6889-3p | 4.10E-02 | 1.00E+00 | 5.15E-02 | 0.537 (0.5; 0.574) | 0.040 |
| hsa-miR-6813-3p | 4.20E-02 | 1.00E+00 | 6.31E-02 | 0.533 (0.496; 0.57) | 0.036 |
| hsa-miR-1237-3p | 4.66E-02 | 1.00E+00 | 7.30E-02 | 0.535 (0.498; 0.572) | 0.029 |
| hsa-miR-362-5p | 5.00E-02 | 1.00E+00 | 1.97E-02 | 0.562 (0.526; 0.599) | -0.048 |
| hsa-miR-29c-3p | 5.68E-02 | 1.00E+00 | 7.47E-03 | 0.542 (0.505; 0.58) | -0.090 |
| hsa-miR-1260a | 5.73E-02 | 1.00E+00 | 9.17E-01 | 0.502 (0.461; 0.535) | 0.077 |
| hsa-miR-625-3p | 5.89E-02 | 1.00E+00 | 2.81E-01 | 0.517 (0.48; 0.554) | -0.022 |
| hsa-miR-210-3p | 6.22E-02 | 1.00E+00 | 2.53E-02 | 0.521 (0.483; 0.559) | -0.108 |
| hsa-miR-23a-3p | 6.33E-02 | 1.00E+00 | 1.12E-01 | 0.507 (0.47; 0.544) | -0.061 |
| hsa-miR-940 | 6.57E-02 | 1.00E+00 | 2.38E-01 | 0.52 (0.483; 0.558) | 0.039 |
| hsa-miR-33b-3p | 6.62E-02 | 1.00E+00 | 9.00E-02 | 0.533 (0.496; 0.57) | 0.035 |
| hsa-miR-1281 | 6.70E-02 | 1.00E+00 | 1.26E-01 | 0.53 (0.493; 0.567) | 0.041 |
| hsa-miR-6858-3p | 6.71E-02 | 1.00E+00 | 8.92E-02 | 0.537 (0.5; 0.574) | 0.033 |
| hsa-miR-1304-3p | 8.76E-02 | 1.00E+00 | 1.17E-01 | 0.53 (0.493; 0.567) | 0.037 |
| hsa-miR-550a-5p | 8.90E-02 | 1.00E+00 | 2.35E-02 | 0.543 (0.506; 0.58) | 0.030 |
| hsa-miR-6819-3p | 8.93E-02 | 1.00E+00 | 1.16E-01 | 0.523 (0.486; 0.56) | 0.033 |
| hsa-miR-4649-3p | 9.25E-02 | 1.00E+00 | 1.36E-01 | 0.521 (0.483; 0.558) | 0.038 |
| hsa-miR-6798-3p | 9.98E-02 | 1.00E+00 | 2.86E-01 | 0.52 (0.483; 0.557) | 0.015 |
| hsa-miR-4465 | 1.07E-01 | 1.00E+00 | 1.60E-01 | 0.506 (0.469; 0.543) | 0.065 |
| hsa-miR-4286 | 1.10E-01 | 1.00E+00 | 5.30E-01 | 0.501 (0.463; 0.538) | 0.079 |
| hsa-miR-6126 | 1.45E-01 | 1.00E+00 | 4.92E-01 | 0.507 (0.47; 0.544) | 0.041 |
| hsa-miR-4306 | 1.53E-01 | 1.00E+00 | 8.90E-01 | 0.515 (0.478; 0.552) | 0.024 |
| hsa-miR-3180-3p | 1.55E-01 | 1.00E+00 | 5.16E-01 | 0.508 (0.471; 0.545) | 0.059 |
| hsa-miR-30c-5p | 1.63E-01 | 1.00E+00 | 3.86E-01 | 0.509 (0.472; 0.546) | -0.034 |
| hsa-miR-22-3p | 1.72E-01 | 1.00E+00 | 2.65E-01 | 0.508 (0.455; 0.529) | 0.031 |
| hsa-miR-129-1-3p | 1.82E-01 | 1.00E+00 | 3.12E-01 | 0.508 (0.455; 0.529) | -0.021 |
| hsa-miR-7114-5p | 1.83E-01 | 1.00E+00 | 8.93E-01 | 0.503 (0.46; 0.535) | 0.063 |
| hsa-miR-4787-3p | 1.89E-01 | 1.00E+00 | 3.74E-01 | 0.511 (0.474; 0.548) | 0.024 |
| hsa-miR-6737-3p | 1.92E-01 | 1.00E+00 | 2.20E-01 | 0.516 (0.479; 0.553) | 0.028 |
| hsa-miR-486-5p | 1.97E-01 | 1.00E+00 | 6.43E-01 | 0.521 (0.484; 0.558) | 0.014 |
| hsa-miR-584-5p | 2.06E-01 | 1.00E+00 | 8.80E-01 | 0.504 (0.467; 0.541) | 0.045 |
| hsa-miR-197-3p | 2.31E-01 | 1.00E+00 | 8.51E-02 | 0.515 (0.478; 0.552) | -0.035 |
| hsa-miR-30a-5p | 2.50E-01 | 1.00E+00 | 3.74E-01 | 0.529 (0.492; 0.566) | 0.040 |
| hsa-miR-324-3p | 2.65E-01 | 1.00E+00 | 4.10E-01 | 0.515 (0.478; 0.552) | 0.021 |
| hsa-miR-186-5p | 2.84E-01 | 1.00E+00 | 7.63E-01 | 0.514 (0.477; 0.551) | 0.024 |
| hsa-miR-4725-5p | 3.22E-01 | 1.00E+00 | 5.02E-01 | 0.502 (0.465; 0.54) | 0.021 |
| hsa-miR-8485 | 3.30E-01 | 1.00E+00 | 3.01E-01 | 0.52 (0.483; 0.557) | -0.044 |
| hsa-miR-3162-3p | 3.43E-01 | 1.00E+00 | 4.44E-01 | 0.508 (0.471; 0.545) | 0.024 |
| hsa-miR-7977 | 3.57E-01 | 1.00E+00 | 7.09E-01 | 0.506 (0.469; 0.544) | 0.055 |
| hsa-miR-3613-3p | 4.07E-01 | 1.00E+00 | 3.37E-01 | 0.521 (0.484; 0.558) | 0.011 |
| hsa-miR-185-5p | 4.49E-01 | 1.00E+00 | 4.51E-01 | 0.503 (0.466; 0.54) | 0.017 |
| hsa-miR-339-3p | 4.62E-01 | 1.00E+00 | 4.55E-01 | 0.511 (0.474; 0.548) | -0.018 |
| hsa-miR-378g | 4.90E-01 | 1.00E+00 | 5.16E-01 | 0.512 (0.475; 0.549) | 0.021 |
| hsa-miR-7975 | 4.95E-01 | 1.00E+00 | 2.37E-01 | 0.512 (0.475; 0.549) | 0.019 |
| hsa-miR-324-5p | 5.73E-01 | 1.00E+00 | 6.17E-01 | 0.509 (0.472; 0.546) | -0.012 |
| hsa-miR-6812-3p | 5.86E-01 | 1.00E+00 | 6.05E-01 | 0.525 (0.488; 0.562) | -0.008 |
| hsa-miR-769-5p | 6.16E-01 | 1.00E+00 | 3.86E-01 | 0.511 (0.474; 0.548) | -0.012 |
| hsa-miR-1275 | 6.18E-01 | 1.00E+00 | 3.43E-02 | 0.527 (0.489; 0.564) | -0.015 |
| hsa-miR-151a-3p | 6.27E-01 | 1.00E+00 | 6.74E-01 | 0.522 (0.485; 0.559) | 0.013 |
| hsa-miR-29b-3p | 6.50E-01 | 1.00E+00 | 1.03E-01 | 0.53 (0.492; 0.567) | 0.021 |
| hsa-miR-3135b | 6.57E-01 | 1.00E+00 | 9.35E-01 | 0.514 (0.477; 0.552) | 0.032 |
| hsa-miR-4284 | 6.78E-01 | 1.00E+00 | 4.40E-01 | 0.502 (0.461; 0.535) | -0.014 |
| hsa-miR-6824-3p | 6.91E-01 | 1.00E+00 | 8.77E-01 | 0.504 (0.467; 0.542) | 0.006 |
| hsa-miR-4313 | 7.09E-01 | 1.00E+00 | 8.97E-01 | 0.501 (0.462; 0.537) | 0.008 |
| hsa-miR-378d | 7.11E-01 | 1.00E+00 | 7.66E-02 | 0.539 (0.502; 0.576) | -0.010 |
| hsa-miR-92b-3p | 7.29E-01 | 1.00E+00 | 6.71E-01 | 0.507 (0.456; 0.53) | -0.005 |
| hsa-miR-1908-3p | 7.47E-01 | 1.00E+00 | 8.84E-01 | 0.505 (0.468; 0.542) | 0.005 |
| hsa-miR-1227-3p | 8.31E-01 | 1.00E+00 | 8.21E-01 | 0.513 (0.476; 0.55) | 0.002 |
| hsa-miR-500a-5p | 8.74E-01 | 1.00E+00 | 1.87E-01 | 0.522 (0.485; 0.559) | -0.005 |
| hsa-miR-425-3p | 9.23E-01 | 1.00E+00 | 8.09E-01 | 0.5 (0.463; 0.537) | 0.002 |
| hsa-miR-4732-5p | 9.23E-01 | 1.00E+00 | 2.27E-01 | 0.521 (0.484; 0.558) | -0.003 |
| hsa-miR-1260b | 9.43E-01 | 1.00E+00 | 3.78E-01 | 0.529 (0.492; 0.566) | 0.002 |
| hsa-miR-151b | 9.54E-01 | 1.00E+00 | 1.73E-01 | 0.506 (0.469; 0.543) | -0.001 |
| hsa-miR-4436b-5p | 9.58E-01 | 1.00E+00 | 8.36E-01 | 0.501 (0.464; 0.538) | -0.001 |
| hsa-miR-4672 | 9.66E-01 | 1.00E+00 | 6.62E-01 | 0.508 (0.471; 0.545) | 0.001 |

**Table 7: Result metrics for the comparison of DCM versus control**

| miRNA | ttest_rawp | ttest_adjp | glm_rawp | AUC | log2FoldChange |
| --- | --- | --- | --- | --- | --- |
| hsa-miR-150-5p | 1.45E-15 | 6.31E-13 | 1.25E-11 | 0.658 (0.605; 0.711) | -0.382 |
| hsa-miR-3651 | 1.53E-15 | 6.67E-13 | 6.94E-15 | 0.703 (0.652; 0.753) | -0.323 |
| hsa-miR-139-3p | 7.83E-14 | 3.40E-11 | 8.83E-13 | 0.665 (0.613; 0.718) | -0.138 |
| hsa-miR-1275 | 1.08E-13 | 4.67E-11 | 1.92E-12 | 0.661 (0.609; 0.714) | -0.273 |
| hsa-let-7e-5p | 7.12E-13 | 3.08E-10 | 1.59E-12 | 0.646 (0.592; 0.7) | -0.253 |
| hsa-miR-652-3p | 1.34E-11 | 5.79E-09 | 1.40E-14 | 0.69 (0.638; 0.741) | -0.209 |
| hsa-miR-186-5p | 2.60E-11 | 1.12E-08 | 2.52E-13 | 0.678 (0.626; 0.731) | 0.205 |
| hsa-miR-564 | 1.41E-10 | 6.06E-08 | 1.70E-11 | 0.663 (0.61; 0.716) | -0.214 |
| hsa-miR-4465 | 1.50E-10 | 6.41E-08 | 1.92E-10 | 0.683 (0.63; 0.735) | -0.314 |
| hsa-miR-664a-3p | 1.84E-10 | 7.84E-08 | 1.93E-12 | 0.674 (0.62; 0.727) | -0.203 |
| hsa-miR-30d-5p | 2.15E-10 | 9.16E-08 | 6.36E-13 | 0.668 (0.615; 0.721) | 0.196 |
| hsa-miR-361-3p | 2.63E-10 | 1.12E-07 | 2.21E-08 | 0.633 (0.578; 0.687) | -0.184 |
| hsa-miR-4306 | 2.73E-10 | 1.16E-07 | 2.18E-12 | 0.658 (0.605; 0.712) | 0.127 |
| hsa-miR-342-5p | 2.87E-10 | 1.21E-07 | 7.76E-13 | 0.646 (0.592; 0.7) | -0.211 |
| hsa-miR-4484 | 8.88E-10 | 3.75E-07 | 8.73E-09 | 0.703 (0.653; 0.754) | 0.106 |
| hsa-miR-98-5p | 1.12E-09 | 4.73E-07 | 1.02E-11 | 0.662 (0.609; 0.716) | -0.268 |
| hsa-miR-126-3p | 3.09E-09 | 1.30E-06 | 3.02E-13 | 0.669 (0.616; 0.722) | -0.365 |
| hsa-miR-4672 | 3.43E-09 | 1.44E-06 | 2.21E-08 | 0.649 (0.595; 0.702) | -0.207 |
| hsa-miR-93-3p | 5.14E-09 | 2.15E-06 | 4.42E-09 | 0.632 (0.577; 0.686) | -0.211 |
| hsa-miR-4449 | 2.43E-08 | 1.01E-05 | 2.41E-08 | 0.638 (0.584; 0.692) | -0.168 |
| hsa-miR-532-3p | 5.54E-08 | 2.30E-05 | 1.55E-07 | 0.632 (0.577; 0.686) | -0.208 |
| hsa-miR-4284 | 5.76E-08 | 2.39E-05 | 1.50E-06 | 0.658 (0.605; 0.711) | -0.232 |
| hsa-miR-221-3p | 7.68E-08 | 3.18E-05 | 3.78E-10 | 0.653 (0.599; 0.707) | -0.295 |
| hsa-miR-222-3p | 8.27E-08 | 3.41E-05 | 2.33E-08 | 0.645 (0.592; 0.699) | -0.253 |
| hsa-miR-6803-3p | 8.52E-08 | 3.51E-05 | 3.08E-08 | 0.66 (0.607; 0.713) | -0.122 |
| hsa-miR-342-3p | 1.21E-07 | 4.98E-05 | 1.09E-06 | 0.591 (0.535; 0.646) | -0.184 |
| hsa-miR-4270 | 1.27E-07 | 5.21E-05 | 5.52E-08 | 0.656 (0.602; 0.709) | 0.159 |
| hsa-miR-532-5p | 1.37E-07 | 5.59E-05 | 1.13E-09 | 0.635 (0.581; 0.689) | -0.183 |
| hsa-miR-4443 | 1.41E-07 | 5.73E-05 | 1.94E-06 | 0.616 (0.561; 0.67) | 0.239 |
| hsa-let-7g-5p | 3.10E-07 | 1.26E-04 | 3.92E-11 | 0.662 (0.608; 0.716) | -0.199 |
| hsa-miR-101-3p | 3.82E-07 | 1.55E-04 | 1.87E-10 | 0.65 (0.597; 0.704) | -0.368 |
| hsa-miR-93-5p | 4.05E-07 | 1.64E-04 | 1.00E-10 | 0.668 (0.615; 0.721) | -0.262 |
| hsa-miR-6511b-3p | 4.78E-07 | 1.93E-04 | 4.49E-08 | 0.655 (0.601; 0.708) | -0.170 |
| hsa-miR-4291 | 6.66E-07 | 2.69E-04 | 3.48E-07 | 0.639 (0.584; 0.694) | -0.192 |
| hsa-miR-6791-5p | 6.71E-07 | 2.70E-04 | 8.44E-07 | 0.623 (0.569; 0.678) | -0.190 |
| hsa-miR-126-5p | 9.25E-07 | 3.71E-04 | 5.20E-08 | 0.631 (0.576; 0.685) | -0.218 |
| hsa-miR-491-5p | 1.24E-06 | 4.96E-04 | 1.38E-07 | 0.633 (0.579; 0.688) | 0.038 |
| hsa-miR-4485-3p | 1.75E-06 | 7.00E-04 | 1.58E-06 | 0.643 (0.589; 0.698) | 0.191 |
| hsa-miR-185-5p | 2.03E-06 | 8.07E-04 | 1.74E-08 | 0.625 (0.57; 0.68) | 0.127 |
| hsa-miR-20b-5p | 2.04E-06 | 8.09E-04 | 7.17E-09 | 0.635 (0.581; 0.689) | -0.222 |
| hsa-miR-25-3p | 4.26E-06 | 1.69E-03 | 3.07E-07 | 0.597 (0.541; 0.653) | 0.080 |
| hsa-miR-22-3p | 4.29E-06 | 1.69E-03 | 2.59E-06 | 0.584 (0.529; 0.64) | 0.137 |
| hsa-miR-7641 | 4.53E-06 | 1.78E-03 | 5.43E-06 | 0.621 (0.566; 0.675) | 0.240 |
| hsa-let-7a-5p | 6.85E-06 | 2.69E-03 | 2.25E-08 | 0.632 (0.577; 0.686) | -0.133 |
| hsa-miR-4318 | 7.20E-06 | 2.82E-03 | 4.26E-06 | 0.623 (0.569; 0.678) | -0.134 |
| hsa-miR-7-5p | 7.34E-06 | 2.87E-03 | 1.38E-09 | 0.639 (0.585; 0.693) | -0.273 |
| hsa-miR-30a-5p | 7.79E-06 | 3.04E-03 | 2.11E-07 | 0.617 (0.562; 0.673) | 0.203 |
| hsa-miR-155-5p | 8.55E-06 | 3.32E-03 | 3.23E-07 | 0.622 (0.567; 0.677) | -0.145 |
| hsa-let-7i-5p | 8.64E-06 | 3.35E-03 | 4.01E-09 | 0.622 (0.567; 0.678) | -0.222 |
| hsa-miR-660-5p | 9.42E-06 | 3.65E-03 | 6.21E-06 | 0.618 (0.563; 0.673) | -0.193 |
| hsa-miR-374b-5p | 9.68E-06 | 3.74E-03 | 5.33E-06 | 0.609 (0.554; 0.664) | -0.199 |
| hsa-miR-130a-3p | 1.03E-05 | 3.96E-03 | 4.44E-08 | 0.609 (0.554; 0.664) | -0.214 |
| hsa-miR-17-5p | 1.11E-05 | 4.26E-03 | 3.28E-08 | 0.629 (0.574; 0.684) | -0.218 |
| hsa-miR-1268a | 1.21E-05 | 4.63E-03 | 4.31E-05 | 0.636 (0.582; 0.69) | -0.151 |
| hsa-let-7f-5p | 1.22E-05 | 4.66E-03 | 1.60E-08 | 0.627 (0.572; 0.682) | -0.146 |
| hsa-miR-744-5p | 1.23E-05 | 4.69E-03 | 4.55E-05 | 0.631 (0.576; 0.686) | -0.123 |
| hsa-miR-145-5p | 1.79E-05 | 6.80E-03 | 8.68E-04 | 0.59 (0.534; 0.645) | -0.187 |
| hsa-miR-628-3p | 2.38E-05 | 9.02E-03 | 2.40E-06 | 0.604 (0.549; 0.66) | -0.111 |
| hsa-miR-20a-5p | 3.69E-05 | 1.40E-02 | 3.27E-07 | 0.623 (0.568; 0.678) | -0.223 |
| hsa-miR-125a-5p | 3.88E-05 | 1.46E-02 | 1.18E-04 | 0.583 (0.527; 0.639) | -0.192 |
| hsa-let-7d-3p | 3.94E-05 | 1.48E-02 | 1.19E-06 | 0.607 (0.552; 0.662) | 0.114 |
| hsa-let-7f-1-3p | 4.11E-05 | 1.54E-02 | 9.34E-06 | 0.656 (0.602; 0.709) | 0.083 |
| hsa-miR-6893-5p | 4.12E-05 | 1.54E-02 | 2.59E-05 | 0.59 (0.534; 0.645) | -0.140 |
| hsa-miR-1260a | 5.40E-05 | 2.02E-02 | 1.00E-04 | 0.582 (0.526; 0.638) | 0.193 |
| hsa-miR-30c-1-3p | 6.95E-05 | 2.59E-02 | 5.75E-04 | 0.567 (0.511; 0.623) | -0.033 |
| hsa-miR-374c-5p | 7.47E-05 | 2.77E-02 | 6.61E-05 | 0.596 (0.541; 0.652) | -0.183 |
| hsa-miR-484 | 7.47E-05 | 2.77E-02 | 3.97E-05 | 0.59 (0.534; 0.646) | -0.134 |
| hsa-miR-454-3p | 9.21E-05 | 3.40E-02 | 9.55E-07 | 0.609 (0.554; 0.665) | -0.247 |
| hsa-miR-500b-5p | 1.19E-04 | 4.39E-02 | 1.25E-04 | 0.586 (0.529; 0.642) | -0.132 |
| hsa-miR-374a-5p | 1.22E-04 | 4.49E-02 | 3.80E-05 | 0.601 (0.545; 0.656) | -0.260 |
| hsa-miR-365a-3p | 1.30E-04 | 4.77E-02 | 2.11E-04 | 0.581 (0.526; 0.637) | -0.139 |
| hsa-miR-4428 | 1.43E-04 | 5.21E-02 | 3.12E-04 | 0.588 (0.532; 0.644) | -0.127 |
| hsa-miR-15a-5p | 1.43E-04 | 5.21E-02 | 1.30E-06 | 0.611 (0.556; 0.667) | -0.188 |
| hsa-miR-874-3p | 1.49E-04 | 5.40E-02 | 8.18E-05 | 0.623 (0.568; 0.678) | -0.097 |
| hsa-miR-942-5p | 2.07E-04 | 7.48E-02 | 1.17E-04 | 0.593 (0.537; 0.649) | -0.162 |
| hsa-miR-142-5p | 2.47E-04 | 8.90E-02 | 1.27E-04 | 0.592 (0.537; 0.648) | -0.157 |
| hsa-miR-144-3p | 2.73E-04 | 9.84E-02 | 2.36E-06 | 0.622 (0.567; 0.677) | -0.362 |
| hsa-miR-193a-5p | 2.75E-04 | 9.88E-02 | 2.44E-03 | 0.58 (0.524; 0.636) | 0.080 |
| hsa-let-7c-5p | 2.84E-04 | 1.02E-01 | 8.32E-07 | 0.617 (0.563; 0.672) | -0.206 |
| hsa-miR-181b-5p | 3.29E-04 | 1.18E-01 | 5.32E-05 | 0.619 (0.564; 0.674) | -0.135 |
| hsa-miR-6875-5p | 3.52E-04 | 1.25E-01 | 1.08E-03 | 0.607 (0.551; 0.662) | 0.123 |
| hsa-miR-107 | 4.40E-04 | 1.56E-01 | 3.09E-07 | 0.604 (0.548; 0.659) | -0.107 |
| hsa-miR-6752-3p | 4.80E-04 | 1.70E-01 | 2.55E-04 | 0.602 (0.547; 0.657) | 0.058 |
| hsa-miR-8069 | 5.13E-04 | 1.81E-01 | 2.13E-02 | 0.563 (0.506; 0.619) | -0.095 |
| hsa-miR-7704 | 5.35E-04 | 1.88E-01 | 5.53E-04 | 0.569 (0.513; 0.625) | -0.109 |
| hsa-miR-6068 | 6.33E-04 | 2.22E-01 | 1.05E-03 | 0.573 (0.517; 0.629) | -0.112 |
| hsa-miR-5100 | 7.46E-04 | 2.61E-01 | 1.61E-03 | 0.562 (0.506; 0.618) | 0.210 |
| hsa-miR-18b-5p | 8.04E-04 | 2.81E-01 | 1.59E-05 | 0.603 (0.547; 0.658) | -0.196 |
| hsa-miR-4685-5p | 8.18E-04 | 2.85E-01 | 1.36E-03 | 0.579 (0.523; 0.635) | 0.221 |
| hsa-miR-33b-3p | 8.20E-04 | 2.85E-01 | 2.66E-04 | 0.595 (0.539; 0.65) | 0.075 |
| hsa-miR-6090 | 8.45E-04 | 2.92E-01 | 5.69E-05 | 0.587 (0.531; 0.643) | 0.102 |
| hsa-miR-3200-5p | 8.55E-04 | 2.95E-01 | 8.99E-06 | 0.583 (0.527; 0.638) | -0.168 |
| hsa-miR-6088 | 8.64E-04 | 2.97E-01 | 8.01E-05 | 0.603 (0.548; 0.659) | 0.076 |
| hsa-miR-24-3p | 9.84E-04 | 3.37E-01 | 1.30E-03 | 0.566 (0.509; 0.622) | -0.076 |
| hsa-miR-7108-5p | 1.00E-03 | 3.44E-01 | 2.74E-03 | 0.608 (0.553; 0.663) | -0.116 |
| hsa-let-7d-5p | 1.03E-03 | 3.50E-01 | 4.82E-06 | 0.591 (0.536; 0.647) | -0.096 |
| hsa-miR-196b-5p | 1.04E-03 | 3.52E-01 | 2.10E-04 | 0.571 (0.515; 0.627) | -0.153 |
| hsa-miR-29a-3p | 1.17E-03 | 3.95E-01 | 2.29E-03 | 0.558 (0.502; 0.615) | -0.138 |
| hsa-miR-132-3p | 1.28E-03 | 4.32E-01 | 4.76E-04 | 0.592 (0.536; 0.648) | -0.095 |
| hsa-miR-4497 | 1.30E-03 | 4.37E-01 | 1.03E-03 | 0.569 (0.513; 0.626) | -0.103 |
| hsa-miR-4732-5p | 1.36E-03 | 4.57E-01 | 1.70E-04 | 0.585 (0.529; 0.64) | -0.117 |
| hsa-miR-194-5p | 1.53E-03 | 5.11E-01 | 1.37E-03 | 0.58 (0.524; 0.636) | -0.134 |
| hsa-miR-183-5p | 1.62E-03 | 5.41E-01 | 1.63E-03 | 0.586 (0.529; 0.642) | 0.153 |
| hsa-miR-610 | 1.96E-03 | 6.54E-01 | 5.02E-04 | 0.579 (0.523; 0.635) | -0.069 |
| hsa-miR-5001-5p | 1.97E-03 | 6.55E-01 | 3.59E-03 | 0.557 (0.501; 0.613) | -0.091 |
| hsa-miR-151b | 2.08E-03 | 6.88E-01 | 8.02E-04 | 0.562 (0.506; 0.619) | -0.081 |
| hsa-miR-4746-3p | 2.15E-03 | 7.11E-01 | 4.30E-03 | 0.549 (0.493; 0.606) | -0.070 |
| hsa-miR-103a-2-5p | 2.17E-03 | 7.15E-01 | 3.31E-04 | 0.595 (0.539; 0.651) | -0.098 |
| hsa-miR-149-5p | 2.25E-03 | 7.39E-01 | 1.18E-03 | 0.599 (0.544; 0.655) | 0.061 |
| hsa-miR-933 | 2.41E-03 | 7.89E-01 | 3.21E-03 | 0.609 (0.554; 0.664) | 0.039 |
| hsa-miR-144-5p | 2.46E-03 | 8.01E-01 | 5.84E-06 | 0.61 (0.555; 0.665) | -0.238 |
| hsa-miR-6858-3p | 2.53E-03 | 8.22E-01 | 2.12E-03 | 0.591 (0.535; 0.646) | 0.068 |
| hsa-miR-1255a | 2.54E-03 | 8.22E-01 | 2.14E-02 | 0.548 (0.492; 0.605) | 0.067 |
| hsa-miR-574-3p | 2.55E-03 | 8.22E-01 | 1.81E-03 | 0.594 (0.539; 0.65) | -0.103 |
| hsa-miR-762 | 2.85E-03 | 9.17E-01 | 3.37E-03 | 0.592 (0.537; 0.648) | 0.084 |
| hsa-miR-629-3p | 3.23E-03 | 1.00E+00 | 1.76E-02 | 0.569 (0.513; 0.625) | -0.096 |
| hsa-miR-18a-5p | 3.29E-03 | 1.00E+00 | 1.20E-04 | 0.584 (0.528; 0.64) | -0.186 |
| hsa-miR-5787 | 3.35E-03 | 1.00E+00 | 8.44E-03 | 0.62 (0.564; 0.675) | 0.126 |
| hsa-miR-4739 | 3.56E-03 | 1.00E+00 | 2.16E-03 | 0.581 (0.525; 0.637) | -0.091 |
| hsa-miR-3614-5p | 3.56E-03 | 1.00E+00 | 1.35E-03 | 0.604 (0.549; 0.659) | 0.053 |
| hsa-miR-590-5p | 3.61E-03 | 1.00E+00 | 2.84E-04 | 0.587 (0.531; 0.643) | -0.199 |
| hsa-miR-642b-3p | 3.69E-03 | 1.00E+00 | 5.92E-03 | 0.576 (0.52; 0.632) | 0.099 |
| hsa-miR-4323 | 3.86E-03 | 1.00E+00 | 3.60E-02 | 0.542 (0.486; 0.599) | -0.119 |
| hsa-miR-1207-5p | 3.95E-03 | 1.00E+00 | 8.01E-03 | 0.611 (0.556; 0.666) | -0.081 |
| hsa-miR-339-5p | 3.96E-03 | 1.00E+00 | 1.30E-03 | 0.597 (0.541; 0.653) | -0.147 |
| hsa-miR-320a | 4.01E-03 | 1.00E+00 | 3.64E-04 | 0.576 (0.52; 0.632) | -0.120 |
| hsa-miR-3200-3p | 4.10E-03 | 1.00E+00 | 4.41E-03 | 0.577 (0.522; 0.633) | -0.116 |
| hsa-miR-6760-3p | 4.25E-03 | 1.00E+00 | 1.63E-03 | 0.599 (0.543; 0.654) | 0.050 |
| hsa-miR-7974 | 4.29E-03 | 1.00E+00 | 2.44E-03 | 0.607 (0.552; 0.662) | 0.055 |
| hsa-miR-4728-5p | 4.30E-03 | 1.00E+00 | 6.06E-03 | 0.541 (0.485; 0.598) | -0.098 |
| hsa-miR-324-5p | 4.39E-03 | 1.00E+00 | 4.16E-04 | 0.579 (0.523; 0.635) | 0.082 |
| hsa-miR-5006-5p | 4.79E-03 | 1.00E+00 | 3.23E-03 | 0.548 (0.491; 0.604) | -0.097 |
| hsa-miR-3653-3p | 5.02E-03 | 1.00E+00 | 1.09E-03 | 0.531 (0.474; 0.588) | -0.159 |
| hsa-miR-629-5p | 5.02E-03 | 1.00E+00 | 4.95E-03 | 0.581 (0.525; 0.636) | -0.093 |
| hsa-miR-338-3p | 5.16E-03 | 1.00E+00 | 7.58E-04 | 0.59 (0.535; 0.646) | 0.138 |
| hsa-miR-4299 | 5.30E-03 | 1.00E+00 | 6.76E-03 | 0.571 (0.514; 0.627) | -0.110 |
| hsa-miR-92b-3p | 5.56E-03 | 1.00E+00 | 8.54E-03 | 0.592 (0.536; 0.648) | -0.049 |
| hsa-miR-195-5p | 6.66E-03 | 1.00E+00 | 1.86E-03 | 0.559 (0.502; 0.615) | -0.126 |
| hsa-miR-6731-3p | 6.72E-03 | 1.00E+00 | 2.97E-03 | 0.59 (0.534; 0.645) | 0.051 |
| hsa-miR-99b-5p | 7.01E-03 | 1.00E+00 | 3.89E-02 | 0.532 (0.475; 0.588) | -0.130 |
| hsa-miR-362-5p | 7.28E-03 | 1.00E+00 | 1.37E-02 | 0.533 (0.477; 0.59) | 0.084 |
| hsa-miR-106b-5p | 7.38E-03 | 1.00E+00 | 1.68E-04 | 0.588 (0.532; 0.644) | -0.139 |
| hsa-miR-6865-3p | 8.47E-03 | 1.00E+00 | 4.46E-03 | 0.586 (0.53; 0.642) | 0.063 |
| hsa-miR-4515 | 9.16E-03 | 1.00E+00 | 6.65E-03 | 0.594 (0.538; 0.65) | 0.068 |
| hsa-miR-15b-3p | 9.71E-03 | 1.00E+00 | 1.01E-03 | 0.557 (0.5; 0.613) | 0.075 |
| hsa-miR-296-5p | 9.85E-03 | 1.00E+00 | 1.99E-03 | 0.573 (0.517; 0.629) | -0.086 |
| hsa-miR-103a-3p | 1.06E-02 | 1.00E+00 | 2.72E-04 | 0.582 (0.527; 0.638) | -0.078 |
| hsa-miR-4459 | 1.11E-02 | 1.00E+00 | 5.38E-03 | 0.549 (0.492; 0.606) | -0.090 |
| hsa-miR-199a-5p | 1.12E-02 | 1.00E+00 | 2.21E-01 | 0.524 (0.467; 0.58) | -0.145 |
| hsa-miR-3613-3p | 1.25E-02 | 1.00E+00 | 6.48E-03 | 0.576 (0.52; 0.631) | 0.040 |
| hsa-miR-19a-3p | 1.28E-02 | 1.00E+00 | 9.52E-03 | 0.565 (0.509; 0.621) | -0.126 |
| hsa-miR-664b-5p | 1.35E-02 | 1.00E+00 | 2.70E-02 | 0.582 (0.526; 0.638) | 0.182 |
| hsa-miR-664a-5p | 1.39E-02 | 1.00E+00 | 7.24E-02 | 0.57 (0.513; 0.626) | 0.102 |
| hsa-miR-4793-5p | 1.52E-02 | 1.00E+00 | 7.02E-03 | 0.576 (0.52; 0.632) | -0.056 |
| hsa-miR-1268b | 1.55E-02 | 1.00E+00 | 1.80E-02 | 0.574 (0.519; 0.63) | -0.093 |
| hsa-miR-641 | 1.55E-02 | 1.00E+00 | 1.02E-03 | 0.569 (0.513; 0.626) | 0.066 |
| hsa-miR-3135b | 1.60E-02 | 1.00E+00 | 5.40E-03 | 0.617 (0.562; 0.672) | -0.208 |
| hsa-miR-183-3p | 1.69E-02 | 1.00E+00 | 1.70E-02 | 0.57 (0.513; 0.626) | 0.084 |
| hsa-miR-2116-3p | 1.81E-02 | 1.00E+00 | 1.13E-02 | 0.589 (0.533; 0.645) | 0.048 |
| hsa-miR-1202 | 1.83E-02 | 1.00E+00 | 4.92E-03 | 0.539 (0.482; 0.595) | 0.083 |
| hsa-miR-486-5p | 1.88E-02 | 1.00E+00 | 8.74E-03 | 0.571 (0.515; 0.627) | 0.034 |
| hsa-miR-6749-5p | 2.05E-02 | 1.00E+00 | 9.87E-03 | 0.546 (0.489; 0.602) | 0.109 |
| hsa-miR-575 | 2.13E-02 | 1.00E+00 | 2.87E-02 | 0.578 (0.522; 0.634) | 0.072 |
| hsa-miR-200c-3p | 2.24E-02 | 1.00E+00 | 4.54E-03 | 0.568 (0.512; 0.624) | -0.057 |
| hsa-miR-6717-5p | 2.34E-02 | 1.00E+00 | 8.28E-02 | 0.555 (0.499; 0.612) | 0.073 |
| hsa-miR-5194 | 2.45E-02 | 1.00E+00 | 5.35E-02 | 0.556 (0.499; 0.612) | 0.071 |
| hsa-miR-6724-5p | 2.55E-02 | 1.00E+00 | 1.20E-02 | 0.561 (0.505; 0.617) | -0.078 |
| hsa-miR-4281 | 2.56E-02 | 1.00E+00 | 3.11E-02 | 0.547 (0.49; 0.603) | -0.073 |
| hsa-let-7b-3p | 2.59E-02 | 1.00E+00 | 1.13E-02 | 0.587 (0.531; 0.643) | 0.048 |
| hsa-miR-148a-3p | 2.61E-02 | 1.00E+00 | 8.30E-03 | 0.559 (0.503; 0.615) | -0.113 |
| hsa-miR-4505 | 2.79E-02 | 1.00E+00 | 2.66E-02 | 0.555 (0.499; 0.612) | 0.075 |
| hsa-miR-2861 | 2.81E-02 | 1.00E+00 | 3.84E-02 | 0.549 (0.493; 0.606) | -0.077 |
| hsa-miR-574-5p | 3.02E-02 | 1.00E+00 | 2.94E-02 | 0.601 (0.545; 0.656) | -0.107 |
| hsa-miR-146a-5p | 3.02E-02 | 1.00E+00 | 4.68E-02 | 0.539 (0.483; 0.596) | -0.073 |
| hsa-miR-664b-3p | 3.02E-02 | 1.00E+00 | 2.92E-02 | 0.564 (0.508; 0.62) | -0.060 |
| hsa-miR-7975 | 3.05E-02 | 1.00E+00 | 4.97E-02 | 0.523 (0.467; 0.58) | 0.064 |
| hsa-miR-6813-3p | 3.06E-02 | 1.00E+00 | 2.16E-02 | 0.562 (0.506; 0.618) | 0.043 |
| hsa-miR-6891-5p | 3.16E-02 | 1.00E+00 | 2.09E-02 | 0.567 (0.511; 0.623) | -0.063 |
| hsa-miR-100-5p | 3.19E-02 | 1.00E+00 | 2.36E-02 | 0.543 (0.486; 0.599) | 0.362 |
| hsa-miR-210-3p | 3.23E-02 | 1.00E+00 | 1.30E-02 | 0.556 (0.5; 0.613) | -0.130 |
| hsa-miR-181a-2-3p | 3.25E-02 | 1.00E+00 | 1.36E-01 | 0.538 (0.482; 0.595) | -0.050 |
| hsa-miR-4732-3p | 3.31E-02 | 1.00E+00 | 5.97E-02 | 0.571 (0.515; 0.627) | -0.087 |
| hsa-miR-6763-3p | 3.33E-02 | 1.00E+00 | 2.07E-02 | 0.57 (0.514; 0.626) | 0.037 |
| hsa-miR-361-5p | 3.36E-02 | 1.00E+00 | 4.32E-01 | 0.502 (0.441; 0.555) | -0.067 |
| hsa-miR-1255b-5p | 3.38E-02 | 1.00E+00 | 2.26E-03 | 0.573 (0.517; 0.629) | -0.064 |
| hsa-miR-29c-3p | 3.44E-02 | 1.00E+00 | 1.52E-02 | 0.559 (0.503; 0.616) | -0.112 |
| hsa-miR-6757-5p | 3.50E-02 | 1.00E+00 | 4.47E-02 | 0.57 (0.514; 0.626) | 0.056 |
| hsa-miR-6512-5p | 3.53E-02 | 1.00E+00 | 6.17E-02 | 0.576 (0.519; 0.632) | 0.075 |
| hsa-miR-628-5p | 3.68E-02 | 1.00E+00 | 9.40E-02 | 0.562 (0.506; 0.618) | 0.062 |
| hsa-miR-6780b-5p | 4.06E-02 | 1.00E+00 | 9.57E-02 | 0.548 (0.492; 0.604) | 0.070 |
| hsa-miR-371b-5p | 4.24E-02 | 1.00E+00 | 5.41E-02 | 0.529 (0.472; 0.586) | -0.069 |
| hsa-miR-6869-5p | 4.38E-02 | 1.00E+00 | 3.31E-02 | 0.543 (0.486; 0.599) | -0.072 |
| hsa-miR-6800-5p | 4.58E-02 | 1.00E+00 | 1.92E-02 | 0.55 (0.494; 0.607) | 0.054 |
| hsa-miR-96-5p | 4.78E-02 | 1.00E+00 | 1.35E-02 | 0.534 (0.478; 0.591) | -0.119 |
| hsa-miR-3125 | 4.81E-02 | 1.00E+00 | 6.01E-02 | 0.553 (0.496; 0.61) | 0.080 |
| hsa-miR-29c-5p | 4.87E-02 | 1.00E+00 | 8.98E-03 | 0.565 (0.509; 0.621) | 0.073 |
| hsa-miR-3940-5p | 4.91E-02 | 1.00E+00 | 9.82E-03 | 0.571 (0.515; 0.627) | 0.055 |
| hsa-miR-494-3p | 4.97E-02 | 1.00E+00 | 5.48E-02 | 0.523 (0.466; 0.579) | -0.112 |
| hsa-miR-324-3p | 5.15E-02 | 1.00E+00 | 5.67E-02 | 0.551 (0.495; 0.607) | -0.047 |
| hsa-miR-3665 | 5.39E-02 | 1.00E+00 | 1.27E-02 | 0.553 (0.497; 0.61) | 0.065 |
| hsa-miR-6789-5p | 5.40E-02 | 1.00E+00 | 1.80E-01 | 0.529 (0.472; 0.585) | -0.094 |
| hsa-miR-1825 | 5.54E-02 | 1.00E+00 | 3.73E-02 | 0.546 (0.489; 0.602) | 0.051 |
| hsa-miR-642a-3p | 5.70E-02 | 1.00E+00 | 4.50E-02 | 0.553 (0.497; 0.61) | 0.066 |
| hsa-miR-638 | 5.92E-02 | 1.00E+00 | 8.61E-02 | 0.545 (0.489; 0.601) | -0.053 |
| hsa-miR-5088-5p | 6.33E-02 | 1.00E+00 | 1.17E-01 | 0.549 (0.493; 0.606) | 0.061 |
| hsa-miR-500a-5p | 6.51E-02 | 1.00E+00 | 4.01E-02 | 0.566 (0.51; 0.622) | -0.073 |
| hsa-miR-151a-3p | 6.68E-02 | 1.00E+00 | 1.37E-02 | 0.549 (0.493; 0.606) | 0.069 |
| hsa-miR-8063 | 7.02E-02 | 1.00E+00 | 5.83E-02 | 0.534 (0.477; 0.59) | -0.062 |
| hsa-miR-197-3p | 7.06E-02 | 1.00E+00 | 1.15E-01 | 0.579 (0.523; 0.635) | -0.063 |
| hsa-miR-6779-5p | 7.15E-02 | 1.00E+00 | 2.13E-02 | 0.574 (0.518; 0.63) | 0.043 |
| hsa-miR-769-5p | 7.20E-02 | 1.00E+00 | 2.33E-02 | 0.555 (0.499; 0.612) | 0.062 |
| hsa-miR-3195 | 7.53E-02 | 1.00E+00 | 7.83E-02 | 0.546 (0.489; 0.603) | 0.070 |
| hsa-miR-4758-3p | 7.55E-02 | 1.00E+00 | 5.73E-02 | 0.563 (0.507; 0.619) | 0.036 |
| hsa-miR-7152-3p | 7.55E-02 | 1.00E+00 | 1.23E-01 | 0.574 (0.518; 0.63) | 0.069 |
| hsa-miR-6777-3p | 7.64E-02 | 1.00E+00 | 5.43E-02 | 0.577 (0.521; 0.633) | 0.029 |
| hsa-miR-1305 | 8.13E-02 | 1.00E+00 | 1.52E-01 | 0.534 (0.478; 0.591) | 0.058 |
| hsa-miR-3198 | 8.45E-02 | 1.00E+00 | 1.76E-01 | 0.544 (0.487; 0.6) | 0.064 |
| hsa-miR-1238-3p | 8.64E-02 | 1.00E+00 | 7.15E-02 | 0.565 (0.509; 0.621) | 0.040 |
| hsa-miR-4665-3p | 8.66E-02 | 1.00E+00 | 3.26E-02 | 0.559 (0.503; 0.616) | 0.043 |
| hsa-miR-3605-3p | 8.68E-02 | 1.00E+00 | 6.07E-02 | 0.529 (0.473; 0.586) | -0.043 |
| hsa-miR-7110-5p | 8.97E-02 | 1.00E+00 | 1.29E-01 | 0.519 (0.463; 0.576) | 0.059 |
| hsa-miR-4669 | 9.01E-02 | 1.00E+00 | 7.10E-02 | 0.567 (0.511; 0.623) | -0.061 |
| hsa-miR-4659a-3p | 9.25E-02 | 1.00E+00 | 2.71E-01 | 0.522 (0.465; 0.579) | -0.076 |
| hsa-miR-6073 | 9.26E-02 | 1.00E+00 | 1.33E-03 | 0.585 (0.529; 0.64) | -0.070 |
| hsa-miR-99a-5p | 9.37E-02 | 1.00E+00 | 1.55E-01 | 0.532 (0.475; 0.588) | -0.092 |
| hsa-miR-23a-3p | 9.42E-02 | 1.00E+00 | 2.02E-03 | 0.58 (0.524; 0.636) | 0.063 |
| hsa-miR-424-5p | 9.56E-02 | 1.00E+00 | 7.00E-03 | 0.54 (0.484; 0.597) | -0.100 |
| hsa-let-7b-5p | 9.90E-02 | 1.00E+00 | 7.42E-03 | 0.562 (0.505; 0.618) | -0.095 |
| hsa-miR-16-5p | 1.00E-01 | 1.00E+00 | 5.88E-03 | 0.555 (0.499; 0.612) | -0.054 |
| hsa-miR-1973 | 1.01E-01 | 1.00E+00 | 1.59E-01 | 0.574 (0.518; 0.63) | 0.068 |
| hsa-miR-501-5p | 1.03E-01 | 1.00E+00 | 5.39E-02 | 0.537 (0.48; 0.593) | -0.050 |
| hsa-miR-425-5p | 1.06E-01 | 1.00E+00 | 1.49E-02 | 0.561 (0.505; 0.617) | 0.036 |
| hsa-miR-6889-3p | 1.07E-01 | 1.00E+00 | 7.45E-02 | 0.552 (0.496; 0.608) | 0.036 |
| hsa-miR-4649-3p | 1.11E-01 | 1.00E+00 | 6.55E-02 | 0.548 (0.492; 0.604) | 0.043 |
| hsa-miR-92a-3p | 1.14E-01 | 1.00E+00 | 8.58E-02 | 0.53 (0.473; 0.587) | 0.033 |
| hsa-miR-1281 | 1.15E-01 | 1.00E+00 | 8.00E-02 | 0.544 (0.488; 0.6) | 0.045 |
| hsa-miR-320d | 1.15E-01 | 1.00E+00 | 1.78E-01 | 0.535 (0.479; 0.591) | 0.047 |
| hsa-miR-30c-5p | 1.19E-01 | 1.00E+00 | 4.94E-03 | 0.561 (0.505; 0.618) | 0.050 |
| hsa-miR-3162-3p | 1.20E-01 | 1.00E+00 | 7.97E-02 | 0.534 (0.477; 0.59) | 0.049 |
| hsa-miR-6727-5p | 1.20E-01 | 1.00E+00 | 1.04E-01 | 0.541 (0.484; 0.598) | 0.065 |
| hsa-miR-215-5p | 1.21E-01 | 1.00E+00 | 1.85E-01 | 0.522 (0.465; 0.578) | -0.069 |
| hsa-miR-766-3p | 1.23E-01 | 1.00E+00 | 4.99E-01 | 0.525 (0.469; 0.582) | -0.060 |
| hsa-miR-6851-3p | 1.23E-01 | 1.00E+00 | 1.15E-01 | 0.557 (0.501; 0.614) | 0.029 |
| hsa-miR-320c | 1.25E-01 | 1.00E+00 | 2.26E-01 | 0.531 (0.474; 0.587) | 0.046 |
| hsa-miR-501-3p | 1.29E-01 | 1.00E+00 | 9.34E-02 | 0.536 (0.479; 0.593) | -0.040 |
| hsa-miR-4687-3p | 1.31E-01 | 1.00E+00 | 7.65E-02 | 0.547 (0.491; 0.604) | 0.038 |
| hsa-miR-502-5p | 1.31E-01 | 1.00E+00 | 2.11E-01 | 0.525 (0.468; 0.582) | -0.064 |
| hsa-miR-550b-2-5p | 1.32E-01 | 1.00E+00 | 4.55E-02 | 0.566 (0.51; 0.622) | 0.041 |
| hsa-miR-4721 | 1.34E-01 | 1.00E+00 | 7.75E-02 | 0.526 (0.469; 0.582) | -0.065 |
| hsa-miR-378a-5p | 1.35E-01 | 1.00E+00 | 1.98E-02 | 0.548 (0.492; 0.605) | 0.065 |
| hsa-miR-6785-5p | 1.35E-01 | 1.00E+00 | 1.41E-01 | 0.511 (0.454; 0.568) | -0.077 |
| hsa-miR-191-3p | 1.35E-01 | 1.00E+00 | 1.23E-01 | 0.557 (0.501; 0.613) | 0.033 |
| hsa-miR-409-3p | 1.36E-01 | 1.00E+00 | 4.82E-01 | 0.51 (0.453; 0.566) | -0.104 |
| hsa-miR-6515-3p | 1.39E-01 | 1.00E+00 | 8.71E-02 | 0.566 (0.51; 0.622) | 0.027 |
| hsa-miR-6737-3p | 1.40E-01 | 1.00E+00 | 9.88E-02 | 0.551 (0.495; 0.608) | 0.037 |
| hsa-miR-6780a-5p | 1.43E-01 | 1.00E+00 | 2.81E-01 | 0.536 (0.48; 0.593) | 0.055 |
| hsa-miR-6880-3p | 1.44E-01 | 1.00E+00 | 9.86E-02 | 0.546 (0.489; 0.602) | 0.024 |
| hsa-miR-4653-3p | 1.45E-01 | 1.00E+00 | 6.55E-02 | 0.545 (0.489; 0.602) | -0.049 |
| hsa-miR-625-5p | 1.49E-01 | 1.00E+00 | 2.93E-01 | 0.543 (0.486; 0.6) | -0.063 |
| hsa-miR-5581-5p | 1.51E-01 | 1.00E+00 | 2.21E-01 | 0.535 (0.478; 0.592) | 0.056 |
| hsa-miR-340-5p | 1.57E-01 | 1.00E+00 | 1.18E-01 | 0.546 (0.489; 0.602) | -0.084 |
| hsa-miR-335-5p | 1.62E-01 | 1.00E+00 | 2.78E-02 | 0.553 (0.496; 0.609) | 0.065 |
| hsa-miR-6797-3p | 1.64E-01 | 1.00E+00 | 1.06E-01 | 0.542 (0.485; 0.598) | 0.031 |
| hsa-miR-1288-3p | 1.74E-01 | 1.00E+00 | 2.91E-01 | 0.534 (0.478; 0.591) | 0.048 |
| hsa-miR-148b-3p | 1.81E-01 | 1.00E+00 | 1.97E-01 | 0.53 (0.473; 0.587) | -0.054 |
| hsa-miR-1285-3p | 1.86E-01 | 1.00E+00 | 4.65E-01 | 0.524 (0.468; 0.581) | -0.031 |
| hsa-miR-6131 | 1.89E-01 | 1.00E+00 | 3.99E-01 | 0.52 (0.464; 0.577) | 0.043 |
| hsa-miR-3163 | 1.89E-01 | 1.00E+00 | 5.16E-02 | 0.515 (0.459; 0.572) | -0.043 |
| hsa-miR-495-3p | 1.91E-01 | 1.00E+00 | 5.62E-01 | 0.5 (0.444; 0.557) | -0.079 |
| hsa-miR-6812-3p | 1.92E-01 | 1.00E+00 | 2.11E-01 | 0.545 (0.488; 0.601) | -0.023 |
| hsa-miR-1249-3p | 1.98E-01 | 1.00E+00 | 1.85E-01 | 0.516 (0.459; 0.573) | -0.028 |
| hsa-miR-1260b | 2.01E-01 | 1.00E+00 | 1.56E-01 | 0.508 (0.451; 0.564) | 0.051 |
| hsa-miR-148b-5p | 2.07E-01 | 1.00E+00 | 1.78E-01 | 0.527 (0.47; 0.584) | 0.041 |
| hsa-miR-598-3p | 2.07E-01 | 1.00E+00 | 1.44E-01 | 0.541 (0.485; 0.598) | -0.040 |
| hsa-miR-139-5p | 2.09E-01 | 1.00E+00 | 4.08E-02 | 0.551 (0.494; 0.608) | 0.038 |
| hsa-miR-4436b-5p | 2.13E-01 | 1.00E+00 | 2.08E-01 | 0.513 (0.456; 0.569) | 0.042 |
| hsa-miR-7150 | 2.21E-01 | 1.00E+00 | 2.09E-01 | 0.501 (0.445; 0.558) | -0.038 |
| hsa-miR-1306-5p | 2.24E-01 | 1.00E+00 | 2.63E-01 | 0.559 (0.503; 0.615) | -0.043 |
| hsa-miR-320e | 2.29E-01 | 1.00E+00 | 3.58E-01 | 0.525 (0.469; 0.582) | 0.036 |
| hsa-miR-140-5p | 2.37E-01 | 1.00E+00 | 1.62E-01 | 0.537 (0.481; 0.594) | -0.058 |
| hsa-miR-4769-3p | 2.41E-01 | 1.00E+00 | 1.70E-01 | 0.528 (0.471; 0.584) | 0.022 |
| hsa-miR-451a | 2.45E-01 | 1.00E+00 | 3.55E-01 | 0.544 (0.487; 0.601) | -0.009 |
| hsa-miR-1915-3p | 2.47E-01 | 1.00E+00 | 2.58E-01 | 0.523 (0.466; 0.579) | -0.041 |
| hsa-miR-627-5p | 2.49E-01 | 1.00E+00 | 2.53E-01 | 0.525 (0.468; 0.583) | 0.052 |
| hsa-miR-6785-3p | 2.51E-01 | 1.00E+00 | 2.27E-01 | 0.525 (0.468; 0.581) | 0.021 |
| hsa-miR-4787-3p | 2.53E-01 | 1.00E+00 | 1.78E-01 | 0.54 (0.483; 0.596) | 0.026 |
| hsa-miR-3656 | 2.53E-01 | 1.00E+00 | 5.06E-01 | 0.509 (0.452; 0.565) | -0.054 |
| hsa-miR-4313 | 2.56E-01 | 1.00E+00 | 1.74E-01 | 0.544 (0.488; 0.601) | 0.030 |
| hsa-miR-1914-3p | 2.57E-01 | 1.00E+00 | 4.78E-01 | 0.534 (0.478; 0.591) | 0.042 |
| hsa-miR-4664-3p | 2.59E-01 | 1.00E+00 | 2.41E-01 | 0.528 (0.472; 0.585) | -0.023 |
| hsa-miR-199a-3p | 2.60E-01 | 1.00E+00 | 3.67E-01 | 0.518 (0.461; 0.575) | -0.070 |
| hsa-miR-4485-5p | 2.61E-01 | 1.00E+00 | 3.49E-01 | 0.552 (0.496; 0.609) | 0.054 |
| hsa-miR-1228-3p | 2.62E-01 | 1.00E+00 | 2.07E-01 | 0.542 (0.486; 0.599) | 0.023 |
| hsa-miR-28-5p | 2.64E-01 | 1.00E+00 | 1.52E-01 | 0.539 (0.482; 0.596) | 0.037 |
| hsa-miR-191-5p | 2.65E-01 | 1.00E+00 | 2.34E-01 | 0.549 (0.492; 0.605) | -0.033 |
| hsa-miR-5739 | 2.71E-01 | 1.00E+00 | 4.05E-01 | 0.509 (0.452; 0.566) | 0.063 |
| hsa-miR-1539 | 2.72E-01 | 1.00E+00 | 3.33E-01 | 0.531 (0.474; 0.588) | 0.022 |
| hsa-miR-942-3p | 2.76E-01 | 1.00E+00 | 4.38E-01 | 0.531 (0.474; 0.588) | 0.037 |
| hsa-miR-6767-5p | 2.78E-01 | 1.00E+00 | 4.64E-01 | 0.519 (0.462; 0.575) | 0.038 |
| hsa-miR-6879-5p | 2.80E-01 | 1.00E+00 | 4.50E-01 | 0.518 (0.461; 0.574) | 0.040 |
| hsa-miR-4530 | 2.91E-01 | 1.00E+00 | 1.89E-01 | 0.538 (0.482; 0.595) | 0.039 |
| hsa-miR-30e-3p | 2.92E-01 | 1.00E+00 | 4.30E-02 | 0.541 (0.484; 0.598) | 0.054 |
| hsa-miR-199b-5p | 2.93E-01 | 1.00E+00 | 4.89E-02 | 0.547 (0.491; 0.604) | 0.061 |
| hsa-miR-181a-5p | 3.02E-01 | 1.00E+00 | 1.41E-01 | 0.545 (0.488; 0.601) | -0.058 |
| hsa-miR-654-3p | 3.08E-01 | 1.00E+00 | 9.77E-01 | 0.515 (0.428; 0.542) | -0.062 |
| hsa-miR-1246 | 3.08E-01 | 1.00E+00 | 7.69E-01 | 0.51 (0.453; 0.567) | 0.050 |
| hsa-miR-140-3p | 3.12E-01 | 1.00E+00 | 1.34E-02 | 0.583 (0.527; 0.639) | 0.028 |
| hsa-miR-423-5p | 3.24E-01 | 1.00E+00 | 1.90E-01 | 0.535 (0.478; 0.592) | -0.032 |
| hsa-miR-6870-3p | 3.33E-01 | 1.00E+00 | 3.30E-01 | 0.52 (0.463; 0.576) | 0.012 |
| hsa-miR-7114-5p | 3.39E-01 | 1.00E+00 | 4.19E-01 | 0.504 (0.447; 0.56) | 0.050 |
| hsa-miR-550a-5p | 3.42E-01 | 1.00E+00 | 1.84E-01 | 0.564 (0.508; 0.621) | 0.019 |
| hsa-miR-6069 | 3.62E-01 | 1.00E+00 | 2.85E-01 | 0.536 (0.48; 0.593) | 0.020 |
| hsa-miR-6734-5p | 3.64E-01 | 1.00E+00 | 5.42E-01 | 0.512 (0.455; 0.569) | 0.038 |
| hsa-miR-3679-5p | 3.68E-01 | 1.00E+00 | 5.58E-01 | 0.505 (0.438; 0.552) | 0.026 |
| hsa-miR-6089 | 3.70E-01 | 1.00E+00 | 1.86E-01 | 0.527 (0.471; 0.584) | 0.029 |
| hsa-miR-6132 | 3.70E-01 | 1.00E+00 | 2.20E-01 | 0.509 (0.434; 0.547) | -0.042 |
| hsa-miR-6800-3p | 3.71E-01 | 1.00E+00 | 3.07E-01 | 0.544 (0.488; 0.601) | 0.020 |
| hsa-miR-1271-5p | 3.80E-01 | 1.00E+00 | 9.28E-01 | 0.526 (0.469; 0.582) | -0.035 |
| hsa-miR-340-3p | 3.82E-01 | 1.00E+00 | 7.71E-02 | 0.543 (0.486; 0.6) | 0.056 |
| hsa-miR-4741 | 3.94E-01 | 1.00E+00 | 3.11E-01 | 0.508 (0.451; 0.565) | -0.036 |
| hsa-miR-142-3p | 3.96E-01 | 1.00E+00 | 2.26E-01 | 0.527 (0.47; 0.584) | 0.048 |
| hsa-miR-485-3p | 4.01E-01 | 1.00E+00 | 2.01E-01 | 0.534 (0.477; 0.591) | 0.045 |
| hsa-miR-3652 | 4.07E-01 | 1.00E+00 | 6.63E-01 | 0.513 (0.43; 0.544) | 0.038 |
| hsa-miR-3196 | 4.10E-01 | 1.00E+00 | 5.68E-01 | 0.518 (0.461; 0.575) | -0.026 |
| hsa-miR-4716-3p | 4.13E-01 | 1.00E+00 | 6.59E-01 | 0.513 (0.456; 0.57) | 0.031 |
| hsa-miR-22-5p | 4.15E-01 | 1.00E+00 | 2.26E-01 | 0.554 (0.497; 0.611) | -0.029 |
| hsa-miR-6126 | 4.18E-01 | 1.00E+00 | 4.95E-01 | 0.506 (0.438; 0.551) | 0.028 |
| hsa-miR-130b-3p | 4.20E-01 | 1.00E+00 | 8.33E-01 | 0.505 (0.438; 0.552) | 0.023 |
| hsa-miR-330-3p | 4.23E-01 | 1.00E+00 | 8.33E-01 | 0.512 (0.456; 0.569) | -0.026 |
| hsa-miR-6127 | 4.27E-01 | 1.00E+00 | 5.64E-01 | 0.528 (0.472; 0.585) | 0.028 |
| hsa-miR-4310 | 4.34E-01 | 1.00E+00 | 4.68E-01 | 0.541 (0.485; 0.598) | 0.016 |
| hsa-miR-328-3p | 4.37E-01 | 1.00E+00 | 4.15E-01 | 0.547 (0.491; 0.603) | -0.029 |
| hsa-miR-17-3p | 4.42E-01 | 1.00E+00 | 4.69E-01 | 0.522 (0.466; 0.579) | -0.037 |
| hsa-miR-30b-5p | 4.44E-01 | 1.00E+00 | 9.65E-01 | 0.502 (0.441; 0.555) | -0.023 |
| hsa-miR-7977 | 4.53E-01 | 1.00E+00 | 4.66E-01 | 0.501 (0.444; 0.558) | 0.050 |
| hsa-miR-1227-3p | 4.54E-01 | 1.00E+00 | 5.13E-01 | 0.518 (0.462; 0.575) | -0.009 |
| hsa-miR-197-5p | 4.66E-01 | 1.00E+00 | 5.35E-01 | 0.507 (0.45; 0.564) | 0.029 |
| hsa-miR-940 | 4.67E-01 | 1.00E+00 | 2.62E-01 | 0.512 (0.455; 0.569) | 0.020 |
| hsa-miR-3162-5p | 4.76E-01 | 1.00E+00 | 7.09E-01 | 0.501 (0.444; 0.558) | 0.025 |
| hsa-miR-6824-3p | 4.81E-01 | 1.00E+00 | 3.56E-01 | 0.526 (0.469; 0.582) | 0.013 |
| hsa-miR-454-5p | 4.81E-01 | 1.00E+00 | 5.66E-01 | 0.503 (0.446; 0.559) | -0.023 |
| hsa-miR-7107-5p | 4.83E-01 | 1.00E+00 | 3.12E-01 | 0.547 (0.49; 0.603) | -0.026 |
| hsa-miR-21-5p | 4.86E-01 | 1.00E+00 | 7.47E-01 | 0.516 (0.459; 0.573) | 0.029 |
| hsa-miR-6821-5p | 4.86E-01 | 1.00E+00 | 2.47E-01 | 0.507 (0.45; 0.564) | 0.018 |
| hsa-miR-362-3p | 4.89E-01 | 1.00E+00 | 8.26E-01 | 0.503 (0.44; 0.553) | -0.041 |
| hsa-miR-29b-3p | 4.90E-01 | 1.00E+00 | 1.17E-01 | 0.537 (0.481; 0.594) | -0.037 |
| hsa-miR-3180-3p | 4.94E-01 | 1.00E+00 | 3.96E-01 | 0.502 (0.442; 0.555) | -0.035 |
| hsa-miR-6513-3p | 4.94E-01 | 1.00E+00 | 5.03E-01 | 0.507 (0.45; 0.564) | -0.025 |
| hsa-miR-129-1-3p | 4.99E-01 | 1.00E+00 | 9.49E-01 | 0.515 (0.459; 0.572) | -0.014 |
| hsa-miR-133b | 4.99E-01 | 1.00E+00 | 6.05E-01 | 0.51 (0.453; 0.567) | -0.041 |
| hsa-miR-6087 | 5.17E-01 | 1.00E+00 | 3.90E-01 | 0.523 (0.466; 0.579) | 0.018 |
| hsa-miR-6165 | 5.18E-01 | 1.00E+00 | 7.86E-01 | 0.527 (0.47; 0.584) | 0.029 |
| hsa-miR-6508-5p | 5.22E-01 | 1.00E+00 | 4.52E-01 | 0.542 (0.486; 0.598) | 0.015 |
| hsa-miR-1273g-3p | 5.26E-01 | 1.00E+00 | 5.30E-01 | 0.53 (0.473; 0.586) | 0.028 |
| hsa-miR-584-5p | 5.27E-01 | 1.00E+00 | 7.59E-01 | 0.511 (0.455; 0.568) | 0.025 |
| hsa-miR-146b-5p | 5.31E-01 | 1.00E+00 | 2.12E-01 | 0.538 (0.481; 0.594) | 0.036 |
| hsa-miR-6803-5p | 5.32E-01 | 1.00E+00 | 8.61E-01 | 0.502 (0.441; 0.555) | -0.032 |
| hsa-miR-4788 | 5.44E-01 | 1.00E+00 | 6.14E-01 | 0.536 (0.48; 0.593) | 0.033 |
| hsa-miR-3940-3p | 5.45E-01 | 1.00E+00 | 4.91E-01 | 0.531 (0.475; 0.588) | -0.015 |
| hsa-miR-1304-3p | 5.47E-01 | 1.00E+00 | 4.89E-01 | 0.526 (0.469; 0.582) | 0.014 |
| hsa-miR-4787-5p | 5.52E-01 | 1.00E+00 | 5.77E-01 | 0.501 (0.444; 0.558) | -0.011 |
| hsa-miR-1237-3p | 5.54E-01 | 1.00E+00 | 5.29E-01 | 0.542 (0.486; 0.599) | 0.010 |
| hsa-miR-339-3p | 5.62E-01 | 1.00E+00 | 3.42E-01 | 0.501 (0.442; 0.556) | 0.020 |
| hsa-miR-5690 | 5.74E-01 | 1.00E+00 | 2.90E-01 | 0.525 (0.468; 0.582) | 0.032 |
| hsa-miR-182-5p | 5.80E-01 | 1.00E+00 | 5.03E-01 | 0.512 (0.431; 0.545) | -0.028 |
| hsa-miR-378d | 5.84E-01 | 1.00E+00 | 9.63E-01 | 0.504 (0.447; 0.561) | 0.017 |
| hsa-miR-4317 | 5.97E-01 | 1.00E+00 | 2.34E-01 | 0.523 (0.466; 0.58) | 0.015 |
| hsa-miR-939-5p | 6.04E-01 | 1.00E+00 | 7.11E-01 | 0.521 (0.465; 0.578) | -0.019 |
| hsa-miR-26b-5p | 6.07E-01 | 1.00E+00 | 8.15E-01 | 0.505 (0.447; 0.563) | 0.030 |
| hsa-miR-4324 | 6.09E-01 | 1.00E+00 | 3.61E-01 | 0.508 (0.451; 0.564) | 0.011 |
| hsa-miR-7-1-3p | 6.32E-01 | 1.00E+00 | 2.85E-01 | 0.556 (0.5; 0.612) | 0.027 |
| hsa-miR-6819-3p | 6.33E-01 | 1.00E+00 | 5.72E-01 | 0.516 (0.46; 0.573) | 0.009 |
| hsa-miR-625-3p | 6.34E-01 | 1.00E+00 | 2.34E-01 | 0.523 (0.466; 0.58) | 0.007 |
| hsa-miR-4713-3p | 6.42E-01 | 1.00E+00 | 9.38E-01 | 0.501 (0.444; 0.558) | 0.017 |
| hsa-miR-550a-3-5p | 6.44E-01 | 1.00E+00 | 2.84E-01 | 0.529 (0.472; 0.586) | 0.017 |
| hsa-miR-937-5p | 6.45E-01 | 1.00E+00 | 8.13E-01 | 0.514 (0.458; 0.571) | -0.015 |
| hsa-miR-26a-5p | 6.47E-01 | 1.00E+00 | 3.14E-01 | 0.512 (0.431; 0.545) | 0.017 |
| hsa-miR-8485 | 6.62E-01 | 1.00E+00 | 7.83E-01 | 0.56 (0.503; 0.616) | -0.025 |
| hsa-miR-27a-3p | 6.65E-01 | 1.00E+00 | 8.92E-01 | 0.518 (0.461; 0.575) | -0.020 |
| hsa-miR-1225-5p | 6.74E-01 | 1.00E+00 | 9.44E-01 | 0.509 (0.452; 0.565) | -0.009 |
| hsa-miR-502-3p | 6.77E-01 | 1.00E+00 | 6.00E-01 | 0.504 (0.447; 0.561) | 0.014 |
| hsa-miR-3960 | 6.80E-01 | 1.00E+00 | 9.34E-01 | 0.512 (0.431; 0.545) | -0.013 |
| hsa-miR-4763-3p | 6.89E-01 | 1.00E+00 | 4.81E-01 | 0.528 (0.471; 0.585) | 0.013 |
| hsa-miR-6124 | 6.91E-01 | 1.00E+00 | 9.58E-01 | 0.517 (0.426; 0.54) | 0.015 |
| hsa-miR-15b-5p | 6.98E-01 | 1.00E+00 | 7.65E-01 | 0.509 (0.435; 0.548) | -0.009 |
| hsa-miR-129-2-3p | 7.06E-01 | 1.00E+00 | 6.30E-01 | 0.507 (0.45; 0.563) | -0.010 |
| hsa-miR-6763-5p | 7.11E-01 | 1.00E+00 | 6.99E-01 | 0.523 (0.467; 0.58) | -0.012 |
| hsa-miR-4749-3p | 7.29E-01 | 1.00E+00 | 6.12E-01 | 0.526 (0.47; 0.583) | 0.006 |
| hsa-miR-4286 | 7.30E-01 | 1.00E+00 | 6.77E-01 | 0.504 (0.44; 0.553) | 0.019 |
| hsa-miR-1234-3p | 7.61E-01 | 1.00E+00 | 6.96E-01 | 0.506 (0.449; 0.562) | 0.007 |
| hsa-miR-6798-3p | 7.65E-01 | 1.00E+00 | 8.82E-01 | 0.503 (0.446; 0.559) | 0.003 |
| hsa-miR-624-5p | 7.68E-01 | 1.00E+00 | 9.76E-01 | 0.505 (0.448; 0.561) | -0.015 |
| hsa-miR-331-3p | 7.78E-01 | 1.00E+00 | 2.18E-01 | 0.55 (0.493; 0.606) | 0.013 |
| hsa-miR-223-3p | 7.80E-01 | 1.00E+00 | 5.08E-02 | 0.543 (0.487; 0.6) | 0.006 |
| hsa-miR-6826-5p | 7.80E-01 | 1.00E+00 | 5.38E-01 | 0.503 (0.446; 0.559) | 0.014 |
| hsa-miR-223-5p | 7.91E-01 | 1.00E+00 | 4.48E-01 | 0.516 (0.459; 0.572) | -0.007 |
| hsa-miR-4516 | 7.91E-01 | 1.00E+00 | 4.78E-01 | 0.506 (0.437; 0.55) | 0.010 |
| hsa-miR-5010-3p | 7.93E-01 | 1.00E+00 | 8.01E-01 | 0.516 (0.459; 0.572) | 0.005 |
| hsa-miR-505-3p | 7.98E-01 | 1.00E+00 | 3.47E-01 | 0.51 (0.453; 0.566) | 0.010 |
| hsa-miR-30e-5p | 7.99E-01 | 1.00E+00 | 7.01E-01 | 0.512 (0.455; 0.569) | -0.010 |
| hsa-miR-10a-5p | 8.01E-01 | 1.00E+00 | 6.26E-01 | 0.521 (0.422; 0.536) | -0.013 |
| hsa-miR-23b-3p | 8.06E-01 | 1.00E+00 | 1.46E-01 | 0.529 (0.472; 0.586) | 0.011 |
| hsa-miR-192-5p | 8.12E-01 | 1.00E+00 | 8.88E-01 | 0.523 (0.466; 0.579) | -0.010 |
| hsa-miR-378i | 8.28E-01 | 1.00E+00 | 9.25E-01 | 0.506 (0.45; 0.563) | 0.007 |
| hsa-miR-152-3p | 8.30E-01 | 1.00E+00 | 8.76E-01 | 0.506 (0.45; 0.563) | -0.006 |
| hsa-miR-27b-3p | 8.30E-01 | 1.00E+00 | 8.41E-01 | 0.506 (0.449; 0.563) | -0.012 |
| hsa-miR-4725-5p | 8.38E-01 | 1.00E+00 | 7.86E-01 | 0.505 (0.439; 0.552) | 0.005 |
| hsa-miR-6125 | 8.46E-01 | 1.00E+00 | 7.52E-01 | 0.519 (0.462; 0.575) | 0.007 |
| hsa-miR-425-3p | 8.48E-01 | 1.00E+00 | 9.63E-01 | 0.522 (0.465; 0.579) | -0.004 |
| hsa-miR-301a-3p | 8.49E-01 | 1.00E+00 | 3.16E-01 | 0.523 (0.466; 0.579) | -0.011 |
| hsa-miR-26b-3p | 8.50E-01 | 1.00E+00 | 3.99E-01 | 0.528 (0.471; 0.584) | 0.004 |
| hsa-miR-378a-3p | 8.56E-01 | 1.00E+00 | 4.89E-01 | 0.524 (0.467; 0.581) | -0.006 |
| hsa-miR-6085 | 8.61E-01 | 1.00E+00 | 8.19E-01 | 0.52 (0.463; 0.577) | 0.009 |
| hsa-miR-4442 | 8.65E-01 | 1.00E+00 | 7.56E-01 | 0.511 (0.454; 0.568) | 0.006 |
| hsa-miR-4466 | 8.80E-01 | 1.00E+00 | 4.80E-01 | 0.508 (0.451; 0.564) | 0.004 |
| hsa-miR-4507 | 8.80E-01 | 1.00E+00 | 5.86E-01 | 0.526 (0.47; 0.583) | 0.007 |
| hsa-miR-151a-5p | 8.84E-01 | 1.00E+00 | 6.96E-01 | 0.516 (0.459; 0.573) | 0.004 |
| hsa-miR-550a-3p | 8.94E-01 | 1.00E+00 | 1.37E-01 | 0.531 (0.474; 0.587) | 0.005 |
| hsa-miR-7976 | 8.94E-01 | 1.00E+00 | 5.14E-01 | 0.516 (0.46; 0.573) | 0.003 |
| hsa-miR-1908-3p | 8.94E-01 | 1.00E+00 | 9.00E-01 | 0.507 (0.436; 0.55) | 0.003 |
| hsa-miR-1587 | 8.95E-01 | 1.00E+00 | 9.20E-01 | 0.503 (0.446; 0.56) | -0.004 |
| hsa-miR-6740-5p | 8.98E-01 | 1.00E+00 | 5.64E-01 | 0.524 (0.468; 0.581) | -0.004 |
| hsa-miR-500a-3p | 8.99E-01 | 1.00E+00 | 9.22E-01 | 0.504 (0.447; 0.56) | 0.004 |
| hsa-miR-19b-3p | 9.08E-01 | 1.00E+00 | 3.79E-01 | 0.509 (0.434; 0.548) | 0.004 |
| hsa-miR-4433a-5p | 9.14E-01 | 1.00E+00 | 7.67E-01 | 0.523 (0.467; 0.58) | 0.002 |
| hsa-miR-423-3p | 9.16E-01 | 1.00E+00 | 4.27E-01 | 0.516 (0.459; 0.573) | 0.004 |
| hsa-miR-326 | 9.17E-01 | 1.00E+00 | 3.01E-01 | 0.531 (0.475; 0.588) | -0.004 |
| hsa-miR-320b | 9.31E-01 | 1.00E+00 | 8.28E-01 | 0.503 (0.44; 0.553) | 0.003 |
| hsa-miR-378g | 9.39E-01 | 1.00E+00 | 5.66E-01 | 0.511 (0.432; 0.546) | -0.003 |
| hsa-miR-128-3p | 9.44E-01 | 1.00E+00 | 3.46E-01 | 0.53 (0.474; 0.587) | -0.003 |
| hsa-miR-7847-3p | 9.59E-01 | 1.00E+00 | 7.32E-01 | 0.501 (0.442; 0.556) | -0.002 |
| hsa-miR-505-5p | 9.66E-01 | 1.00E+00 | 2.92E-01 | 0.515 (0.427; 0.542) | -0.001 |
| hsa-miR-125b-5p | 9.78E-01 | 1.00E+00 | 7.81E-01 | 0.51 (0.453; 0.566) | -0.002 |
| hsa-miR-16-2-3p | 9.88E-01 | 1.00E+00 | 8.64E-01 | 0.531 (0.475; 0.588) | 0.001 |
| hsa-miR-363-3p | 9.98E-01 | 1.00E+00 | 8.23E-01 | 0.527 (0.471; 0.584) | 0.000 |
| hsa-miR-130b-5p | 1.00E+00 | 1.00E+00 | 7.66E-01 | 0.502 (0.446; 0.559) | -0.000 |

**Table 8: Result metrics for the comparison of ICM versus control**

| miRNA | ttest_rawp | ttest_adjp | glm_rawp | AUC | log2FoldChange |
| --- | --- | --- | --- | --- | --- |
| hsa-miR-130a-3p | 2.30E-77 | 1.04E-74 | 6.91E-56 | 0.834 (0.805; 0.863) | 0.689 |
| hsa-let-7b-5p | 4.30E-73 | 1.94E-70 | 3.42E-51 | 0.816 (0.785; 0.847) | 0.883 |
| hsa-miR-6511b-3p | 2.39E-66 | 1.08E-63 | 3.05E-48 | 0.805 (0.775; 0.836) | -0.459 |
| hsa-miR-26b-3p | 1.36E-59 | 6.11E-57 | 1.19E-41 | 0.776 (0.743; 0.809) | -0.289 |
| hsa-miR-130b-5p | 7.33E-59 | 3.29E-56 | 1.40E-40 | 0.774 (0.74; 0.807) | -0.316 |
| hsa-miR-150-5p | 2.40E-57 | 1.08E-54 | 4.12E-39 | 0.772 (0.739; 0.805) | -0.715 |
| hsa-miR-4659a-3p | 5.03E-56 | 2.25E-53 | 1.69E-39 | 0.776 (0.743; 0.81) | -0.590 |
| hsa-miR-3653-3p | 3.12E-54 | 1.39E-51 | 1.90E-41 | 0.791 (0.759; 0.823) | -0.721 |
| hsa-miR-10a-5p | 3.31E-53 | 1.47E-50 | 2.64E-37 | 0.755 (0.721; 0.789) | -0.701 |
| hsa-let-7c-5p | 4.14E-53 | 1.84E-50 | 3.94E-41 | 0.772 (0.738; 0.806) | 0.741 |
| hsa-miR-139-3p | 1.01E-52 | 4.49E-50 | 4.09E-36 | 0.764 (0.73; 0.797) | -0.221 |
| hsa-miR-222-3p | 1.27E-50 | 5.63E-48 | 2.81E-41 | 0.799 (0.766; 0.831) | 0.520 |
| hsa-miR-484 | 1.15E-49 | 5.08E-47 | 1.07E-42 | 0.771 (0.738; 0.805) | -0.409 |
| hsa-miR-6513-3p | 3.38E-49 | 1.49E-46 | 2.91E-39 | 0.766 (0.732; 0.8) | -0.469 |
| hsa-miR-942-5p | 7.82E-48 | 3.43E-45 | 1.30E-40 | 0.78 (0.747; 0.813) | -0.593 |
| hsa-miR-30b-5p | 1.36E-45 | 5.96E-43 | 3.22E-38 | 0.766 (0.732; 0.8) | -0.454 |
| hsa-miR-107 | 9.86E-45 | 4.31E-42 | 4.15E-38 | 0.768 (0.734; 0.802) | 0.302 |
| hsa-miR-3651 | 1.46E-44 | 6.37E-42 | 1.22E-35 | 0.766 (0.732; 0.799) | -0.474 |
| hsa-miR-103a-3p | 3.57E-44 | 1.55E-41 | 7.42E-37 | 0.768 (0.734; 0.802) | 0.294 |
| hsa-miR-100-5p | 2.39E-43 | 1.04E-40 | 4.15E-36 | 0.739 (0.704; 0.773) | -2.064 |
| hsa-miR-4270 | 9.69E-43 | 4.20E-40 | 6.19E-31 | 0.772 (0.739; 0.806) | 0.323 |
| hsa-miR-139-5p | 1.28E-41 | 5.55E-39 | 1.23E-29 | 0.726 (0.69; 0.761) | -0.303 |
| hsa-miR-532-3p | 2.82E-41 | 1.21E-38 | 1.54E-34 | 0.738 (0.703; 0.773) | -0.450 |
| hsa-miR-181a-5p | 5.95E-41 | 2.56E-38 | 2.01E-33 | 0.751 (0.717; 0.786) | 0.629 |
| hsa-miR-584-5p | 1.66E-40 | 7.13E-38 | 1.90E-32 | 0.741 (0.706; 0.776) | 0.443 |
| hsa-miR-125b-5p | 6.29E-40 | 2.69E-37 | 1.01E-33 | 0.72 (0.685; 0.755) | -0.967 |
| hsa-miR-361-3p | 6.24E-39 | 2.66E-36 | 7.55E-34 | 0.734 (0.698; 0.769) | -0.375 |
| hsa-miR-199a-5p | 6.68E-39 | 2.85E-36 | 2.20E-27 | 0.713 (0.677; 0.749) | -0.641 |
| hsa-miR-17-3p | 7.33E-39 | 3.12E-36 | 2.83E-35 | 0.759 (0.724; 0.793) | 0.485 |
| hsa-miR-1275 | 1.29E-38 | 5.47E-36 | 1.05E-28 | 0.731 (0.695; 0.766) | -0.412 |
| hsa-miR-342-3p | 1.64E-38 | 6.92E-36 | 8.66E-30 | 0.711 (0.675; 0.748) | -0.417 |
| hsa-miR-4449 | 2.57E-38 | 1.09E-35 | 1.67E-28 | 0.723 (0.687; 0.759) | -0.294 |
| hsa-let-7i-5p | 1.21E-37 | 5.11E-35 | 1.63E-32 | 0.741 (0.706; 0.776) | 0.448 |
| hsa-miR-5690 | 1.47E-36 | 6.19E-34 | 6.52E-31 | 0.742 (0.706; 0.778) | -0.612 |
| hsa-miR-22-3p | 4.49E-35 | 1.88E-32 | 1.66E-27 | 0.727 (0.691; 0.762) | 0.307 |
| hsa-miR-4443 | 5.66E-35 | 2.36E-32 | 2.00E-25 | 0.705 (0.668; 0.741) | 0.461 |
| hsa-miR-221-3p | 1.70E-34 | 7.11E-32 | 3.42E-31 | 0.736 (0.7; 0.772) | 0.478 |
| hsa-miR-664a-3p | 6.77E-34 | 2.81E-31 | 5.89E-34 | 0.754 (0.719; 0.788) | -0.340 |
| hsa-miR-3200-5p | 1.21E-33 | 5.01E-31 | 1.29E-30 | 0.733 (0.698; 0.769) | -0.518 |
| hsa-miR-3605-3p | 1.96E-33 | 8.13E-31 | 9.94E-28 | 0.707 (0.67; 0.743) | -0.250 |
| hsa-miR-34a-5p | 2.80E-33 | 1.16E-30 | 2.67E-23 | 0.702 (0.665; 0.739) | 0.441 |
| hsa-miR-23a-3p | 7.46E-33 | 3.07E-30 | 2.62E-27 | 0.714 (0.677; 0.751) | -0.384 |
| hsa-miR-6794-5p | 7.72E-33 | 3.17E-30 | 6.10E-26 | 0.704 (0.667; 0.741) | 0.340 |
| hsa-miR-30c-5p | 1.13E-32 | 4.65E-30 | 1.21E-29 | 0.713 (0.676; 0.749) | -0.374 |
| hsa-miR-93-3p | 6.02E-32 | 2.46E-29 | 3.94E-27 | 0.711 (0.675; 0.748) | -0.351 |
| hsa-miR-148b-5p | 7.50E-32 | 3.06E-29 | 1.07E-27 | 0.726 (0.689; 0.762) | -0.304 |
| hsa-miR-15b-3p | 3.94E-31 | 1.60E-28 | 1.70E-25 | 0.713 (0.676; 0.75) | -0.285 |
| hsa-miR-145-5p | 4.65E-31 | 1.89E-28 | 1.92E-23 | 0.683 (0.645; 0.72) | -0.411 |
| hsa-miR-361-5p | 9.03E-31 | 3.66E-28 | 1.56E-24 | 0.685 (0.647; 0.722) | -0.325 |
| hsa-miR-6803-3p | 1.07E-30 | 4.33E-28 | 3.29E-26 | 0.707 (0.671; 0.744) | -0.204 |
| hsa-miR-454-5p | 1.30E-30 | 5.23E-28 | 4.23E-25 | 0.709 (0.672; 0.745) | -0.355 |
| hsa-miR-106b-5p | 2.16E-30 | 8.69E-28 | 2.85E-26 | 0.737 (0.701; 0.773) | 0.405 |
| hsa-miR-210-3p | 4.69E-30 | 1.88E-27 | 3.65E-27 | 0.754 (0.718; 0.789) | 0.485 |
| hsa-miR-425-5p | 6.87E-30 | 2.75E-27 | 7.97E-27 | 0.709 (0.672; 0.745) | -0.223 |
| hsa-miR-664b-3p | 4.14E-29 | 1.65E-26 | 1.73E-23 | 0.699 (0.662; 0.736) | -0.242 |
| hsa-miR-4306 | 8.19E-29 | 3.26E-26 | 1.52E-26 | 0.722 (0.686; 0.758) | 0.187 |
| hsa-miR-942-3p | 3.14E-28 | 1.24E-25 | 5.23E-25 | 0.691 (0.654; 0.728) | -0.307 |
| hsa-miR-93-5p | 4.57E-28 | 1.81E-25 | 6.80E-29 | 0.71 (0.673; 0.747) | 0.415 |
| hsa-miR-4732-3p | 6.12E-28 | 2.42E-25 | 4.99E-24 | 0.693 (0.656; 0.73) | -0.356 |
| hsa-miR-20b-5p | 1.21E-27 | 4.77E-25 | 4.43E-30 | 0.708 (0.672; 0.745) | 0.429 |
| hsa-miR-326 | 1.51E-27 | 5.92E-25 | 6.32E-20 | 0.677 (0.64; 0.715) | -0.372 |
| hsa-miR-7976 | 5.15E-27 | 2.02E-24 | 3.12E-21 | 0.679 (0.641; 0.717) | -0.175 |
| hsa-miR-18b-5p | 3.15E-26 | 1.23E-23 | 2.92E-24 | 0.684 (0.647; 0.721) | 0.474 |
| hsa-miR-495-3p | 3.88E-26 | 1.51E-23 | 3.00E-17 | 0.651 (0.612; 0.689) | -0.519 |
| hsa-miR-199b-5p | 1.10E-25 | 4.29E-23 | 1.08E-19 | 0.681 (0.643; 0.719) | -0.480 |
| hsa-miR-6800-5p | 2.62E-25 | 1.02E-22 | 1.51E-23 | 0.697 (0.661; 0.734) | 0.249 |
| hsa-miR-532-5p | 4.09E-25 | 1.58E-22 | 9.28E-22 | 0.727 (0.691; 0.764) | 0.270 |
| hsa-miR-320b | 5.07E-25 | 1.96E-22 | 1.53E-19 | 0.688 (0.651; 0.726) | 0.255 |
| hsa-miR-320d | 7.86E-25 | 3.03E-22 | 5.77E-19 | 0.686 (0.649; 0.723) | 0.244 |
| hsa-miR-142-5p | 1.51E-24 | 5.81E-22 | 2.35E-20 | 0.691 (0.654; 0.728) | -0.333 |
| hsa-miR-30e-3p | 1.76E-24 | 6.74E-22 | 1.16E-20 | 0.689 (0.651; 0.727) | -0.438 |
| hsa-miR-335-5p | 1.85E-24 | 7.06E-22 | 2.40E-19 | 0.67 (0.632; 0.708) | -0.378 |
| hsa-miR-642b-3p | 2.59E-24 | 9.88E-22 | 1.35E-18 | 0.689 (0.652; 0.726) | 0.257 |
| hsa-miR-101-3p | 3.26E-24 | 1.24E-21 | 2.86E-21 | 0.68 (0.642; 0.718) | 0.504 |
| hsa-miR-590-5p | 4.04E-24 | 1.53E-21 | 1.30E-19 | 0.677 (0.639; 0.715) | 0.538 |
| hsa-miR-4653-3p | 1.27E-23 | 4.81E-21 | 1.50E-22 | 0.729 (0.693; 0.764) | -0.247 |
| hsa-miR-140-5p | 1.45E-23 | 5.48E-21 | 2.35E-22 | 0.706 (0.669; 0.743) | 0.414 |
| hsa-miR-4284 | 2.30E-23 | 8.65E-21 | 1.53E-16 | 0.704 (0.667; 0.74) | -0.294 |
| hsa-miR-766-3p | 4.43E-23 | 1.66E-20 | 1.03E-16 | 0.671 (0.632; 0.709) | -0.324 |
| hsa-miR-6779-5p | 9.85E-23 | 3.69E-20 | 1.10E-19 | 0.697 (0.66; 0.734) | 0.174 |
| hsa-miR-320c | 1.14E-22 | 4.27E-20 | 5.85E-17 | 0.676 (0.638; 0.713) | 0.229 |
| hsa-miR-128-3p | 1.25E-22 | 4.64E-20 | 3.37E-20 | 0.679 (0.641; 0.717) | -0.352 |
| hsa-miR-654-3p | 1.34E-22 | 4.99E-20 | 6.02E-14 | 0.632 (0.593; 0.671) | -0.487 |
| hsa-miR-142-3p | 1.99E-22 | 7.37E-20 | 1.90E-19 | 0.687 (0.649; 0.724) | -0.455 |
| hsa-miR-4323 | 2.95E-22 | 1.09E-19 | 1.13E-15 | 0.665 (0.626; 0.703) | -0.328 |
| hsa-miR-185-5p | 7.25E-22 | 2.67E-19 | 1.99E-20 | 0.699 (0.662; 0.736) | 0.209 |
| hsa-miR-4687-3p | 1.09E-21 | 3.99E-19 | 3.10E-19 | 0.683 (0.645; 0.72) | 0.207 |
| hsa-miR-6088 | 1.13E-21 | 4.12E-19 | 1.43E-20 | 0.693 (0.655; 0.73) | 0.184 |
| hsa-let-7g-5p | 1.16E-21 | 4.22E-19 | 4.65E-22 | 0.679 (0.641; 0.717) | 0.288 |
| hsa-miR-4787-5p | 1.69E-21 | 6.13E-19 | 3.77E-19 | 0.678 (0.641; 0.716) | 0.150 |
| hsa-miR-183-5p | 2.85E-21 | 1.03E-18 | 1.13E-20 | 0.683 (0.645; 0.721) | 0.355 |
| hsa-miR-4317 | 7.25E-21 | 2.63E-18 | 8.52E-17 | 0.657 (0.619; 0.696) | -0.216 |
| hsa-miR-223-3p | 9.31E-21 | 3.36E-18 | 3.38E-14 | 0.653 (0.614; 0.691) | -0.189 |
| hsa-miR-4465 | 1.70E-20 | 6.13E-18 | 4.46E-19 | 0.689 (0.651; 0.726) | -0.374 |
| hsa-miR-3163 | 5.64E-20 | 2.02E-17 | 1.45E-17 | 0.655 (0.616; 0.693) | -0.240 |
| hsa-miR-18a-5p | 1.02E-19 | 3.65E-17 | 1.70E-19 | 0.664 (0.625; 0.702) | 0.438 |
| hsa-miR-7-5p | 1.80E-19 | 6.44E-17 | 2.59E-16 | 0.663 (0.625; 0.702) | 0.355 |
| hsa-miR-340-3p | 2.13E-19 | 7.58E-17 | 2.86E-15 | 0.662 (0.623; 0.7) | -0.516 |
| hsa-miR-4763-3p | 2.43E-19 | 8.62E-17 | 5.86E-17 | 0.675 (0.637; 0.713) | 0.208 |
| hsa-miR-125a-5p | 3.20E-19 | 1.13E-16 | 4.64E-17 | 0.64 (0.601; 0.679) | -0.404 |
| hsa-miR-4299 | 3.32E-19 | 1.17E-16 | 9.13E-16 | 0.676 (0.639; 0.714) | -0.270 |
| hsa-miR-99b-5p | 3.98E-19 | 1.40E-16 | 4.96E-14 | 0.634 (0.595; 0.674) | -0.359 |
| hsa-miR-181b-5p | 5.81E-19 | 2.04E-16 | 3.11E-15 | 0.669 (0.631; 0.707) | 0.267 |
| hsa-miR-126-5p | 7.76E-19 | 2.72E-16 | 3.27E-15 | 0.667 (0.629; 0.705) | -0.309 |
| hsa-miR-1587 | 8.71E-19 | 3.04E-16 | 1.67E-17 | 0.658 (0.62; 0.697) | 0.197 |
| hsa-miR-4466 | 1.14E-18 | 3.95E-16 | 6.55E-17 | 0.687 (0.65; 0.725) | 0.194 |
| hsa-miR-4436b-5p | 1.29E-18 | 4.48E-16 | 1.34E-15 | 0.698 (0.66; 0.735) | 0.265 |
| hsa-miR-330-3p | 1.66E-18 | 5.73E-16 | 7.17E-14 | 0.626 (0.587; 0.665) | -0.207 |
| hsa-miR-151a-3p | 2.18E-18 | 7.54E-16 | 1.58E-18 | 0.66 (0.622; 0.699) | -0.254 |
| hsa-miR-320e | 2.90E-18 | 9.98E-16 | 2.67E-14 | 0.658 (0.62; 0.697) | 0.206 |
| hsa-miR-362-3p | 5.55E-18 | 1.90E-15 | 5.12E-15 | 0.648 (0.609; 0.687) | -0.432 |
| hsa-miR-99a-5p | 8.53E-18 | 2.92E-15 | 1.96E-15 | 0.641 (0.603; 0.68) | -0.404 |
| hsa-miR-7975 | 1.13E-17 | 3.87E-15 | 2.68E-12 | 0.665 (0.626; 0.703) | 0.209 |
| hsa-miR-7847-3p | 1.33E-17 | 4.54E-15 | 4.80E-14 | 0.66 (0.622; 0.699) | 0.249 |
| hsa-let-7b-3p | 1.84E-17 | 6.24E-15 | 7.97E-17 | 0.688 (0.65; 0.726) | 0.132 |
| hsa-miR-642a-3p | 2.02E-17 | 6.81E-15 | 9.12E-17 | 0.69 (0.653; 0.728) | 0.286 |
| hsa-miR-29b-3p | 3.38E-17 | 1.14E-14 | 7.70E-11 | 0.64 (0.601; 0.679) | 0.337 |
| hsa-miR-30c-1-3p | 5.87E-17 | 1.97E-14 | 1.65E-15 | 0.665 (0.627; 0.703) | 0.064 |
| hsa-miR-4291 | 6.59E-17 | 2.21E-14 | 8.05E-14 | 0.653 (0.614; 0.692) | -0.237 |
| hsa-miR-16-5p | 2.03E-16 | 6.79E-14 | 1.53E-18 | 0.657 (0.618; 0.696) | 0.176 |
| hsa-miR-4505 | 2.15E-16 | 7.16E-14 | 3.36E-13 | 0.656 (0.618; 0.695) | 0.211 |
| hsa-miR-1225-5p | 2.20E-16 | 7.31E-14 | 8.38E-15 | 0.661 (0.623; 0.699) | 0.161 |
| hsa-miR-762 | 2.23E-16 | 7.39E-14 | 3.82E-13 | 0.66 (0.622; 0.698) | 0.208 |
| hsa-miR-1246 | 4.86E-16 | 1.60E-13 | 3.09E-12 | 0.648 (0.609; 0.688) | 0.373 |
| hsa-miR-17-5p | 7.26E-16 | 2.39E-13 | 3.13E-19 | 0.662 (0.623; 0.7) | 0.296 |
| hsa-miR-6869-5p | 1.78E-15 | 5.84E-13 | 9.88E-15 | 0.656 (0.617; 0.694) | 0.278 |
| hsa-miR-574-5p | 2.45E-15 | 8.00E-13 | 5.96E-11 | 0.62 (0.581; 0.66) | 0.319 |
| hsa-miR-181a-2-3p | 3.15E-15 | 1.03E-12 | 2.95E-11 | 0.624 (0.585; 0.663) | -0.152 |
| hsa-miR-20a-5p | 4.10E-15 | 1.33E-12 | 4.05E-19 | 0.646 (0.607; 0.685) | 0.351 |
| hsa-miR-937-5p | 4.28E-15 | 1.39E-12 | 2.84E-13 | 0.653 (0.615; 0.692) | 0.184 |
| hsa-miR-638 | 6.98E-15 | 2.26E-12 | 5.27E-14 | 0.65 (0.611; 0.689) | 0.186 |
| hsa-miR-29c-3p | 3.86E-14 | 1.24E-11 | 1.79E-11 | 0.658 (0.619; 0.697) | 0.287 |
| hsa-miR-92a-3p | 4.01E-14 | 1.29E-11 | 9.57E-15 | 0.639 (0.6; 0.678) | -0.131 |
| hsa-miR-409-3p | 6.35E-14 | 2.03E-11 | 4.00E-09 | 0.604 (0.564; 0.643) | -0.436 |
| hsa-miR-378i | 7.86E-14 | 2.51E-11 | 1.11E-10 | 0.64 (0.6; 0.679) | 0.199 |
| hsa-miR-7110-5p | 8.66E-14 | 2.76E-11 | 1.49E-11 | 0.629 (0.59; 0.668) | 0.243 |
| hsa-miR-500a-3p | 1.31E-13 | 4.16E-11 | 1.96E-11 | 0.655 (0.616; 0.694) | 0.185 |
| hsa-miR-365a-3p | 2.61E-13 | 8.26E-11 | 7.49E-12 | 0.619 (0.58; 0.659) | -0.255 |
| hsa-miR-4281 | 2.93E-13 | 9.23E-11 | 5.52E-11 | 0.63 (0.591; 0.669) | 0.179 |
| hsa-miR-1281 | 3.53E-13 | 1.11E-10 | 1.60E-12 | 0.654 (0.615; 0.693) | 0.154 |
| hsa-miR-1271-5p | 6.93E-13 | 2.17E-10 | 1.86E-08 | 0.607 (0.567; 0.647) | -0.257 |
| hsa-miR-629-3p | 1.11E-12 | 3.46E-10 | 1.17E-08 | 0.608 (0.568; 0.647) | -0.172 |
| hsa-miR-7-1-3p | 1.37E-12 | 4.27E-10 | 1.03E-09 | 0.62 (0.58; 0.66) | -0.332 |
| hsa-miR-502-3p | 1.59E-12 | 4.93E-10 | 1.42E-12 | 0.652 (0.613; 0.691) | 0.190 |
| hsa-miR-6089 | 1.73E-12 | 5.35E-10 | 1.15E-12 | 0.641 (0.602; 0.68) | 0.217 |
| hsa-let-7a-5p | 2.26E-12 | 6.96E-10 | 3.24E-14 | 0.638 (0.599; 0.677) | 0.168 |
| hsa-miR-215-5p | 2.32E-12 | 7.11E-10 | 1.62E-11 | 0.623 (0.583; 0.662) | -0.271 |
| hsa-miR-378d | 3.34E-12 | 1.02E-09 | 5.26E-09 | 0.616 (0.576; 0.656) | 0.187 |
| hsa-miR-15a-5p | 3.87E-12 | 1.18E-09 | 8.68E-13 | 0.634 (0.595; 0.674) | 0.223 |
| hsa-miR-320a | 4.42E-12 | 1.34E-09 | 1.53E-09 | 0.633 (0.594; 0.673) | 0.195 |
| hsa-miR-328-3p | 7.48E-12 | 2.27E-09 | 1.14E-09 | 0.619 (0.58; 0.659) | -0.187 |
| hsa-miR-4507 | 9.19E-12 | 2.78E-09 | 2.66E-11 | 0.633 (0.594; 0.672) | 0.238 |
| hsa-miR-4516 | 9.83E-12 | 2.96E-09 | 1.70E-10 | 0.626 (0.587; 0.665) | 0.204 |
| hsa-miR-6803-5p | 1.24E-11 | 3.73E-09 | 1.08E-10 | 0.632 (0.593; 0.671) | 0.265 |
| hsa-miR-564 | 1.49E-11 | 4.45E-09 | 1.52E-09 | 0.61 (0.57; 0.649) | -0.146 |
| hsa-miR-423-3p | 1.61E-11 | 4.79E-09 | 3.28E-10 | 0.629 (0.589; 0.669) | -0.217 |
| hsa-miR-1202 | 2.34E-11 | 6.94E-09 | 1.96E-09 | 0.624 (0.584; 0.663) | 0.193 |
| hsa-miR-502-5p | 2.39E-11 | 7.08E-09 | 2.18E-08 | 0.604 (0.564; 0.644) | -0.231 |
| hsa-miR-4530 | 2.74E-11 | 8.07E-09 | 9.73E-11 | 0.62 (0.581; 0.66) | 0.203 |
| hsa-miR-939-5p | 3.03E-11 | 8.90E-09 | 3.00E-09 | 0.627 (0.588; 0.666) | 0.183 |
| hsa-miR-6760-3p | 4.03E-11 | 1.18E-08 | 1.21E-11 | 0.64 (0.601; 0.679) | 0.085 |
| hsa-miR-6087 | 4.77E-11 | 1.39E-08 | 2.86E-10 | 0.615 (0.576; 0.655) | 0.158 |
| hsa-miR-6769b-5p | 4.79E-11 | 1.39E-08 | 2.80E-10 | 0.642 (0.603; 0.681) | 0.325 |
| hsa-miR-324-3p | 4.97E-11 | 1.44E-08 | 3.19E-11 | 0.622 (0.582; 0.661) | -0.124 |
| hsa-miR-1227-3p | 5.06E-11 | 1.46E-08 | 1.42E-08 | 0.629 (0.589; 0.668) | -0.057 |
| hsa-miR-30a-5p | 7.64E-11 | 2.20E-08 | 1.92E-10 | 0.653 (0.614; 0.692) | 0.221 |
| hsa-miR-296-5p | 8.57E-11 | 2.46E-08 | 4.99E-11 | 0.608 (0.568; 0.647) | -0.167 |
| hsa-miR-331-3p | 1.11E-10 | 3.17E-08 | 1.97E-08 | 0.602 (0.561; 0.642) | -0.250 |
| hsa-miR-3162-3p | 1.36E-10 | 3.88E-08 | 1.23E-10 | 0.629 (0.589; 0.669) | 0.151 |
| hsa-let-7f-5p | 1.75E-10 | 4.98E-08 | 2.06E-12 | 0.617 (0.577; 0.657) | 0.170 |
| hsa-miR-6126 | 2.36E-10 | 6.68E-08 | 1.01E-11 | 0.649 (0.611; 0.688) | -0.173 |
| hsa-miR-4433a-5p | 2.72E-10 | 7.67E-08 | 1.65E-10 | 0.644 (0.605; 0.683) | 0.088 |
| hsa-miR-146b-5p | 4.09E-10 | 1.15E-07 | 3.81E-06 | 0.601 (0.561; 0.641) | -0.275 |
| hsa-miR-15b-5p | 4.93E-10 | 1.38E-07 | 1.10E-13 | 0.673 (0.634; 0.711) | 0.114 |
| hsa-miR-191-5p | 6.93E-10 | 1.93E-07 | 4.45E-10 | 0.605 (0.565; 0.646) | -0.140 |
| hsa-miR-2861 | 7.05E-10 | 1.96E-07 | 2.05E-09 | 0.617 (0.577; 0.656) | 0.169 |
| hsa-miR-23b-3p | 7.29E-10 | 2.02E-07 | 2.64E-07 | 0.594 (0.554; 0.634) | -0.224 |
| hsa-miR-3940-3p | 8.85E-10 | 2.44E-07 | 8.70E-09 | 0.616 (0.576; 0.655) | -0.119 |
| hsa-miR-6090 | 9.84E-10 | 2.71E-07 | 2.59E-08 | 0.622 (0.583; 0.662) | 0.140 |
| hsa-miR-182-5p | 1.06E-09 | 2.90E-07 | 2.43E-09 | 0.61 (0.57; 0.65) | -0.250 |
| hsa-miR-1915-3p | 1.70E-09 | 4.63E-07 | 1.46E-08 | 0.616 (0.576; 0.655) | 0.155 |
| hsa-miR-4672 | 2.07E-09 | 5.64E-07 | 3.17E-08 | 0.604 (0.564; 0.644) | -0.160 |
| hsa-miR-550a-3-5p | 2.34E-09 | 6.33E-07 | 7.47E-14 | 0.655 (0.616; 0.694) | 0.162 |
| hsa-miR-28-5p | 2.75E-09 | 7.41E-07 | 2.34E-07 | 0.596 (0.556; 0.636) | -0.180 |
| hsa-miR-378g | 3.06E-09 | 8.24E-07 | 1.29E-06 | 0.603 (0.564; 0.643) | 0.185 |
| hsa-miR-4515 | 3.16E-09 | 8.48E-07 | 3.63E-11 | 0.67 (0.632; 0.708) | -0.127 |
| hsa-miR-8485 | 4.57E-09 | 1.22E-06 | 1.20E-07 | 0.601 (0.561; 0.641) | 0.282 |
| hsa-miR-6757-5p | 4.93E-09 | 1.31E-06 | 5.51E-07 | 0.6 (0.56; 0.64) | 0.118 |
| hsa-miR-5787 | 5.10E-09 | 1.35E-06 | 7.60E-08 | 0.657 (0.618; 0.696) | 0.311 |
| hsa-miR-6791-5p | 5.22E-09 | 1.38E-06 | 9.33E-08 | 0.596 (0.556; 0.635) | -0.146 |
| hsa-miR-30d-5p | 9.30E-09 | 2.45E-06 | 4.40E-08 | 0.623 (0.584; 0.663) | 0.129 |
| hsa-miR-27a-3p | 9.37E-09 | 2.45E-06 | 6.45E-06 | 0.593 (0.553; 0.633) | -0.209 |
| hsa-miR-4486 | 9.72E-09 | 2.54E-06 | 8.31E-08 | 0.621 (0.582; 0.661) | 0.161 |
| hsa-miR-378a-3p | 1.14E-08 | 2.97E-06 | 1.31E-05 | 0.592 (0.552; 0.632) | 0.149 |
| hsa-miR-7974 | 1.18E-08 | 3.07E-06 | 6.27E-08 | 0.623 (0.584; 0.663) | 0.075 |
| hsa-miR-130b-3p | 1.34E-08 | 3.46E-06 | 2.75E-07 | 0.612 (0.572; 0.652) | 0.138 |
| hsa-miR-1288-3p | 1.44E-08 | 3.69E-06 | 4.88E-12 | 0.638 (0.599; 0.677) | -0.137 |
| hsa-miR-485-3p | 1.60E-08 | 4.09E-06 | 1.85E-04 | 0.555 (0.514; 0.595) | -0.218 |
| hsa-miR-4716-3p | 1.90E-08 | 4.84E-06 | 1.39E-11 | 0.646 (0.607; 0.684) | -0.151 |
| hsa-miR-4746-3p | 1.93E-08 | 4.90E-06 | 1.91E-07 | 0.595 (0.555; 0.635) | -0.096 |
| hsa-miR-195-5p | 2.56E-08 | 6.47E-06 | 1.48E-06 | 0.598 (0.558; 0.638) | -0.198 |
| hsa-miR-6125 | 3.91E-08 | 9.86E-06 | 3.57E-08 | 0.6 (0.561; 0.64) | 0.155 |
| hsa-miR-6749-5p | 3.93E-08 | 9.87E-06 | 6.30E-06 | 0.6 (0.56; 0.641) | 0.227 |
| hsa-miR-374b-5p | 5.03E-08 | 1.26E-05 | 1.03E-06 | 0.587 (0.547; 0.628) | -0.229 |
| hsa-miR-6727-5p | 5.54E-08 | 1.38E-05 | 5.79E-07 | 0.617 (0.578; 0.657) | 0.174 |
| hsa-miR-940 | 5.94E-08 | 1.47E-05 | 6.22E-07 | 0.607 (0.568; 0.647) | -0.109 |
| hsa-miR-1825 | 6.63E-08 | 1.64E-05 | 5.07E-08 | 0.608 (0.568; 0.648) | 0.104 |
| hsa-miR-4732-5p | 6.97E-08 | 1.71E-05 | 1.82E-05 | 0.591 (0.551; 0.631) | 0.150 |
| hsa-miR-4793-5p | 7.92E-08 | 1.94E-05 | 2.30E-08 | 0.642 (0.603; 0.681) | -0.100 |
| hsa-miR-550a-3p | 8.01E-08 | 1.96E-05 | 4.63E-06 | 0.573 (0.532; 0.613) | -0.182 |
| hsa-miR-6865-3p | 8.03E-08 | 1.96E-05 | 6.78E-08 | 0.618 (0.578; 0.658) | 0.093 |
| hsa-miR-92b-3p | 9.05E-08 | 2.19E-05 | 2.94E-06 | 0.597 (0.557; 0.637) | -0.068 |
| hsa-miR-339-5p | 9.47E-08 | 2.28E-05 | 1.00E-07 | 0.595 (0.554; 0.635) | -0.197 |
| hsa-miR-5581-5p | 1.01E-07 | 2.42E-05 | 7.92E-11 | 0.639 (0.6; 0.678) | -0.140 |
| hsa-miR-3665 | 1.12E-07 | 2.68E-05 | 7.36E-08 | 0.627 (0.587; 0.666) | 0.133 |
| hsa-miR-4324 | 1.21E-07 | 2.87E-05 | 8.05E-07 | 0.618 (0.578; 0.657) | -0.090 |
| hsa-miR-21-5p | 1.98E-07 | 4.68E-05 | 2.19E-07 | 0.596 (0.556; 0.636) | 0.157 |
| hsa-miR-144-3p | 2.15E-07 | 5.07E-05 | 1.04E-07 | 0.592 (0.551; 0.632) | 0.376 |
| hsa-miR-1255b-5p | 3.09E-07 | 7.26E-05 | 6.23E-09 | 0.595 (0.555; 0.635) | -0.123 |
| hsa-miR-6724-5p | 4.78E-07 | 1.12E-04 | 1.90E-06 | 0.593 (0.553; 0.633) | 0.141 |
| hsa-miR-550a-5p | 5.73E-07 | 1.34E-04 | 2.41E-08 | 0.626 (0.586; 0.665) | 0.082 |
| hsa-miR-6798-3p | 6.29E-07 | 1.46E-04 | 2.25E-06 | 0.616 (0.577; 0.656) | -0.042 |
| hsa-miR-1207-5p | 9.24E-07 | 2.13E-04 | 1.61E-05 | 0.581 (0.541; 0.621) | 0.113 |
| hsa-miR-6767-5p | 9.31E-07 | 2.14E-04 | 1.96E-09 | 0.63 (0.59; 0.669) | -0.117 |
| hsa-miR-513a-5p | 1.00E-06 | 2.29E-04 | 1.18E-05 | 0.613 (0.573; 0.653) | 0.195 |
| hsa-miR-29a-3p | 1.06E-06 | 2.41E-04 | 6.04E-08 | 0.614 (0.575; 0.654) | 0.136 |
| hsa-miR-149-5p | 1.08E-06 | 2.45E-04 | 9.33E-07 | 0.604 (0.564; 0.644) | 0.068 |
| hsa-miR-6797-3p | 1.14E-06 | 2.58E-04 | 3.38E-07 | 0.602 (0.562; 0.642) | 0.082 |
| hsa-miR-133b | 1.23E-06 | 2.76E-04 | 7.48E-04 | 0.547 (0.507; 0.588) | -0.234 |
| hsa-miR-641 | 1.30E-06 | 2.90E-04 | 2.92E-05 | 0.585 (0.545; 0.625) | -0.099 |
| hsa-miR-22-5p | 1.38E-06 | 3.08E-04 | 2.20E-06 | 0.597 (0.556; 0.637) | -0.134 |
| hsa-miR-4649-3p | 1.51E-06 | 3.34E-04 | 5.05E-07 | 0.598 (0.558; 0.639) | 0.096 |
| hsa-miR-192-5p | 1.89E-06 | 4.17E-04 | 4.73E-06 | 0.58 (0.539; 0.62) | -0.178 |
| hsa-miR-338-3p | 1.98E-06 | 4.35E-04 | 4.39E-05 | 0.581 (0.54; 0.621) | -0.166 |
| hsa-miR-3195 | 2.36E-06 | 5.16E-04 | 9.09E-04 | 0.596 (0.555; 0.636) | 0.151 |
| hsa-miR-628-5p | 2.68E-06 | 5.84E-04 | 3.05E-05 | 0.578 (0.538; 0.619) | -0.111 |
| hsa-miR-892b | 2.78E-06 | 6.03E-04 | 1.04E-09 | 0.609 (0.569; 0.648) | -0.094 |
| hsa-miR-3180-3p | 2.86E-06 | 6.18E-04 | 7.74E-05 | 0.586 (0.546; 0.627) | 0.187 |
| hsa-miR-1539 | 3.05E-06 | 6.56E-04 | 1.29E-05 | 0.599 (0.559; 0.639) | 0.070 |
| hsa-let-7f-1-3p | 3.26E-06 | 6.99E-04 | 2.21E-06 | 0.614 (0.574; 0.654) | 0.065 |
| hsa-miR-5739 | 3.32E-06 | 7.07E-04 | 8.34E-04 | 0.573 (0.532; 0.613) | 0.229 |
| hsa-miR-197-3p | 3.62E-06 | 7.67E-04 | 3.01E-05 | 0.58 (0.54; 0.62) | -0.127 |
| hsa-miR-513b-5p | 4.48E-06 | 9.45E-04 | 5.12E-06 | 0.582 (0.541; 0.622) | 0.106 |
| hsa-miR-7114-5p | 5.22E-06 | 1.10E-03 | 7.38E-04 | 0.577 (0.537; 0.617) | 0.187 |
| hsa-miR-6068 | 6.33E-06 | 1.32E-03 | 7.03E-06 | 0.606 (0.566; 0.646) | 0.110 |
| hsa-miR-378a-5p | 6.65E-06 | 1.38E-03 | 8.29E-05 | 0.569 (0.528; 0.61) | -0.166 |
| hsa-miR-5088-5p | 6.67E-06 | 1.38E-03 | 3.65E-09 | 0.61 (0.57; 0.649) | -0.115 |
| hsa-miR-301a-3p | 7.31E-06 | 1.51E-03 | 3.75E-06 | 0.581 (0.54; 0.621) | 0.212 |
| hsa-let-7e-5p | 9.04E-06 | 1.85E-03 | 4.56E-05 | 0.568 (0.527; 0.608) | -0.133 |
| hsa-miR-181c-5p | 9.08E-06 | 1.85E-03 | 1.57E-05 | 0.594 (0.554; 0.634) | 0.144 |
| hsa-miR-362-5p | 1.01E-05 | 2.05E-03 | 8.01E-05 | 0.596 (0.556; 0.636) | 0.124 |
| hsa-miR-4745-5p | 1.07E-05 | 2.16E-03 | 5.76E-06 | 0.566 (0.525; 0.606) | -0.055 |
| hsa-miR-339-3p | 1.22E-05 | 2.44E-03 | 2.39E-06 | 0.6 (0.56; 0.64) | 0.109 |
| hsa-miR-660-5p | 1.28E-05 | 2.56E-03 | 3.75E-06 | 0.605 (0.564; 0.645) | 0.131 |
| hsa-miR-629-5p | 1.29E-05 | 2.57E-03 | 7.70E-06 | 0.609 (0.569; 0.649) | 0.121 |
| hsa-miR-26b-5p | 1.32E-05 | 2.61E-03 | 8.18E-05 | 0.588 (0.547; 0.628) | -0.198 |
| hsa-miR-96-5p | 1.47E-05 | 2.89E-03 | 9.66E-07 | 0.581 (0.54; 0.621) | 0.183 |
| hsa-miR-4664-3p | 1.68E-05 | 3.30E-03 | 1.95E-05 | 0.587 (0.547; 0.627) | 0.063 |
| hsa-miR-424-5p | 1.94E-05 | 3.77E-03 | 1.68E-04 | 0.576 (0.536; 0.617) | 0.200 |
| hsa-miR-6893-5p | 2.28E-05 | 4.43E-03 | 2.53E-06 | 0.603 (0.563; 0.643) | -0.119 |
| hsa-miR-501-3p | 2.73E-05 | 5.27E-03 | 2.31E-05 | 0.611 (0.571; 0.651) | 0.094 |
| hsa-miR-3200-3p | 2.79E-05 | 5.36E-03 | 2.19E-03 | 0.558 (0.517; 0.598) | -0.133 |
| hsa-miR-7107-5p | 2.98E-05 | 5.70E-03 | 3.85E-04 | 0.555 (0.515; 0.596) | 0.142 |
| hsa-miR-6085 | 3.33E-05 | 6.33E-03 | 5.02E-03 | 0.563 (0.522; 0.603) | 0.158 |
| hsa-miR-550b-2-5p | 4.76E-05 | 9.00E-03 | 4.60E-08 | 0.601 (0.561; 0.641) | 0.086 |
| hsa-miR-4310 | 4.90E-05 | 9.21E-03 | 7.25E-05 | 0.589 (0.548; 0.629) | 0.066 |
| hsa-miR-374a-5p | 6.24E-05 | 1.17E-02 | 2.00E-03 | 0.563 (0.523; 0.604) | -0.225 |
| hsa-miR-3613-3p | 6.74E-05 | 1.25E-02 | 1.50E-05 | 0.586 (0.546; 0.626) | 0.048 |
| hsa-miR-7152-3p | 6.89E-05 | 1.28E-02 | 4.84E-03 | 0.565 (0.524; 0.605) | 0.115 |
| hsa-miR-625-5p | 8.54E-05 | 1.57E-02 | 7.22E-07 | 0.596 (0.556; 0.636) | 0.147 |
| hsa-miR-6731-3p | 9.01E-05 | 1.65E-02 | 4.36E-05 | 0.578 (0.537; 0.618) | 0.051 |
| hsa-miR-24-3p | 1.00E-04 | 1.83E-02 | 3.47E-04 | 0.561 (0.52; 0.602) | -0.073 |
| hsa-miR-625-3p | 1.11E-04 | 2.02E-02 | 2.46E-06 | 0.595 (0.555; 0.635) | 0.048 |
| hsa-miR-3679-5p | 1.22E-04 | 2.20E-02 | 1.04E-03 | 0.553 (0.513; 0.594) | 0.110 |
| hsa-miR-5194 | 1.40E-04 | 2.50E-02 | 2.04E-07 | 0.6 (0.56; 0.64) | -0.089 |
| hsa-miR-451a | 1.61E-04 | 2.86E-02 | 5.02E-05 | 0.598 (0.558; 0.639) | 0.022 |
| hsa-miR-6813-3p | 1.68E-04 | 2.97E-02 | 7.87E-05 | 0.574 (0.533; 0.614) | 0.056 |
| hsa-let-7d-3p | 1.79E-04 | 3.16E-02 | 2.47E-03 | 0.571 (0.53; 0.611) | -0.066 |
| hsa-miR-4769-3p | 1.80E-04 | 3.16E-02 | 8.92E-05 | 0.575 (0.535; 0.615) | 0.053 |
| hsa-miR-6717-5p | 2.17E-04 | 3.78E-02 | 1.17E-06 | 0.603 (0.563; 0.643) | -0.092 |
| hsa-miR-598-3p | 2.21E-04 | 3.83E-02 | 5.01E-05 | 0.568 (0.528; 0.609) | 0.094 |
| hsa-miR-4442 | 2.23E-04 | 3.83E-02 | 1.64E-02 | 0.547 (0.506; 0.587) | 0.102 |
| hsa-miR-769-5p | 2.35E-04 | 4.02E-02 | 3.89E-04 | 0.588 (0.548; 0.628) | 0.090 |
| hsa-miR-19b-3p | 2.77E-04 | 4.71E-02 | 3.27E-03 | 0.563 (0.522; 0.604) | -0.104 |
| hsa-miR-146a-5p | 2.82E-04 | 4.76E-02 | 3.71E-03 | 0.55 (0.509; 0.591) | -0.100 |
| hsa-miR-4725-5p | 3.20E-04 | 5.38E-02 | 1.87E-04 | 0.565 (0.524; 0.605) | 0.067 |
| hsa-miR-6785-5p | 3.70E-04 | 6.18E-02 | 2.10E-03 | 0.594 (0.554; 0.635) | 0.212 |
| hsa-miR-374c-5p | 4.38E-04 | 7.27E-02 | 2.59E-03 | 0.541 (0.5; 0.581) | -0.138 |
| hsa-miR-6777-3p | 4.41E-04 | 7.27E-02 | 2.25E-04 | 0.573 (0.533; 0.613) | 0.046 |
| hsa-miR-29c-5p | 4.55E-04 | 7.46E-02 | 8.23E-05 | 0.563 (0.523; 0.604) | -0.100 |
| hsa-miR-342-5p | 5.52E-04 | 9.00E-02 | 1.38E-03 | 0.544 (0.503; 0.585) | -0.094 |
| hsa-miR-1973 | 6.43E-04 | 1.04E-01 | 2.22E-05 | 0.575 (0.534; 0.615) | -0.117 |
| hsa-miR-1268b | 6.82E-04 | 1.10E-01 | 3.01E-03 | 0.559 (0.518; 0.599) | 0.095 |
| hsa-miR-6734-5p | 6.88E-04 | 1.10E-01 | 6.84E-07 | 0.616 (0.576; 0.655) | -0.093 |
| hsa-miR-4513 | 7.98E-04 | 1.27E-01 | 1.49E-02 | 0.555 (0.514; 0.595) | 0.117 |
| hsa-miR-193a-5p | 8.92E-04 | 1.41E-01 | 4.17E-03 | 0.582 (0.541; 0.622) | 0.065 |
| hsa-miR-505-3p | 1.07E-03 | 1.69E-01 | 4.24E-03 | 0.546 (0.505; 0.587) | -0.105 |
| hsa-miR-6858-3p | 1.08E-03 | 1.69E-01 | 5.08E-04 | 0.558 (0.518; 0.599) | 0.053 |
| hsa-miR-7704 | 1.15E-03 | 1.78E-01 | 1.67E-03 | 0.57 (0.53; 0.611) | 0.083 |
| hsa-miR-6821-5p | 1.17E-03 | 1.80E-01 | 4.27E-03 | 0.559 (0.519; 0.6) | 0.060 |
| hsa-miR-4758-3p | 1.18E-03 | 1.80E-01 | 4.47E-04 | 0.563 (0.522; 0.603) | 0.048 |
| hsa-miR-6165 | 1.20E-03 | 1.83E-01 | 5.54E-02 | 0.546 (0.506; 0.587) | 0.112 |
| hsa-miR-4428 | 1.30E-03 | 1.96E-01 | 4.91E-03 | 0.549 (0.508; 0.59) | 0.126 |
| hsa-miR-19a-3p | 1.45E-03 | 2.17E-01 | 1.71E-04 | 0.575 (0.535; 0.616) | 0.115 |
| hsa-miR-4318 | 1.51E-03 | 2.26E-01 | 2.80E-03 | 0.558 (0.517; 0.598) | -0.076 |
| hsa-miR-505-5p | 1.91E-03 | 2.82E-01 | 7.40E-05 | 0.556 (0.515; 0.596) | -0.086 |
| hsa-miR-33b-3p | 2.04E-03 | 3.00E-01 | 1.50E-03 | 0.562 (0.522; 0.603) | 0.044 |
| hsa-miR-144-5p | 2.53E-03 | 3.69E-01 | 7.60E-04 | 0.575 (0.534; 0.615) | -0.173 |
| hsa-miR-194-5p | 2.71E-03 | 3.93E-01 | 3.58E-03 | 0.572 (0.531; 0.612) | 0.106 |
| hsa-miR-6132 | 2.71E-03 | 3.93E-01 | 1.24E-02 | 0.556 (0.515; 0.597) | 0.125 |
| hsa-miR-324-5p | 2.77E-03 | 3.96E-01 | 6.21E-04 | 0.583 (0.543; 0.624) | 0.069 |
| hsa-miR-6508-5p | 2.90E-03 | 4.12E-01 | 1.37E-03 | 0.58 (0.539; 0.62) | 0.055 |
| hsa-miR-152-3p | 2.90E-03 | 4.12E-01 | 1.81E-03 | 0.566 (0.526; 0.607) | 0.063 |
| hsa-miR-3960 | 2.91E-03 | 4.12E-01 | 1.99E-03 | 0.56 (0.52; 0.601) | 0.078 |
| hsa-miR-27b-3p | 2.99E-03 | 4.15E-01 | 1.34E-01 | 0.538 (0.497; 0.579) | -0.121 |
| hsa-miR-8063 | 3.08E-03 | 4.25E-01 | 1.63E-02 | 0.555 (0.514; 0.595) | 0.093 |
| hsa-miR-4286 | 3.39E-03 | 4.65E-01 | 1.52E-02 | 0.542 (0.501; 0.583) | 0.128 |
| hsa-miR-1237-3p | 3.71E-03 | 5.05E-01 | 3.49E-03 | 0.572 (0.531; 0.612) | 0.039 |
| hsa-miR-6131 | 4.03E-03 | 5.45E-01 | 2.83E-05 | 0.59 (0.55; 0.63) | -0.074 |
| hsa-miR-1228-3p | 4.06E-03 | 5.45E-01 | 3.49E-03 | 0.565 (0.524; 0.605) | 0.046 |
| hsa-miR-196b-5p | 4.14E-03 | 5.51E-01 | 2.80E-05 | 0.57 (0.53; 0.611) | 0.106 |
| hsa-miR-3125 | 4.16E-03 | 5.51E-01 | 1.02E-05 | 0.583 (0.543; 0.623) | -0.077 |
| hsa-miR-6737-3p | 4.19E-03 | 5.51E-01 | 1.52E-03 | 0.568 (0.527; 0.609) | 0.054 |
| hsa-miR-6127 | 4.27E-03 | 5.56E-01 | 2.32E-05 | 0.576 (0.536; 0.617) | -0.081 |
| hsa-miR-4485-3p | 4.40E-03 | 5.68E-01 | 8.28E-04 | 0.557 (0.517; 0.598) | -0.084 |
| hsa-miR-6763-5p | 4.53E-03 | 5.79E-01 | 3.59E-02 | 0.552 (0.511; 0.592) | 0.070 |
| hsa-miR-4728-5p | 4.65E-03 | 5.91E-01 | 1.73E-02 | 0.55 (0.51; 0.591) | 0.098 |
| hsa-miR-6780a-5p | 4.76E-03 | 6.00E-01 | 9.44E-06 | 0.578 (0.538; 0.618) | -0.073 |
| hsa-miR-491-5p | 4.77E-03 | 6.00E-01 | 6.56E-03 | 0.54 (0.499; 0.581) | -0.018 |
| hsa-miR-26a-5p | 4.81E-03 | 6.00E-01 | 1.63E-02 | 0.55 (0.509; 0.591) | -0.081 |
| hsa-miR-624-5p | 5.36E-03 | 6.59E-01 | 4.99E-02 | 0.54 (0.499; 0.58) | -0.115 |
| hsa-miR-627-5p | 5.36E-03 | 6.59E-01 | 2.88E-03 | 0.542 (0.501; 0.582) | -0.094 |
| hsa-miR-6807-5p | 5.63E-03 | 6.81E-01 | 2.91E-05 | 0.575 (0.535; 0.615) | -0.058 |
| hsa-miR-4713-3p | 6.01E-03 | 7.21E-01 | 3.57E-05 | 0.595 (0.555; 0.635) | -0.074 |
| hsa-miR-371b-5p | 6.76E-03 | 8.05E-01 | 6.50E-03 | 0.575 (0.535; 0.615) | 0.068 |
| hsa-miR-4739 | 6.87E-03 | 8.10E-01 | 6.44E-03 | 0.535 (0.495; 0.576) | -0.055 |
| hsa-miR-1305 | 7.80E-03 | 9.12E-01 | 8.32E-05 | 0.591 (0.551; 0.631) | -0.063 |
| hsa-miR-3656 | 8.20E-03 | 9.52E-01 | 2.63E-03 | 0.567 (0.527; 0.608) | 0.096 |
| hsa-miR-4459 | 8.69E-03 | 9.99E-01 | 2.71E-02 | 0.529 (0.488; 0.569) | 0.093 |
| hsa-miR-1234-3p | 9.77E-03 | 1.00E+00 | 7.03E-03 | 0.546 (0.505; 0.586) | 0.044 |
| hsa-miR-5001-5p | 9.83E-03 | 1.00E+00 | 1.90E-02 | 0.562 (0.521; 0.602) | 0.056 |
| hsa-miR-6069 | 1.04E-02 | 1.00E+00 | 5.81E-03 | 0.557 (0.516; 0.598) | 0.043 |
| hsa-miR-494-3p | 1.13E-02 | 1.00E+00 | 6.34E-02 | 0.543 (0.502; 0.584) | 0.144 |
| hsa-miR-25-3p | 1.17E-02 | 1.00E+00 | 3.55E-03 | 0.559 (0.518; 0.6) | -0.030 |
| hsa-miR-3162-5p | 1.34E-02 | 1.00E+00 | 2.14E-01 | 0.53 (0.49; 0.571) | 0.062 |
| hsa-miR-6826-5p | 1.37E-02 | 1.00E+00 | 5.33E-02 | 0.54 (0.499; 0.58) | 0.089 |
| hsa-miR-6763-3p | 1.55E-02 | 1.00E+00 | 1.08E-02 | 0.548 (0.507; 0.588) | 0.032 |
| hsa-miR-5010-3p | 1.73E-02 | 1.00E+00 | 4.88E-02 | 0.552 (0.512; 0.593) | -0.037 |
| hsa-miR-30e-5p | 1.80E-02 | 1.00E+00 | 3.88E-02 | 0.551 (0.51; 0.591) | 0.064 |
| hsa-miR-197-5p | 2.07E-02 | 1.00E+00 | 3.23E-01 | 0.524 (0.484; 0.565) | 0.057 |
| hsa-miR-200c-3p | 2.25E-02 | 1.00E+00 | 2.12E-02 | 0.564 (0.524; 0.605) | 0.047 |
| hsa-miR-155-5p | 2.25E-02 | 1.00E+00 | 6.08E-03 | 0.566 (0.525; 0.606) | 0.064 |
| hsa-miR-6800-3p | 2.39E-02 | 1.00E+00 | 1.40E-02 | 0.562 (0.521; 0.602) | 0.041 |
| hsa-miR-486-5p | 2.41E-02 | 1.00E+00 | 2.95E-02 | 0.553 (0.513; 0.594) | 0.025 |
| hsa-miR-874-3p | 2.64E-02 | 1.00E+00 | 2.81E-02 | 0.532 (0.491; 0.573) | -0.038 |
| hsa-miR-4485-5p | 2.94E-02 | 1.00E+00 | 1.00E-02 | 0.55 (0.509; 0.591) | -0.085 |
| hsa-miR-1255a | 2.95E-02 | 1.00E+00 | 1.28E-01 | 0.515 (0.474; 0.556) | 0.037 |
| hsa-miR-18a-3p | 3.04E-02 | 1.00E+00 | 1.21E-02 | 0.537 (0.496; 0.578) | -0.044 |
| hsa-miR-7108-5p | 3.16E-02 | 1.00E+00 | 2.81E-02 | 0.543 (0.502; 0.583) | -0.058 |
| hsa-miR-7150 | 3.40E-02 | 1.00E+00 | 1.02E-01 | 0.536 (0.495; 0.577) | 0.064 |
| hsa-let-7d-5p | 3.43E-02 | 1.00E+00 | 8.31E-03 | 0.549 (0.508; 0.59) | 0.049 |
| hsa-miR-1238-3p | 3.59E-02 | 1.00E+00 | 2.29E-02 | 0.553 (0.512; 0.593) | 0.038 |
| hsa-miR-223-5p | 3.75E-02 | 1.00E+00 | 1.15E-01 | 0.533 (0.492; 0.574) | 0.044 |
| hsa-miR-6891-5p | 4.03E-02 | 1.00E+00 | 2.17E-01 | 0.542 (0.501; 0.583) | 0.045 |
| hsa-miR-501-5p | 4.06E-02 | 1.00E+00 | 5.11E-02 | 0.553 (0.512; 0.593) | 0.055 |
| hsa-miR-2116-3p | 4.77E-02 | 1.00E+00 | 2.94E-02 | 0.546 (0.505; 0.587) | 0.031 |
| hsa-miR-6512-5p | 5.26E-02 | 1.00E+00 | 9.34E-04 | 0.551 (0.511; 0.592) | -0.049 |
| hsa-miR-3196 | 5.64E-02 | 1.00E+00 | 1.06E-01 | 0.551 (0.51; 0.591) | 0.046 |
| hsa-miR-1260b | 5.68E-02 | 1.00E+00 | 2.53E-02 | 0.556 (0.515; 0.596) | -0.061 |
| hsa-miR-3198 | 5.94E-02 | 1.00E+00 | 1.12E-03 | 0.568 (0.527; 0.608) | -0.051 |
| hsa-miR-4741 | 7.07E-02 | 1.00E+00 | 2.50E-01 | 0.527 (0.486; 0.568) | 0.073 |
| hsa-miR-1268a | 7.74E-02 | 1.00E+00 | 7.09E-02 | 0.554 (0.514; 0.595) | -0.049 |
| hsa-miR-191-3p | 7.77E-02 | 1.00E+00 | 6.41E-02 | 0.54 (0.499; 0.58) | 0.031 |
| hsa-miR-3652 | 7.77E-02 | 1.00E+00 | 4.53E-01 | 0.518 (0.478; 0.559) | 0.057 |
| hsa-miR-148a-3p | 8.73E-02 | 1.00E+00 | 5.28E-02 | 0.526 (0.485; 0.567) | -0.068 |
| hsa-miR-1285-3p | 8.79E-02 | 1.00E+00 | 2.64E-01 | 0.518 (0.477; 0.559) | -0.033 |
| hsa-miR-4484 | 9.86E-02 | 1.00E+00 | 1.78E-01 | 0.544 (0.503; 0.585) | 0.019 |
| hsa-miR-575 | 9.96E-02 | 1.00E+00 | 4.01E-03 | 0.552 (0.511; 0.592) | -0.042 |
| hsa-miR-140-3p | 1.09E-01 | 1.00E+00 | 9.42E-01 | 0.516 (0.475; 0.557) | -0.036 |
| hsa-miR-500b-5p | 1.11E-01 | 1.00E+00 | 1.90E-01 | 0.506 (0.466; 0.547) | -0.039 |
| hsa-miR-6073 | 1.14E-01 | 1.00E+00 | 2.15E-03 | 0.557 (0.517; 0.598) | -0.049 |
| hsa-miR-4532 | 1.18E-01 | 1.00E+00 | 6.11E-02 | 0.508 (0.467; 0.549) | -0.021 |
| hsa-miR-4788 | 1.18E-01 | 1.00E+00 | 4.18E-01 | 0.525 (0.484; 0.566) | 0.064 |
| hsa-miR-103a-2-5p | 1.22E-01 | 1.00E+00 | 9.29E-03 | 0.515 (0.475; 0.556) | -0.036 |
| hsa-miR-423-5p | 1.27E-01 | 1.00E+00 | 4.90E-03 | 0.532 (0.492; 0.573) | -0.037 |
| hsa-miR-6875-5p | 1.45E-01 | 1.00E+00 | 8.78E-01 | 0.513 (0.472; 0.554) | 0.043 |
| hsa-miR-6785-3p | 1.67E-01 | 1.00E+00 | 1.08E-01 | 0.518 (0.477; 0.559) | 0.018 |
| hsa-miR-4685-5p | 1.67E-01 | 1.00E+00 | 5.28E-03 | 0.55 (0.51; 0.591) | -0.070 |
| hsa-miR-186-5p | 1.72E-01 | 1.00E+00 | 1.67E-01 | 0.529 (0.488; 0.57) | 0.030 |
| hsa-miR-7641 | 1.73E-01 | 1.00E+00 | 4.44E-03 | 0.553 (0.512; 0.594) | -0.056 |
| hsa-miR-6889-3p | 1.76E-01 | 1.00E+00 | 1.15E-01 | 0.536 (0.495; 0.577) | 0.023 |
| hsa-miR-151b | 1.81E-01 | 1.00E+00 | 4.27E-01 | 0.542 (0.501; 0.583) | 0.028 |
| hsa-miR-16-2-3p | 1.84E-01 | 1.00E+00 | 8.43E-01 | 0.505 (0.464; 0.545) | 0.039 |
| hsa-miR-6812-3p | 1.91E-01 | 1.00E+00 | 2.75E-01 | 0.534 (0.493; 0.574) | -0.017 |
| hsa-miR-4749-3p | 1.92E-01 | 1.00E+00 | 2.97E-01 | 0.526 (0.485; 0.567) | -0.017 |
| hsa-miR-1908-3p | 2.02E-01 | 1.00E+00 | 2.96E-01 | 0.547 (0.506; 0.587) | -0.020 |
| hsa-miR-4497 | 2.07E-01 | 1.00E+00 | 1.62E-01 | 0.521 (0.48; 0.562) | -0.028 |
| hsa-miR-6515-3p | 2.23E-01 | 1.00E+00 | 1.82E-01 | 0.54 (0.499; 0.58) | 0.017 |
| hsa-miR-6789-5p | 2.48E-01 | 1.00E+00 | 1.57E-01 | 0.545 (0.505; 0.586) | 0.042 |
| hsa-miR-6880-3p | 2.53E-01 | 1.00E+00 | 3.43E-01 | 0.531 (0.49; 0.572) | -0.015 |
| hsa-miR-744-5p | 2.57E-01 | 1.00E+00 | 1.22E-01 | 0.555 (0.514; 0.596) | 0.031 |
| hsa-miR-619-5p | 2.72E-01 | 1.00E+00 | 2.08E-01 | 0.569 (0.529; 0.61) | -0.053 |
| hsa-miR-4313 | 2.81E-01 | 1.00E+00 | 1.75E-01 | 0.531 (0.491; 0.572) | 0.020 |
| hsa-miR-6819-3p | 2.85E-01 | 1.00E+00 | 4.29E-01 | 0.524 (0.483; 0.564) | -0.017 |
| hsa-miR-98-5p | 2.86E-01 | 1.00E+00 | 1.17E-01 | 0.524 (0.483; 0.565) | 0.038 |
| hsa-miR-1249-3p | 2.92E-01 | 1.00E+00 | 9.79E-02 | 0.522 (0.481; 0.563) | -0.015 |
| hsa-miR-4665-3p | 2.94E-01 | 1.00E+00 | 3.80E-01 | 0.521 (0.48; 0.562) | -0.020 |
| hsa-miR-6124 | 2.98E-01 | 1.00E+00 | 8.87E-01 | 0.514 (0.473; 0.555) | 0.032 |
| hsa-miR-1914-3p | 3.38E-01 | 1.00E+00 | 7.33E-01 | 0.512 (0.447; 0.529) | 0.027 |
| hsa-miR-6824-3p | 3.42E-01 | 1.00E+00 | 2.06E-01 | 0.5 (0.459; 0.541) | 0.013 |
| hsa-miR-7977 | 3.48E-01 | 1.00E+00 | 8.60E-01 | 0.509 (0.468; 0.55) | 0.050 |
| hsa-miR-132-3p | 3.55E-01 | 1.00E+00 | 2.08E-01 | 0.503 (0.462; 0.543) | -0.024 |
| hsa-miR-148b-3p | 3.61E-01 | 1.00E+00 | 5.34E-02 | 0.539 (0.498; 0.58) | 0.028 |
| hsa-miR-574-3p | 3.67E-01 | 1.00E+00 | 3.26E-01 | 0.515 (0.474; 0.556) | -0.025 |
| hsa-miR-1304-3p | 3.77E-01 | 1.00E+00 | 3.06E-01 | 0.523 (0.483; 0.564) | 0.017 |
| hsa-miR-4721 | 3.87E-01 | 1.00E+00 | 8.58E-01 | 0.502 (0.461; 0.543) | 0.039 |
| hsa-miR-3614-5p | 3.90E-01 | 1.00E+00 | 5.00E-01 | 0.502 (0.461; 0.543) | -0.010 |
| hsa-miR-6879-5p | 4.05E-01 | 1.00E+00 | 1.13E-02 | 0.547 (0.507; 0.588) | -0.022 |
| hsa-miR-5100 | 4.11E-01 | 1.00E+00 | 5.30E-01 | 0.5 (0.459; 0.541) | 0.041 |
| hsa-miR-652-3p | 4.12E-01 | 1.00E+00 | 1.37E-01 | 0.541 (0.501; 0.582) | 0.017 |
| hsa-miR-1306-5p | 4.17E-01 | 1.00E+00 | 2.86E-01 | 0.513 (0.472; 0.554) | -0.022 |
| hsa-miR-425-3p | 4.19E-01 | 1.00E+00 | 2.14E-01 | 0.535 (0.495; 0.576) | 0.013 |
| hsa-miR-3135b | 4.46E-01 | 1.00E+00 | 8.49E-01 | 0.515 (0.474; 0.556) | 0.052 |
| hsa-miR-6851-3p | 4.56E-01 | 1.00E+00 | 5.48E-01 | 0.511 (0.47; 0.552) | -0.011 |
| hsa-miR-126-3p | 4.89E-01 | 1.00E+00 | 1.88E-01 | 0.514 (0.473; 0.555) | 0.029 |
| hsa-miR-6740-5p | 5.18E-01 | 1.00E+00 | 3.49E-02 | 0.557 (0.517; 0.598) | -0.018 |
| hsa-miR-5006-5p | 5.23E-01 | 1.00E+00 | 9.25E-01 | 0.502 (0.462; 0.543) | 0.020 |
| hsa-miR-3940-5p | 5.34E-01 | 1.00E+00 | 3.83E-01 | 0.536 (0.496; 0.577) | 0.013 |
| hsa-miR-6870-3p | 5.42E-01 | 1.00E+00 | 4.37E-01 | 0.501 (0.46; 0.541) | 0.006 |
| hsa-miR-4787-3p | 5.45E-01 | 1.00E+00 | 4.25E-01 | 0.515 (0.474; 0.556) | 0.010 |
| hsa-miR-500a-5p | 5.54E-01 | 1.00E+00 | 6.75E-01 | 0.525 (0.484; 0.566) | 0.016 |
| hsa-miR-129-2-3p | 5.88E-01 | 1.00E+00 | 3.16E-01 | 0.508 (0.467; 0.549) | -0.011 |
| hsa-miR-1273g-3p | 6.16E-01 | 1.00E+00 | 5.17E-01 | 0.505 (0.454; 0.536) | 0.018 |
| hsa-miR-6780b-5p | 6.28E-01 | 1.00E+00 | 3.39E-01 | 0.529 (0.488; 0.57) | 0.014 |
| hsa-miR-363-3p | 6.29E-01 | 1.00E+00 | 9.86E-02 | 0.521 (0.48; 0.562) | 0.013 |
| hsa-miR-454-3p | 6.56E-01 | 1.00E+00 | 6.03E-01 | 0.508 (0.467; 0.549) | -0.022 |
| hsa-miR-199a-3p | 6.59E-01 | 1.00E+00 | 2.54E-02 | 0.514 (0.473; 0.555) | 0.022 |
| hsa-miR-1260a | 6.84E-01 | 1.00E+00 | 6.36E-01 | 0.513 (0.472; 0.554) | 0.016 |
| hsa-miR-664b-5p | 7.07E-01 | 1.00E+00 | 3.36E-01 | 0.501 (0.46; 0.542) | 0.021 |
| hsa-miR-8069 | 7.34E-01 | 1.00E+00 | 5.43E-01 | 0.505 (0.454; 0.535) | 0.009 |
| hsa-miR-664a-5p | 7.67E-01 | 1.00E+00 | 2.33E-01 | 0.515 (0.444; 0.526) | 0.009 |
| hsa-miR-628-3p | 7.80E-01 | 1.00E+00 | 9.60E-01 | 0.52 (0.479; 0.561) | 0.006 |
| hsa-miR-610 | 7.96E-01 | 1.00E+00 | 8.05E-01 | 0.503 (0.456; 0.538) | 0.005 |
| hsa-miR-151a-5p | 8.05E-01 | 1.00E+00 | 4.72E-01 | 0.53 (0.489; 0.571) | 0.006 |
| hsa-miR-6752-3p | 8.12E-01 | 1.00E+00 | 9.50E-01 | 0.504 (0.455; 0.536) | -0.003 |
| hsa-miR-129-1-3p | 8.23E-01 | 1.00E+00 | 2.13E-01 | 0.522 (0.481; 0.563) | 0.004 |
| hsa-miR-3176 | 8.64E-01 | 1.00E+00 | 3.40E-01 | 0.519 (0.478; 0.559) | -0.003 |
| hsa-miR-933 | 8.64E-01 | 1.00E+00 | 8.49E-01 | 0.504 (0.463; 0.544) | -0.002 |
| hsa-miR-125a-3p | 8.80E-01 | 1.00E+00 | 2.79E-01 | 0.51 (0.469; 0.551) | -0.003 |
| hsa-miR-4669 | 9.00E-01 | 1.00E+00 | 6.23E-01 | 0.524 (0.483; 0.565) | 0.004 |
| hsa-miR-183-3p | 9.10E-01 | 1.00E+00 | 5.89E-01 | 0.505 (0.454; 0.536) | 0.003 |
| hsa-miR-3156-5p | 9.13E-01 | 1.00E+00 | 1.20E-01 | 0.522 (0.481; 0.563) | -0.003 |
| hsa-miR-340-5p | 9.56E-01 | 1.00E+00 | 6.54E-01 | 0.511 (0.47; 0.552) | -0.002 |

**Table 9: Intersections of differentially expressed miRNAs in distinct cardiovascular phenotypes.** This table details the shared top differentially expressed miRNAs per disease as indicated by an adjusted p-value of 0.05 and an abs(log2 Fold Change) > 0.2 across different cardiovascular conditions. This summary provides insights into the common miRNAs observed in various cardiovascular diseases. This data-driven table mirrors Supplementary Table S3 (literature-based overlaps), enabling comparison between reported and observed patterns. “Number of miRNAs” indicates the total count for each intersection.

| Intersection | miRNAs | Number.of.miRNAs |
| --- | --- | --- |
| CAD:ICM  *Ischemic spectrum; progression from CAD to heart failure* | hsa-miR-301a-3p, hsa-miR-6090, hsa-miR-195-5p, hsa-miR-6087, hsa-miR-424-5p, hsa-miR-6089, hsa-miR-6088, hsa-miR-629-3p, hsa-miR-6800-5p, hsa-miR-6869-5p, hsa-miR-4507, hsa-miR-6125, hsa-miR-4516, hsa-miR-502-5p, hsa-miR-96-5p, hsa-miR-4787-5p, hsa-miR-144-3p, hsa-miR-4530, hsa-miR-18b-5p, hsa-miR-4687-3p, hsa-miR-4659a-3p, hsa-miR-6513-3p, hsa-miR-130b-5p, hsa-miR-4505, hsa-miR-215-5p, hsa-miR-22-5p, hsa-miR-16-5p, hsa-miR-140-5p, hsa-miR-6803-5p, hsa-miR-454-5p, hsa-miR-942-5p, hsa-miR-6749-5p, hsa-miR-21-5p, hsa-miR-3200-5p, hsa-miR-17-3p, hsa-miR-3163, hsa-miR-199a-5p, hsa-miR-4515, hsa-miR-28-5p, hsa-miR-2861, hsa-miR-183-5p, hsa-miR-10a-5p, hsa-miR-362-3p, hsa-miR-550b-2-5p, hsa-miR-148b-5p, hsa-miR-27a-3p, hsa-miR-192-5p, hsa-miR-642b-3p, hsa-miR-4323, hsa-miR-30b-5p, hsa-miR-7976, hsa-miR-423-3p, hsa-miR-24-3p, hsa-miR-326, hsa-miR-99a-5p, hsa-miR-1255b-5p, hsa-miR-340-3p, hsa-miR-762, hsa-miR-7-1-3p, hsa-miR-142-3p, hsa-miR-5194, hsa-miR-328-3p, hsa-miR-182-5p, hsa-miR-590-5p, hsa-miR-26b-3p, hsa-miR-1587, hsa-miR-3665, hsa-miR-15b-5p, hsa-miR-146b-5p, hsa-miR-1271-5p, hsa-miR-5739, hsa-miR-550a-3-5p, hsa-miR-339-5p, hsa-miR-331-3p, hsa-miR-6717-5p, hsa-miR-335-5p, hsa-miR-598-3p, hsa-miR-5690, hsa-miR-766-3p, hsa-miR-3605-3p, hsa-miR-3200-3p, hsa-miR-4317, hsa-miR-4281, hsa-miR-628-5p, hsa-miR-330-3p, hsa-miR-638, hsa-miR-103a-3p, hsa-miR-133b, hsa-miR-107, hsa-miR-26b-5p, hsa-miR-19b-3p, hsa-miR-128-3p, hsa-miR-142-5p, hsa-miR-1246, hsa-miR-4763-3p, hsa-miR-23b-3p, hsa-miR-6727-5p, hsa-miR-30e-3p, hsa-miR-181a-2-3p, hsa-miR-942-3p, hsa-miR-361-5p, hsa-miR-199b-5p, hsa-miR-378a-5p, hsa-miR-5088-5p, hsa-miR-1915-3p, hsa-miR-5581-5p, hsa-miR-5787, hsa-miR-15b-3p, hsa-miR-4442, hsa-miR-625-5p, hsa-miR-6724-5p, hsa-miR-4746-3p, hsa-miR-6779-5p, hsa-miR-664b-3p, hsa-miR-3195, hsa-miR-6085, hsa-miR-641, hsa-miR-1288-3p, hsa-miR-550a-3p, hsa-miR-495-3p, hsa-miR-4716-3p, hsa-miR-3679-5p, hsa-let-7b-5p, hsa-miR-139-5p, hsa-miR-937-5p, hsa-miR-191-5p, hsa-miR-338-3p, hsa-miR-654-3p, hsa-miR-629-5p, hsa-miR-99b-5p, hsa-miR-6068, hsa-miR-106b-5p, hsa-miR-501-3p, hsa-miR-7107-5p, hsa-miR-502-3p, hsa-miR-296-5p, hsa-miR-4466, hsa-miR-1207-5p, hsa-miR-3940-3p, hsa-miR-223-3p, hsa-miR-320d, hsa-miR-146a-5p, hsa-miR-125b-5p, hsa-miR-181a-5p, hsa-miR-485-3p, hsa-miR-4433a-5p, hsa-miR-6757-5p, hsa-miR-500a-3p, hsa-miR-4299, hsa-miR-130b-3p, hsa-miR-320c, hsa-miR-4769-3p, hsa-miR-7152-3p, hsa-miR-4653-3p, hsa-miR-6767-5p | 155 |
| CAD:DCM:ICM  *Pan-cardiomyopathy; cardiac remodeling across etiologies* | hsa-miR-374a-5p, hsa-miR-374b-5p, hsa-miR-660-5p, hsa-miR-4443, hsa-miR-4291, hsa-miR-93-3p, hsa-miR-139-3p, hsa-miR-3651, hsa-miR-130a-3p, hsa-let-7e-5p, hsa-miR-4449, hsa-miR-7-5p, hsa-miR-532-3p, hsa-let-7f-5p, hsa-miR-150-5p, hsa-let-7g-5p, hsa-miR-30d-5p, hsa-miR-222-3p, hsa-miR-145-5p, hsa-miR-361-3p, hsa-let-7a-5p, hsa-miR-4270, hsa-miR-664a-3p, hsa-let-7i-5p, hsa-miR-365a-3p, hsa-miR-342-3p, hsa-miR-6893-5p, hsa-miR-125a-5p, hsa-miR-221-3p, hsa-miR-484, hsa-miR-30c-1-3p, hsa-let-7f-1-3p | 32 |
| DCM:ICM  *Heart failure phenotypes; end-stage systolic dysfunction markers* | hsa-miR-1275, hsa-miR-564, hsa-miR-4465, hsa-miR-4306, hsa-miR-4672, hsa-miR-4284, hsa-miR-6791-5p, hsa-miR-185-5p, hsa-miR-22-3p, hsa-miR-30a-5p, hsa-let-7d-3p | 11 |
| CAD:DCM | hsa-miR-454-3p, hsa-miR-7641, hsa-miR-98-5p, hsa-miR-374c-5p, hsa-miR-500b-5p, hsa-miR-744-5p, hsa-miR-4485-3p, hsa-miR-155-5p, hsa-miR-628-3p, hsa-miR-4484 | 10 |
| ACS:CAD:DCM:ICM  *Pan-cardiovascular* | hsa-miR-17-5p, hsa-miR-93-5p, hsa-miR-126-5p, hsa-miR-6511b-3p, hsa-miR-20b-5p, hsa-miR-20a-5p, hsa-miR-6803-3p, hsa-miR-101-3p, hsa-miR-532-5p | 9 |
| ACS:CAD:ICM  *Ischemic spectrum; acute-to-chronic progression* | hsa-miR-451a, hsa-miR-1202, hsa-miR-7110-5p, hsa-miR-1225-5p, hsa-miR-4732-3p, hsa-miR-15a-5p, hsa-miR-18a-5p, hsa-miR-642a-3p | 8 |
| ACS:CAD:DCM | hsa-miR-4318, hsa-miR-652-3p, hsa-miR-342-5p, hsa-miR-126-3p | 4 |
| ACS:CAD | hsa-miR-664b-5p | 1 |
| ACS:ICM | hsa-miR-625-3p | 1 |

Table 10: ACS risk groups stratified by aPRIORI ACS model probabilities. This table provides the patient demographics and laboratory values stratified by tertiles of probability for ACS derived from the aPRIORI model, analysing only patients from the University Hospital of Heidelberg. ACS, acute coronary syndrome

|  | **Low (n=32)** | **Intermediate (n=32)** | **High (n=32)** | **P-value** |
| --- | --- | --- | --- | --- |
| **AC-Mortality = Died (%)** | 6 (18.8) | 8 (25.0) | 10 (31.2) | 0.437 |
| **Age (mean (SD))** | 68.69 (13.61) | 68.66 (12.19) | 68.09 (13.06) | 0.979 |
| **Gender = Male (%)** | 23 (71.9) | 22 (68.8) | 17 (53.1) | 0.244 |
| **Weight in kg (mean (SD))** | 84.72 (13.51) | 80.83 (15.14) | 76.18 (21.26) | 0.225 |
| **Body Mass Index (mean (SD))** | 28.42 (4.99) | 27.59 (4.28) | 26.32 (5.91) | 0.373 |
| **Smoking = Yes (%)** | 18 (56.2) | 20 (62.5) | 21 (65.6) | 0.735 |
| **Diabetes = Yes (%)** | 10 (31.2) | 11 (34.4) | 13 (40.6) | 0.727 |
| **Hypertension = Yes (%)** | 25 (78.1) | 29 (90.6) | 28 (87.5) | 0.337 |
| **Family History = Yes (%)** | 13 (40.6) | 9 (28.1) | 6 (18.8) | 0.155 |
| **Creatinine (median [IQR])** | 0.88 [0.81, 1.02] | 0.84 [0.77, 1.06] | 0.87 [0.72, 1.11] | 0.748 |
| **hs-TroponinT (median [IQR])** | 95.50 [43.25, 185.50] | 146.00 [48.00, 332.75] | 272.50 [86.50, 842.75] | 0.038 |
| **nt-proBNP (median [IQR])** | 671.00 [196.00, 2,502.00] | 465.00 [94.00, 17,364.00] | 172.00 [87.50, 1,177.75] | 0.687 |
| **Hemoglobin (median [IQR])** | 13.75 [13.05, 14.33] | 13.90 [12.25, 15.10] | 13.10 [10.75, 14.65] | 0.439 |
| **INR (median [IQR])** | 1.03 [1.00, 1.10] | 1.02 [0.99, 1.05] | 1.04 [1.01, 1.09] | 0.355 |
| **Bilirubin (median [IQR])** | 0.60 [0.60, 0.85] | 0.70 [0.60, 0.92] | 0.50 [0.40, 0.80] | 0.141 |
| **Leukocytes (median [IQR])** | 8.73 [7.34, 10.14] | 10.51 [8.78, 13.17] | 8.10 [6.58, 11.27] | 0.053 |
| **CRP (median [IQR])** | 14.40 [8.95, 37.05] | 21.90 [9.30, 80.70] | 13.70 [8.85, 35.77] | 0.693 |
| **Cholesterol (median [IQR])** | 178.00 [145.00, 226.50] | 182.00 [171.00, 239.00] | 185.00 [161.00, 219.00] | 0.727 |
| **aPRIORI ACS Probability (mean (SD))** | **0.42 (0.14)** | **0.72 (0.07)** | **0.90 (0.05)** | **<0.001** |

Table 11: DCM risk groups stratified by aPRIORI DCM model probabilities. This table provides the patient demographics and laboratory values stratified by tertiles of probability for DCM derived from the aPRIORI model, analysing only patients from the University Hospital of Heidelberg. DCM, dilated cardiomyopathy

|  | **Low (n=28)** | **Intermediate (n=27)** | **High (n=27)** | **P-value** |
| --- | --- | --- | --- | --- |
| **AC-Mortality = Died (%)** | 3 (10.7) | 3 (11.1) | 9 (33.3) | 0.022 |
| **Age (mean (SD))** | 65.39 (12.80) | 59.93 (16.59) | 54.78 (16.36) | 0.042 |
| **Gender = Male (%)** | 21 (75.0) | 19 (70.4) | 23 (85.2) | 0.418 |
| **Weight in kg (mean (SD))** | 88.25 (20.71) | 82.42 (16.21) | 93.68 (32.55) | 0.253 |
| **Body Mass Index (mean (SD))** | 28.08 (4.71) | 26.38 (4.94) | 29.77 (9.03) | 0.191 |
| **Smoking = Yes (%)** | 12 (42.9) | 14 (51.9) | 13 (48.1) | 0.798 |
| **Diabetes = Yes (%)** | 7 (25.0) | 6 (22.2) | 10 (37.0) | 0.435 |
| **Hypertension = Yes (%)** | 16 (57.1) | 18 (66.7) | 17 (63.0) | 0.763 |
| **Family History = Yes (%)** | 7 (25.0) | 10 (37.0) | 5 (18.5) | 0.297 |
| **Creatinine (median [IQR])** | 1.00 [0.90, 1.11] | 0.94 [0.87, 1.17] | 1.03 [0.82, 1.25] | 0.831 |
| **hs-TroponinT (median [IQR])** | 17.50 [12.25, 25.50] | 14.00 [9.00, 19.00] | 13.50 [9.75, 15.75] | 0.709 |
| **nt-proBNP (median [IQR])** | 1,058.50 [364.50, 2,071.25] | 490.00 [164.00, 1,304.00] | 1,618.00 [753.50, 9,441.49] | 0.123 |
| **Hemoglobin (median [IQR])** | 13.80 [13.00, 15.35] | 14.30 [12.80, 15.35] | 13.10 [11.95, 15.30] | 0.722 |
| **INR (median [IQR])** | 1.02 [0.99, 1.05] | 1.04 [0.98, 1.09] | 1.04 [1.00, 1.19] | 0.754 |
| **Bilirubin (median [IQR])** | 0.65 [0.52, 0.78] | 0.50 [0.40, 1.00] | 0.85 [0.70, 1.30] | 0.252 |
| **Leukocytes (median [IQR])** | 7.20 [6.15, 8.89] | 7.23 [6.37, 9.66] | 7.38 [6.12, 9.32] | 0.935 |
| **CRP (median [IQR])** | 13.20 [7.10, 25.70] | 9.15 [7.30, 11.15] | 14.55 [13.60, 36.65] | 0.114 |
| **Cholesterol (median [IQR])** | 170.00 [164.75, 193.25] | 180.50 [158.50, 222.00] | 158.00 [141.50, 167.00] | 0.326 |
| **Ejection Fraction (mean (SD))** | 33.86 (13.95) | 30.37 (14.06) | 23.94 (10.36) | 0.092 |
| **aPRIORI DCM Probability (mean (SD))** | **0.16 (0.06)** | **0.36 (0.08)** | **0.69 (0.14)** | **<0.001** |

Table 12: CAD risk groups stratified by aPRIORI DCM model probabilities. This table provides the patient demographics and laboratory values stratified by tertiles of probability for CAD derived from the aPRIORI model, analysing only patients from the University Hospital of Heidelberg. CAD,, coronary artery disease

|  | **Low (n=91)** | **Intermediate (n=91)** | **High (n=91)** | **P-value** |
| --- | --- | --- | --- | --- |
| **AC-Mortality = Died (%)** | ﻿11 (12.1) | 23 (25.3) | 22 (24.2) | 0.049 |
| **Age (mean (SD))** | 69.18 (11.11) | 69.51 (9.69) | 71.29 (10.93) | 0.353 |
| **Gender = Male (%)** | 62 (68.1) | 72 (79.1) | 75 (82.4) | 0.059 |
| **Weight in kg (mean (SD))** | 80.27 (14.35) | 82.93 (17.70) | 84.86 (18.90) | 0.211 |
| **Body Mass Index (mean (SD))** | 27.32 (4.19) | 27.57 (4.65) | 28.37 (5.32) | 0.319 |
| **Smoking = Yes (%)** | 43 (47.3) | 51 (56.0) | 50 (54.9) | 0.433 |
| **Diabetes = Yes (%)** | 34 (37.4) | 36 (39.6) | 30 (33.0) | 0.643 |
| **Hypertension = Yes (%)** | 71 (78.0) | 72 (79.1) | 75 (82.4) | 0.744 |
| **Family History = Yes (%)** | 30 (33.0) | 30 (33.0) | 20 (22.0) | 0.171 |
| **Creatinine (median [IQR])** | 0.92 [0.79, 1.03] | 0.93 [0.82, 1.09] | 1.11 [0.87, 1.54] | <0.001 |
| **hs-TroponinT (median [IQR])** | 11.00 [8.00, 16.00] | 13.00 [8.00, 20.00] | 18.00 [12.00, 31.00] | 0.009 |
| **nt-proBNP (median [IQR])** | 178.00 [84.00, 720.00] | 190.00 [94.00, 629.25] | 401.00 [101.50, 899.00] | 0.773 |
| **Hemoglobin (median [IQR])** | 13.50 [12.83, 15.05] | 13.90 [12.85, 14.95] | 13.35 [11.78, 14.22] | 0.179 |
| **INR (median [IQR])** | 1.00 [0.97, 1.06] | 1.06 [0.98, 1.15] | 1.05 [0.99, 1.18] | 0.055 |
| **Bilirubin (median [IQR])** | 0.60 [0.50, 0.80] | 0.70 [0.60, 0.80] | 0.70 [0.55, 1.05] | 0.200 |
| **Leukocytes (median [IQR])** | 7.01 [5.96, 8.17] | 7.21 [6.14, 8.80] | 6.39 [5.08, 8.55] | 0.628 |
| **CRP (median [IQR])** | 4.90 [3.55, 11.95] | 9.10 [3.35, 16.85] | 4.30 [3.42, 6.42] | 0.346 |
| **Cholesterol (median [IQR])** | 173.50 [150.50, 204.50] | 153.00 [135.25, 196.50] | 160.00 [134.00, 215.00] | 0.173 |
| **aPRIORI CAD Probability (mean (SD))** | **0.39 (0.15)** | **0.74 (0.07)** | **0.93 (0.04)** | **<0.001** |

Table 13: CAD risk groups stratified by aPRIORI CAD model probabilities. This table provides the patient demographics and laboratory values stratified by median probability values for CAD derived from the aPRIORI model, analysing only patients from the University Hospital of Heidelberg. CAD, coronary artery disease

|  | **Low (n=137)** | **High (n=136)** | **P-value** |
| --- | --- | --- | --- |
| **AC-Mortality = Died (%)** | ﻿20 (14.6) | 36 (26.5) | 0.013 |
| **Age (mean (SD))** | 69.38 (10.39) | 70.60 (10.80) | 0.341 |
| **Gender = Male (%)** | 100 (73.0) | 109 (80.1) | 0.210 |
| **Weight in Kg (mean (SD))** | 80.78 (15.42) | 84.66 (18.60) | 0.069 |
| **Body Mass Index (mean (SD))** | 27.30 (4.37) | 28.22 (5.08) | 0.124 |
| **Smoking = Yes (%)** | 66 (48.2) | 78 (57.4) | 0.162 |
| **Diabetes = Yes (%)** | 48 (35.0) | 52 (38.2) | 0.672 |
| **Hypertension = Yes (%)** | 107 (78.1) | 111 (81.6) | 0.567 |
| **Family History = Yes (%)** | 42 (30.7) | 38 (27.9) | 0.719 |
| **Creatinine (median [IQR])** | 0.92 [0.78, 1.03] | 1.05 [0.86, 1.32] | <0.001 |
| **hs-TroponinT (median [IQR])** | 12.00 [7.25, 16.00] | 16.00 [10.00, 29.25] | 0.011 |
| **nt-proBNP (median [IQR])** | 172.50 [74.75, 623.25] | 318.50 [105.00, 865.00] | 0.276 |
| **Hemoglobin (median [IQR])** | 13.60 [12.80, 15.10] | 13.65 [12.47, 14.65] | 0.301 |
| **INR (median [IQR])** | 1.01 [0.97, 1.11] | 1.06 [0.99, 1.18] | 0.031 |
| **Bilirubin (median [IQR])** | 0.60 [0.50, 0.80] | 0.70 [0.50, 0.90] | 0.141 |
| **Leukocytes (median [IQR])** | 7.25 [5.89, 8.34] | 6.88 [5.62, 8.53] | 0.393 |
| **CRP (median [IQR])** | 6.50 [3.55, 12.85] | 5.00 [3.40, 12.50] | 0.800 |
| **Cholesterol (median [IQR])** | 170.00 [143.00, 206.00] | 157.50 [136.00, 198.50] | 0.282 |
| **aPRIORI CAD Probability (mean (SD))** | **0.49 (0.18)** | **0.89 (0.07)** | **<0.001** |

**Table 14: Diagnostic performance of miRNAs and combined models with NT-proBNP in all DCM patients.** Performance metrics for individual miRNAs, the DCM aPRIORI miRNA signature, and combined NT-proBNP + miRNA models are shown. Incremental Δ represents the absolute AUC gain of the combined model compared with NT-proBNP alone. P-values were obtained from likelihood-ratio (χ²) tests comparing nested logistic regression models.

| **miRNA** | **miRNA AUC** | **Combined NTproBNP AUC** | **Incremental Δ** | **P-value** |
| --- | --- | --- | --- | --- |
| **miRNA Signature** | 0.899 | 0.978 | 0.043 | <0.001 |
| **hsa-miR-3651** | 0.699 | 0.958 | 0.193 | <0.001 |
| **hsa-miR-150-5p** | 0.697 | 0.958 | 0.226 | <0.001 |
| **hsa-miR-652-3p** | 0.690 | 0.950 | 0.207 | <0.001 |
| **hsa-miR-139-3p** | 0.681 | 0.958 | 0.212 | <0.001 |
| **hsa-miR-1275** | 0.675 | 0.953 | 0.216 | <0.001 |
| **hsa-miR-664a-3p** | 0.673 | 0.963 | 0.222 | <0.001 |
| **hsa-miR-564** | 0.668 | 0.950 | 0.214 | <0.001 |
| **hsa-miR-186-5p** | 0.668 | 0.942 | 0.233 | <0.001 |
| **hsa-miR-30d-5p** | 0.665 | 0.951 | 0.210 | <0.001 |

**Footnote: *N* = 959 patients; NT-proBNP alone AUC = 0.926.**
